# Lignanamides from the Roots of *Metternichia macrocalyx* and Their Anti-inflammatory Activity

**DOI:** 10.1021/acsomega.4c07336

**Published:** 2024-11-13

**Authors:** Thiago Araújo de Medeiros Brito, Ana Carolina Ferreira de Albuquerque, Fernando Martins dos Santos Junior, Paulo Bruno Araújo Loureiro, Francisco Allysson Assis Ferreira Gadelha, Marianna Vieira Sobral, Domingos Benício Oliveira Silva Cardoso, Eudes da Silva Velozo, Josean Fechine Tavares, Lucas Silva Abreu, Marcelo Sobral da Silva

**Affiliations:** † Laboratório Multiusuário de Caracterização e Análises, Programa de Pós-Graduação em Produtos Naturais e Sintéticos Bioativos, Centro de Ciências da Saúde, Universidade Federal da Paraíba, João Pessoa 58051-900, Paraíba, Brazil; ‡ Departamento de Química Orgânica, Instituto de Química, Universidade Federal Fluminese, Niterói 24020-141, Rio de Janeiro, Brazil; § Laboratório de Oncofarmacologia, Programa de Pós-Graduação em Produtos Naturais e Sintéticos Bioativos, Centro de Ciências da Saúde, Universidade Federal da Paraíba, João Pessoa 58051-900, Paraíba, Brazil; ∥ Laboratório de Pesquisa em Matéria Médica, Departamento do Medicamento, Faculdade de Farmácia, Universidade Federal da Bahia, Salvador 40170-115, Bahia, Brazil; ⊥ Instituto de Biologia, Universidade Federal da Bahia, Salvador 40170-115, Bahia, Brazil

## Abstract

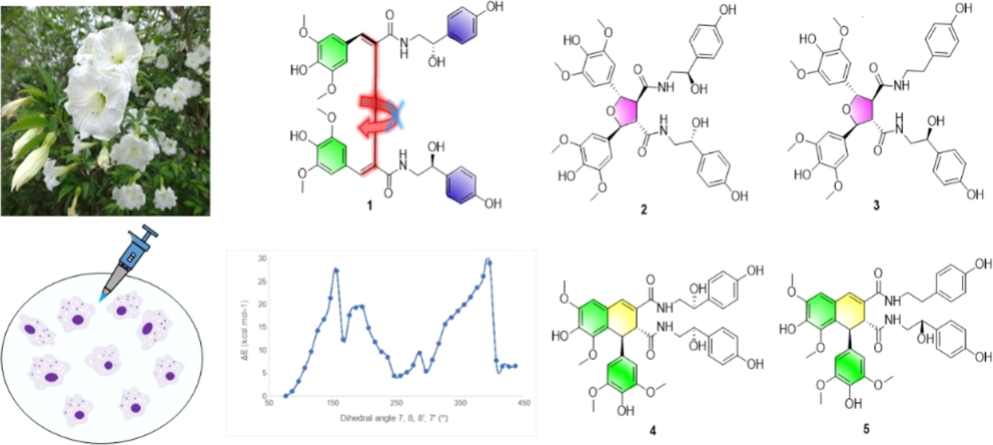

Five new lignanamides (**1**–**5**) and ten known amides (**6**–**15**) were isolated from the chloroform extract of *Metternichia macrocalyx* roots. The structures of the new compounds were elucidated via analysis of NMR spectroscopic and mass spectrometry data and known structures by comparison with data from the literature. Compound **1** had its absolute configuration established through ECD experiments, NMR calculations and quantum mechanical calculations. The anti-inflammatory activity of compounds (**1**–**5**) was assessed on RAW 264.7 macrophages. All the tested compounds reduced nitric oxide levels. In the cytokine quantification assays, only compound **2** did not significantly reduce IL-10 levels. Moreover, compounds **1**–**3** and **5** also reduced IL-1β levels. These results suggest the anti-inflammatory potential of these compounds.

*Metternichia* J. C. Mikan (Solanaceae) was historically a monospecific genus, formed by two native varieties from geographically contrasting environments: a typical variety (*M. princeps* var. *princeps*), found in the humid forests of eastern Brazil (Atlantic Forest region) and *M. princeps* macrocalyx Carv., from semiarid areas (Caatinga region). Recently, the variety *M. princeps* macrocalyx Carv. was elevated to species status with the new combination *Metternichia macrocalyx*.^1^

The species of the Solanaceae family are producers of biologically active compounds,^2^ among them, lignanamides consist of a subclass of lignans first discovered in the roots of *Capsicum annuum* var. *grossum* (Solanaceae).^3^ Sequentially, other lignanamides have been identified through phytochemical investigations of several members of the Solanaceae family, including: *Hyocyamus niger*,^4^*Solanum melongena*,^5^*Solanum tuberosum*,^6^*Lycium yunnanense*,^7^ and *Lycium chinense*.^8^ These metabolites have demonstrated anti-inflammatory activity *in vivo* and *in vitro* models.^5,9,10^ In our ongoing study with Brazilian species from semiarid regions, we report the isolation and structural determination of five new lignanamides (**1**–**5**), along with ten known amides (**6**–**15**) and the evaluation of their anti-inflammatory activity through *in vitro* tests.

## Results and Discussion

**Chart 1:**
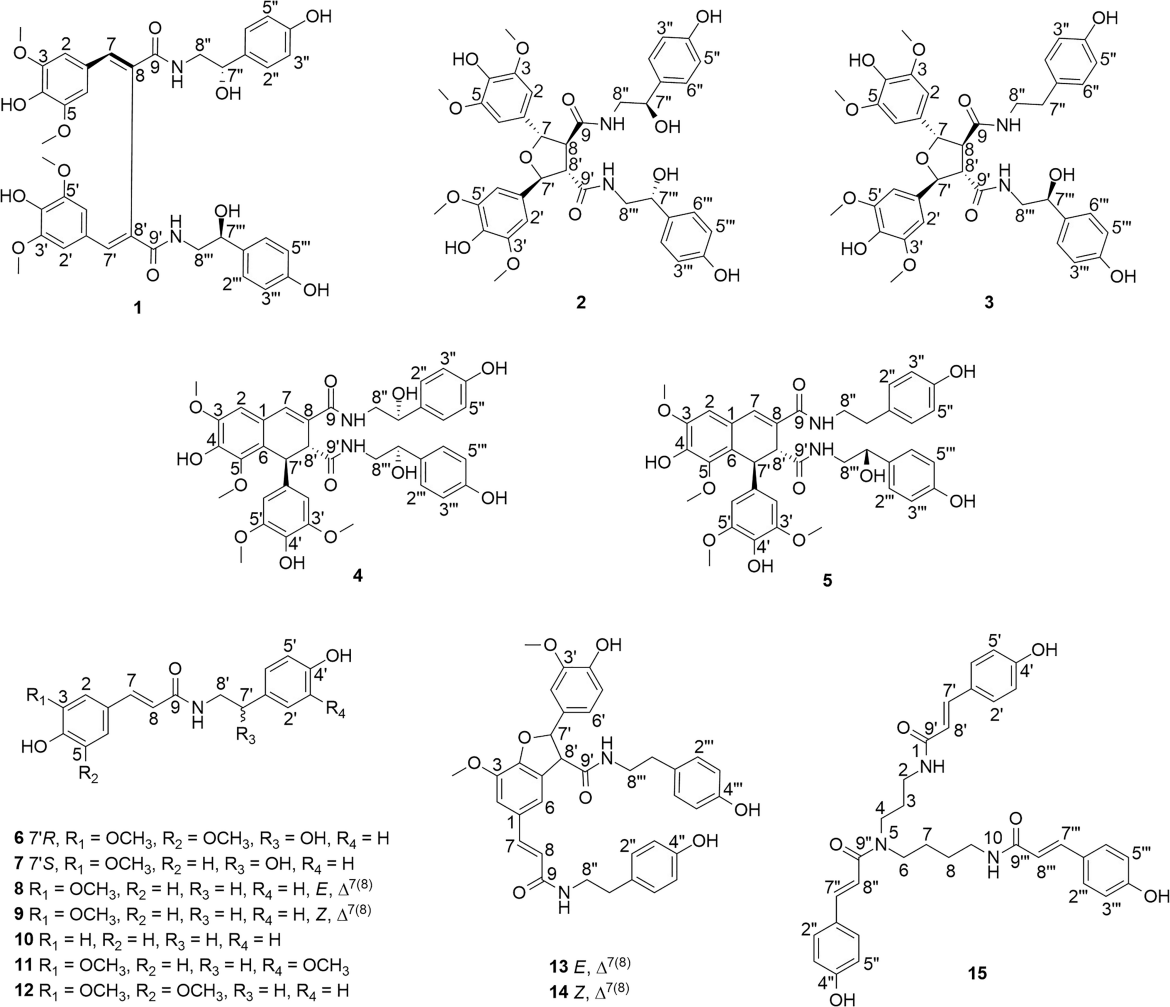
Chemical Structures of Isolated Compounds **1**–**15** from the Roots of *M. macrocalyx*

The chloroform extract of *M. macrocalyx* roots was subjected to fractionation via medium pressure liquid chromatography (MPLC) using silica gel 60 (70–230 mesh), followed by HPLC. These processes resulted in the isolation of five new lignanamides (**1**–**5**) and ten known amides (**6**–**15**). In addition, we evaluated their anti-inflammatory activities through *in vitro* tests. The structures were characterized using ^1^H and ^13^C 1D and 2D NMR spectroscopy, HRESIMS, NMR calculations, electronic circular dichroism (ECD), specific optical rotation and infrared spectroscopy (IR). The known compounds *N-trans*-sinapoyloctopamine (**6**),^11^*N-trans*-feruloyloctopamine (**7**),^11^*N-trans*-feruloyltyramine (**8**),^11^*N-cis*-feruloyltyramine (**9**),^11^*N-trans*-coumaroyltyramine (**10**),^12^*N-trans*-feruloyl-3-methoxytyramine (**11**),^11^*N-trans*-sinapoyltyramine (**12**),^11,13^*N-trans*-grossamide (**13**),^6^*N-cis*-grossamide (**14**),^6^ and *N*^1^,*N*^5^,*N*^10^-Tri*-p*-coumaroylspermidine (**15**)^14,15^ were identified by comparing their spectroscopic data (Figures S54–S100) with those reported in the literature. Additionally, the ECD spectrum (Figure 3) established the absolute configuration of compound **6**, which was isolated for the first time in *Solanum melongena* as *R-N-trans-*sinapoyloctopamine. (Chart 1).

Compound **1** was isolated as a yellowish amorphous powder. Its molecular formula was established as C_38_H_41_N_2_O_12_ based on HRESIMS [M + H]^+^ at *m*/*z* 717.2626, calcd 717.2654, Δ = 3.9 ppm, with the hydrogen deficiency index (HDI) equal to 20; (Figure S9). The infrared spectrum showed absorptions at 3343 cm^–1^ (hydroxyl), 1651 cm^–1^ (amide carbonyl), 1611 and 1514 cm^–1^ (C=C of aromatic ring). The ^1^H NMR spectrum (Figures S1–S3) showed signals at δ_H_ 7.82 (1H, brs, H-7) and 7.86 (1H, brs, H-7′) characteristic of olefin protons in α,β-conjugated systems, signals of oxymethine protons at δ_H_ 4.38 (H-7‴) and 4.54 (H-7″), in addition to a set aromatic proton signals at δ_H_ 6.87 (brs, 4H), δ_H_ 6.94 (d, 2H, *J* = 8.5 Hz), δ_H_ 7.03 (d, 2H, *J* = 8.4 Hz), δ_H_ 6.63 (d, 2H, *J* = 8.5 Hz) and δ_H_ 6.69 (d, 2H, *J* = 8.4 Hz) indicating the presence of two pairs of 1,3,4,5-tetrasubstituted and 1,4-disubstituted aromatic rings (Table 1). The ^13^C-APT NMR spectrum (Figure S4) showed signals of olefinic carbons at δ_C_ 142.3 (C-7), and 142.5 (C-7′), 127.4 (C-8), and 127.6 (C-8′), signals of the oxygenated methine carbons at δ_C_ 73.0 (C-7″), 73.5 (C-7‴), in addition to signals of methine carbons at δ_C_ 48.5 (C-8″,8‴) and carbonyls at δ_C_ 168.2 (C-9,9′). In the HSQC spectrum (Figure S5), the correlation signals at δ_H_ 7.82/δ_C_ 142.3 (H-7/C-7) and at δ_H_ 7.86/δ_C_ 142.5 (H-7′/C-7′) confirmed the presence of two trisubstituted double bonds. These data were compatible with the presence of two sinapoyl and octopamine moieties (Tables 1 and 2). The molecular formula of compound **1** was consistent with a bis-phenylpropene-type lignanamide formed by two *N-trans*-sinapoyloctopamine monomers, similar to cannabisin G.^4,16^

**Table 1:** ^1^H NMR Spectroscopic Data for Compounds **1**–**5** in MeOD

	**1**^a^	**2**^a^	**3**^a^	**4**^a^	**5**^b^
position	δ_H_ (*J* in Hz)	δ_H_ (*J* in Hz)	δ_H_ (*J* in Hz)	δ_H_ (*J* in Hz)	δ_H_ (*J* in Hz)
1					
2	6.87, brs	6.73, s	6.71, s	6.77, brs	6.76, brs
3					
4					
5					
6	6.87, brs	6.73, s	6.71, s		
7	7.82, brs	5.27, d (6.3)	5.26, d (6.6)	7.33, brs	7.27, brs
8		3.38, dd (6.3, 2.8)	3.35, m		
9					
1′					
2′	6.87, brs	6.73, s	6.73, s	6.33, s	6.33, s
3′					
4′					
5′					
6′	6.87, brs	6.73, s	6.73, s	6.33, s	6.33, s
7′	7.86, brs	5.29, d (6.3)	5.28, d (6.4)	4.87, brs	4.86, brs
8′		3.38, dd (6.3, 2.8)	3.40, m	3.72, d (1.8)	3.72, d (1.3)
9′					
1″					
2″	6.94 d (8.5)	6.96, d (8.5)	6.83, d (8.5)	7.14, d (8.6)	6.94, d (8.5)
3″	6.63 d (8.5)	6.66, d (8.5)	6.63, d (8.5)	6.72, d (8.6)	6.66, d (8.5)
4″					
5″	6.63 d (8.5)	6.66 d (8.5)	6.63, d (8.5)	6.72, d (8.6)	6.66, d (8.5)
6″	6.94 d (8.5)	6.96 d (8.5)	6.83, d (8.5)	7.14, d (8.6)	6.94, d (8.5)
7″	4.54, m	4.49 t (6.5)		4.68 dd (7.6, 5.1)	2.68, t (7.2)
7a″			2.59, m		
7b″			2.53, m		
8″					3.33–3.43, m
8a″	3.40, m	3.45, dd (13.3, 6.5)	3.37, m	3.39–3.42, m	
8b″	3.40, m	3.22, dd (13.3, 6.5)	3.18, m	3.39–3.42, m	
1‴					
2‴	7.03 d (8.4)	6.96, d (8.5)	6.95, d (8.5)	6.96, d (8.6)	6.83, d (8.5)
3‴	6.69 d (8.4)	6. 66, d (8.5)	6.66, d (8.5)	6.67, d (8.6)	6.63, d (8.5)
4‴					
5‴	6.69 d (8.4)	6.66 d (8.5)	6.66, d (8.5)	6.67, d (8.6)	6.63, d (8.5)
6‴	7.03 d (8.4)	6.96 d (8.5)	6.95, d (8.5)	6.96, d (8.6)	6.83, d (8.5)
7‴	4.38, m	4.49 t (6.5)	4.46, t (6.7)	4.53, t (6.2)	4.52, t (6.2)
8a‴	3.37, m	3.45, dd (13.3, 6.5)	3.49, dd (13.3, 6.7)	3.34–3.37, m	3.33–3.38, m
8b‴	3.37, m	3.22, dd (13.3, 6.5)	3.18, dd (13.3, 6.7)	3.25, dd (6.0, 1.6)	3.26, dd (13.4, 6.0)
OCH_3_-3′, 5′	3.75, s	3.87, s	3.86, s	3.68, s	3.70, s
OCH_3_-3	3.75, s	3.87, s	3.88, s	3.92, s	3.92, s
OCH_3_-5	3.75, s	3.87, s	3.88, s	3.58, s	3.53, s

a Measured at 400 MHz.

b Measured at 500 MHz.

**Table 2:** ^13^C NMR Spectroscopic Data for Compounds **1**–**5** in MeOD

	**1**^a^	**2**^a^	**3**^a^	**4**^a^	**5**^b^
position	δ_C_	δ_C_	δ_C_	δ_C_	δ_C_
1	126.7, C	132.3, C	131.0, C	124.2, C	124.2, C
2	108.8, CH	104.8, CH	104.7, CH	109.1, CH	109.1, CH
3	139.0, C	149.4, C	149.5, C	149.3, C	149.3, C
4	149.2, C	136.6, C	136.7, C	143.3, C	143.2, C
5	139.0, C	149.4, C	149.5, C	146.9, C	147.0, C
6	108.8, CH	104.8, CH	104.7, CH	125.2, C	125.3, C
7	142.3, CH	86.3, CH	86.4, CH	135.4, CH	135.0, CH
8	127.4, C	60.3, CH	60.6, CH	126.8, C	127.1, C
9	168.2, C	172.5, C	172.1, C	170.3, C	170.0, C
1′	126.7, C	132.3, C	132.4, C	135.3, C	135.2, C
2′	108.9, CH	104.8, CH	104.9, CH	106.0, CH	106.1, CH
3′	139.1, C	149.4, C	149.4, C	149.0, C	149.0, C
4′	149.2, C	136.6, C	136.7, C	135.3, C	135.3, C
5′	139.1, C	149.4, C	149.4, C	149.0, C	149.5, C
6′	108.9, CH	104.8, CH	104.9, CH	106.0, CH	106.1, CH
7′	142.5, CH	86.3, CH	86.4, CH	41.4, CH	41.4, CH
8′	127.6, C	60.3, CH	60.3, CH	50.2, CH	50.1, CH
9′	168.2, C	172.5, C	172.5, C	174.3, C	174.2, C
1″	134.2, C	134.2, C	130.8, C	134.7, C	131.4, C
2″	128.4, CH	128.5, CH	130.7, CH	128.4, CH	130.7, CH
3″	116.0, CH	116.1, CH	116.2, CH	116.1, CH	116.2, CH
4″	158.0, C	158.0, C	156.9, C	158.0, C	156.8, C
5″	116.0, CH	116.1, CH	116.2, CH	116.1, CH	116.2, CH
6″	128.4, CH	128.5, CH	130.7, CH	128.4, CH	130.7, CH
7″	73.0, CH	73.1, CH	35.7, CH_2_	73.4, CH	35.6, CH_2_
8″	48.5, CH_2_	48.1, CH_2_	42.6, CH_2_	48.4, CH_2_	42.8, CH_2_
1‴	134.3, C	134.2, C	134.5, C	134.3, C	134.4, C
2‴	128.3, CH	128.5, CH	128.5, CH	128.4, CH	130.8, CH
3‴	116.1, CH	116.1, CH	116.1, CH	116.1, CH	116.2, CH
4‴	158.0, C	158.0, C	158.0, C	157.9, C	156.9, C
5‴	116.1, CH	116.1, CH	116.1, CH	116.1, CH	116.2, CH
6‴	128.3, CH	128.5, CH	128.5, CH	128.4, CH	130.8, CH
7‴	73.5, CH	73.1, CH	73.2, CH	72.8, CH	72.8, CH
8‴	48.5, CH_2_	48.1, CH_2_	48.1, CH_2_	48.0, CH_2_	47.9, CH_2_
OCH_3_-3′, 5′	56.7, CH_3_	56.8, CH_3_	56.9, CH_3_	56.7, CH_3_	56.7, CH_3_
OCH_3_-3	56.7, CH_3_	56.8, CH_3_	56.8, CH_3_	56.8, CH_3_	56.8, CH_3_
OCH_3_-5	56.7, CH_3_	56.8, CH_3_	56.8, CH_3_	60.8, CH_3_	60.8, CH_3_

a Measured at 100 MHz.

b Measured at 125 MHz.

**Figure 1:**
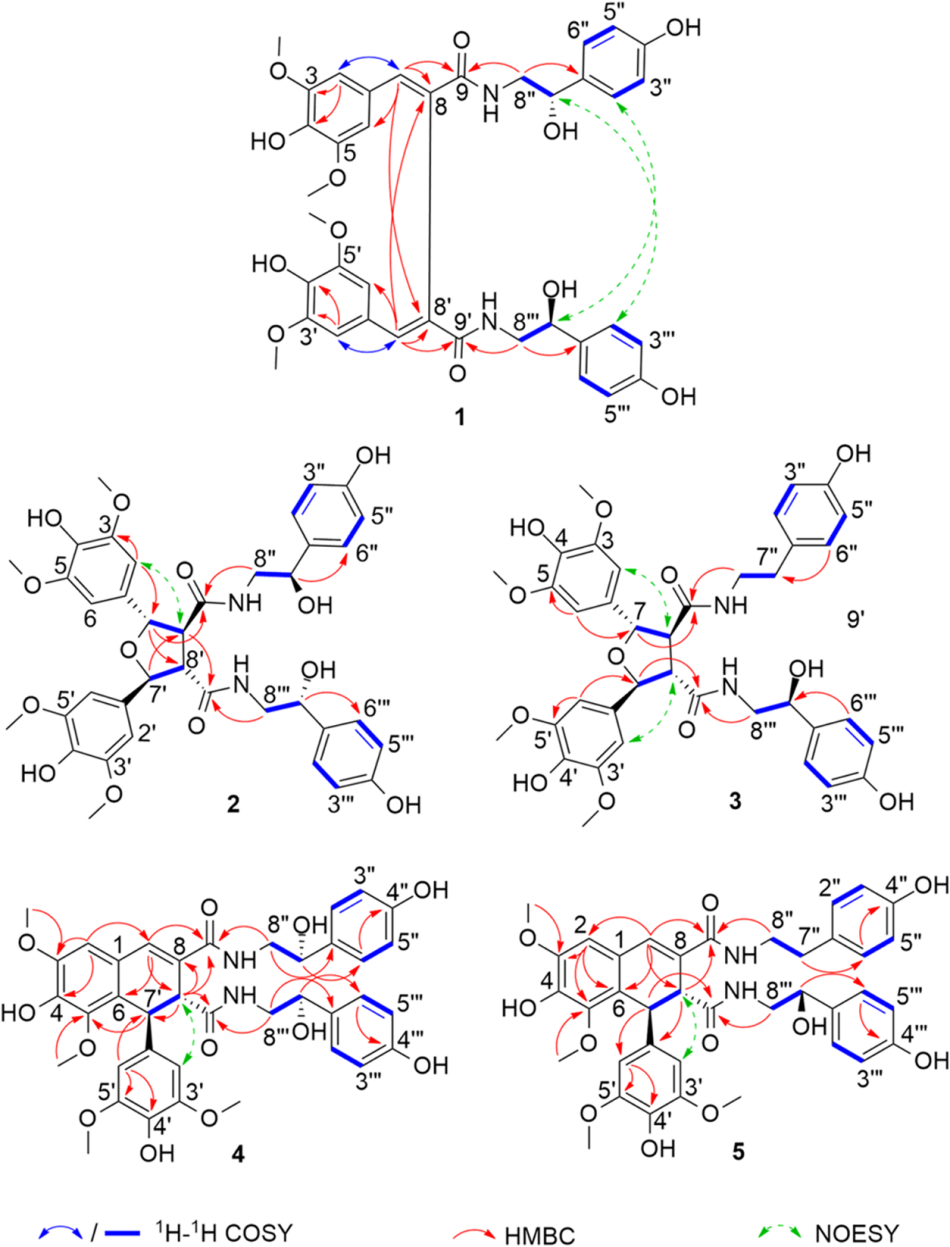
Key HMBC correlations, ^1^H–^1^H COSY, and NOESY of compounds **1**–**5**.

**Figure 2:**
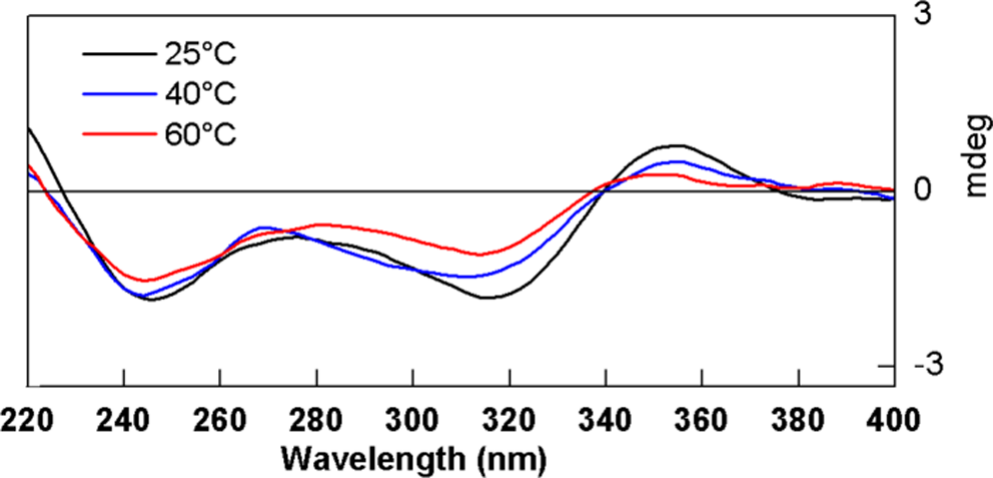
Comparison of the experimental ECD spectra of compound **1** at different temperatures: 25 °C (black), 40 °C (blue), and 60 °C (red).

**Figure 3:**
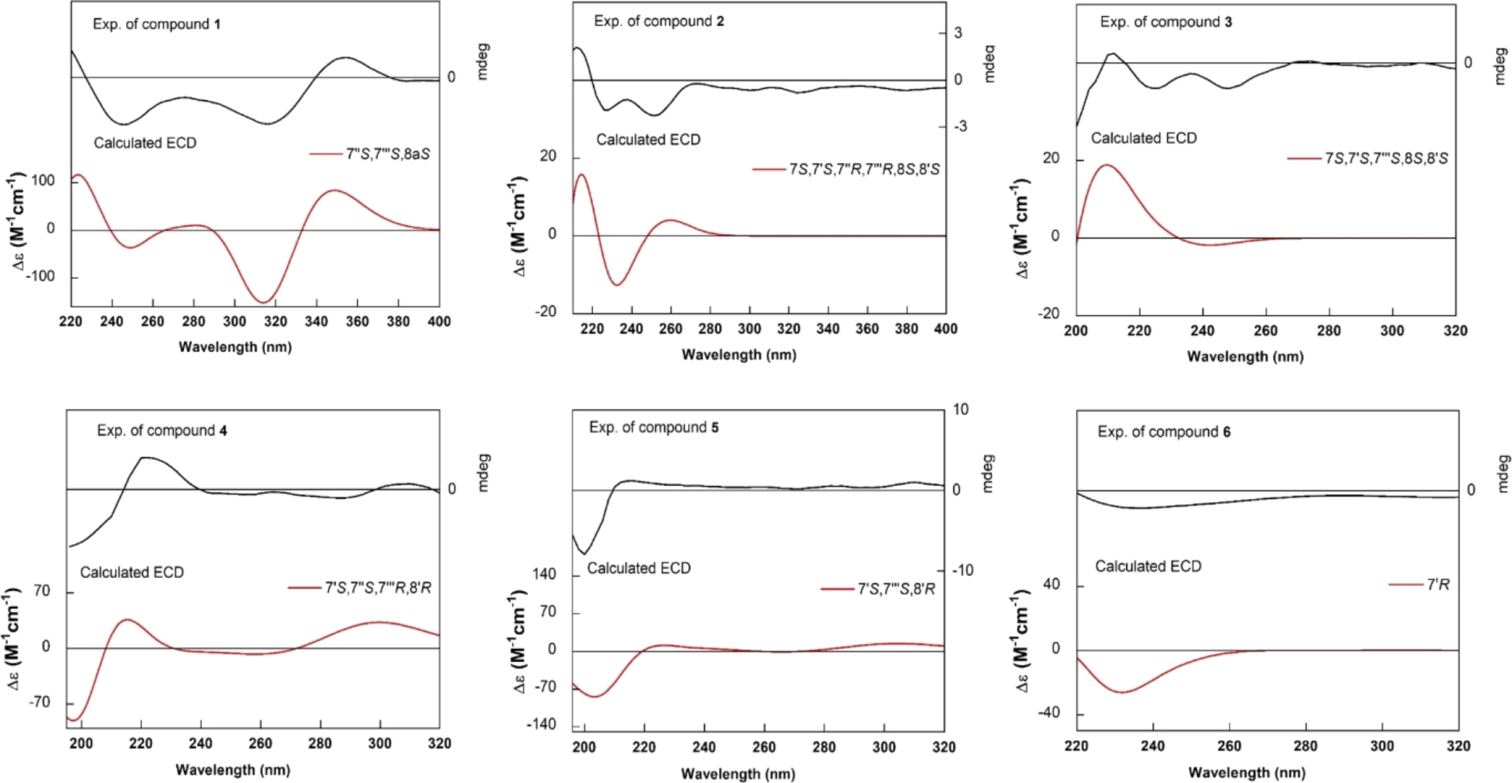
Experimental (top) and calculated (bottom) ECD spectra for compounds **1**–**6**.

In the HMBC spectrum (Figures 1 and S6), the mutual correlations between the proton signals at δ_H_ 7.82 (H-7) and δ_H_ 7.86 (H-7′) with the signals of the aromatic methine carbons at δ_C_ 108.8 (C-2,6), δ_C_ 108.9 (C-2′,6′), with the signals of the unhydrogenated olefinic carbons at δ_C_ 127.4 (C-8), and 127.6 (C-8′) and with the carbonyl carbon signal at δ_C_ 168.2 (C-9,9′) confirmed the chemical shifts of H-7 and H-7′. These correlations, together with those of ^4^*J*_HH_ COSY (see Figures 1 and S7) (H-7/H-2, 6 and H-7′/H-2′,6′), corresponding to benzylic coupling, and NOESY spectra (Figure S8) (H-2″,6″/H-7‴ and H-2‴,6‴/H-7″), were in line with the union of the two monomer units through the carbons C-8 and C-8′. In the ^1^H NMR spectrum, paired signals of different intensities (δ_H_ 7.819 and 7.857, 7.810 and 7.865), suggested axial chirality due to impeded rotation in the C-8-C-8′ axis, which is compatible with atropisomerism. Additionally, the chemical shifts of the oxymethine protons (δ_H_ 4.38 and 4.54) together with the signal pairs for the systems AA′,BB′ (Table 1) of the octopamine units were compatible with stereogenic centers in C-7″ and C7‴. Thus, quantum mechanical calculations on the free energy barrier as a function of rotation around the dihedral angle C7-C8-C8′-C7′ (Figure S101) established the relative free energy value of 29.04 kcal mol^–1^, confirming the presence of axial chirality. For compound **1**, eight stereoisomers were possible: (7″*R*,7‴*R*,8a*S*), (7″*S*,7‴*S*,8a*S*), (7″*S*,7‴*R*,8a*S*), (7″*R*,7‴*S*,8a*S*), (7″*S*,7‴*S*,8a*R*), (7″*R*,7‴*R*,8a*R*), (7″*R*,7‴*S*,8a*R*), and (7″*S*,7‴*R*,8a*R*). The ECD spectrum was predominantly dominated by axial chirality, as demonstrated by Polavarapu.^17^ This became evident since the *aS* stereoisomers presented similarity with the experimental spectra (Figure S10), allowing us to determine the axial chirality present in the compound. Among the four *aS* stereoisomers (Figure S11), similarity was observed between the ECD spectra in the region of the negative Cotton effects at 315 nm and positive Cotton effects at 355 nm, although exhibiting distinct patterns in the region of the negative Cotton effect at 240 nm. Thus, it could be inferred that the first two would be intrinsically correlated with the axial chirality of the molecule, leading to the phenomenon of atropisomerism, and the latter to the two stereogenic centers at 7″ and 7‴. In addition, it was demonstrated that, by varying the temperature from 25 to 60 °C (Figure 2), the Cotton effects observed at 315 and 355 nm showed a significant decrease in intensity at higher temperature. On the other hand, the Cotton effect at 240 nm remained unchanged (Figure 2). These results indicated plausibility of atropisomerism. The calculation of ^13^C and ^1^H NMR chemical shifts, followed by the application of the DP4+ methodology,^18,19^ allowed the determination of the relative stereochemistry, with a probability of 100% for the stereoisomer with the 7″S*,7‴S*,8a*S* configuration (Tables S1 and S3). This proposal is confirmed by the simulated ECD spectrum, which bears the closest resemblance to the experimental one (Figure 3). Therefore, it was possible to establish the absolute configuration as 7″*S*, 7‴*S*, 8a*S* (Figure 3) and compound **1** received the trivial name metternichiamide A.

Compound **2** was isolated as a white amorphous powder. Its molecular formula was established as C_38_H_42_N_2_O_13_ based on HRESIMS ([M + Na]^+^ at *m*/*z* 757.2528, calcd 757.2579, Δ = 2.8 ppm; HDI = 19; Figure S21). The ^1^H NMR spectrum (Figures S12 and S14) showed aromatic proton signals at δ_H_ 6.73 (s, 4H), δ_H_ 6.96 (d, 4H, *J* = 8.5 Hz) and δ_H_ 6.66 (d, 4H, *J* = 8.5 Hz), suggesting the presence of 1,3,4,5-tetrasubstituted and 1,4-disubstituted aromatic rings. The IR spectrum showed absorptions at 3306 cm^–1^ (hydroxyl), 1659 cm^–1^ (amide carbonyl), 1612 and 1516 cm^–1^ (C=C of aromatic ring). In the NMR spectrum of ^13^C-BB and DEPT135 (Figures S15 and S16), signals from oxymethine carbons at δ_C_ 73.1 (C-7″,7‴) and from methylene carbons at δ_C_ 48.1 (C-8″, C8‴) suggested the presence of octopamine moiety.^11^ In the HSQC spectrum (Figure S17), correlations were observed at δ_H_ 3.38, (dd, *J* = 6.3, 2.8 Hz)/δ_C_ 60.3 (H-8,8′/C-8,8′), δ_H_ 5.27 (d, *J* = 6.3 Hz)/δ_C_ 86.3 (H-7/C-7) and at δ_H_ 5.29, (d, *J* = 6.3 Hz)/δ_C_ 86.3 (H-7′/C-7′), which are compatible with a tetrahydrofuran-type lignan^7^ (Figure 1). The overlapping ^1^H and ^13^C NMR signals suggested that compound **2** would be a C2-symmetry dimer similar to lyciumamide K,^7^ except for the constituent monomer being the *N-trans*-sinapoyloctopamine.

In the HMBC spectrum (Figures 1 and S18), the mutual correlations of the protons H-7 (δ_H_ 5.27), H-7′ (δ_H_ 5.29), with signals at δ_C_ 60.3 (C-8,8′), 132.3 (C-1,1′), 104.8 (C-2,2′,6,6′) and 172.5 (C-9, C-9′), confirmed the presence of a tetrahydrofuran-type lignan. In the COSY spectrum (Figure S19), the correlations between H-7/H-8 and H-7′/H-8′; H-7″ with H_2_-8″, and of H-7‴ with H_2_-8‴, corroborated the proposal for compound **2**. Its relative configuration was established based on scalar coupling constants (Table 1) and on the NOESY correlations (Figure S20). NOEs between H-8 and the aromatic protons H-2,6 (δ_H_ 6.73) suggested that they are *syn* oriented, analogously to H-2′,6′ with H-8′ (Figure 1). This was consistent with a *trans* arrangement between the protons H-7 (δ_H_ 5.27) and H-7′ (δ_H_ 5.29), both with ^3^*J*_HH_ = 6.3 Hz. The proposed relative configuration was confirmed by the calculation of ^13^C and ^1^H NMR chemical shifts. Based on the spectroscopic data, four possible diastereoisomers for compound **2** were calculated: (7S*,7′S*,7″S*,7‴R*,8S*,8′S*), (7S*,7′S*,7″R*,7‴S*,8S*,8′S*), (7S*,7′S*,7″R*,7‴R*,8S*,8′S*) and (7S*,7′S*,7″S*,7‴S*,8S*,8′S*) (Tables S5 and S6). The DP4+ methodology^18,19^ was applied and yielded a probability of 100% for the candidate 7S*,7′S*,7″R*,7‴R*,8S*,8′S* (Table S4). By the comparison of the simulated and experimental ECD, the absolute configuration was determined to be 7*S*,7′*S*,7″*R*,7″*R*,8*S*,8′*S* (Figure 3). Thus, compound **2** was identified as a new lignanamide, receiving the trivial name of metternichiamide B.

Compound **3** was isolated as a white amorphous powder. Its molecular formula was established as C_38_H_42_N_2_O_12_ based on HRESIMS ([M + H]^+^ at *m*/*z* 719.2797 calcd 719.2811, Δ = 1.9 ppm; HDI = 19; Figure S31). Analysis of the NMR data revealed that compound **3** was similar to compound **2** (Tables 1 and 2), but it did not consist of a homodimer. The ^1^H NMR spectrum (Figures S22–S24) displayed the signals that correspond to the characteristic aromatic protons of two 1,3,4,5-tetrasubstituted and two 1,4-disubstituted aromatic rings (Table 1). In the NMR spectrum of ^13^C-BB and DEPT135 (Figures S25 and S26), signals of one methine carbon at δ_C_ 73.2 (C-7‴) and three methylene carbons at δ_C_ 35.7 (C-7″), 42.6 (C-8″) and 48.1 (C-8‴) suggested the presence of octopamine and tyramine units. In the HSQC spectrum (Figure S27), the set of correlations of the signals at δ_H_ 3.35, m/δ_C_ 60.6 (H-8/C-8), δ_H_ 3.40, m/δ_C_ 60.3 (H-8′/C-8′), δ_H_ 5.26, d, *J* = 6.6 Hz/δ_C_ 86.4 (H-7/C-7), and δ_H_ 5.28, d, *J* = 6.4 Hz/δ_C_ 86.4 (H-7′/C-7′) corroborated the presence of oxygenated carbons, which are characteristic of tetrahydrofuran-type lignans.

In the HMBC spectra (Figures 1 and S28), the mutual correlations of H-7 (δ_H_ 5.26), H-8a″ (δ_H_ 3.37, m), H-8b″ (δ_H_ 3.18, m) with a signal at δ_C_ 172.1 (C-9), and H-7′ (δ_H_ 5.28), H-8a‴ (δ_H_ 3.49), H-8b‴ (δ_H_ 3.18) with a signal at δ_C_ 172.5 (C-9′) confirmed the union of the two monomers (*N-trans*-sinapoyltyramine, and *N-trans*-sinapoyloctopamine) via the tetrahydrofuran unit. In the COSY spectrum (Figure S29), the correlations between H-7/H-8 and H-7′/H-8′, those of the signals H-7a″ and H-7b″ with H-8a″ and H-8b″, and between H-7‴, H-8a‴ H-8b‴, supported the proposed structure of a tetrahydrofuran-type lignan (Figure 1). The NOESY spectrum (Figures 1 and S30) revealed correlations of H-2,6 and H-2′,6′ with H-8 and H-8′, respectively, suggesting that they are *syn* oriented. Thus, four-stereoisomers were possible for compound **3**: (7*S*,7′*S*,7‴*S*,8*S*,8′*S*), (7*R*,7′*R*,7‴*R*,8*R*,8′*R*), (7*S*,7′*S*,7‴*R*,8*S*,8′*S*) and (7*R*,7′*R*,7‴*S*,8*R*,8′*R*). The ECD spectrum (Figure S32) indicated that, specifically, two stereoisomers, 7*S*,7′*S*,7‴*S*,8*S*,8′*S* and 7*R*,7′*R*,7‴*S*,8*R*,8′*R* show the best correspondence with the experimental data. Therefore, the ^1^H and ^13^C NMR chemical shifts were calculated using a GIAO-HDFT at theory level^20^ and were compared with the experimental data (Tables S8 and S9). After the simulations, the DP4+ methodology^18,19^ was applied, resulting in 100% probability for the stereoisomer with the configuration 7*S*,7′*S*,7‴*S*,8*S*,8′*S* (Table S7 and Figure 3). Based on these spectral data, compound **3** was determined to be a novel tetrahydrofuran lignanamide with the trivial name of metternichiamide C.

Compound **4** was isolated as a white amorphous powder. Its molecular formula was established as C_38_H_40_N_2_O_12_ based on HRESIMS ([M + H]^+^ at *m*/*z* 717.2634 calcd, 717.2654, Δ = 2.8 ppm; HDI = 20; Figure S42). In the ^1^H NMR spectrum (Figures S33–S35), the set of doublets between δ_H_ 6.67 and 7.14, in addition to the signals at δ_H_ 4.68 (1H, dd, *J* = 7.6, 5.1 Hz), 4.53 (1H, t, *J* = 6.2 Hz), 3.39–3.42 (m), 3.34–3.37 (m), 3.25 (dd, 6.0, 1.6 Hz), were compatible with a lignanamide formed by two octopamine moieties. Moreover, signals were observed at δ_H_ 4.87 (1H, brs) and δ_H_ 3.72 (1H, d, *J* = 1.8), referring to the protons of the aryldihydronaphthalene unit,^3,21^ in addition to the signals at δ_H_ 3.92 (3H, s), 3.68 (6H, s), 3.58 (3H, s), corresponding to four methoxyls. The ^1^H and ^13^C NMR spectroscopic data (Tables 1 and 2 and Figures S33–S37) of compound **4** were similar to those of flavifloramide B,^22^ differing only by the insertion of hydroxyls in carbons C-7″,7‴. The HSQC spectrum (Figure S38) showed the correlations of δ_H_ 4.87, brs/δ_C_ 41.4 (H-7′/C-7′) and δ_H_ 3.72, d/δ_C_ 50.2 (H-8′/C-8′) and in the COSY (Figures 1 and S40) between the protons of the H-7″ and H-7‴ with H-8″ H-8‴, respectively. In the HMBC spectrum (Figures 1 and S39), the mutual correlations of H-7″ with C-2″,6″ and C-8″, and H-7‴ with C-2‴,6‴ and C-8‴, confirmed the hydroxyls in the carbons C-7″ and C-7‴. In the NOESY spectrum (Figures 1 and S41), the correlation of H-2′-6′ with H-7 and H-8′ inferred relative *trans* configuration between H-7′ and H-8′. Therefore, there are four possible stereoisomers for compound **4**, (7′S*,7″R*,7‴R*,8′R*), (7′S*,7″S*,7‴S*,8′R*), (7′S*,7″R*,7‴S*,8′R*) and (7′S*,7″S*,7‴R*,8′R*). Among these, the calculated ^13^C and ^1^H NMR chemical shifts, along with the DP4+ method,^18,19^ defined the candidate 7′S*,7″R*,7‴S*,8′R* with a probability of 100% (Tables S10–S12). The comparison between simulated and experimental ECD spectra defined the absolute configuration of compound **4** as 7′*R*,7″*S*,7‴*R*,8′*S* (Figure 3). This new lignanamide was given the trivial name metternichiamide D.

Compound **5** was isolated as a white amorphous powder. Its molecular formula was established as C_38_H_40_N_2_O_11_ based on HRESIMS ([M + H]^+^ at *m*/*z* 701.2674 calcd, 701.2705, Δ = 4.5 ppm, HDI = 20; Figure S53). Analysis of ^1^H and ^13^C-BB NMR data and DEPT135 (Figures S43–S47) indicated that compound **5** was similar to compound **4** (Tables 1 and 2), differing only by the presence of a single oxymethine proton signal at δ_H_ 4.52 (t, *J* = 6.2 Hz) and three methylene carbon signals at δ_C_ 35.6 (C-7″), δ_C_ 42.8 (C-8″) and δ_C_ 47.9 (C-8‴). The correlations in the HSQC spectrum (Figure S48) at δ_H_ 2.68, t, *J* = 7.2 Hz/δ_C_ 35.6 (H_2_-7″/C-7″) at δ_H_ 3.33–3.43 m/δ_C_ 42.8 (H_2_-8″/C-8″), at δ_H_ 4.52/δ_C_ 72.8 (H-7‴/C-7‴) and at δ_H_ 3.33–3.38, m and δ_H_ 3.26, dd, *J* = 13.4, 6.0 Hz,/δ_C_ 47.9 (H-8a‴, H-8b‴/C-8‴), associated with those of the HMBC spectrum (Figures 1 and S49) of the protons H_2_-8″ with the carbon at δ_C_ 170.0 (C-9), and the H-8a‴ H-8b‴ with δ_C_ 174.2 (C-9′), confirmed the presence of a tyramine moiety and an octopamine moiety bonded at C-9 and C-9′, respectively. The relative configuration of C-7′, C-7‴, and C-8′ was determined unequivocally by calculations of the ^13^C and ^1^H NMR chemical shifts (Tables S14 and S15). Subsequently, the DP4+ methodology^18,19^ was applied, resulting in a probability of 100% for the candidate with configuration 7′S*,7‴S*,8′R* (Table S13), as indicated by the NOESY spectrum (Figures S51 and S52). Analysis of the ECD data established the absolute configuration as 7′*S*,7‴*S*,8′*R* (Figure 3), and compound **5** received the trivial name of metternichiamide E.

The MTT assay, a colorimetric test for assessing cell metabolic activity, was used to establish noncytotoxic concentrations of compounds **1**–**5** in RAW 264.7 macrophages. None of the compounds significantly reduced cell viability up to a concentration of 100 μM (50% cytotoxic concentration—CC_50_ > 100 μM) (Table 3). Then, the effect of the compounds **1**–**5** on NO, IL-1β and IL-10 production was evaluated in LPS and IFN-γ stimulated RAW 264.7 cell line.

**Table 3:** Cell Viability (%) of RAW 264.7 Macrophages After Treatment with Compounds **1**–**5**^a^

	cell viability (%)		
compound	25 μM	50 μM	100 μM
control	100 ± 1.3		
**1**	95.3 ± 1.7	96.9 ± 2.8	101.8 ± 6
**2**	102.3 ± 1.3	100.5 ± 1.3	103.4 ± 1.7
**3**	102.1 ± 5.8	109.1 ± 3	114.6 ± 1.4
**4**	103.6 ± 2.4	104.1 ± 3.5	92.6 ± 3.1
**5**	92.3 ± 1.5	94.8 ± 0.7	98.1 ± 1.8

a Results are mean ± standard error of the mean (SEM), *n* = 5.

**Figure 4:**
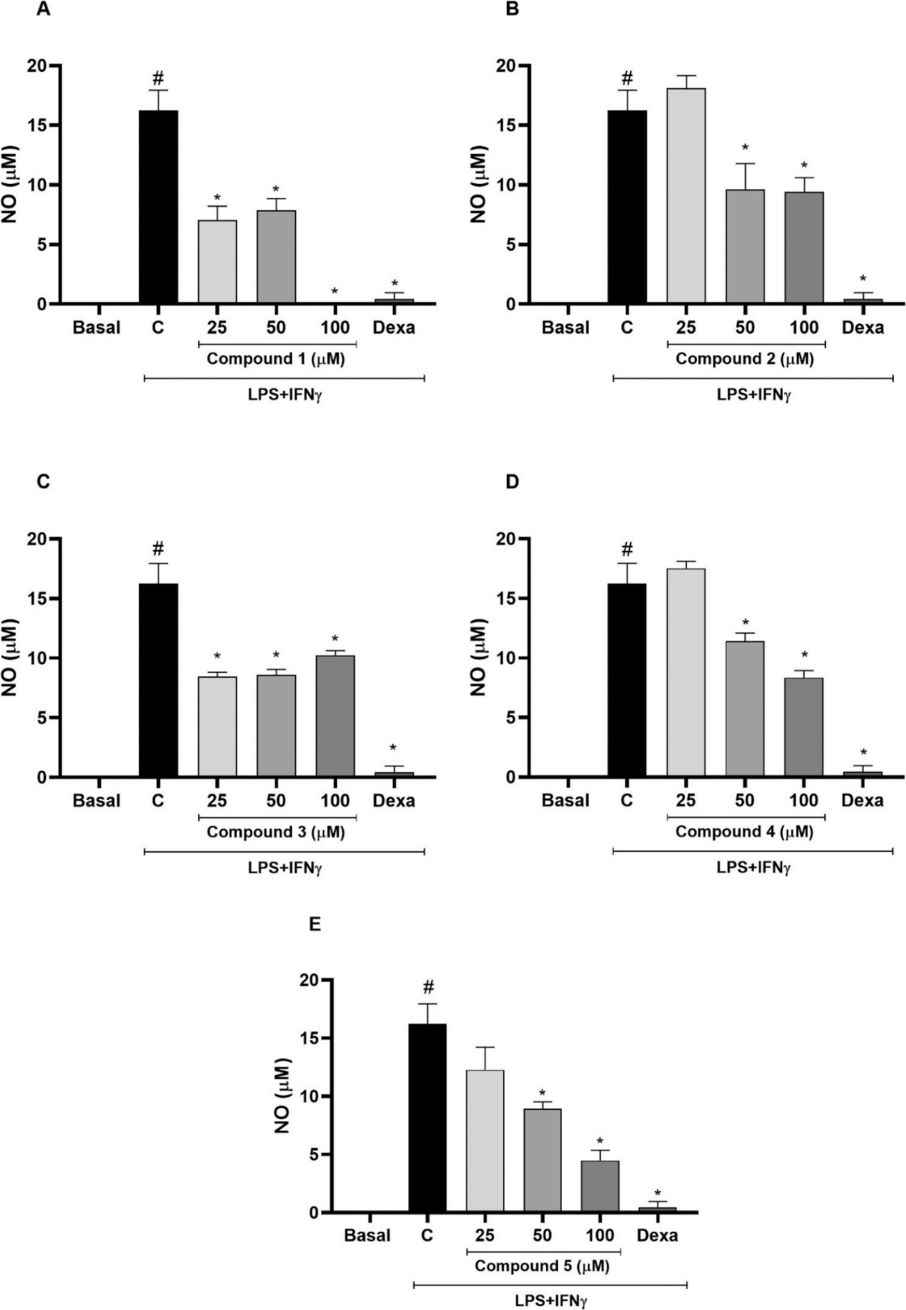
Inhibitory effect of compounds **1**–**5** on NO production in RAW 264.7 macrophages stimulated with LPS and IFN-γ. Results are mean ± standard error of the mean (SEM), *n* = 5. ^#^Different from the basal group (*p* < 0.05). *Different from the control group (C) (*p* < 0.05). Dexa: standard drug (positive control, 20 μM).

Compounds **1** and **3** reduced nitrite levels compared to the stimulated group (*p* < 0.05) at all tested concentrations (25, 50, and 100 μM) (Figure 4). The significant effect of compounds **2**, **4**, and **5** on nitrite levels reduction was observed at 50 and 100 μM (*p* < 0.05). As expected, the standard drug dexamethasone (20 μM) significantly reduced nitrite levels in stimulated RAW 264.7 cells (*p* < 0.05). Nitric oxide plays a crucial role in inflammation regulation,^23,24^ therefore, our results suggest anti-inflammatory effect for compounds **1**–**5**.

**Figure 5:**
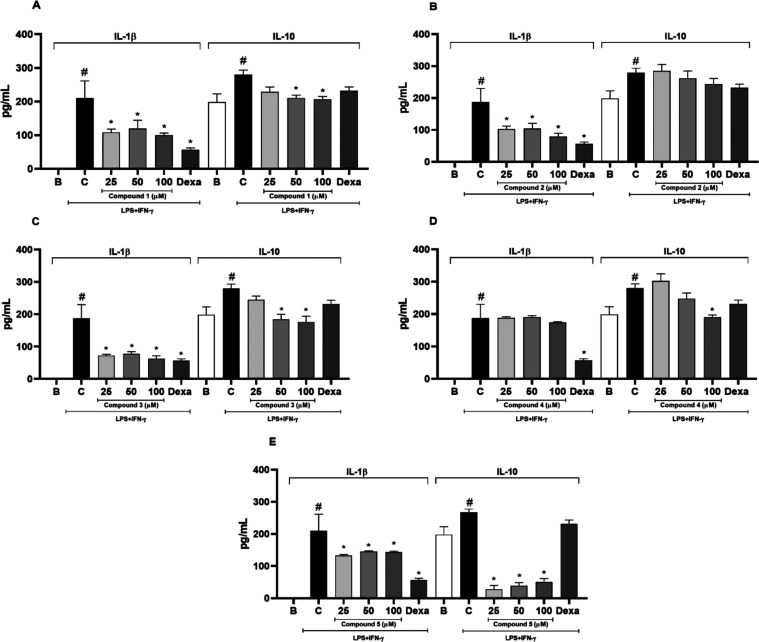
Inhibitory effect of compounds **1**–**5** on IL-1β and IL-10 levels in RAW 264.7 macrophages stimulated with LPS and IFN-γ. Results are mean ± standard error of the mean (SEM), *n* = 5. ^#^Different from the basal group (*p* < 0.05). ^*^Different from the control group (*p* < 0.05). Dexa: Standard drug (positive control, 20 μM). IL: Interleukin.

Regarding cytokines analysis, only **2** did not show a significant change on IL-10 levels compared to the vehicle-treated control group (Figure 5). The maximum inhibitory effects on IL-10 production for compounds **1, 3**, and **4** at 100 μM were 26.1, 37.2, and 31.9%, respectively. For **5**, the maximum inhibitory effect was 89.6% at 25 μM, and dexamethasone (20 μM) reduced IL-10 levels by 17.2%. IL-10 is classically defined as an anti-inflammatory cytokine; however, literature data from experimental and clinical studies have shown that in some pathological conditions IL-10 levels may be elevated, potentially producing detrimental effects.^25^ In fact, the reduction of IL-10 levels has been associated with the anti-inflammatory effect of pharmacologically active drugs tested *in vivo*,^26^ or *in vitro* (RAW 264.7 macrophages).^27,28^

Moreover, only **4** did not alter IL-1β levels. The other compounds reduced the IL-1β measurement at all tested concentrations (25, 50, and 100 μM) compared to the control group (*p* < 0.05) (Figure 2). The maximum inhibitory effects on IL-1β levels were 52.6, 57.7, 66.4, 36.9, and 70% for **1**, **2**, and **3** at 100 μM, **5** (25 μM), and dexamethasone (20 μM), respectively (Figure 2). Considering that reducing IL-1β levels is crucial to attenuating inflammation,^29^ our results for IL-1β production in RAW 264.7 macrophages model reinforce the potential anti-inflammatory of these compounds.

## Experimental Section

### General Experimental Procedures

The experimental ECD spectra were recorded with a Jasco J-1100 spectrometer (Jasco, Tokyo, Japan) in the 200–400 nm region using the following parameters: bandwidth 1 nm; response 1s; scanning speed 100 nm min^–1^; 3 accumulations; room temperature (25 °C); sample in methanol solution; 0.1 cm cell path length; concentration 0.1 mg mL^–1^. Spectra were smoothed in Origin 8 software.^30^ A polarimeter (Jasco P-2000 Easton, MD, USA) was used to measure optical rotation at 20 °C. IR spectra were recorded on a Fourier transform infrared spectrophotometer (Shimadzu Proeminence IR Prestige-21) using KBr granules, as well as a FTIR spectrophotometer (Shimadzu IRSpirit-T) using the QATR-S accessory. The 1D and 2D NMR experiments were conducted using two NMR spectrometers (Bruker Ascend −400 and 100 MHz for ^1^H and ^13^C and Bruker AvanceNeo 500–500 and 125 MHz for ^1^H and ^13^C). The ^1^H and ^13^C NMR chemical shifts were referenced to the solvent peaks for residual internal MeOD at δ_H_ 3.31 and δ_C_ 49.00. A mass spectrometer (MicroTOF II, Bruker Daltonics, Billerica, MA, USA) with an electrospray ion source (ESI) was used to perform ESI-TOF-MS analysis. Isolation, purification and analysis of the chemical constituents were conducted using various chromatographic methods, including medium pressure liquid chromatography (MPLC), column chromatography (CC), thin layer chromatography (TLC) and high-performance liquid chromatography (HPLC). A preparative RP C18 column (ACE, 250 mm × 21.2 mm × 5 μm) and a preparative RP C18 column (YMC, 250 mm × 20.0 mm × 5 μm) were used for preparative scale isolation. Silica gel (Siliaflash, particle size 0.060–0.200 mm) was the stationary phase used in the MPLC, while the Sephadex LH-20 support was used in CC. Commercial silica gel plates (Whatman) were used in the TLC in layers with a thickness of 0.25 mm on an aluminum support (20 cm × 20 cm). The substances were analyzed via ultraviolet radiation at wavelengths of 254 and 366 nm (Botton brand apparatus).

### Plant Material

The plant material was collected at Fazenda Esperança, in the municipality of Boa Vista do Tupim, Bahia, Brazil (13°13′15″S, 41°11′08″W) in January 2019. The species was identified by Prof. Dr. Domingos Benício Oliveira Silva Cardoso of the Department of Botany of the Institute of Biology at the Federal University of Bahia (UFBA). The access registry in the National System for the Management of Genetic Heritage and Associated Traditional Knowledge (SISGEN) was obtained under the number AA54545. An exsiccate of the collected material was produced and deposited at the Alexandre Leal Costa Herbarium (ALCB) at UFBA. The plant material underwent a drying process in an air circulation oven at a temperature of 40 °C for 72 h.

### Extraction and Isolation

The dried and ground roots of *M. macrocalyx* (3.4 kg) were extracted with EtOH (x3 for 72 h) at room temperature. The resulting extract was concentrated under reduced pressure in a rotary evaporator at 40 °C to obtain 70 g of crude ethanolic extract. An aliquot of 65 g was resuspended in a solution of EtOH-H_2_O (7:3) and partitioned successively with *n*-hexane, CHCl_3_, EtOAc, culminating in 7.1, 34.1, and 0.6 g of the respective extracts and 16.4 g of the hydroethanolic extract. The chloroform extract (3.0 g) was subjected to silica gel MPLC and eluted with hexane, EtOAc and MeOH, pure or in a binary mixture, in an increasing gradient of polarity. From this procedure, 12 fractions were obtained that were grouped into 10 groups (A1–12) according to their chromatographic profile in TLC. The fraction group A4–6 (130 mg) was subjected to preparative chromatographic separation using HPLC, in a C18 column (ACE, 250 mm × 21.2 mm × 5 μm) and the following elution gradient: solvent A = HCOOH:H_2_O 0.1% v/v; solvent B = MeOH; elution profile = 0.0–45.0 min (5–35% B); 45.0–70.0 min (5–35% B); (35–45% B); 70.0–95.0 min (45–70% B); 95.0–100.0 min (70–100% B); 100.0–105.0 min (100% B); 105.0–120.0 min (100% B); 120.0–125.0 min (100–5% B); 125.0–145.0 min (5% B); injection volume of 100 μL and flow rate of 8.0 mL/min; 15 fractions were obtained. Analysis of the ^1^H and ^13^C NMR spectra of the isolated peaks led to the identification of ten compounds: **1** (1.3 mg, *t*_R_ = 59.7 min), **6** (6.6 mg, *t*_*R*_ = 66.7 min), **7** (5.6 mg, *t*_R_ = 65.2 min), **8** (4.8 mg, *t*_R_ = 82.0 min), **9** (3.0 mg, *t*_*R*_ = 71.1 min), **10** (1.0 mg, *t*_*R*_ = 79.7 min), as well as a mixture of two positional isomers **11** and **12** (3.2 mg, *t*_*R*_ = 83.3 min) and two geometric isomers **13** and **14** (3.0 mg, *t*_R_ = 101.2 min). The amide-rich fraction A7 (1.1 g) was separated in Sephadex LH-20 and eluted with MeOH to obtain 15 fractions (B1–15), which were monitored via LC-DAD. Fractions B8–10 (377.1 mg) with similar chromatographic profiles were pooled and subjected to preparative chromatographic separation using HPLC, in a C18 column (ACE, 250 mm × 21.2 mm × 5 μm) and the following elution gradient: solvent A = H_2_O; solvent B = MeOH; elution profile = 0.0–45.0 min (5–35% B); 45.0–70.0 min (5–35% B); (35–45% B); 70.0–120.0 min (45–60% B); 120–125.0 min (60–100% B); 125.0–140.0 min (100% B); 140.0–145.0 min (5% b); 145.0–165.0 min (5% B); injection volume of 200 μL and flow rate of 8.0 mL/min; 21 fractions were obtained. The analysis of the ^1^H and ^13^C NMR spectra of the isolated peaks led to the identification of compounds **2** (6.0 mg, *t*_R_ = 59.9 min) and **15** (5.0 mg, *t*_R_ = 109.5 min), in addition to the reisolated compounds **6** (10 mg, *t*_R_ = 68.0 min) and **7** (8.6 mg, *t*_R_ = 66.6 min). Given the manifest potential for biosynthesis of new lignanamides, and the need for reisolation, for complementary analysis and evaluation of biological activity, an aliquot of the chloroform extract (4.0 g) was subjected to MPLC on silica gel and eluted with hexane, EtOAc and MeOH, pure or in a binary mixture in an increasing gradient of polarity. From this procedure, 10 fractions (C1–10) were obtained, which were combined into 7 fractions according to their chromatographic profile in HPLC-DAD. The group fraction C4–7 (1.3 g) was subjected to reverse phase MPLC (C18) and eluted with MEOH and H_2_O in a binary mixture by means of an increasing gradient of polarity up to 100% MeOH. From this procedure, 8 fractions (D1–8) were obtained. Fraction D2 (148 mg) was subjected to preparative chromatographic separation using HPLC in a C18 column (YMC, 250 mm × 20.0 mm × 5 μm) and the following elution gradient: solvent A = H_2_O; solvent B = MeOH; elution profile = 0.0–40.0 min (25–40% B); 40.0–70.0 min (40–46% B); 70.0–70.0 min (40–46% B); 75.0 min (46–100% B); 75.0–90.0 min (100% B); 90.0–95.0 min (100–25% B); 95.0–115.0 min (25% B); injection volume of 100 μL and flow rate of 8.0 mL/min; 16 fractions were obtained (D2.1–D2.16). The analysis of the ^1^H NMR spectrum of the isolated peaks led to the identification of compounds **3** (2.6 mg, *t*_R_ = 73.5 min), **4** (7.2 mg, *t*_R_ = 43.4 min) and **5** (4.4 mg, *t*_R_ = 65.9 min), in addition to the reisolated compounds, **2** (10.4 mg, *t*_R_ = 51.9 min), **6** (16.6 mg, *t*_R_= 60.6 min) and **7** (8.1 mg, *t*_R_ = 63.0 min). Fraction D2.9 (3.1 mg) was purified using semipreparative HPLC in a C18 column (Venusil, 250 mm × 10.0 mm × 10 μm) and the following elution gradient: solvent A = H_2_O; solvent B = MeOH/ACN 50% v/v; elution profile = 0.0–25.0 min (5–25% B); 25.0–75.0 min (25–40% B); 75.0–80.0 min (40–100% B); 80.0–95.0 min (100% B); 95.0–100.0 min (100–5% B); 100.0–120.0 min (5% B); injection volume of 100 μL and a flow rate of 3.0 mL/min to obtain compound **1** (1.0 mg). Fraction D1 (151 mg) was fractionated via preparative RP-HPLC using MeOH and H_2_O as the eluent (8 mL/min), by means of a gradient system with the following parameters: 0.0–83.0 min (25–46% B); 83.0–87.0 min (46–100% B); 87.0–102.0 min (100% B); 102.0–107.0 min (100% B); 100–25% (B); 107.0–127.0 min (25% B); 12 fractions (D1.1–D1.12) were obtained. This process led to the reisolation of compound **4** (7.7 mg, *t*_R_ = 38.2 min) and **5** (1.0 mg, *t*_R_ = 59.1 min). In addition, fraction D1.5 (2.6 mg) was purified via semipreparative RP-HPLC using MeOH/ACN 50% v/v (solvent B) and H_2_O (solvent A) using an isocratic flow of 3 mL/min with 30% B and a running time of 70 min to obtain compound **1** (1.2 mg).

#### Metternichiamide A (**1**)

yellowish amorphous powder; [α]_D_^22^ +4 (*c* 0.1, MeOH); IR (ATR) ν_max_ 3343, 1651, 1611, and 1514 cm^–1^; melting point: 131.0 °C; ^1^H and ^13^C NMR data, see Tables 1 and 2; positive-ion HRESIMS *m*/*z* 717.2626 [M + H]^+^ (calcd for C_38_H_41_N_2_O_12_, 717.2654, Δ = 3.9 ppm).

#### Metternichiamide B (**2**)

white amorphous powder; [α]_D_^20^ +15 (*c* 0.09, MeOH); IR (KBr) ν_max_ 3306, 1659, 1612, and 1516 cm^–1^; melting point: 148.0 °C; ^1^H and ^13^C NMR data, see Tables 1 and 2; positive-ion HRESIMS *m*/*z* 757.2528 [M + Na]^+^ (calcd for C_38_H_42_N_2_NaO_13_, 757.2579, Δ = 2.8 ppm).

#### Metternichiamide C (**3**)

white amorphous powder; [α]_D_^20^ +6 (*c* 0.22, MeOH); IR (KBr) ν_max_ 3310, 1659, 1612, and 1516 cm^–1^; melting point: 124.0 °C; ^1^H and ^13^C NMR data, see Tables 1 and 2; positive-ion HRESIMS *m*/*z* 719.2797 [M + H]^+^ (calcd for C_38_H_43_N_2_O_12_, 719.2811, Δ = 1.9 ppm).

#### Metternichiamide D (**4**)

white amorphous powder; [α]_D_^20^ −21 (*c* 0.37, MeOH); IR (KBr) ν_max_ 3275, 2920, 2847, 1639, 1609, 1512, 1454, 1223, 1111, and 833 cm^–1^; melting point: 147.0 °C; ^1^H and ^13^C NMR data, see Tables 1 and 2; positive-ion HRESIMS *m*/*z* 717.2634 [M + H]^+^ (calcd for C_38_H_41_N_2_O_12_, 757.2654, Δ = 2.8 ppm).

#### Metternichiamide E (**5**)

white amorphous powder; [α]_D_^20^ −10 (*c* 0.26, MeOH); IR (KBr) ν_max_ 3418, 1597, 1512, 1458, 1223, and 1111 cm^–1^; melting point: 136.0 °C; ^1^H and ^13^C NMR data, see Tables 1 and 2; positive-ion HRESIMS *m*/*z* 701.2674 [M + H]^+^ (calcd for C_38_H_41_N_2_O_11_, 701.2705, Δ = 4.5 ppm).

### ECD and NMR Calculations

Randomized conformational searches were performed for all the possible stereoisomers using the Monte Carlo algorithm with an MMFF force field in SPARTAN ’14 software. All the conformers within a relative free energy window of 10 kcal mol^–1^ were selected for geometric optimization calculations in the gas phase, employing the B3LYP/6-31G(d) level of theory. Vibrational frequency calculations were performed at the same level of theory to confirm that stationary points correspond to minima on the potential energy surface. Subsequently, conformers within a relative energy window of 3 kcal mol^–1^ were selected for simulations of ECD spectra and/or calculations of ^13^C and ^1^H (σ) nuclear magnetic shielding constants. For the ECD simulations, the TD-DFT level of theory was applied: CAM-B3LYP/TZVP, employing a polarizable continuous model with integral equation formalism (IEF-PCM) to implicitly simulate MeOH as the solvent. The final ECD spectra were generated based on Boltzmann statistics of the selected conformers and plotted using Origin 8 software.^30^ Nuclear magnetic shielding calculations were conducted using the GIAO-mPW1PW91/6-31G(d) level of theory, along with the IEF-PCM method to simulate solvation by MeOH. The mean shielding constants of the population were obtained by assuming the Boltzmann statistics at a temperature of 298 K. Finally, the ^13^C and ^1^H NMR (δ) chemical shifts were obtained as δ calc = σTMS – σ, where σTMS represents the shielding constant of the reference compound (tetramethylsilane, TMS), which was calculated using the same levels of theory. The DP4+ method, which relies on Bayesian analysis, was used to establish a statistical correlation between the calculated and experimental ^13^C and ^1^H chemical shifts. Each structure was ranked based on probabilities, and probabilities greater than 90% indicated a high level of confidence in which the candidate exhibited the best agreement with the experimental data.^18,19^ All quantum mechanical calculations were performed using the Gaussian 16 software package.^31^

### Cytotoxicity Assay

The 3-(4,5-dimethylthiazol-2-yl)-2,5-diphenyltetrazolium bromide (MTT) assay was used to assess the cytotoxicity of the compounds **1**–**5**.^32^ The murine macrophage-like RAW 264.7 cell line was obtained from the Rio de Janeiro Cell Bank (BCRJ), Brazil, and cultured in Dulbecco’s Modified Eagle Medium (DMEM; Sigma-Aldrich, St. Louis, MO, USA), supplemented with 10% fetal bovine serum (FBS; GIBCO, Grand Island, NY, USA) and 1% penicillin-streptomycin (Sigma-Aldrich) at 37 °C with 5% CO_2_. The cells were plated in 96-well plates at a cell density of 1 × 10^5^ cells/mL and incubated overnight. The compounds **1**–**5** were added at three concentrations (25, 50, or 100 μM) in five replicates, and the plates were incubated for 24 h. After the treatments, the supernatant (110 μL) was removed and 10 μL of MTT solution was added (5 mg/mL) (Sigma-Aldrich, St. Louis, MO, USA). The plates were incubated for an additional four hours, and sodium dodecyl sulfate (SDS) (100 μL/well) was added to dissolved the formazan. Optical densities were measured on a spectrophotometer (BioTek Instruments microplate reader, Sinergy HT, Winooski, VT, USA) at a wavelength of 570 nm.

### Nitric Oxide and Cytokine Production Assays

For NO, IL-1β, and IL-10 determinations, RAW 264.7 cells were seeded in 96-well culture plates at a density of 1 × 10^6^ cells/mL in DMEM medium supplemented with 10% FBS and 1% penicillin-streptomycin in a 5% CO_2_ incubator at 37 °C, as previously described. After a four-hour period, the cells were stimulated with LPS (500 ng/mL, Sigma-Aldrich) and IFN-γ (5 ng/mL, Thermofisher) in the presence of the compounds **1**–**5** (25, 50, or 100 μM) or dexamethasone (20 μM) in five replicates. Additionally, a stimulated control group (LPS/IFN-γ), and treated with vehicle was included. After 24 h, cell-free supernatants were collected to quantify the NO using the Griess method,^33^ or kept at −80 °C to determine cytokine concentrations. The IL-1β and IL-10 concentrations in macrophage culture supernatants were determined by enzyme-linked immunosorbent assay (ELISA) using the Invitrogen kit (ThermoFisher, VIE, Austria).

### Statistical Analysis

The data are presented as mean ± Standard Error of the Mean (SEM). Comparisons between groups were performed using one-way analysis of variance (ANOVA) with Tukey’s post-test (*p* < 0.05). Statistical analysis was performed using GraphPad Prism 8.0.2 (Graphpad Software Inc., San Diego, CA, USA).

## Supporting Information Available

MS and NMR data for compounds **1–15** (PDF)
