# Supplementary material for: Lignanamides from the Roots of Metternichia macrocalyx and Their Anti-inflammatory Activity
Source: ACS Omega. 2024 Nov 13;9(47):47065–76. doi: 10.1021/acsomega.4c07336 (PMC11603264; doi:10.1021/acsomega.4c07336)
Supplement: Supplementary file 1 — ao4c07336_si_001.pdf [file ao4c07336_si_001.pdf]

SUPPORTING INFORMATION

Lignanamides from the roots of *Metternichia macrocalyx* and their anti-inflammatory activity

Thiago A. M. Brito, Ana C. F. de Albuquerque, Fernando M. dos Santos Junior, Paulo B. A. Loureiro, Francisco A. A. F. Gadelha, Marianna V. Sobral, Domingos B. O. S. Cardoso, Eudes da Silva Velozo, Josean F. Tavares, Lucas S. Abreu, Marcelo S. da Silva.

Table of Contents

1 Spectroscopic data for compound **1** 1
2 Spectroscopic data for compound **2** 7
3 Spectroscopic data for Compound **3**....................12
4 Spectroscopic data for Compound **4**....................18
5 Spectroscopic data for compound **5** 23
6 Spectroscopic data for compound **6** 28
7 Spectroscopic data for compound **7** 31
8 Spectroscopic data for compound **8** 33
9 Spectroscopic data for compound **9** 36
10 Spectroscopic data for compound **10** 39
11 Spectroscopic data for compounds **11**,**12**....................42
12 Spectroscopic data for compounds **13**,**14**....................45
13 Spectroscopic data for compound **15** 50
14 Quantum mechanical calculations for compound **1**. 53
15 NMR calculation data for compound **1**. 54
16 NMR calculation data for compound **2**. 57
17 NMR calculation data for compound **3**. 60
18 NMR calculation data for compound **4**. 62
19 NMR calculation data for compound **5**. 65
20 Monte Carlo/MMFF force field calculations and Boltzmann population. 67

1 Spectroscopic data for compound 1

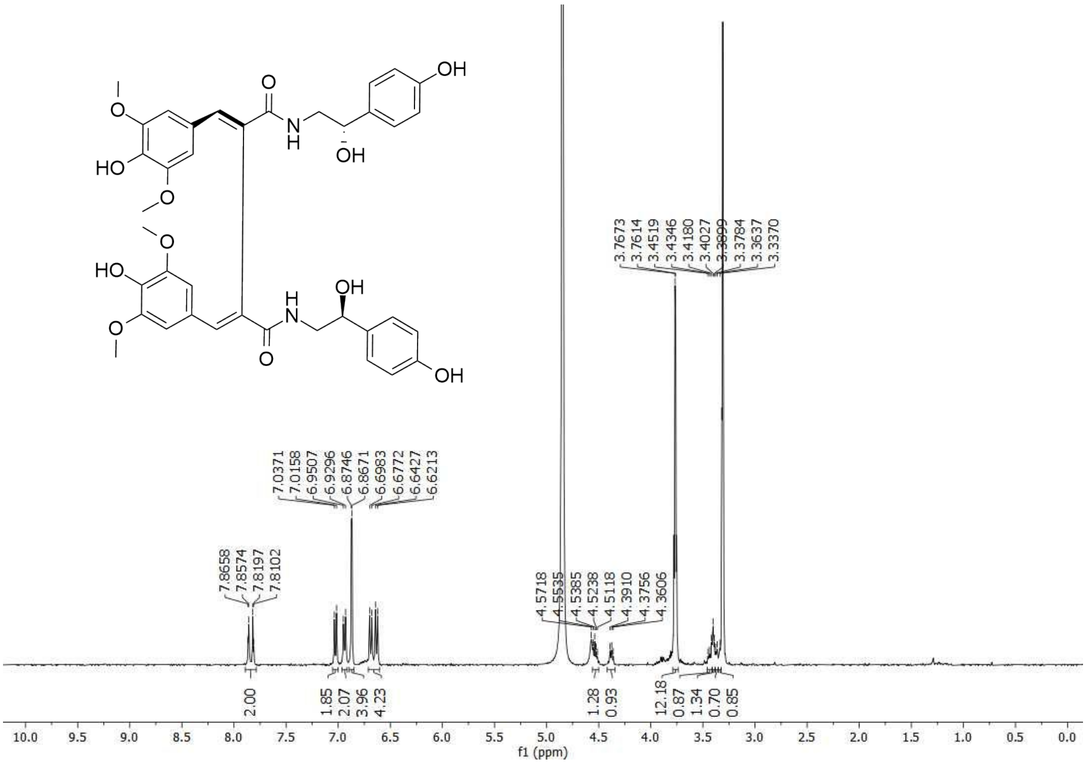

Figure S1. ^{1}H NMR spectrum of compound **1** (400 MHz, MeOD).

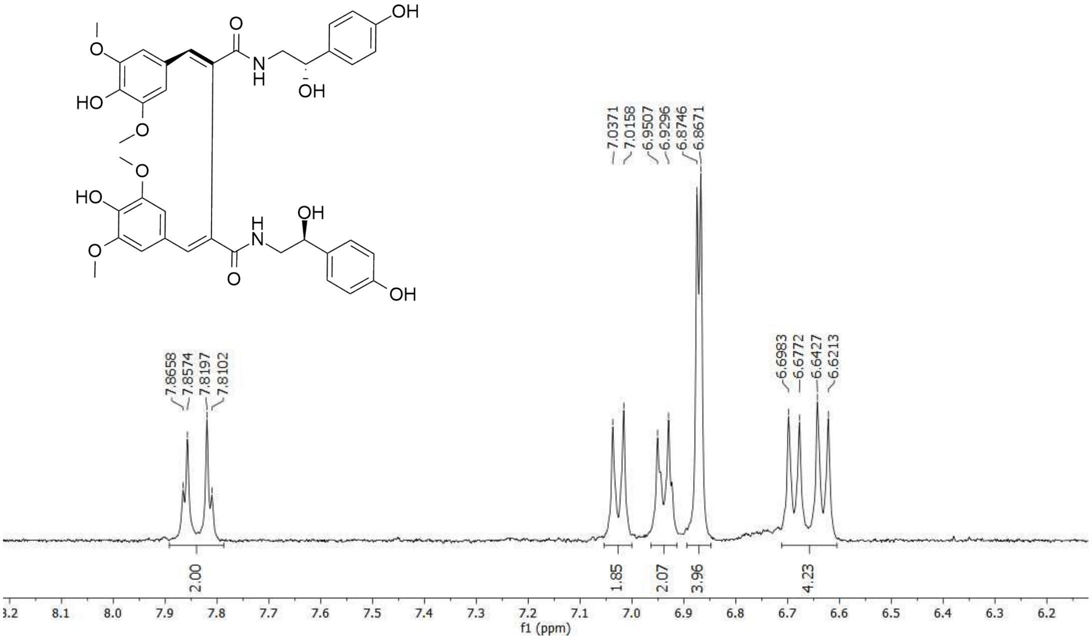

Figure S2. Amplification of the ^{1}H NMR spectrum of compound **1** (400 MHz, MeOD).

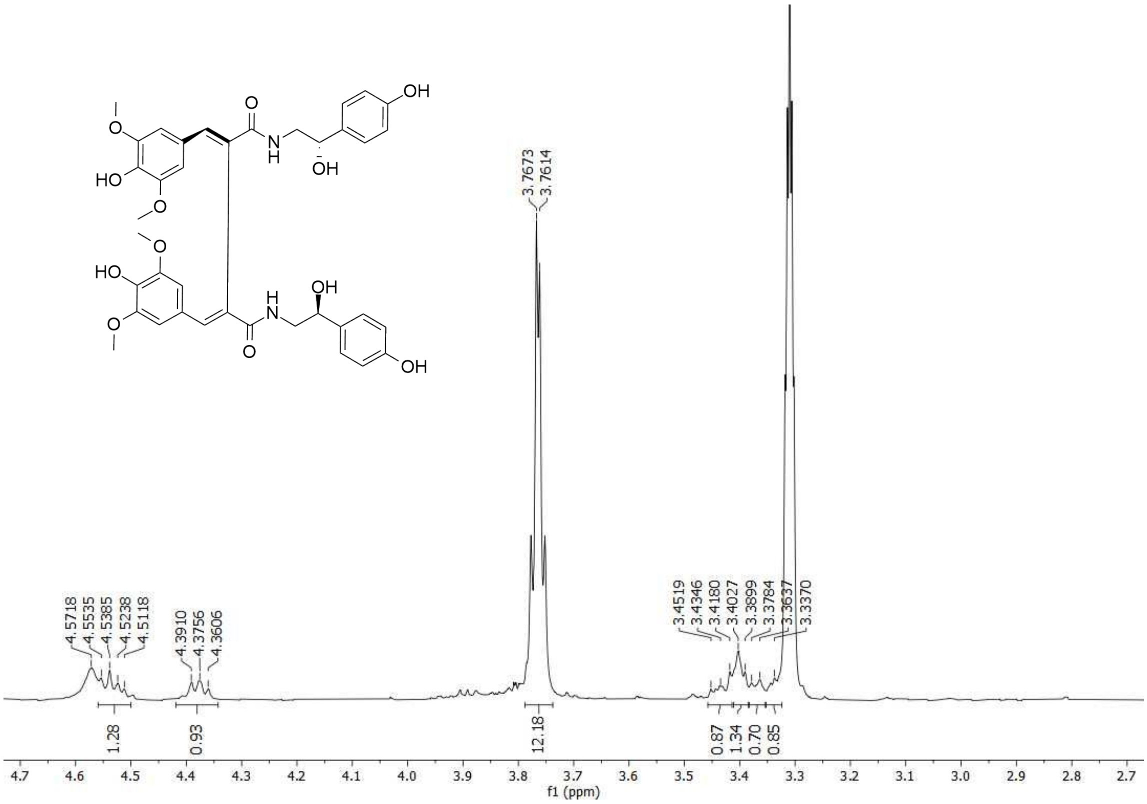

Figure S3. Amplification of the ^{1}H NMR spectrum of compound **1 (**400 MHz, MeOD).

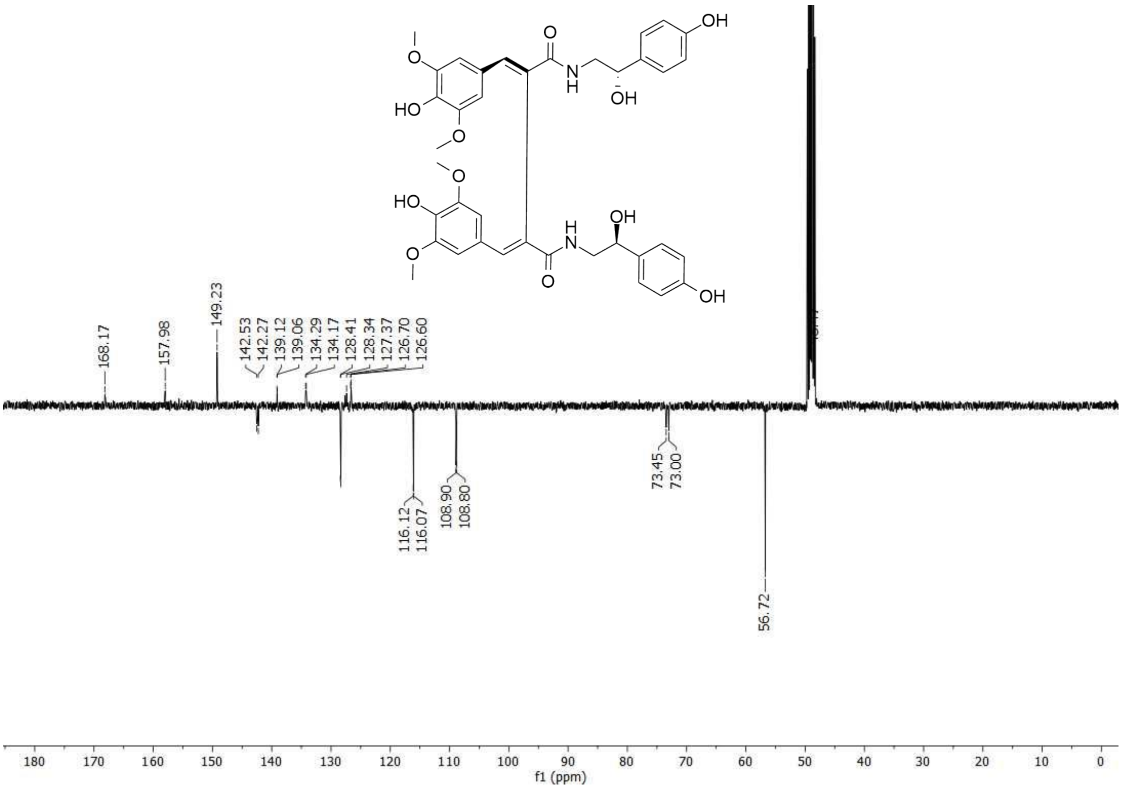

Figure S4. ^{13}C-APT NMR spectrum of compound **1** (100 MHz, MeOD).

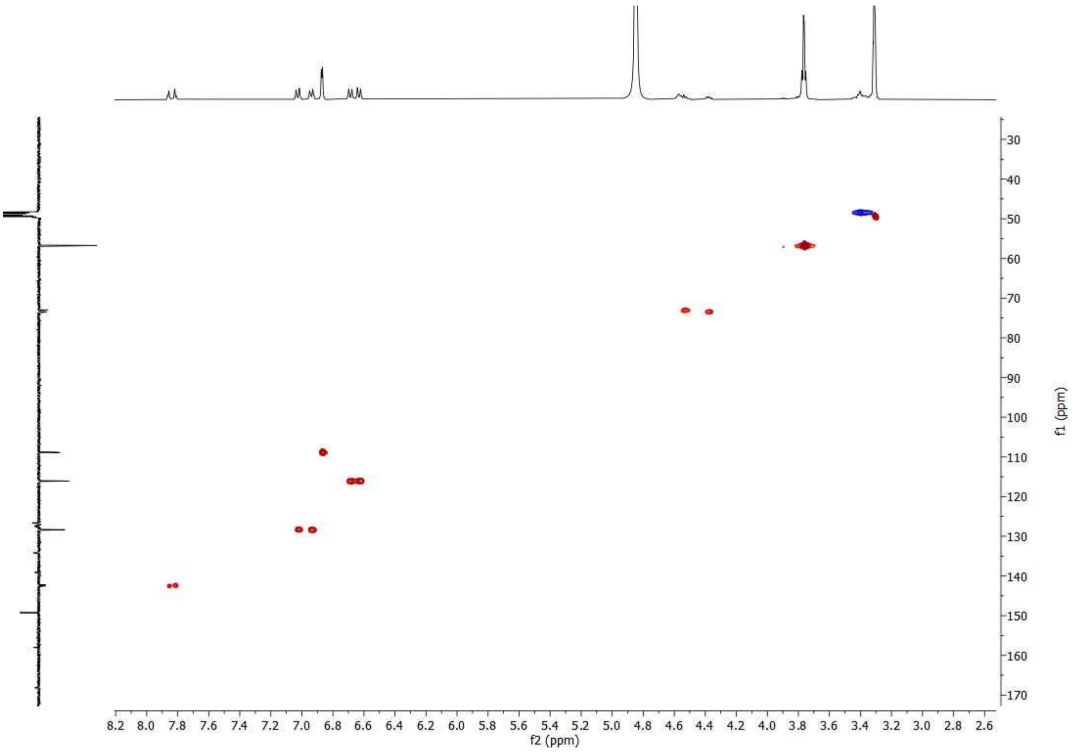

Figure S5. HSQC spectrum of compound **1 (**400/100 MHz, MeOD).

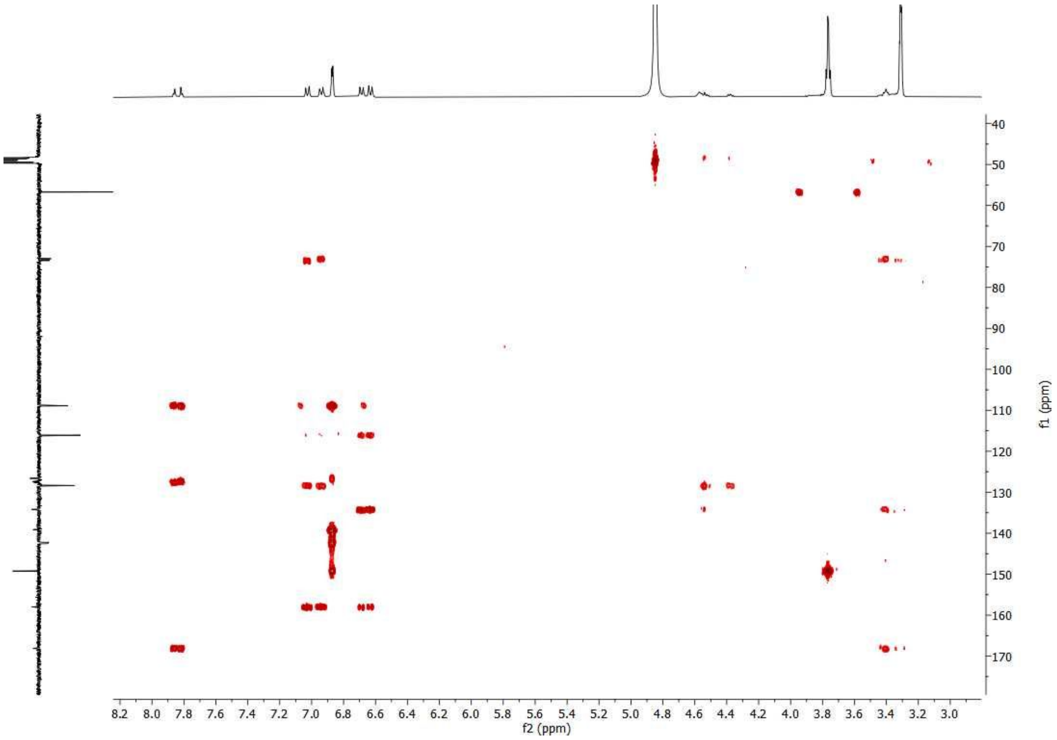

Figure S6. HMBC spectrum of compound **1 (**400/100 MHz, MeOD).

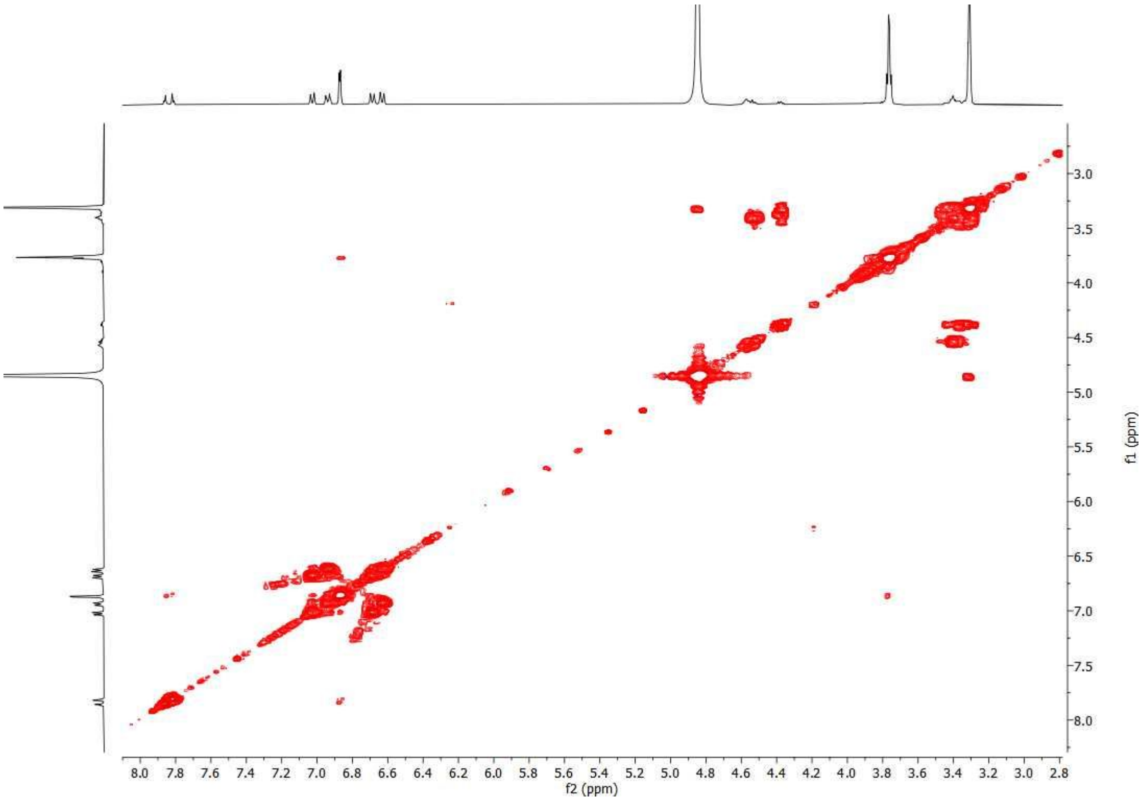

Figure S7. COSY spectrum of compound **1 (**400 MHz, MeOD).

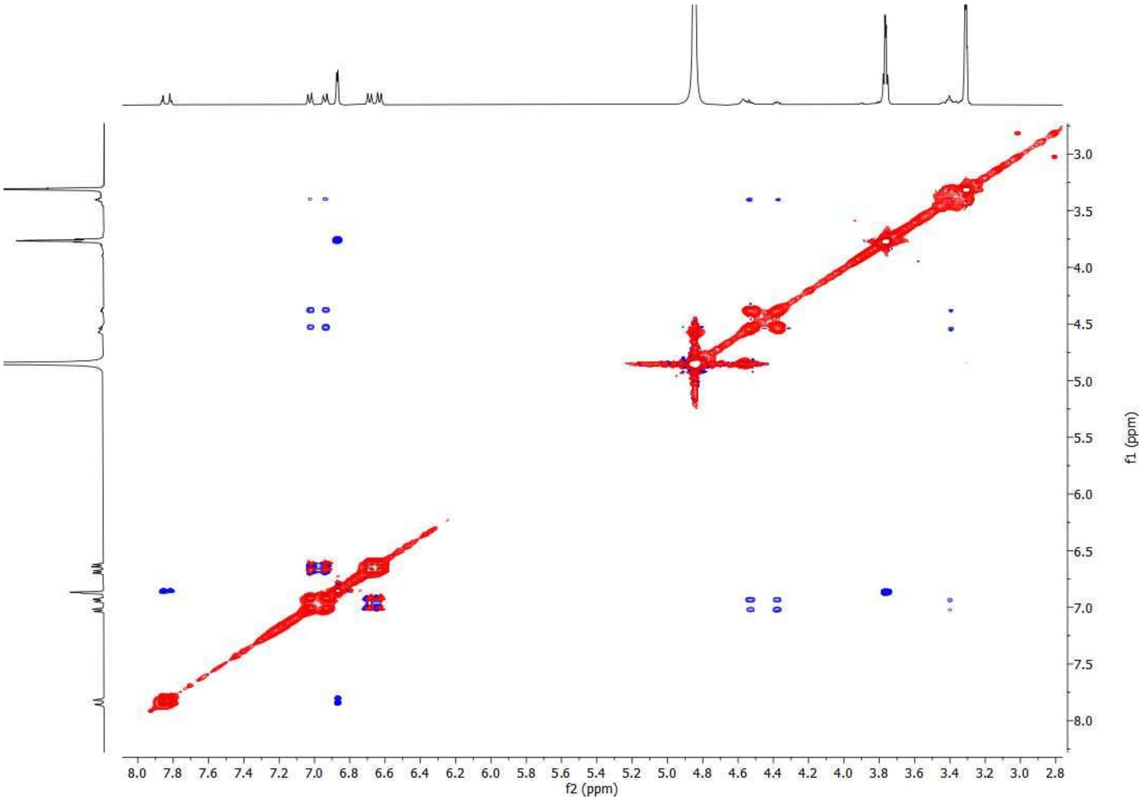

Figure S8. NOESY spectrum of compound **1 (**400 MHz, MeOD).

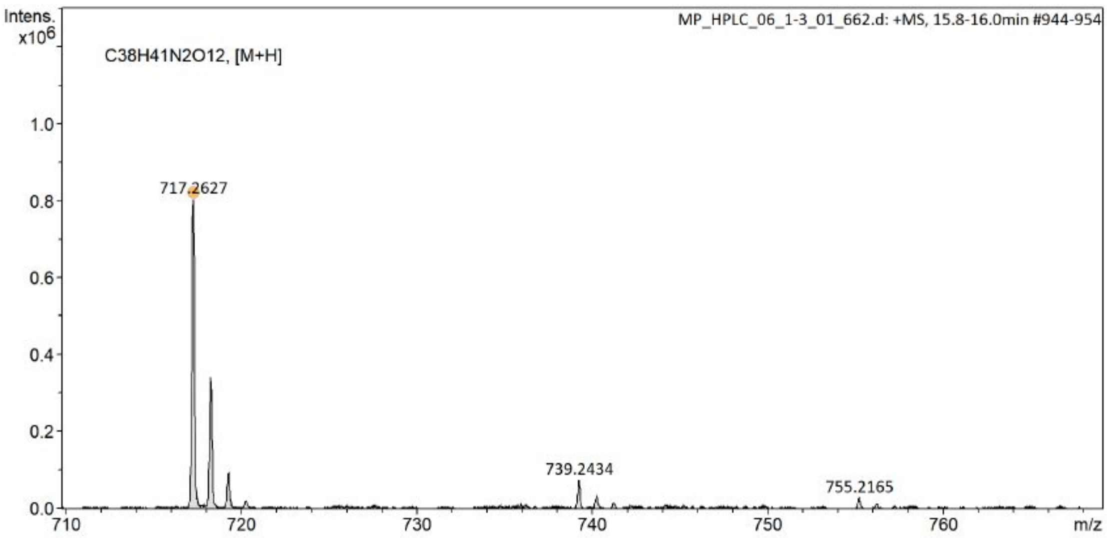

Figure S9. HRESIMS spectrum of compound **1** ($[M + H]^+$, positive mode).

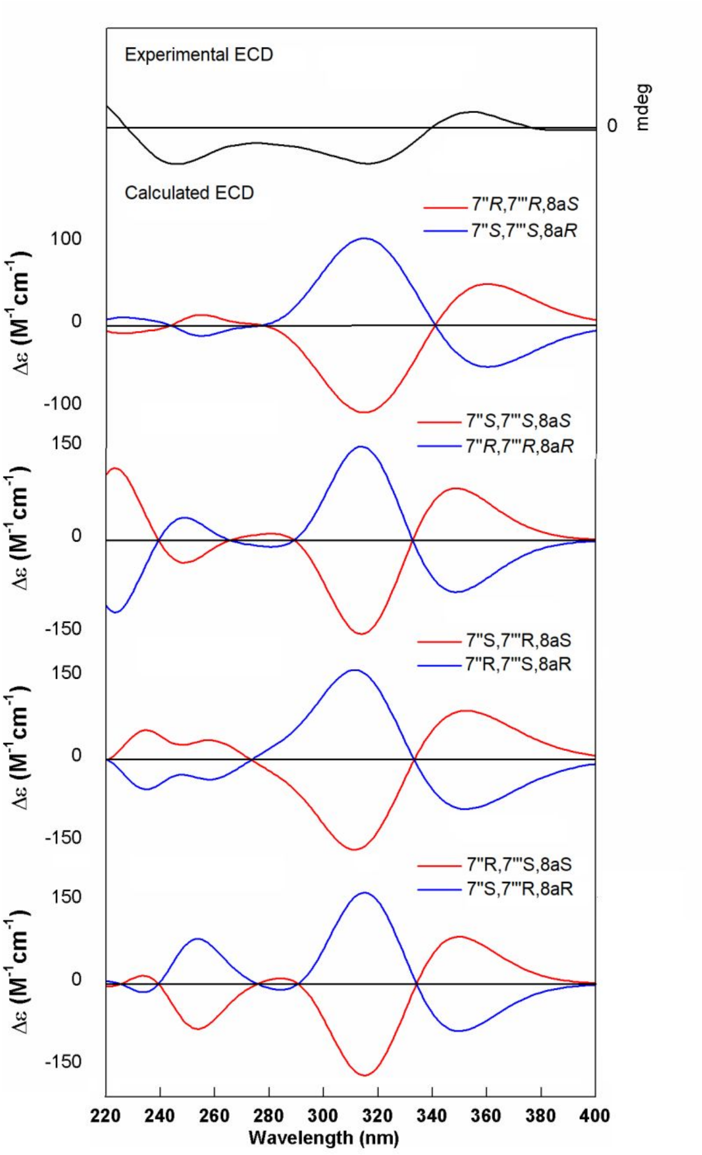

Figure S10. Experimental (top) and calculated (bottom) ECD spectra of all possible stereoisomers of compound **1**. a*S* atropisomers are marked in red and a*R* atropisomers are marked in blue.

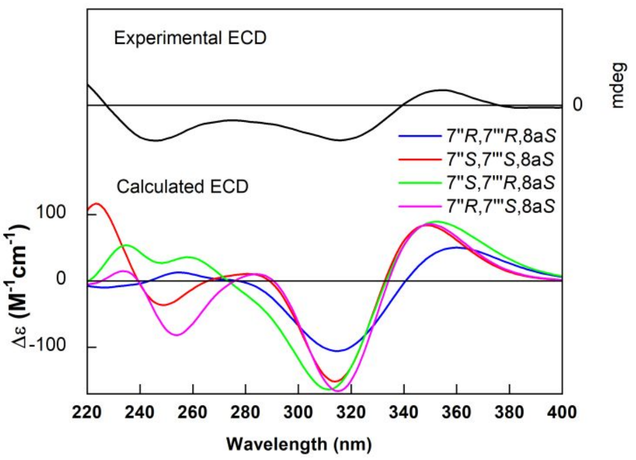

Figure S11. Experimental (top) and calculated (bottom) ECD spectra of a*S* atropoisomers of compound **1**.

2 Spectroscopic data for compound 2

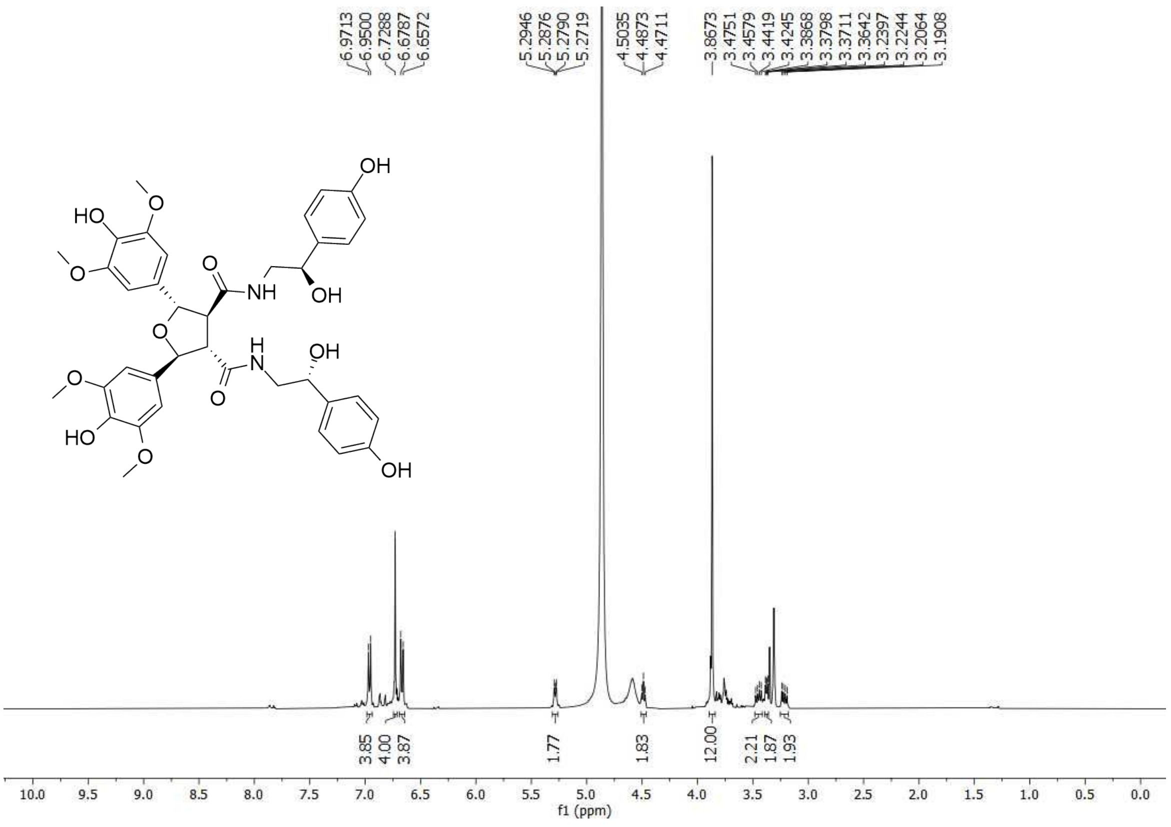

Figure S12. ^{1}H NMR spectrum of compound **2** (400 MHz, MeOD).

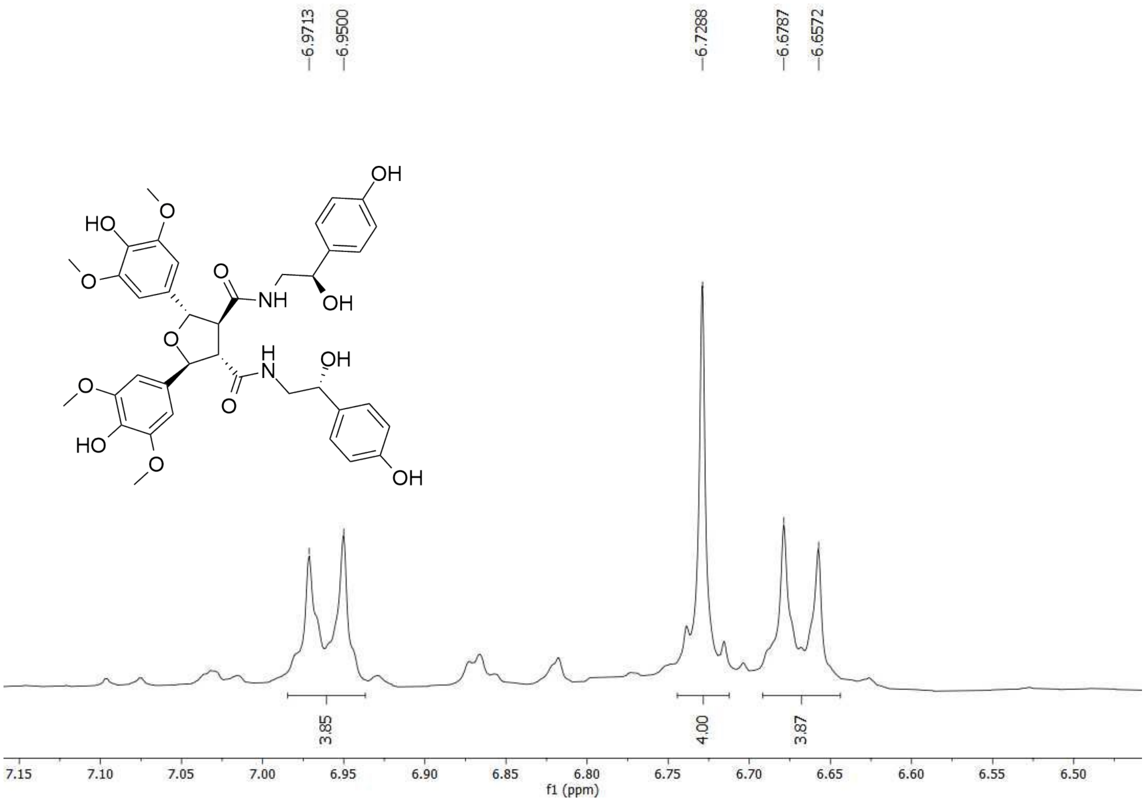

Figure S13. Amplification of the ^{1}H NMR spectrum of compound **2 (**400 MHz, MeOD).

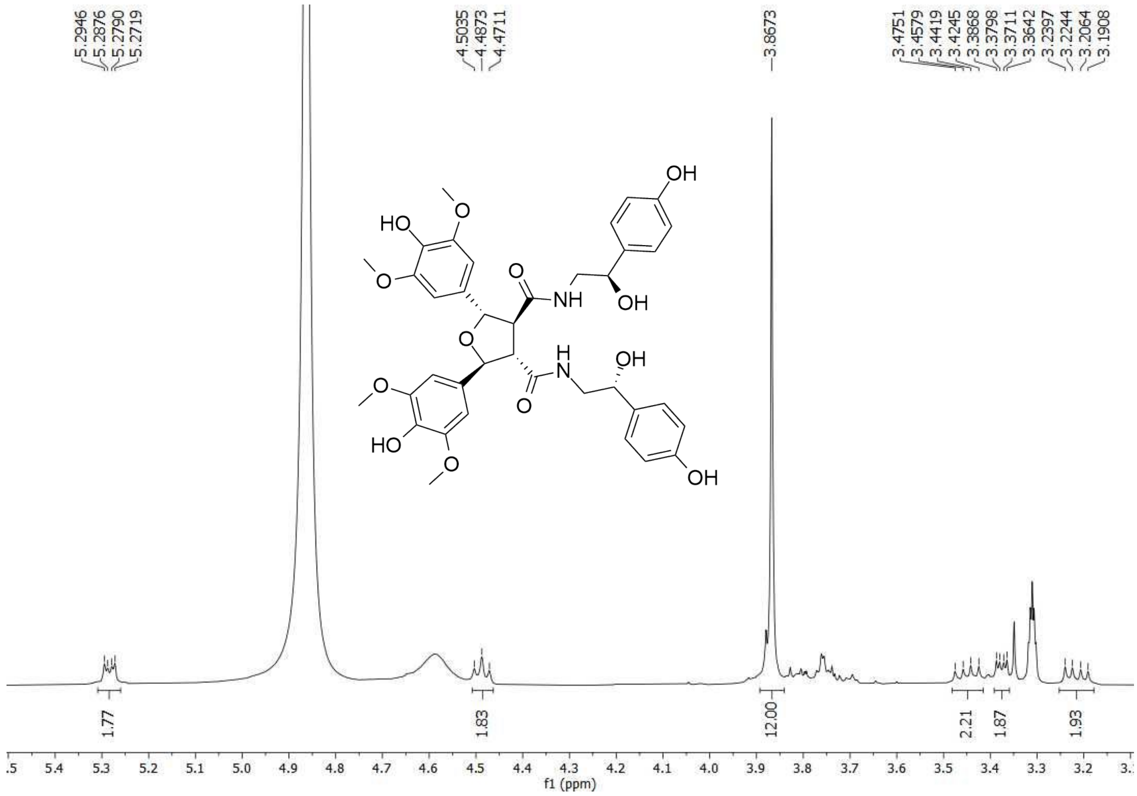

Figure S14. Amplification of the ^{1}H NMR spectrum of compound **2 (**400 MHz, MeOD).

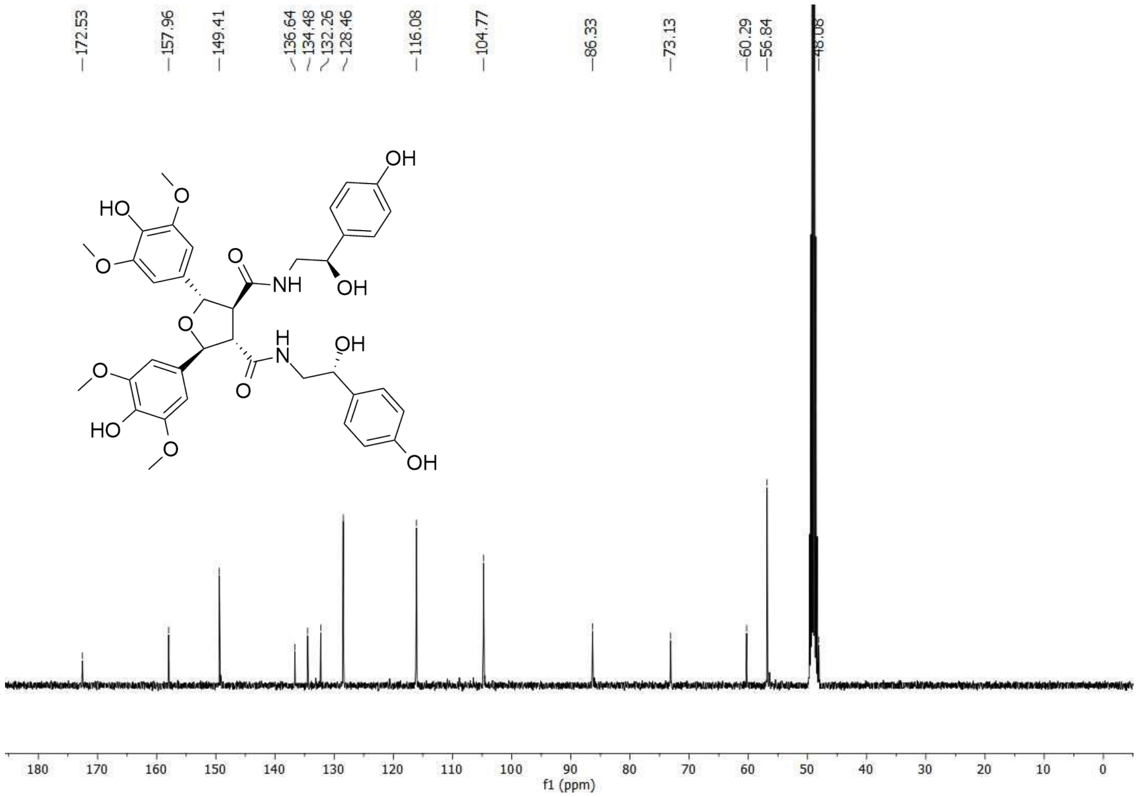

Figure S15. ^{13}C-BB NMR spectrum of compound **2** (100 MHz, MeOD).

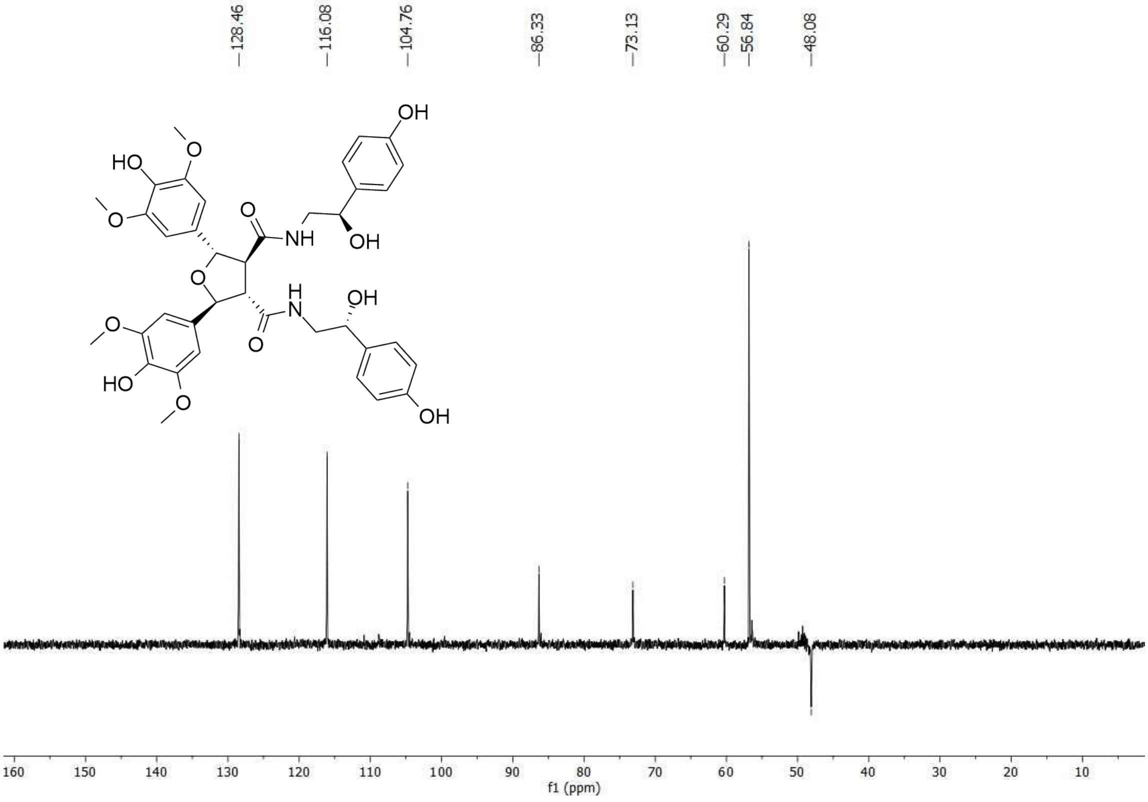

Figure S16. ^{13}C-DEPT135 NMR spectrum of compound **2** (100 MHz, MeOD).

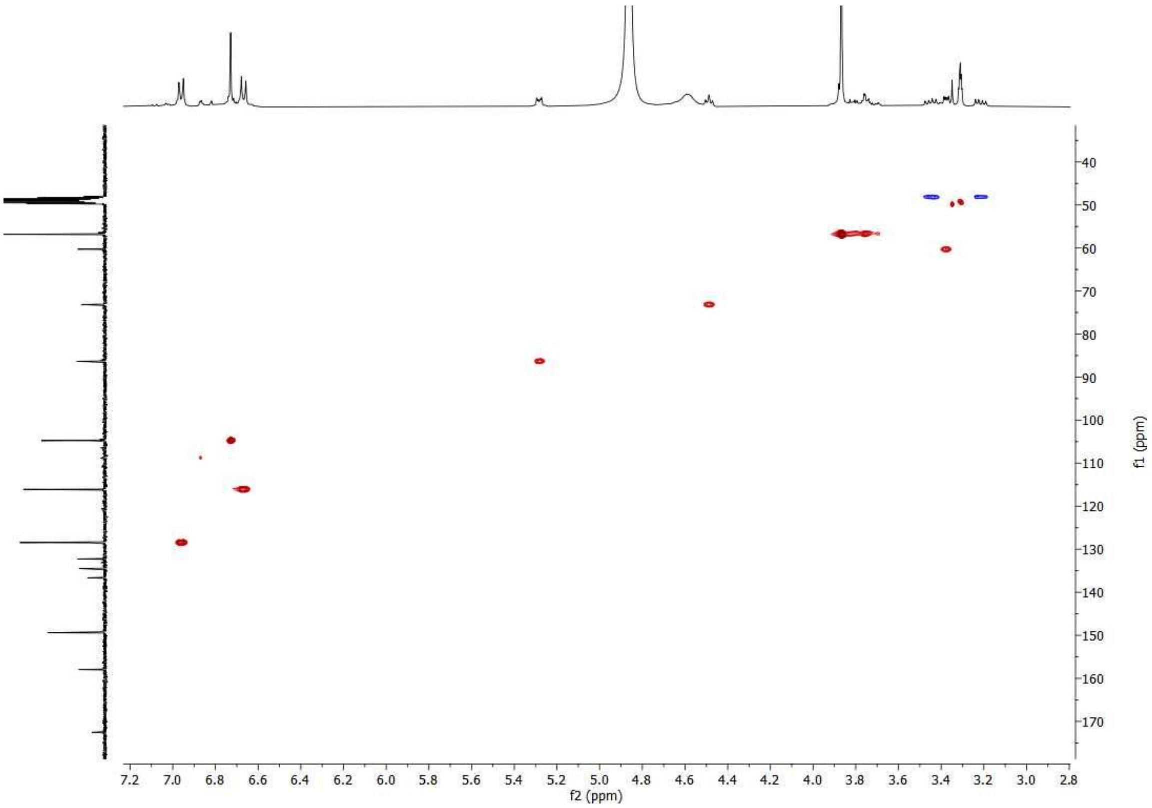

Figure S17. HSQC spectrum of compound **2** (400/100 MHz, MeOD).

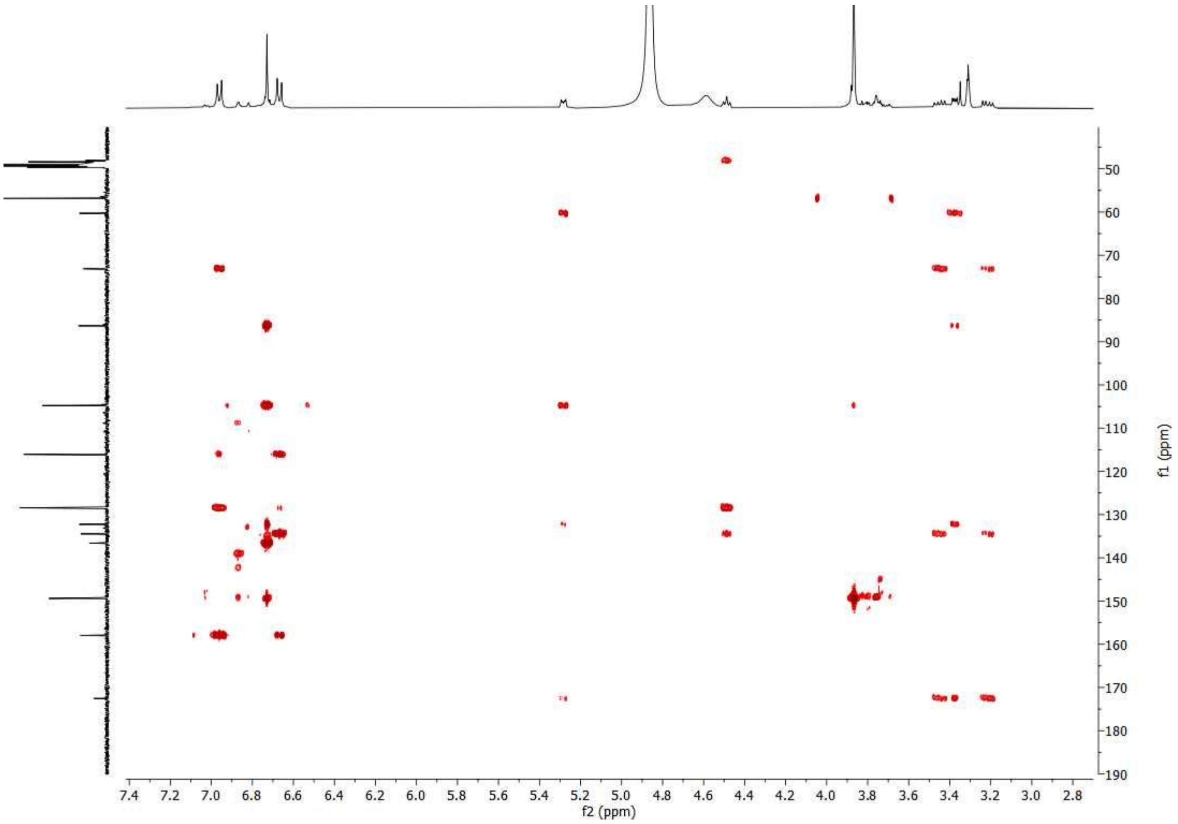

Figure S18. HMBC spectrum of compound **2** (400/100 MHz, MeOD).

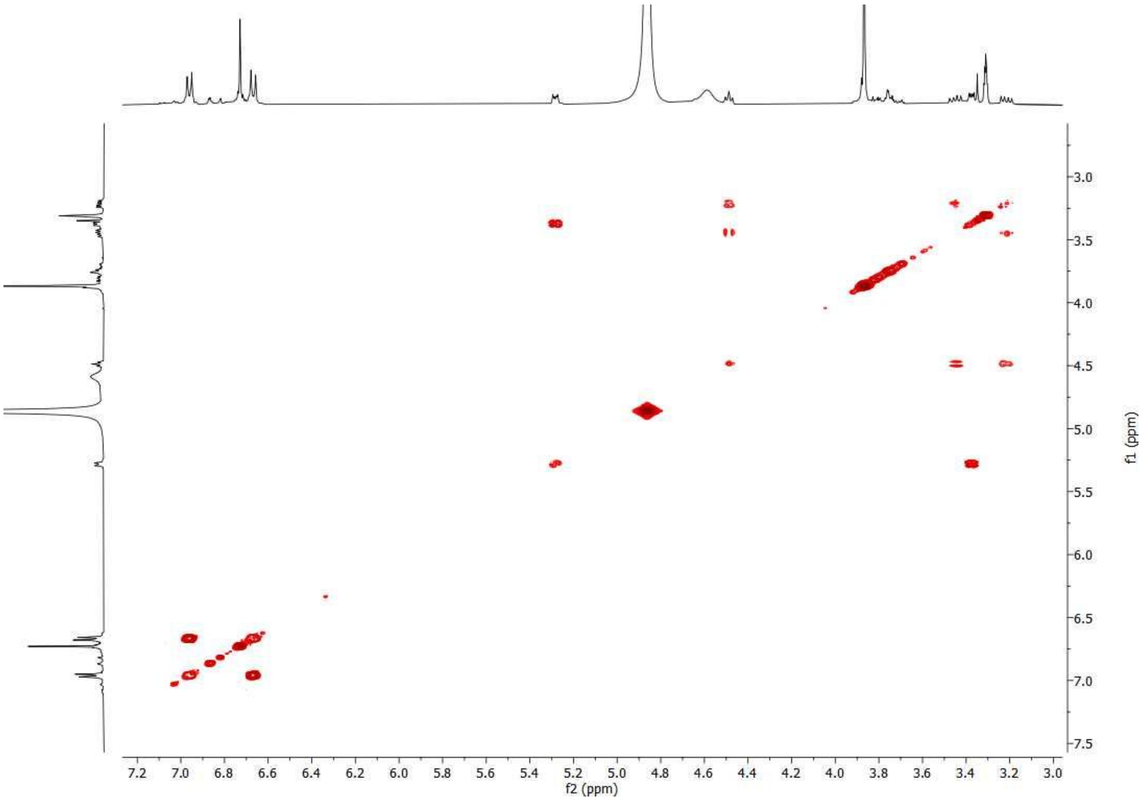

Figure S19. COSY spectrum of compound **2** (400 MHz, MeOD).

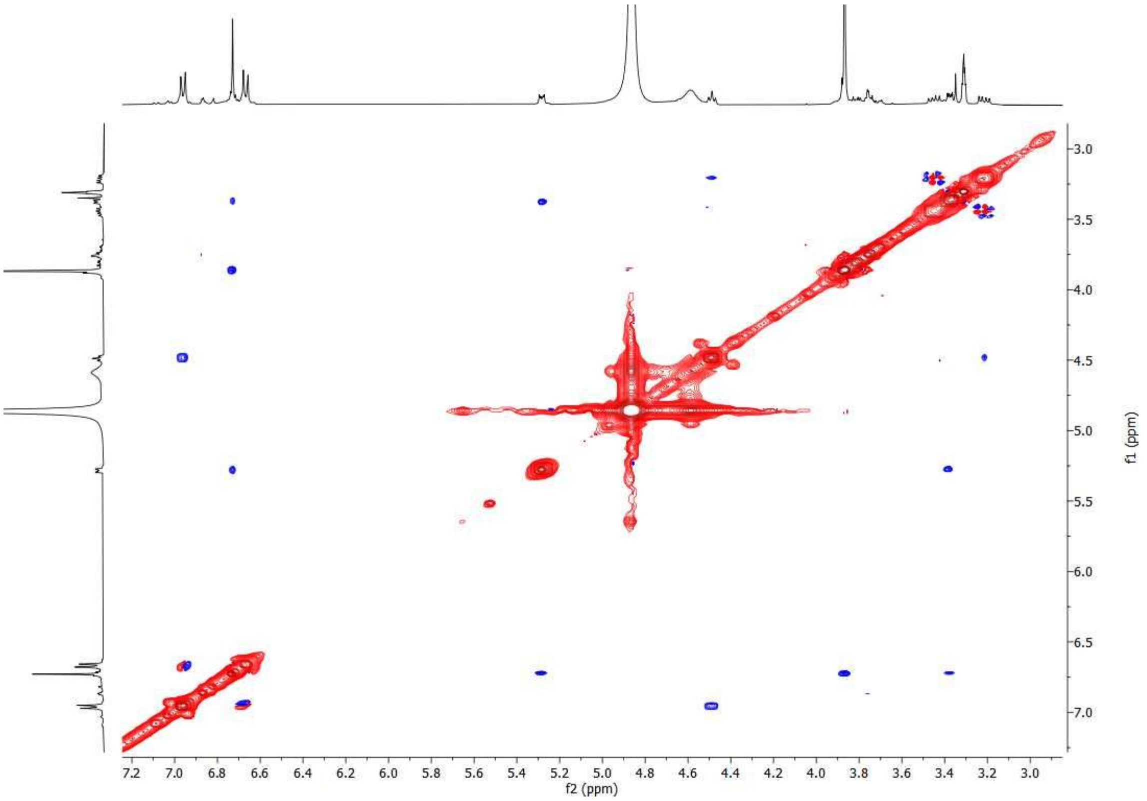

Figure S20. NOESY spectrum of compound **2** (400 MHz, MeOD).

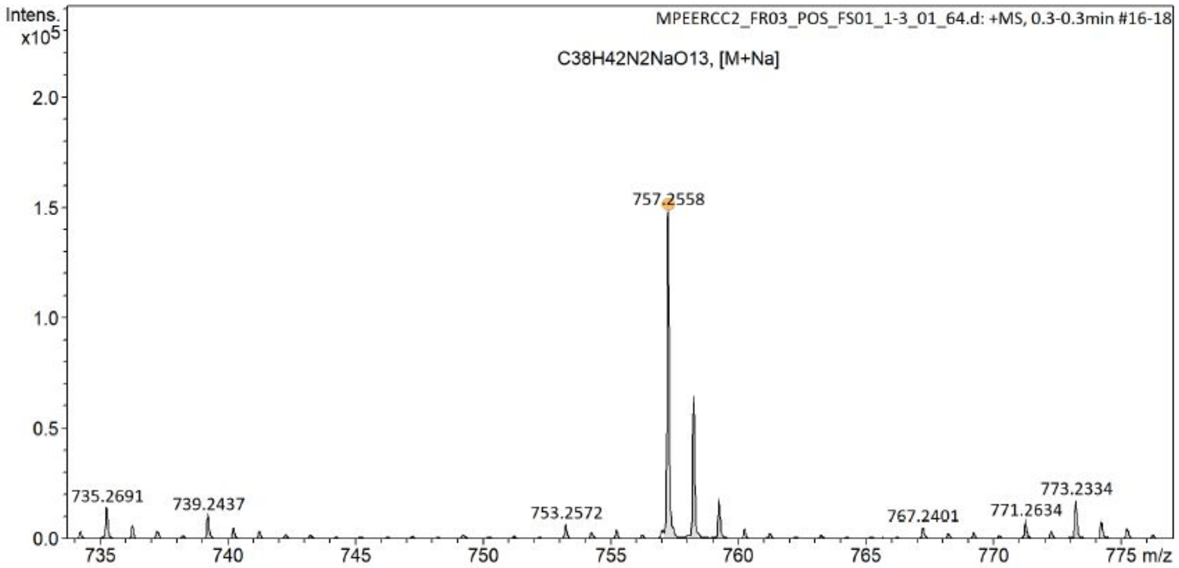

Figure S21. HRESIMS spectrum of compound **2** ([M + Na]$^+$, positive mode).

3 Spectroscopic data for Compound 3

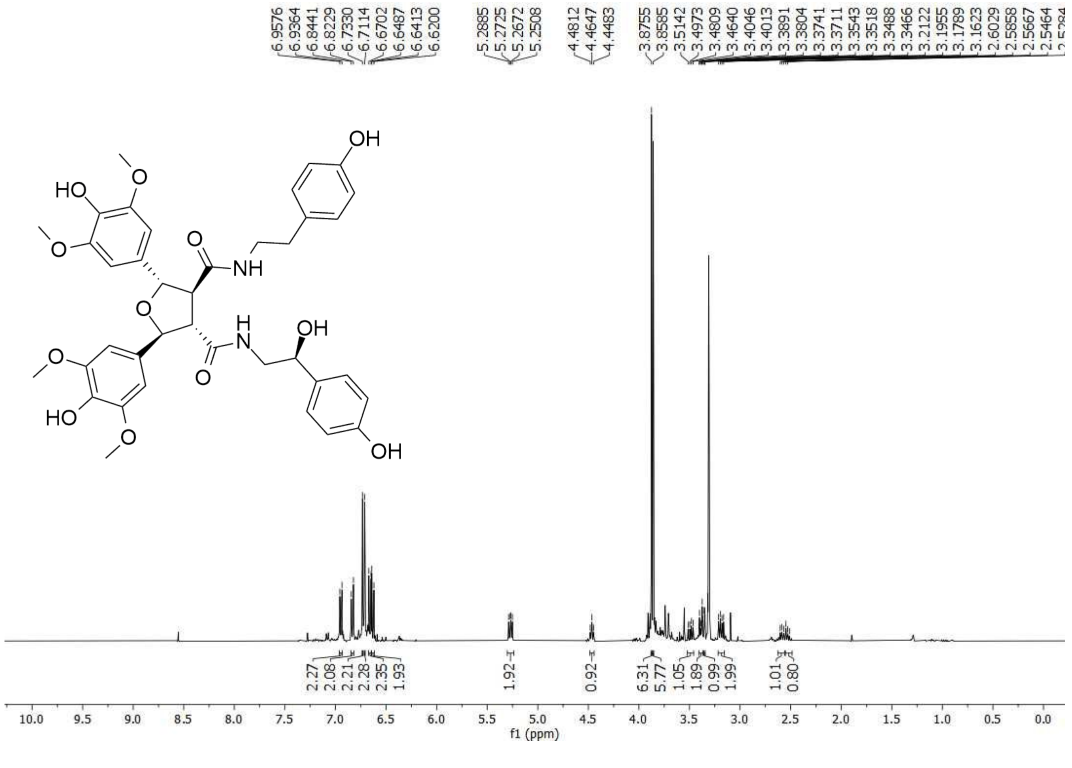

Figure S22. ^{1}H NMR spectrum of compound **3** (400 MHz, MeOD).

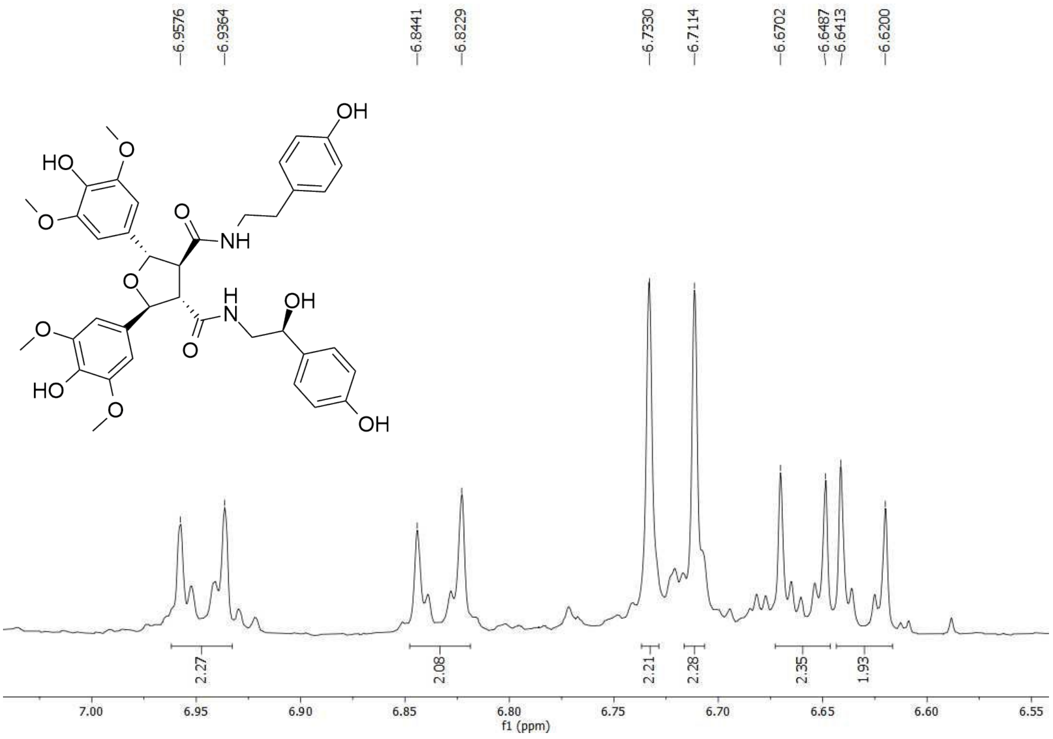

Figure S23. Amplification of the ^{1}H NMR spectrum of compound **3** (400 MHz, MeOD).

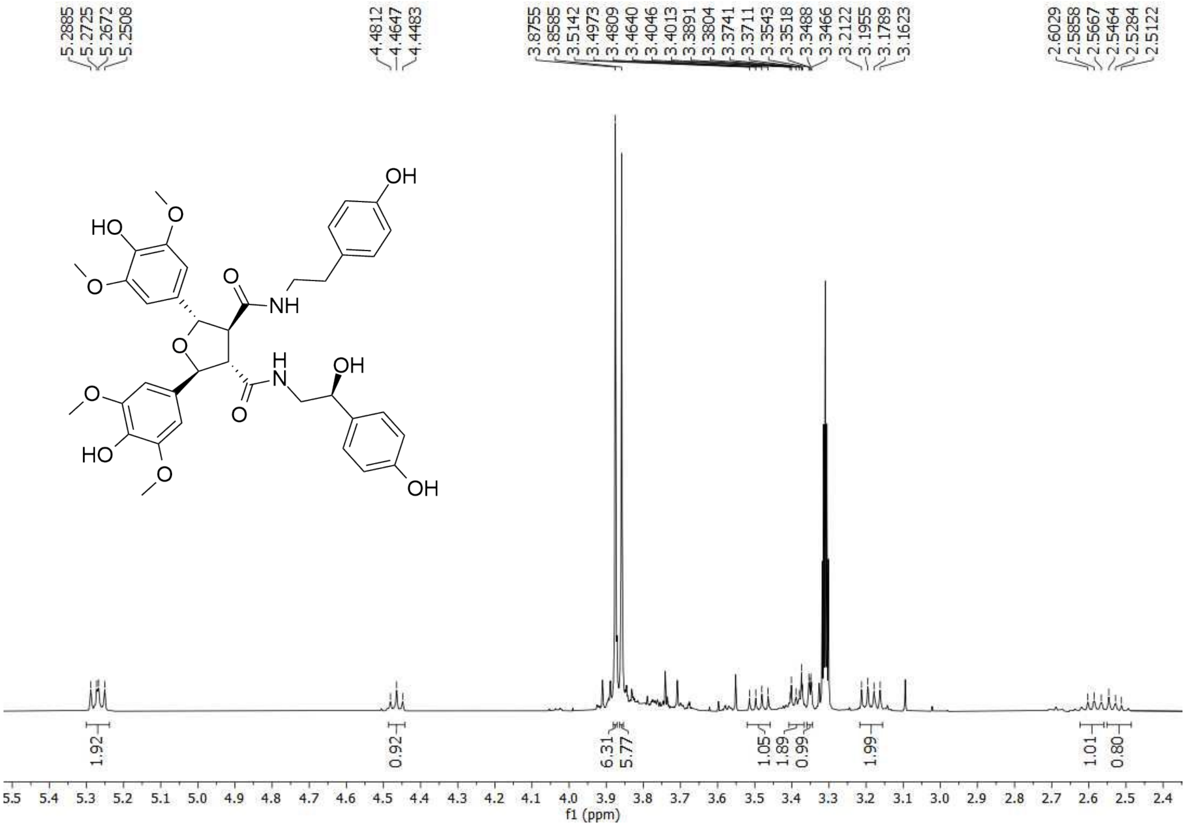

Figure S24. Amplification of the ^{1}H NMR spectrum of compound **3** (400 MHz, MeOD).

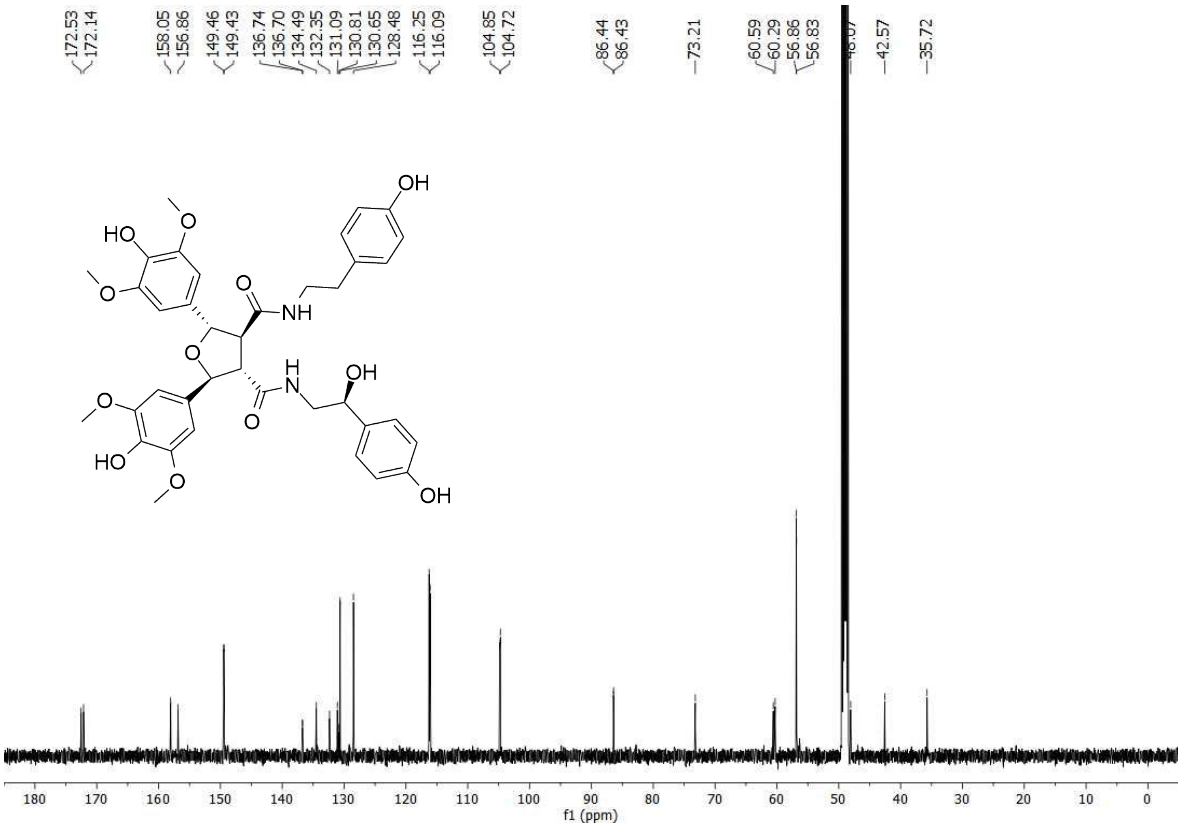

Figure S25. ^{13}C-BB NMR spectrum of compound **3** (100 MHz, MeOD).

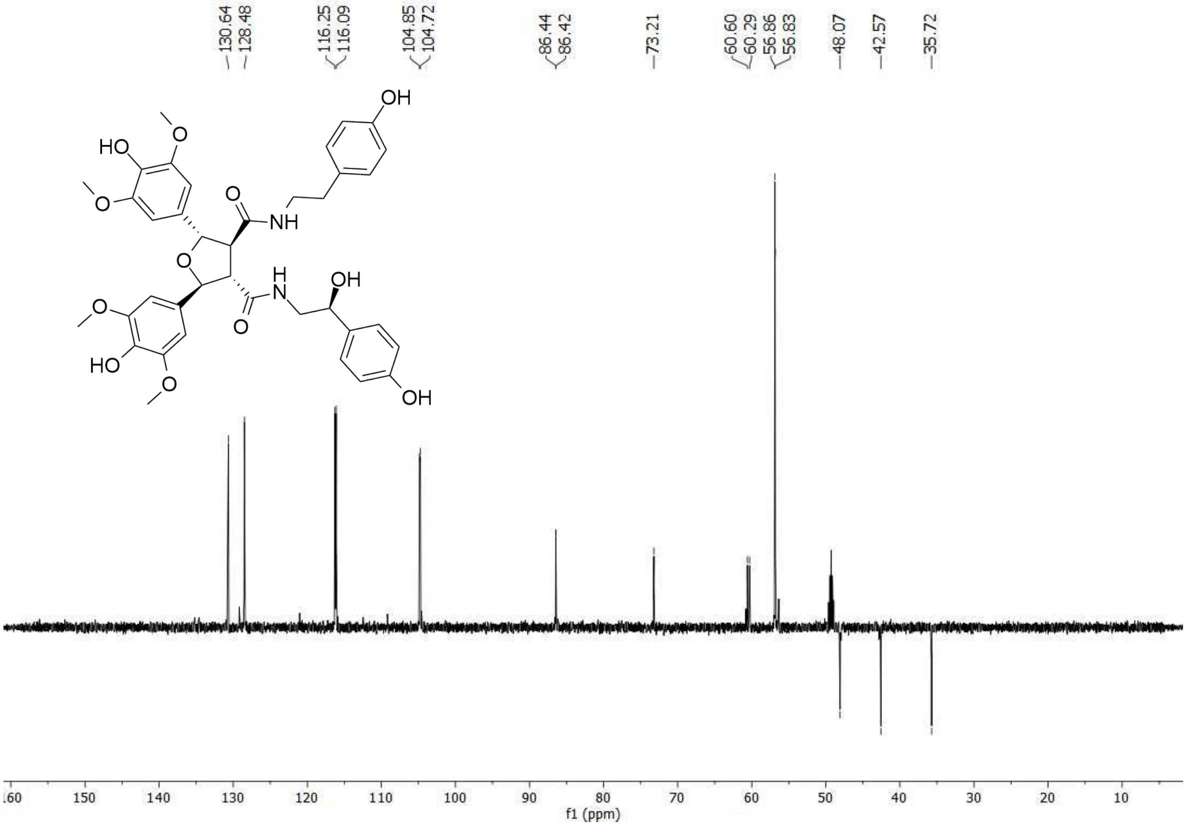

Figure S26. ^{13}C-DEPT135 NMR spectrum of compound **3** (100 MHz, MeOD).

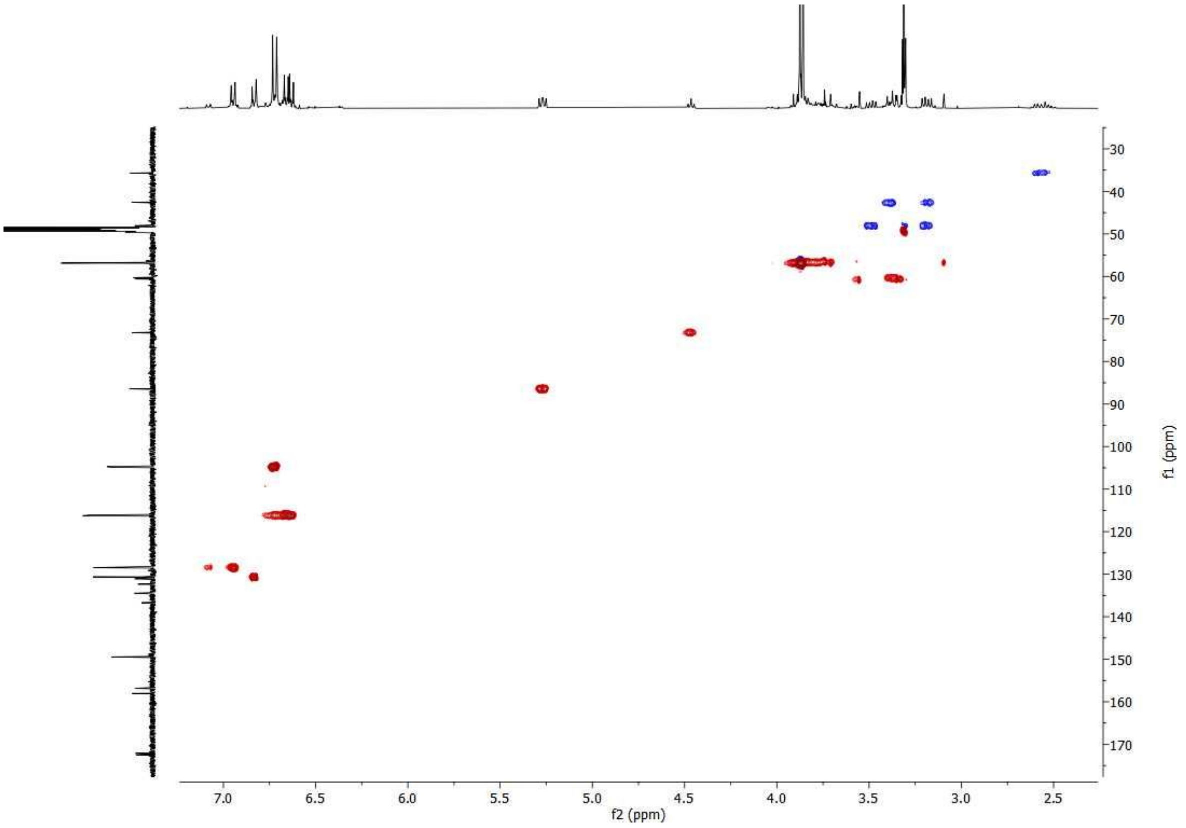

Figure S27. HSQC spectrum of compound **3 (**400/100 MHz, MeOD).

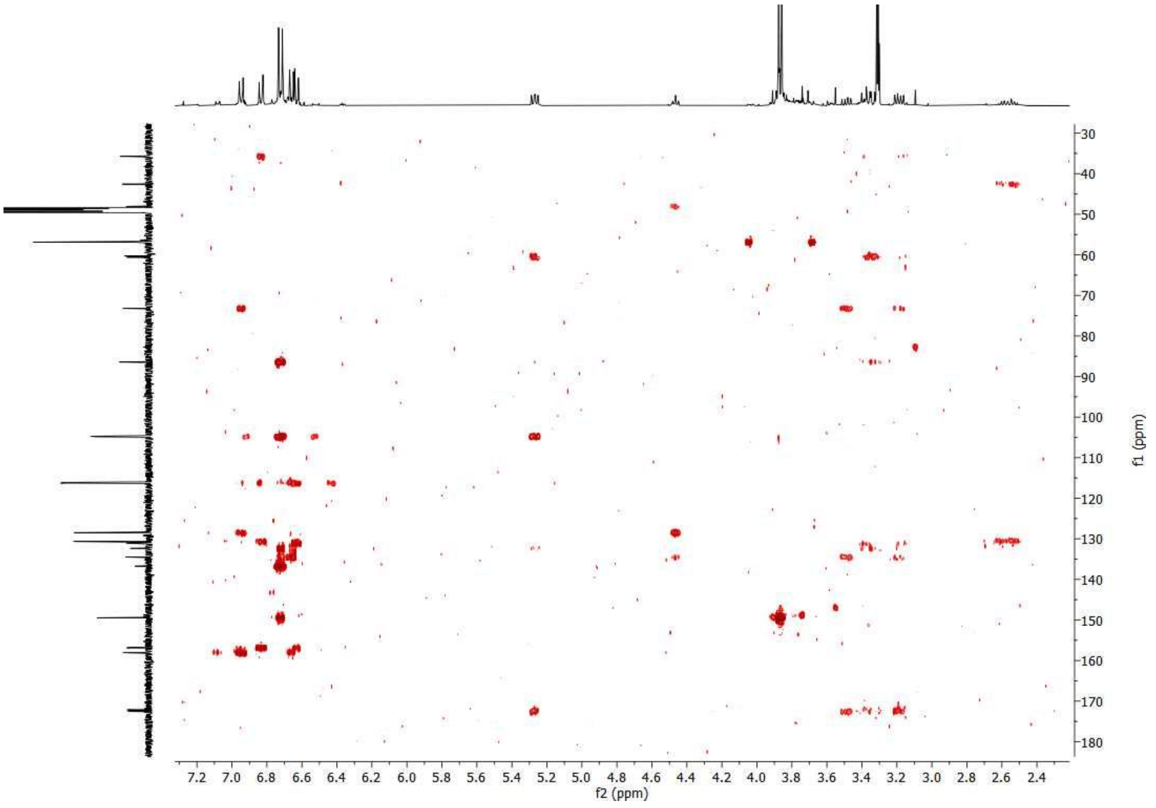

Figure S28. HMBC spectrum of compound **3 (**400/100 MHz, MeOD).

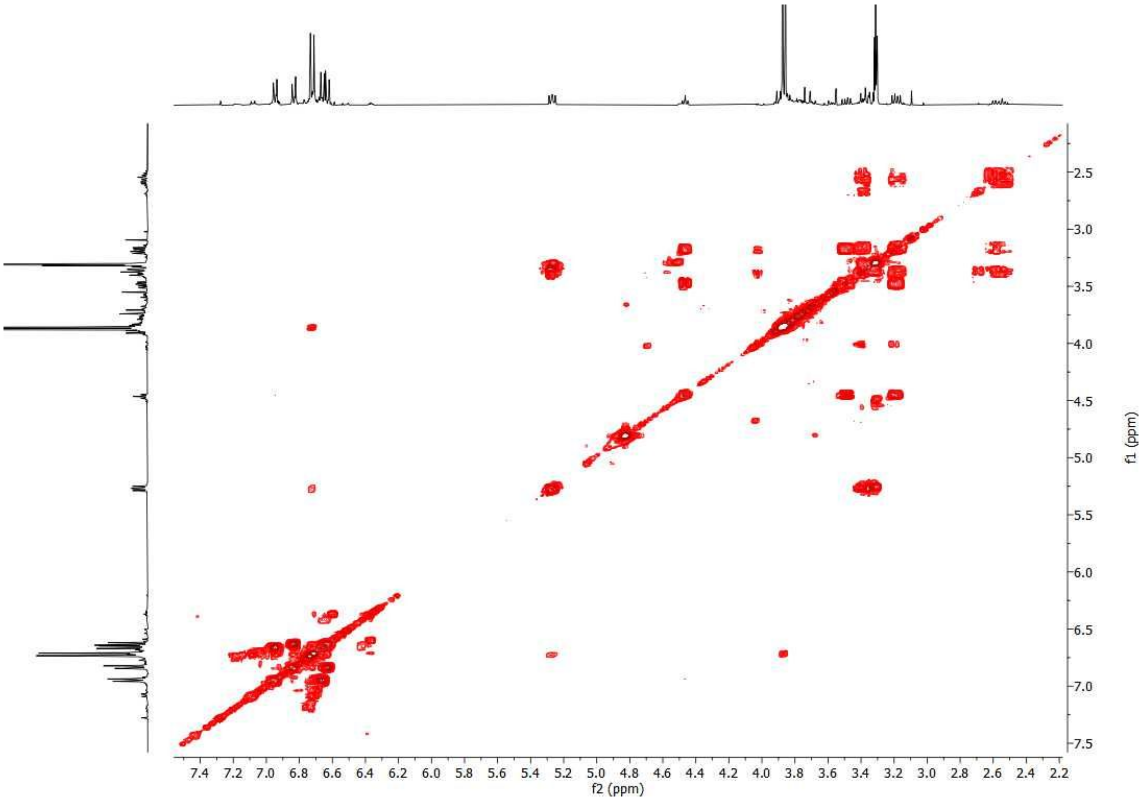

Figure S29. COSY spectrum of compound **3** **(**400 MHz, MeOD).

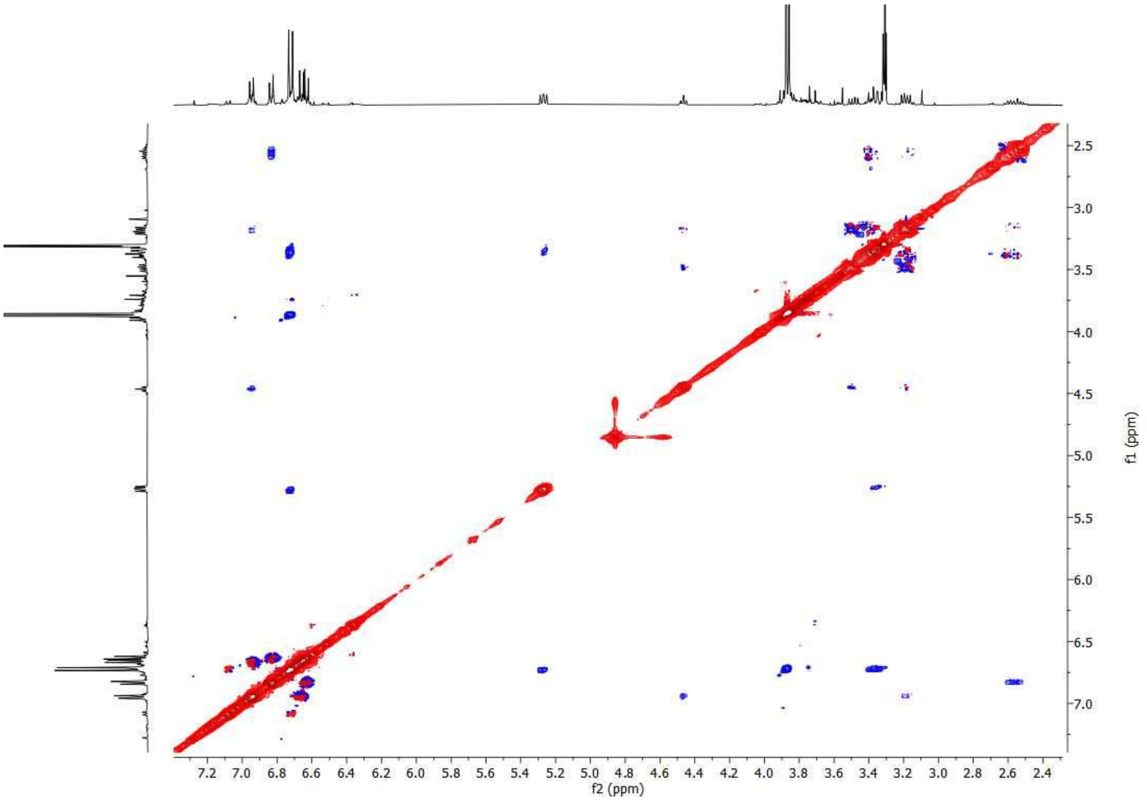

Figure S30. NOESY spectrum of compound **3** **(**400 MHz, MeOD).

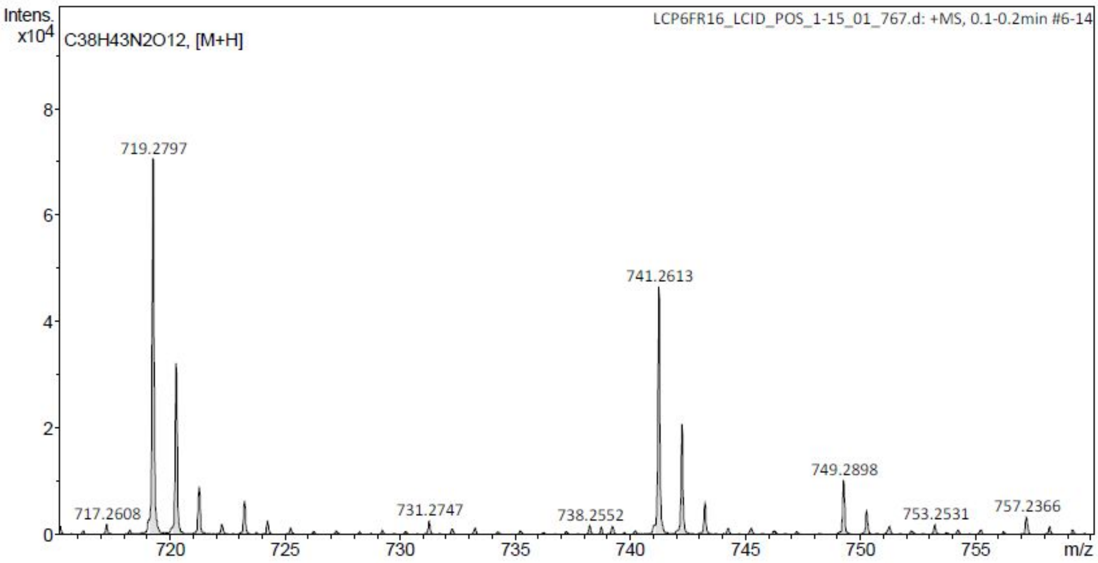

Figure S31. HRESIMS spectrum of compound **3** ([M + H]$^+$, positive mode).

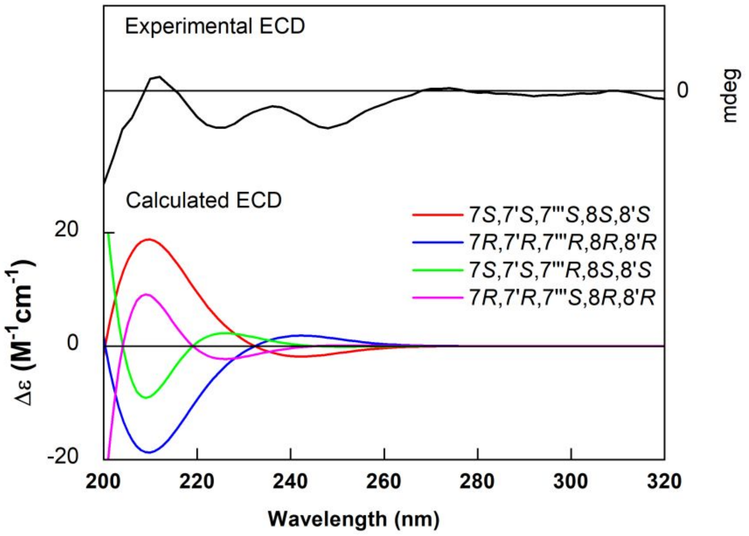

Figure S32. Experimental (top) and calculated (bottom) ECD spectra of all possible stereoisomers of compound **3**.

4 Spectroscopic data for Compound 4

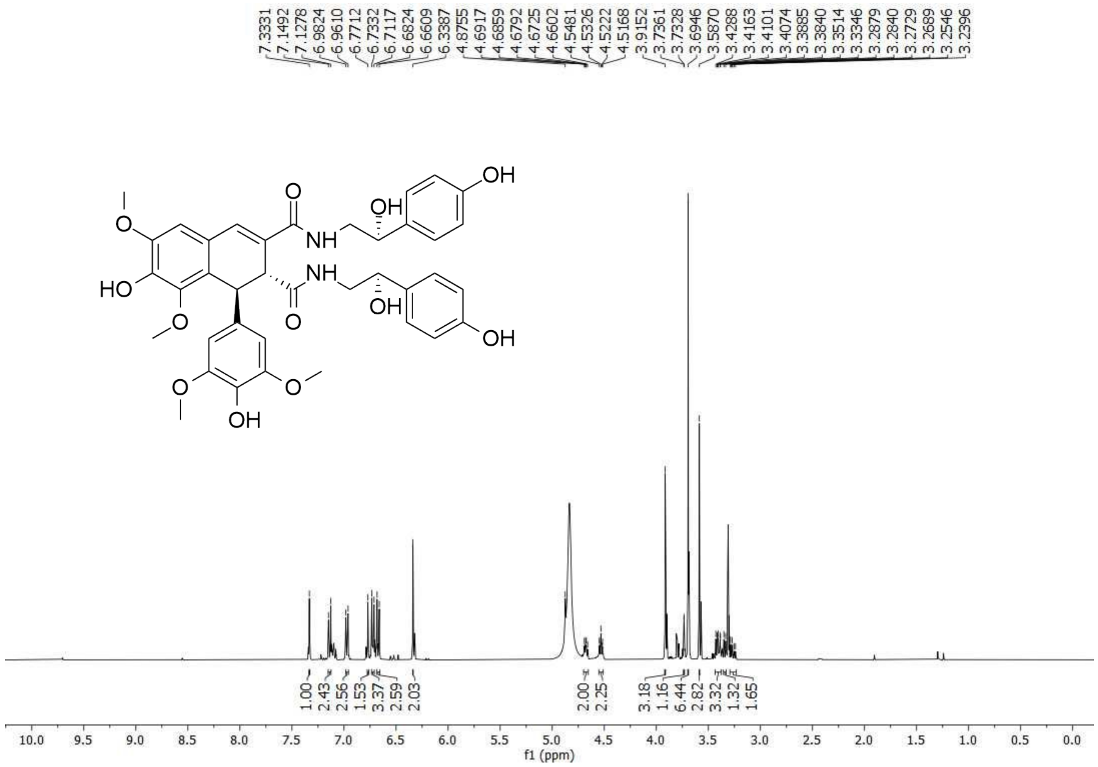

Figure S33. ^{1}H NMR spectrum of compound **4** (400 MHz, MeOD).

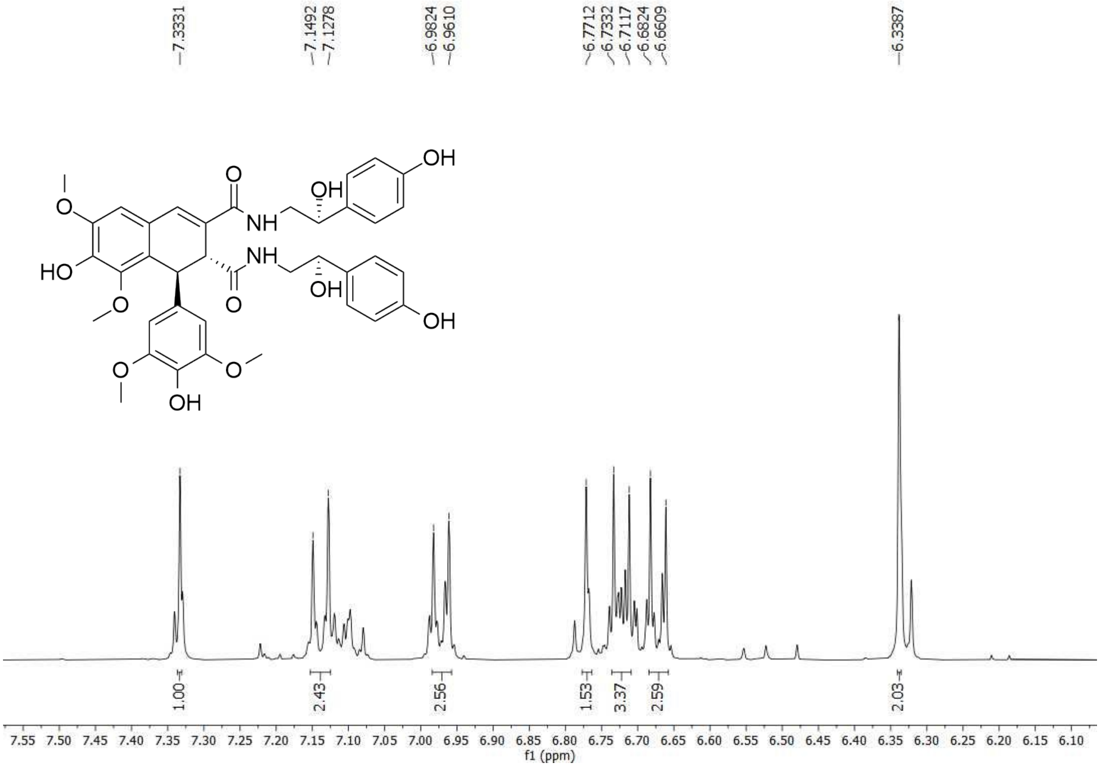

Figure S34. Amplification of the ^{1}H NMR spectrum of compound **4** (400 MHz, MeOD).

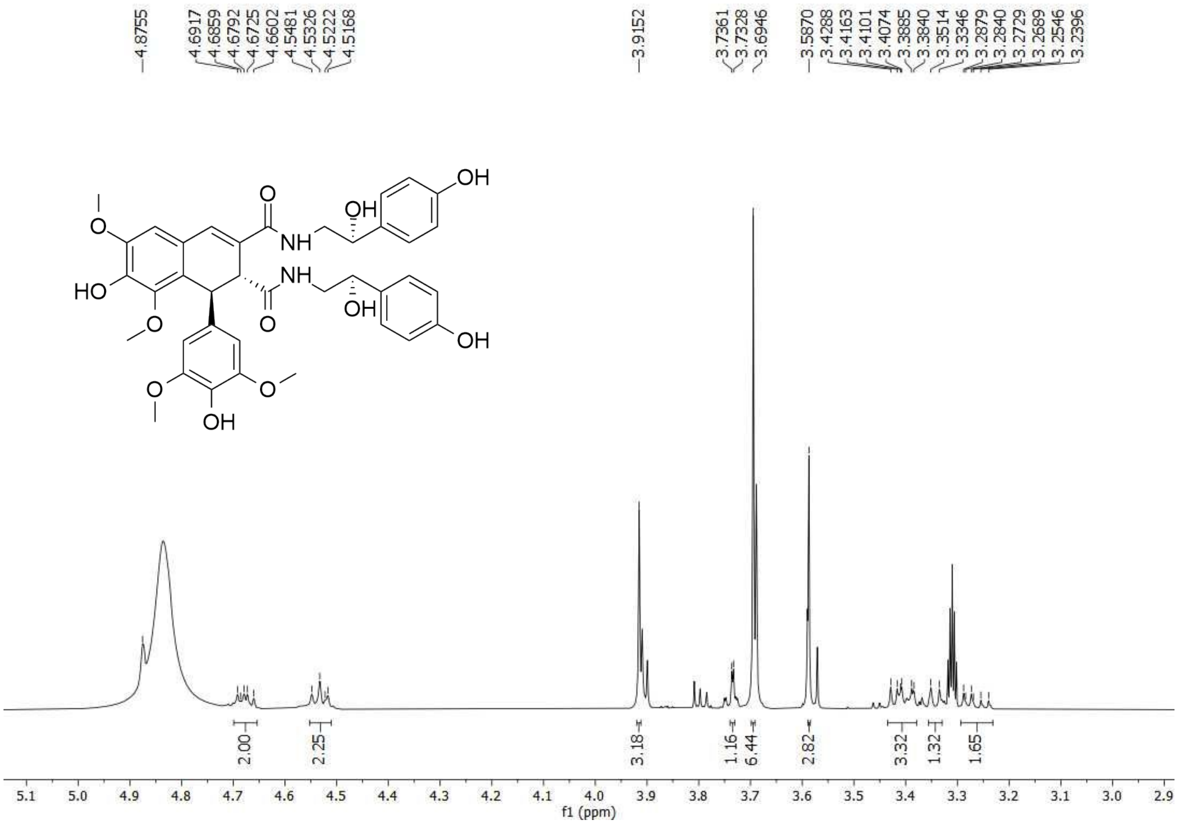

Figure S35. Amplification of the ^{1}H NMR spectrum of compound **4** (400 MHz, MeOD).

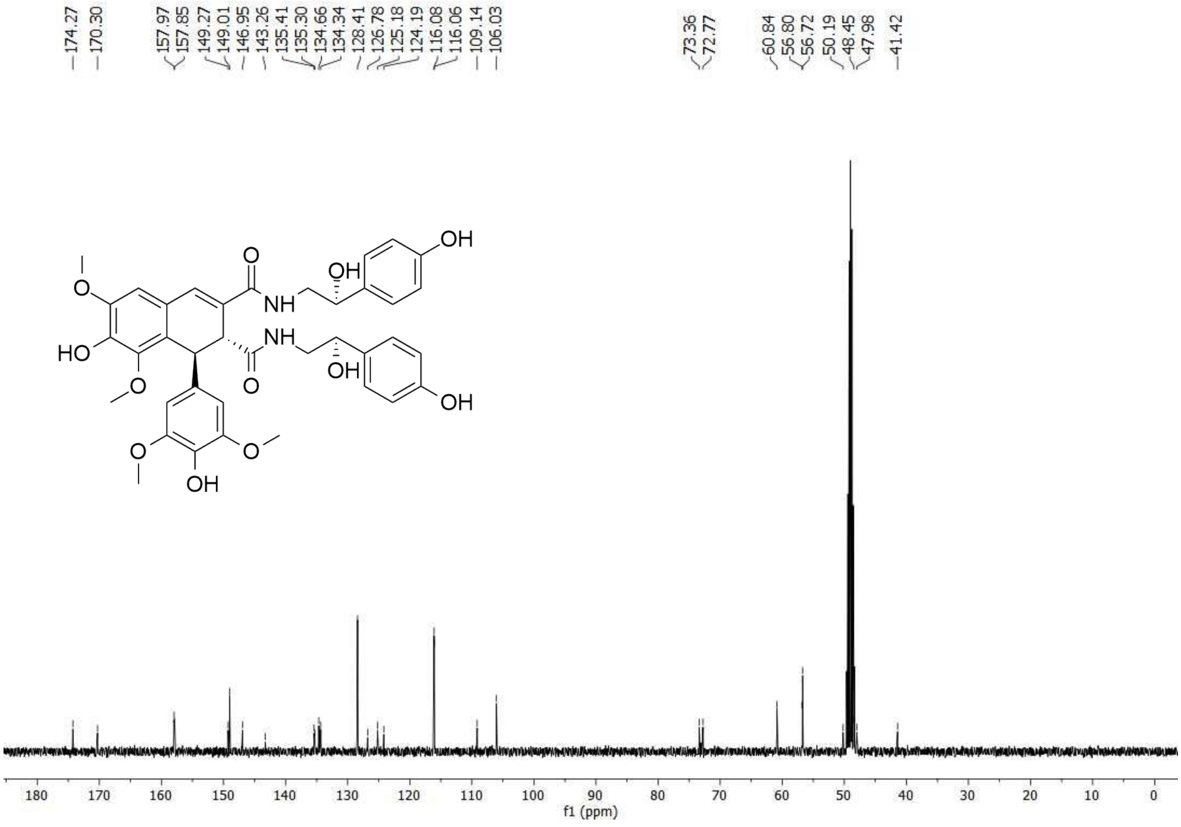

Figure S36. ^{13}C-BB NMR spectrum of compound **4** (100 MHz, MeOD).

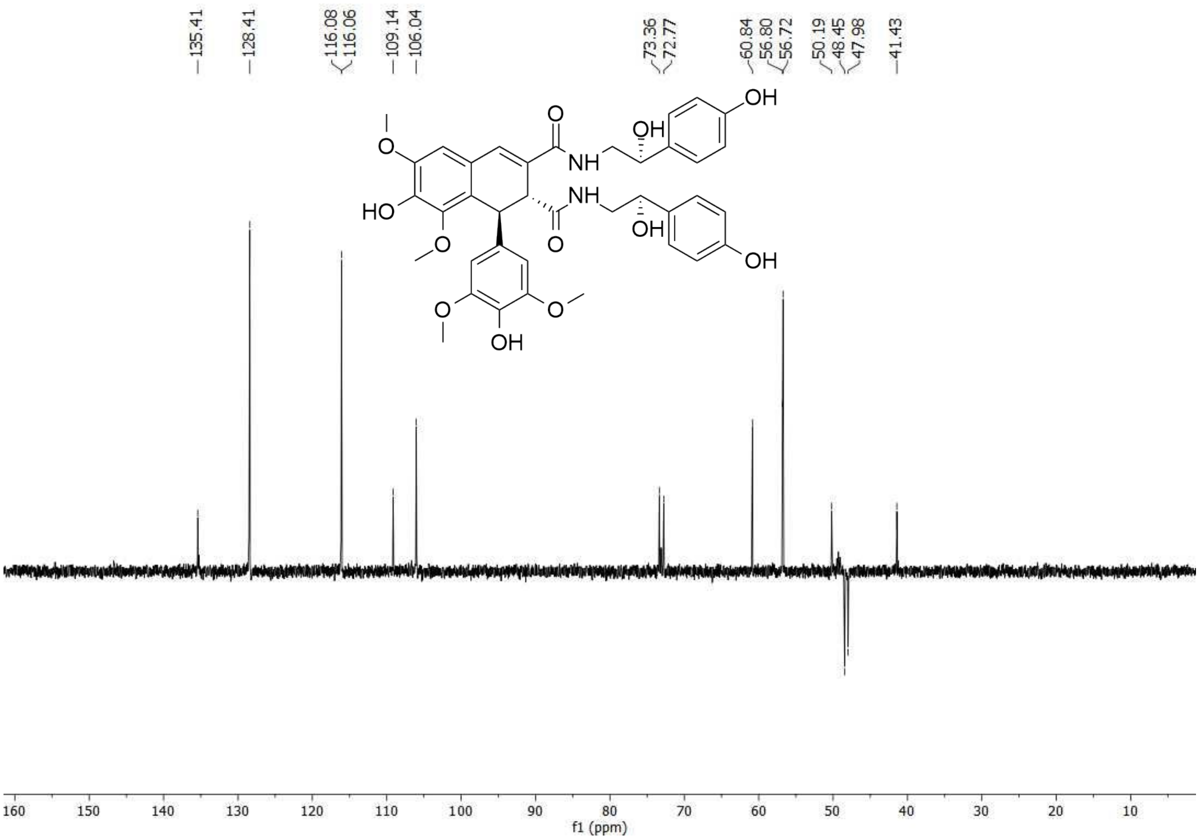

Figure S37. ^{13}C-DEPT135 NMR spectrum of compound **4** (100 MHz, MeOD).

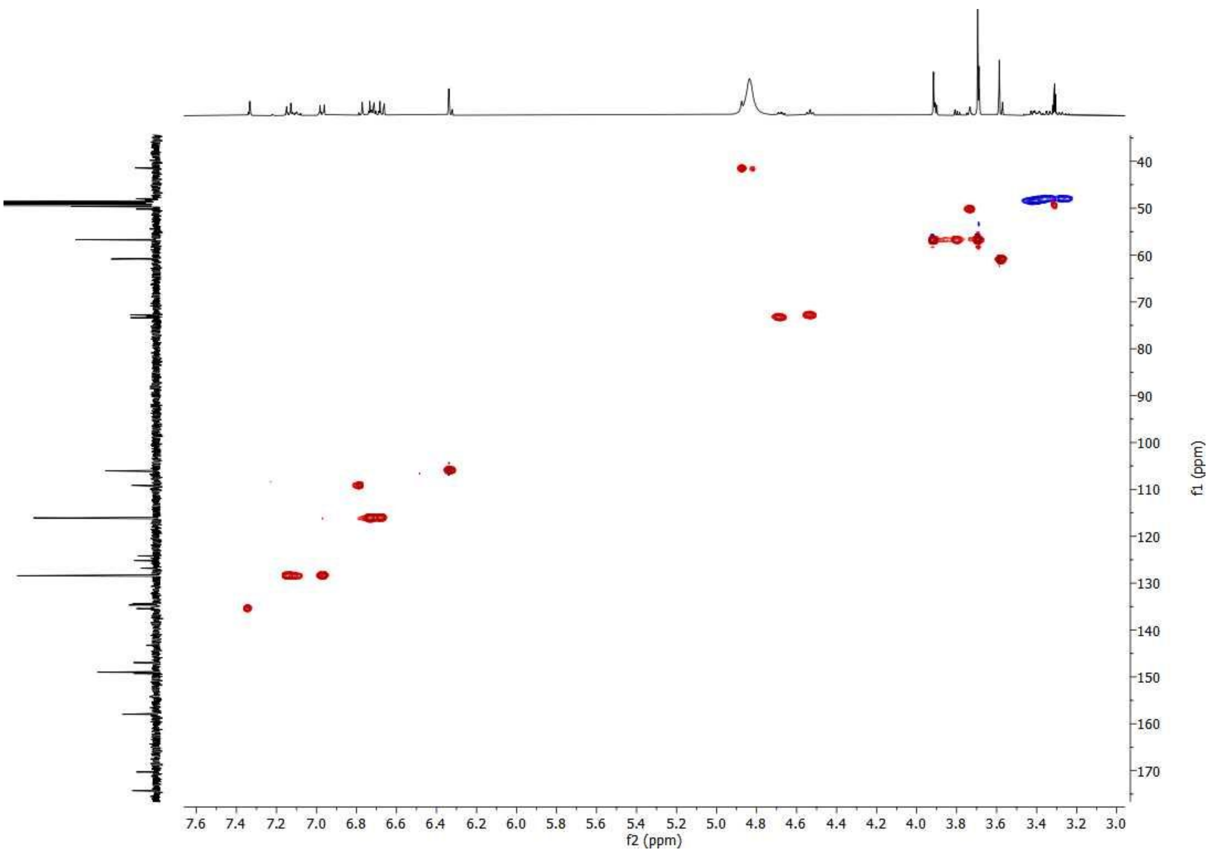

Figure S38. HSQC spectrum of compound **4** (400/100 MHz, MeOD).

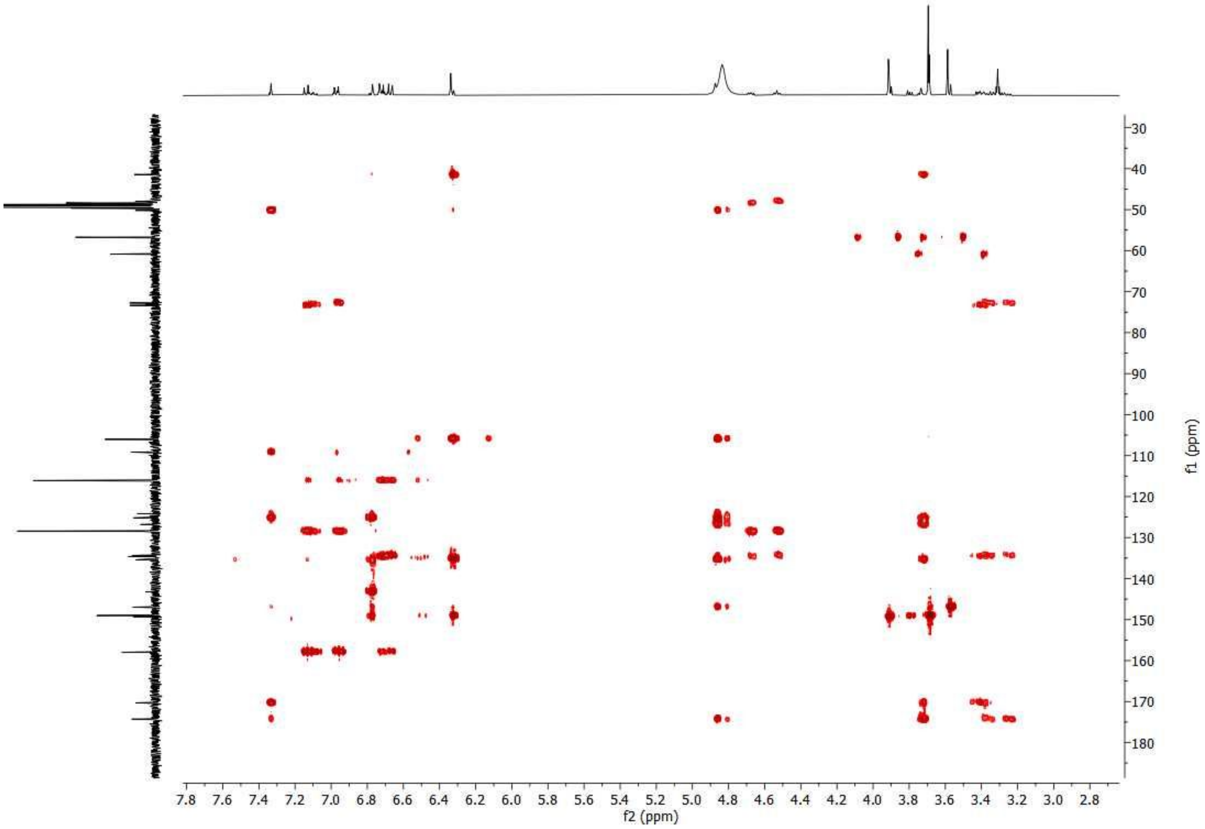

Figure S39. HMBC spectrum of compound **4 (**400/100 MHz, MeOD).

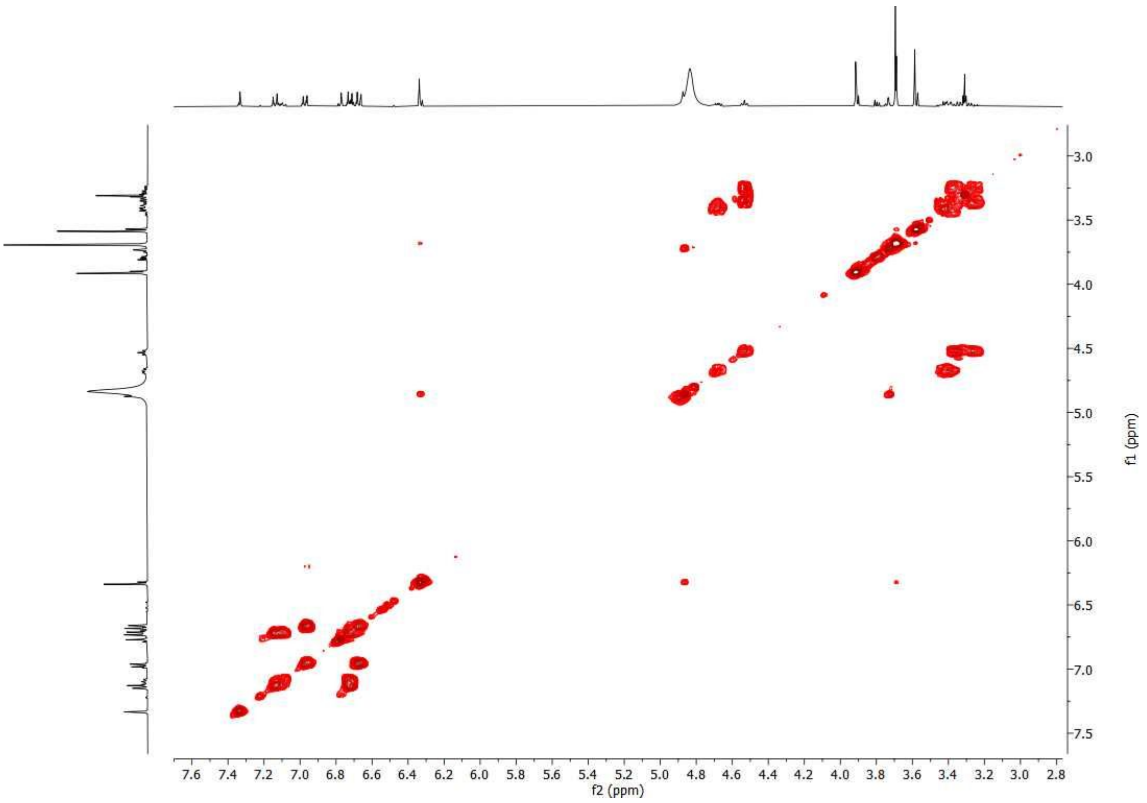

Figure S40. COSY spectrum of compound **4 (**400 MHz, MeOD).

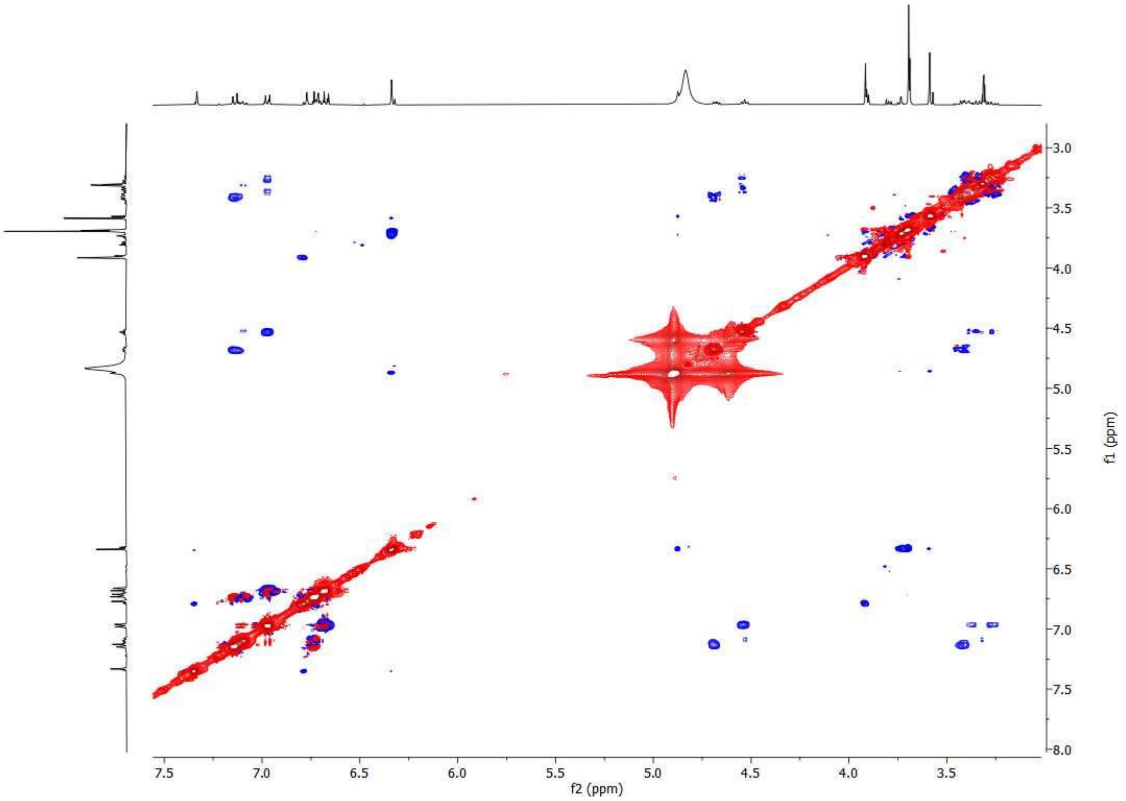

Figure S41. NOESY spectrum of compound **4 (**400 MHz, MeOD).

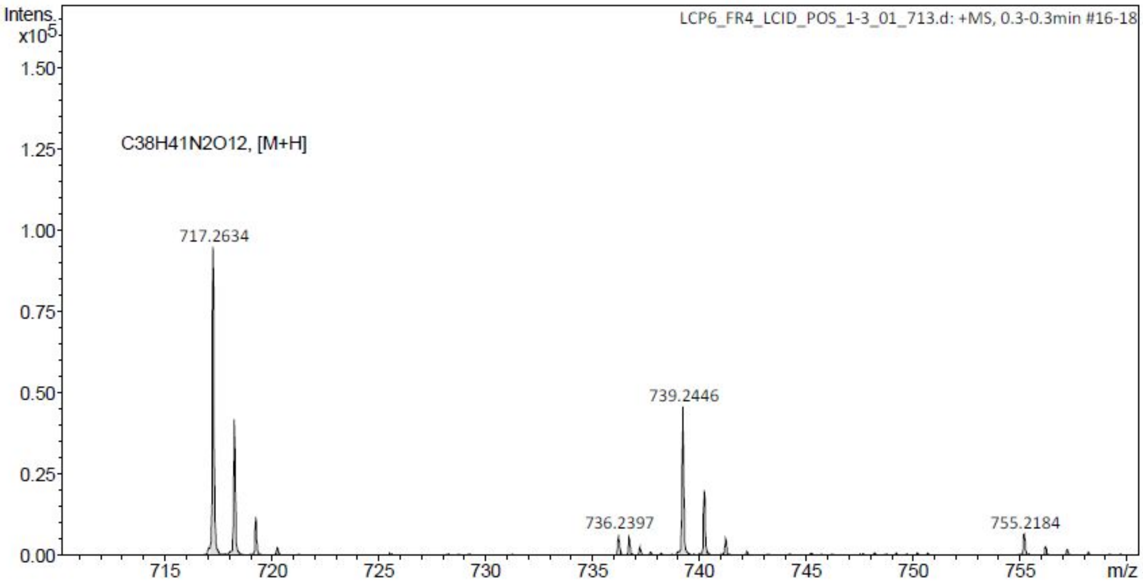

Figure S42. HRESIMS spectrum of compound **4** ($[M + H]^+$, positive mode).

5 Spectroscopic data for compound 5

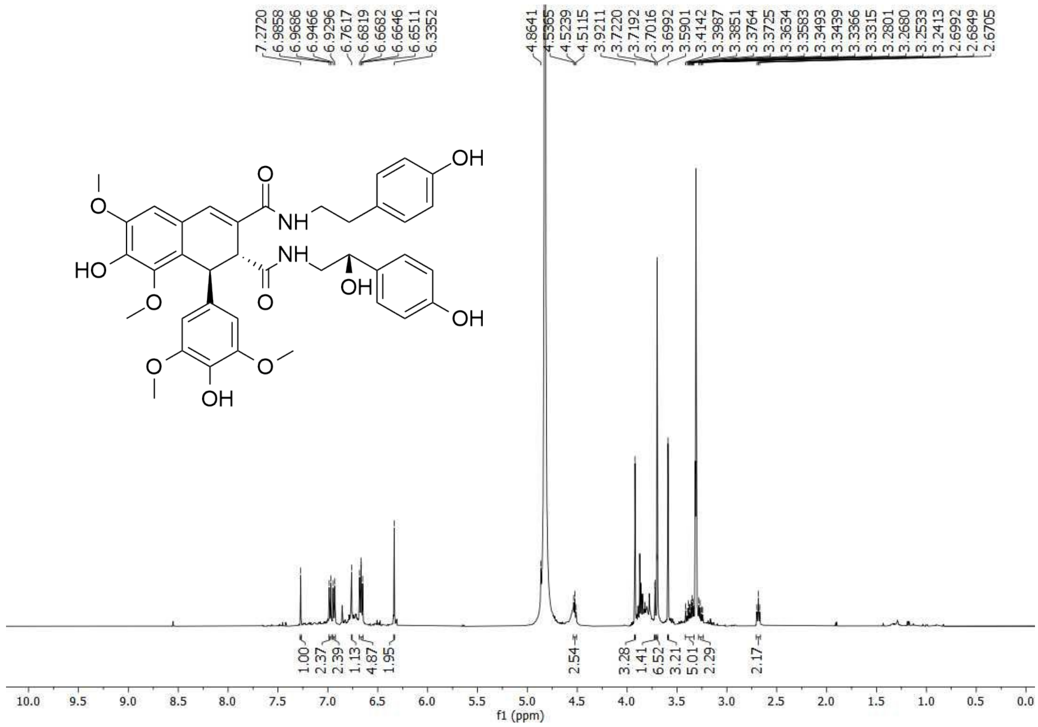

Figure S43. ^{1}H NMR spectrum of compound **5 (**500 MHz, MeOD).

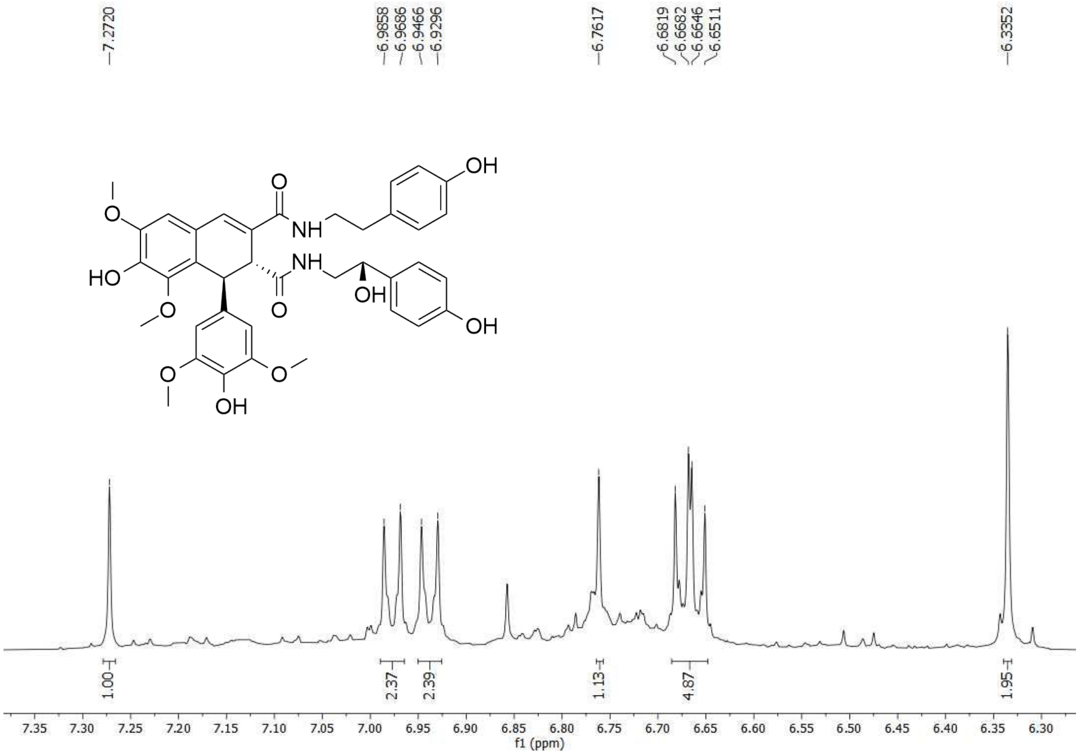

Figure S44. Amplification of the ^{1}H NMR spectrum of compound **5 (**500 MHz, MeOD).

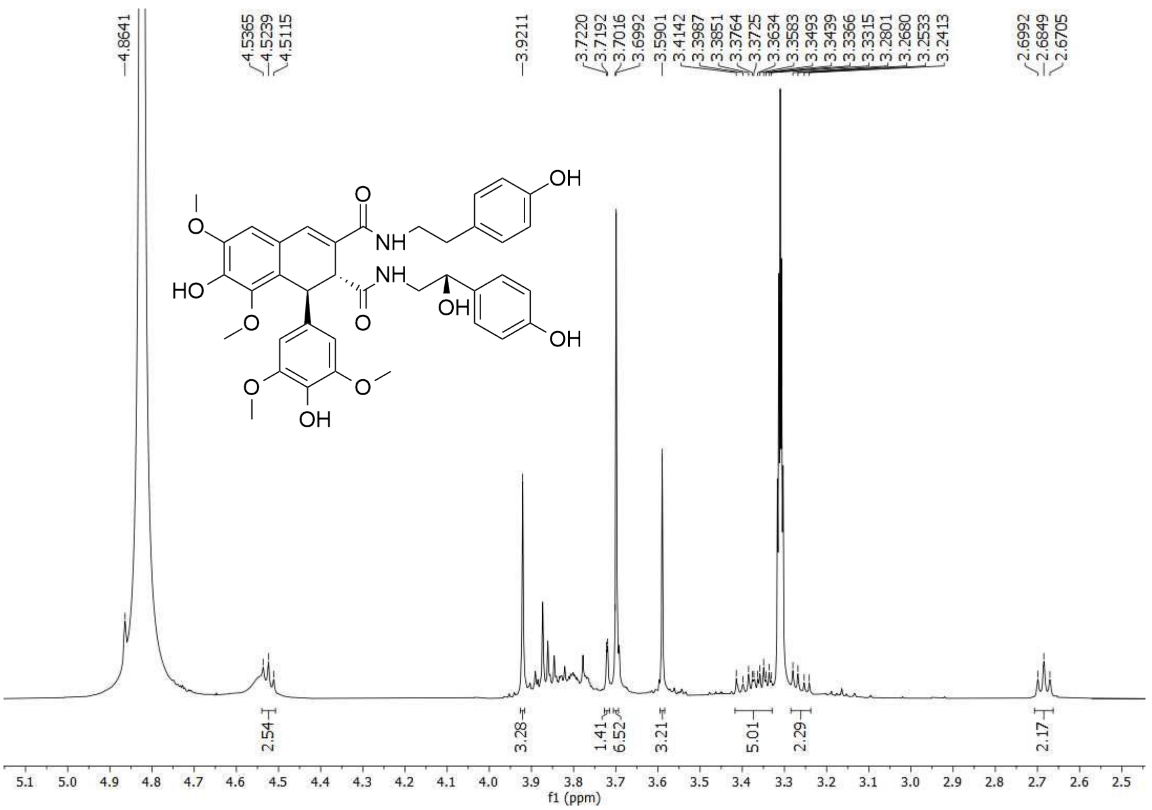

Figure S45. Amplification of the ^{1}H NMR spectrum of compound **5 (**500 MHz, MeOD).

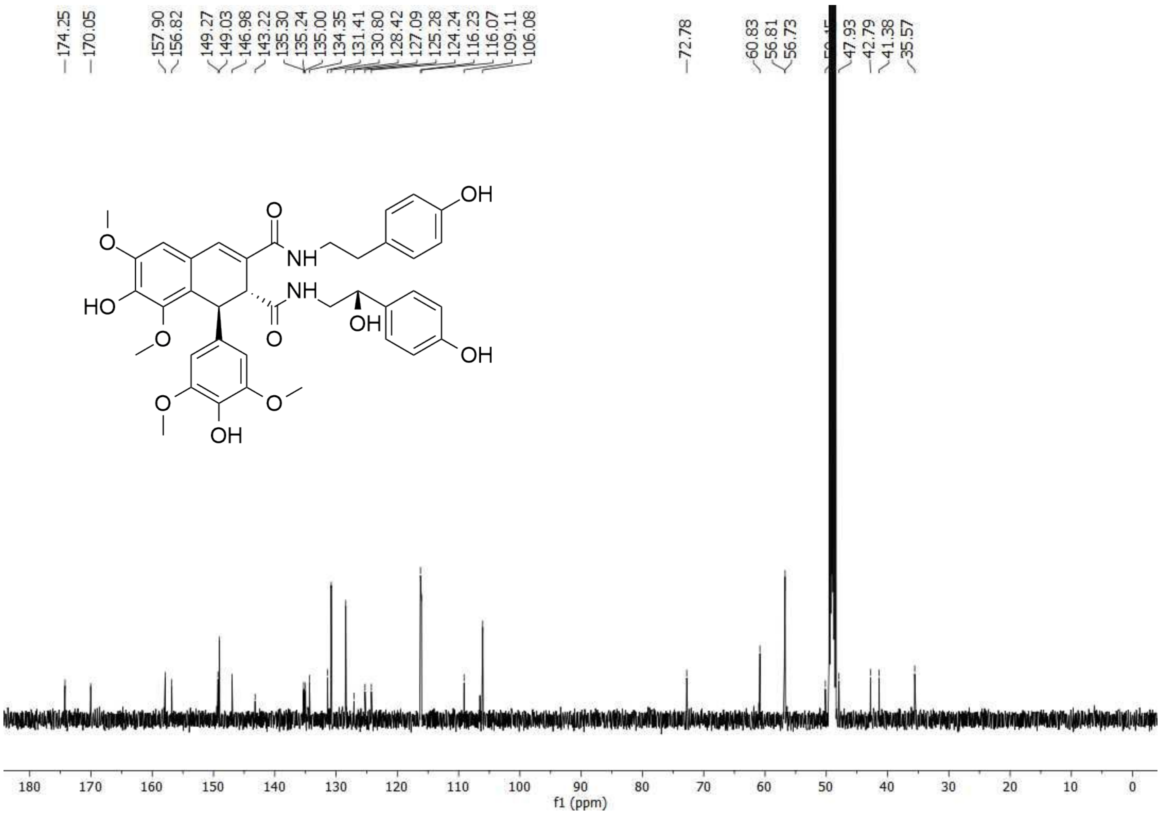

Figure S46. ^{13}C-BB NMR spectrum of compound **5** (125 MHz, MeOD).

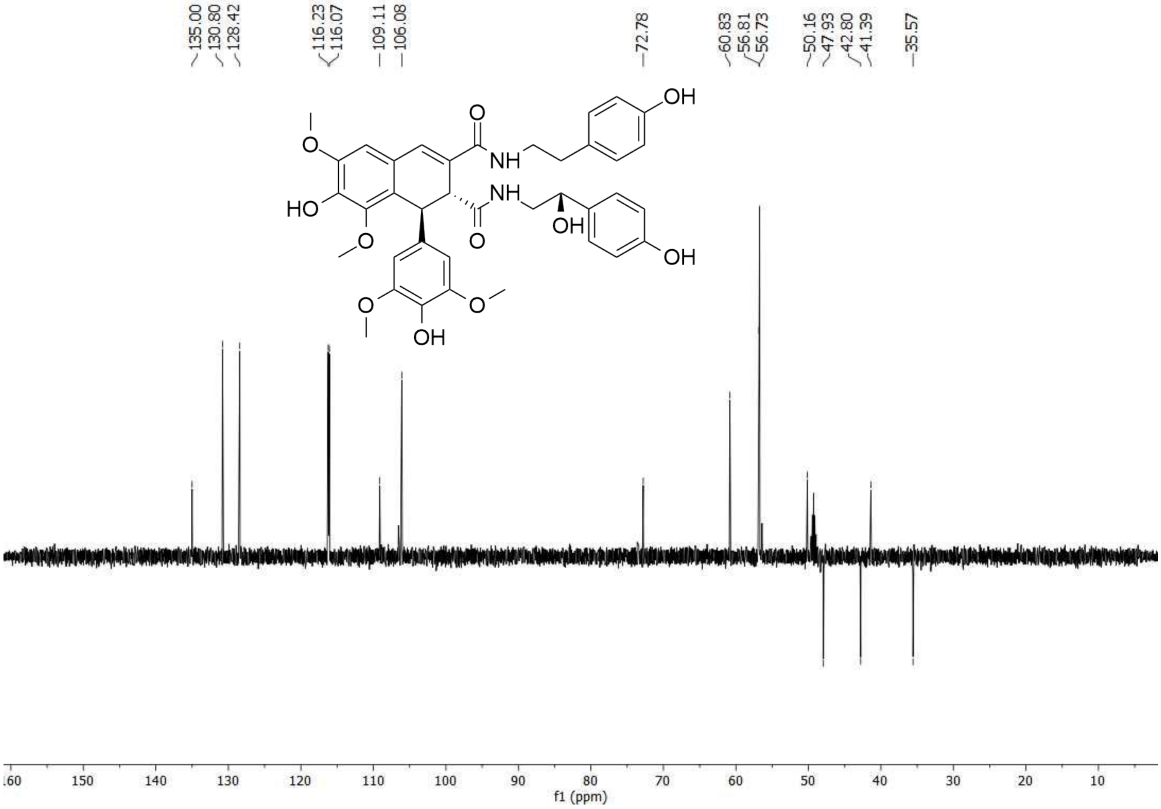

Figure S47. ^{13}C-DEPT135 NMR spectrum of compound **5** (125 MHz, MeOD).

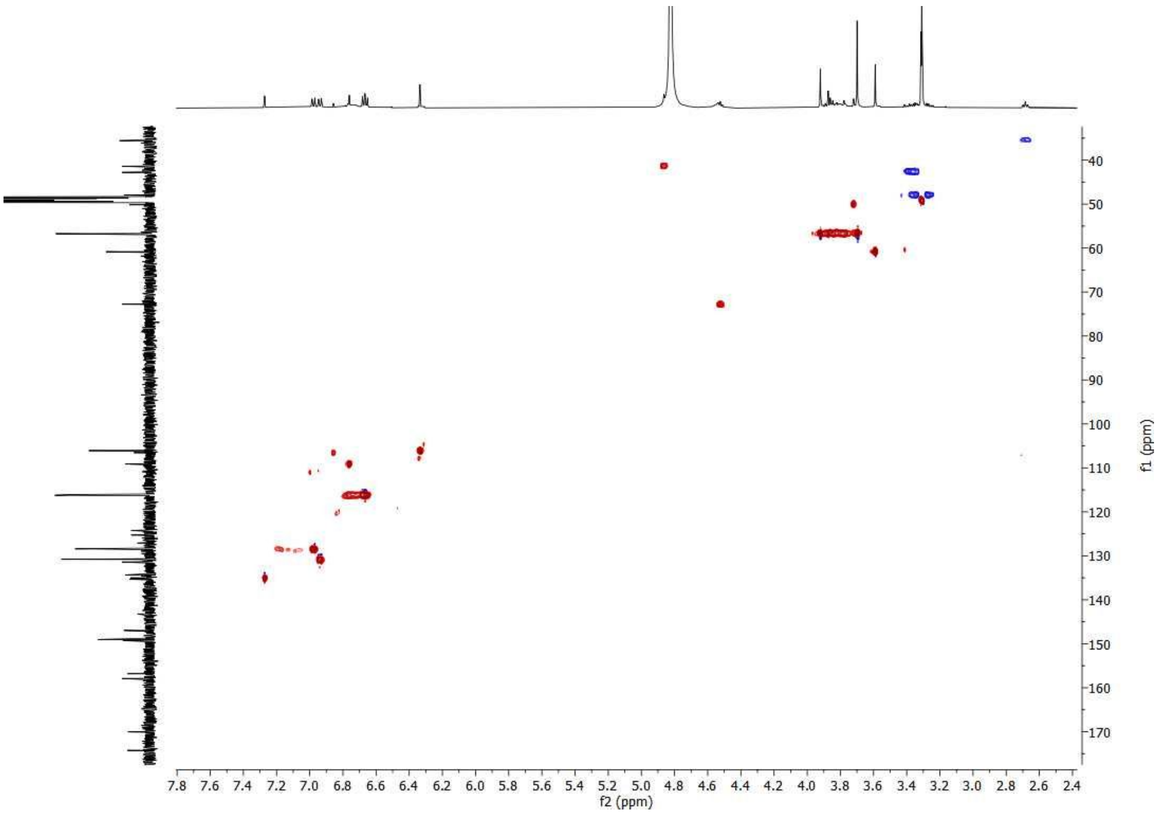

Figure S48. HSQC spectrum of compound **5** (500/125 MHz, MeOD).

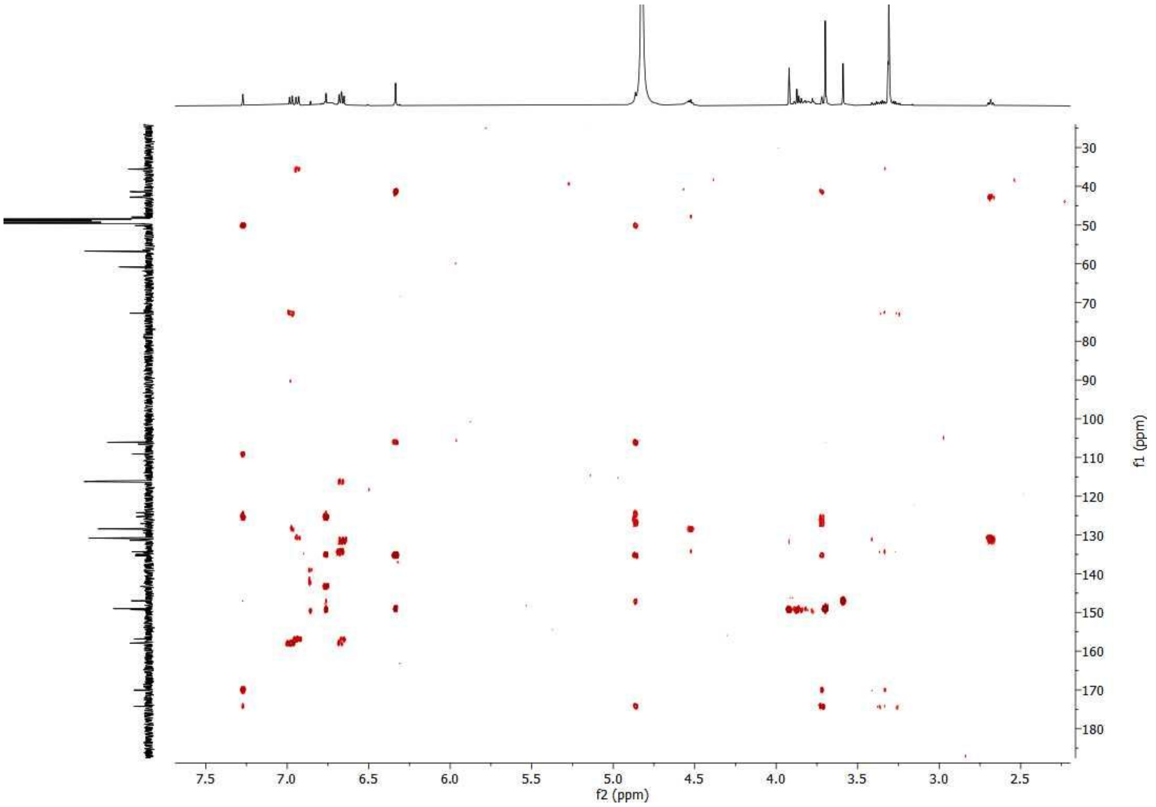

Figure S49. HMBC spectrum of compound **5 (**500/125 MHz, MeOD).

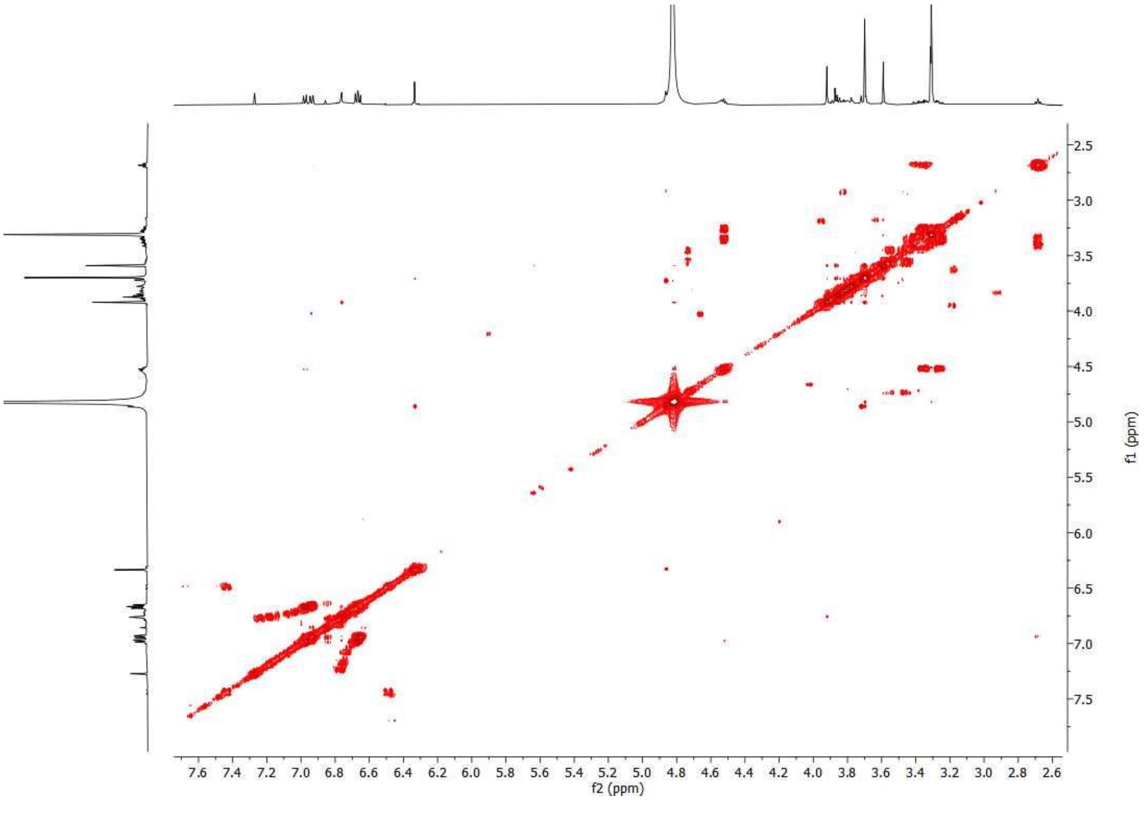

Figure S50. COSY spectrum of compound **5 (**500 MHz, MeOD).

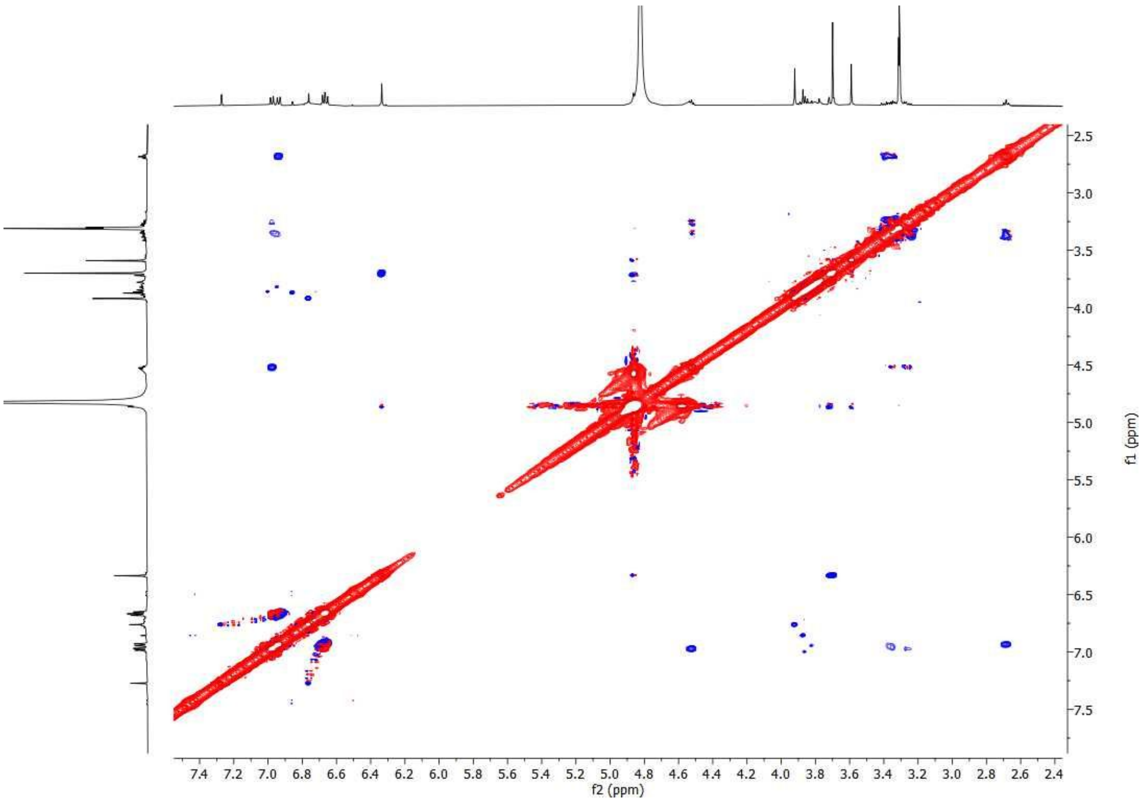

Figure S51. NOESY spectrum of compound **5 (**500 MHz, MeOD).

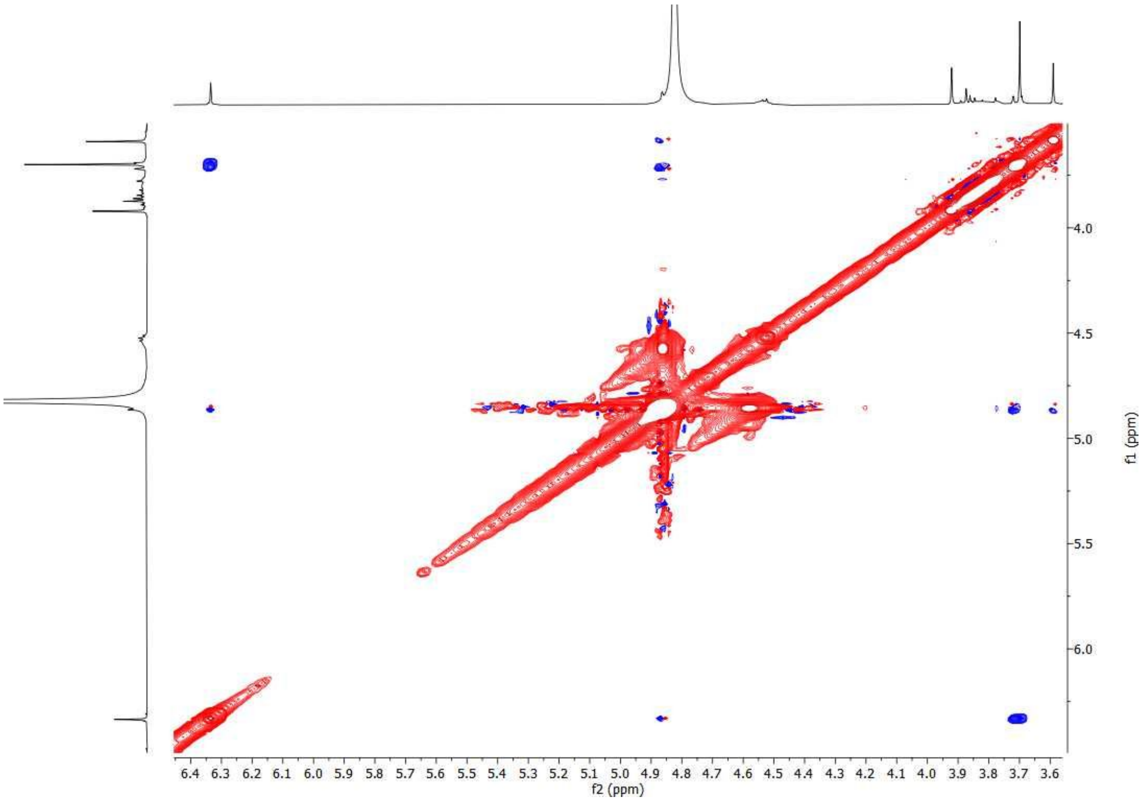

Figure S52. Amplification of the NOESY spectrum of compound **5 (**500 MHz, MeOD).

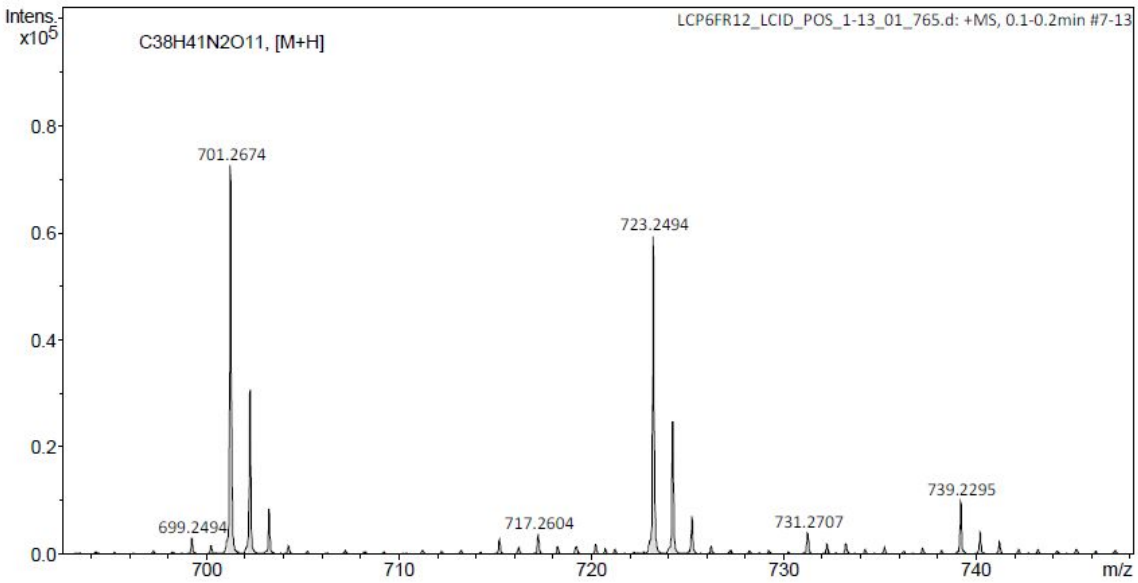

Figure S53. HRESIMS spectrum of compound **5** ([M + H]$^+$, positive mode).

6 Spectroscopic data for compound 6

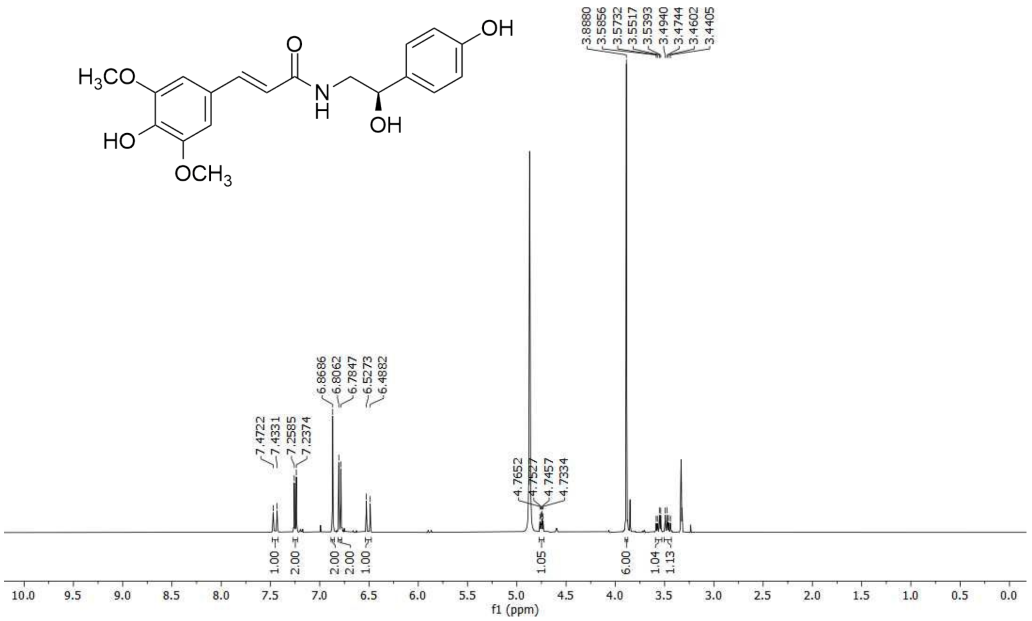

Figure S54. ^{1}H NMR spectrum of compound **6** (400 MHz, MeOD).

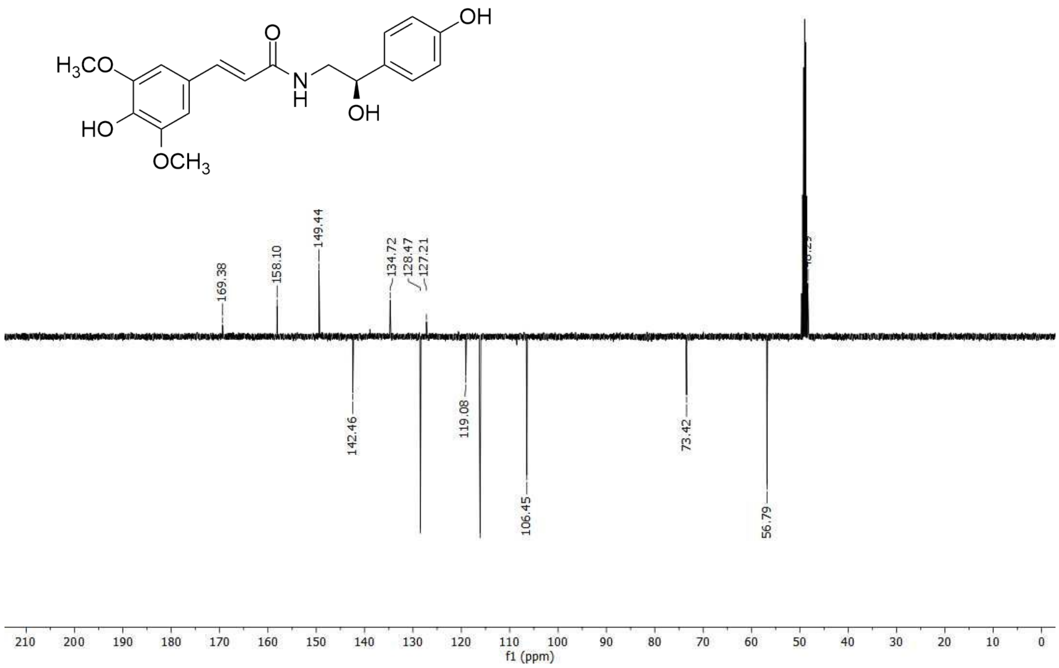

Figure S55. ^{13}C-APT NMR spectrum of compound **6** (100 MHz, MeOD).

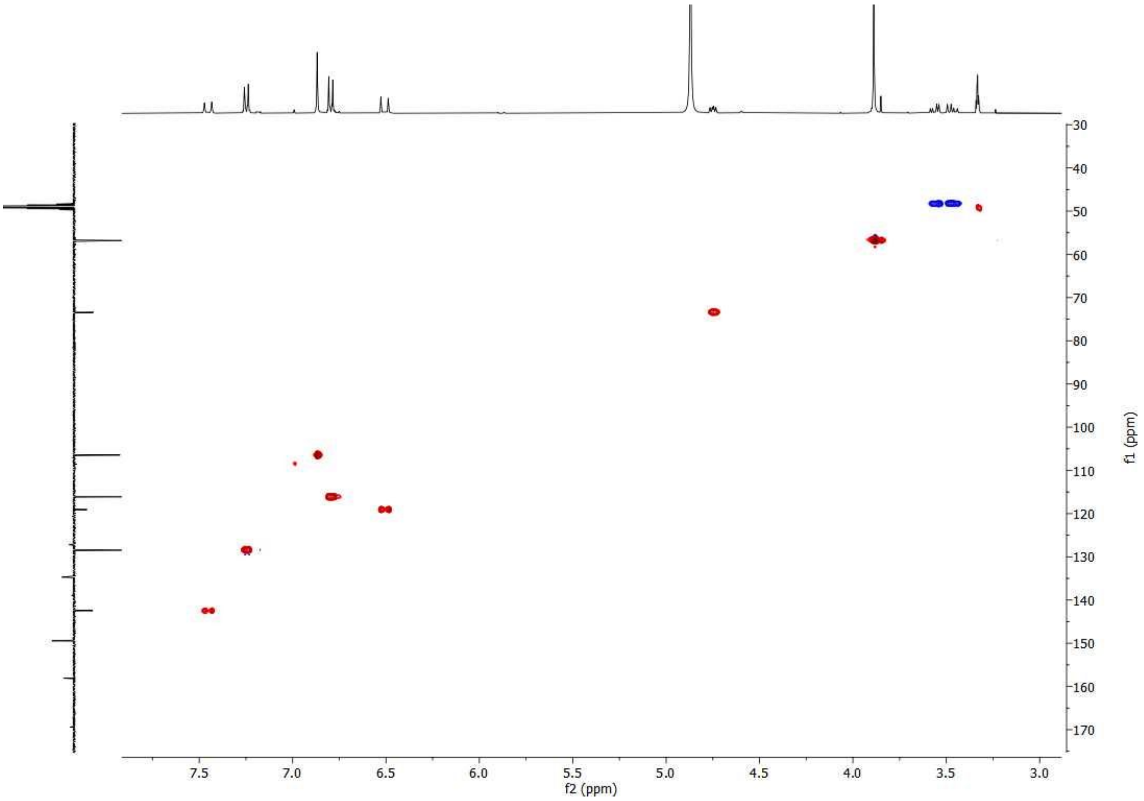

Figure S56. HSQC spectrum of compound **6** (400/100 MHz, MeOD).

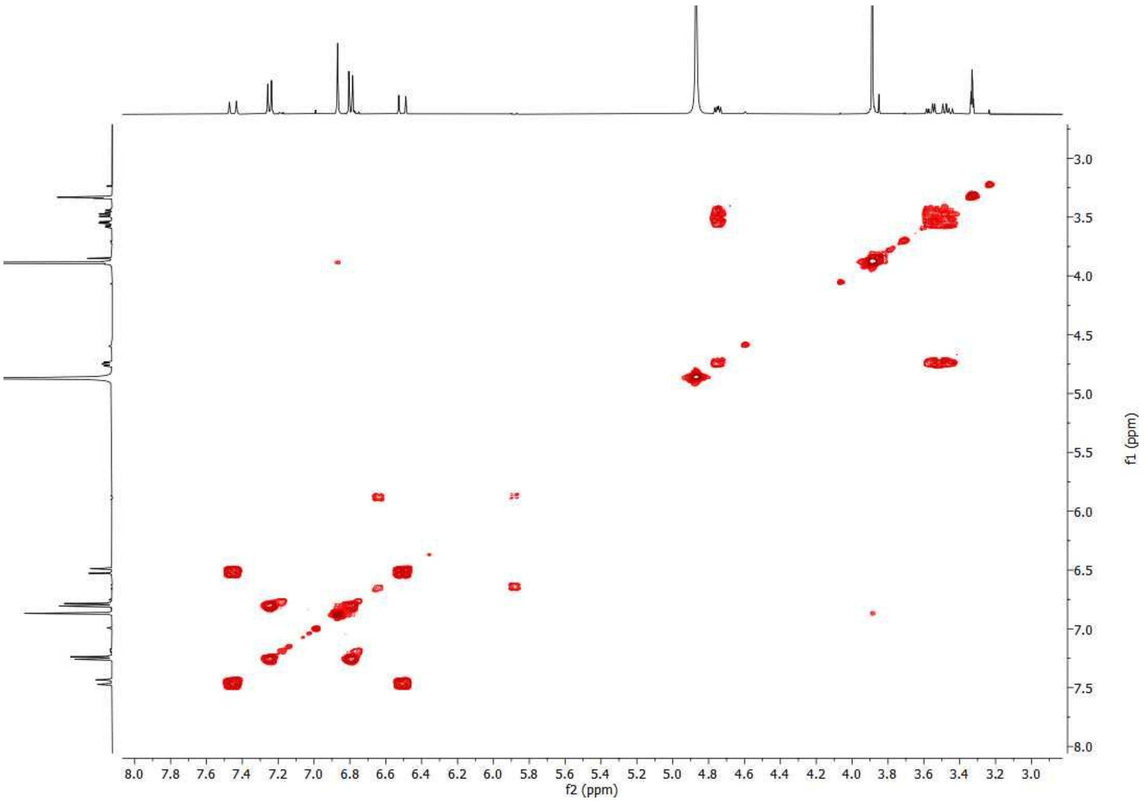

Figure S57. COSY spectrum of compound **6 (**400 MHz, MeOD).

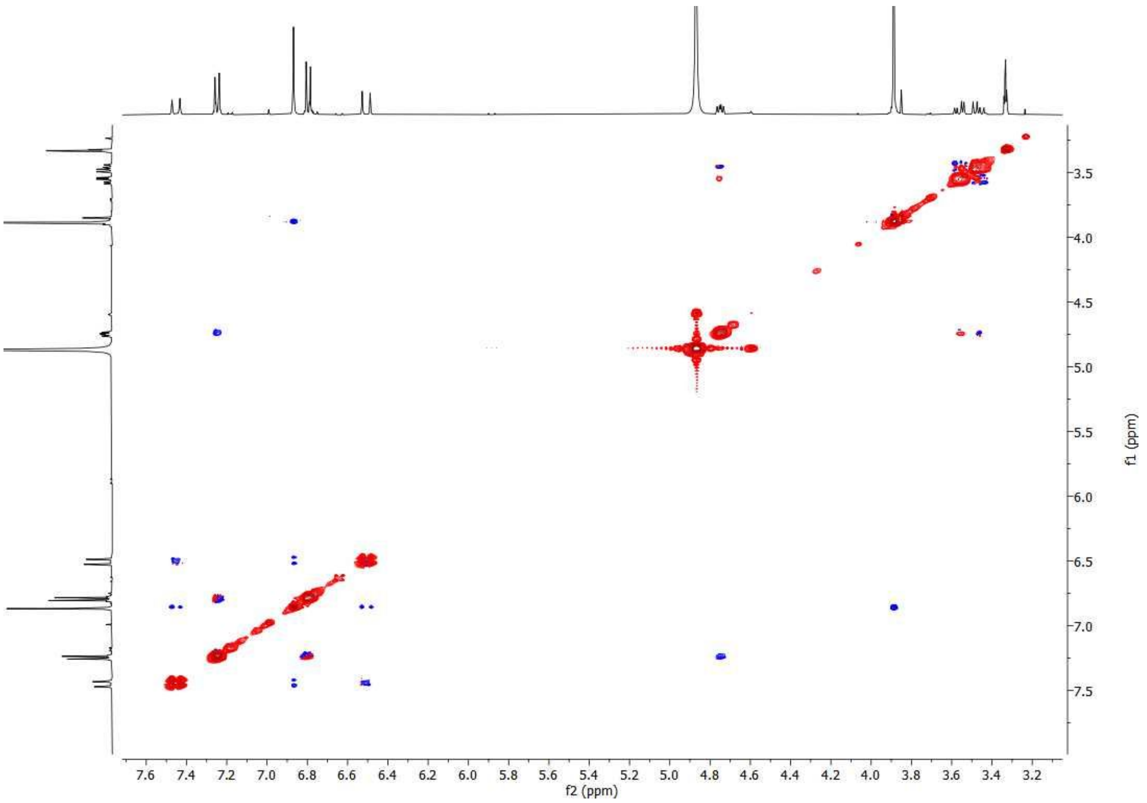

Figure S58. NOESY spectrum of compound **6 (**400 MHz, MeOD).

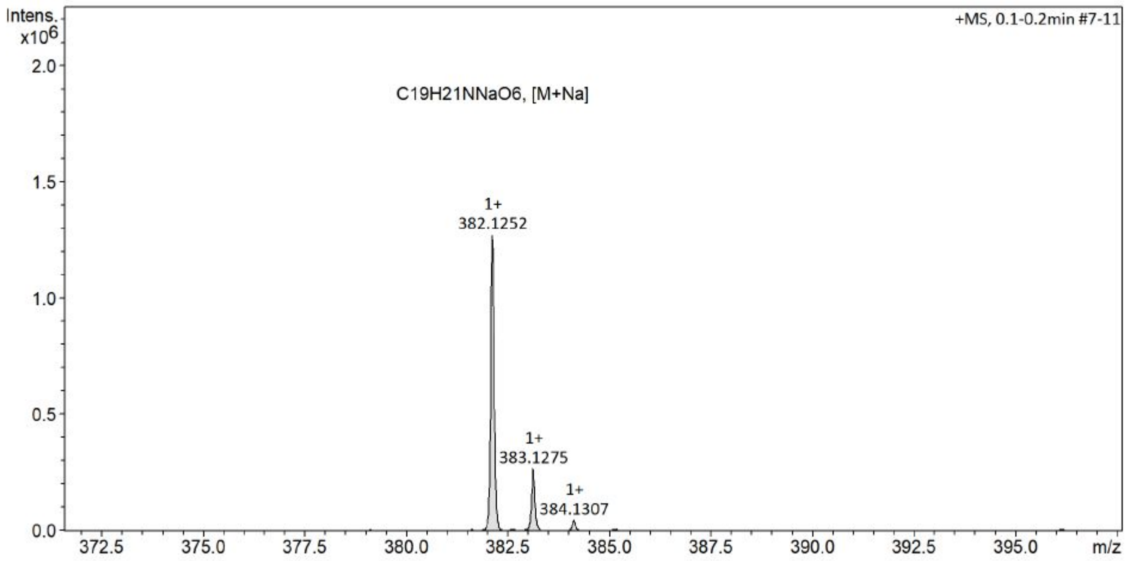

Figure S59. HRESIMS spectrum of compound **6** ([M + Na]$^+$, positive mode).

7 Spectroscopic data for compound 7

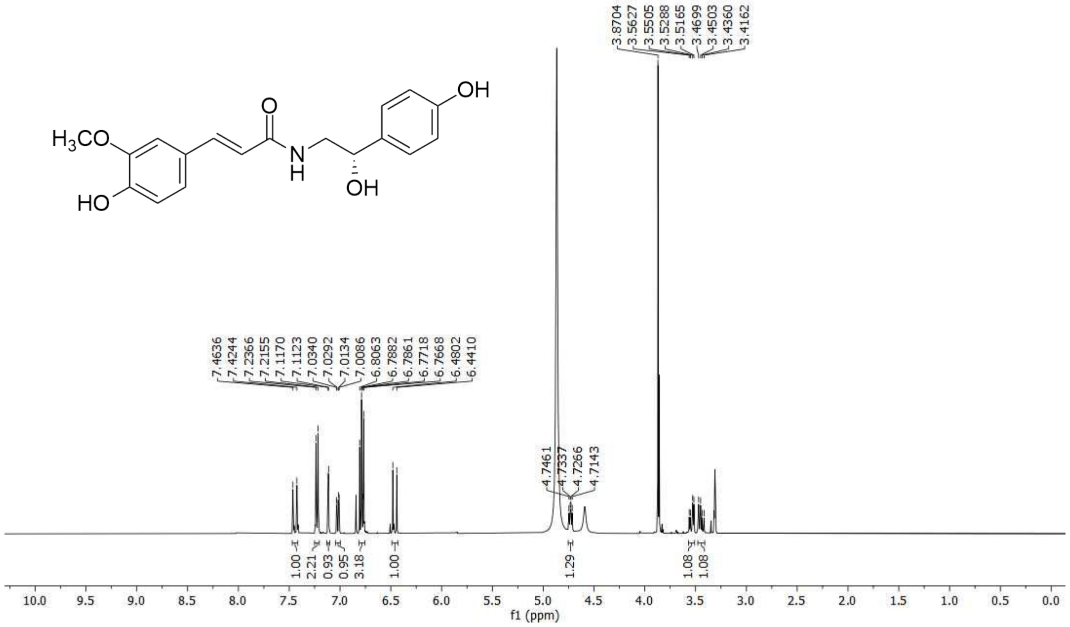

Figure S60. ^{1}H NMR spectrum of compound **7** (400 MHz, MeOD).

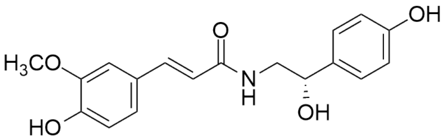

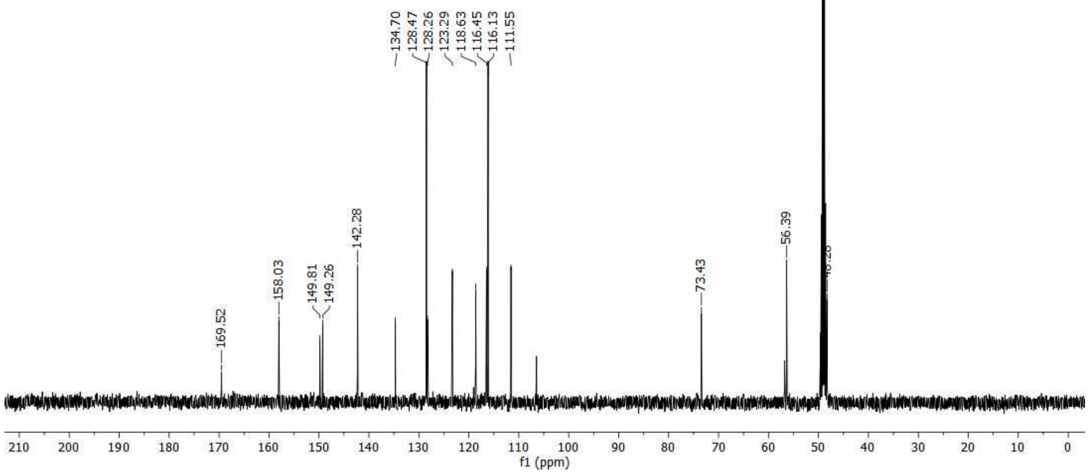

Figure S61. ^{13}C-BB NMR spectrum of compound **7** (100 MHz, MeOD).

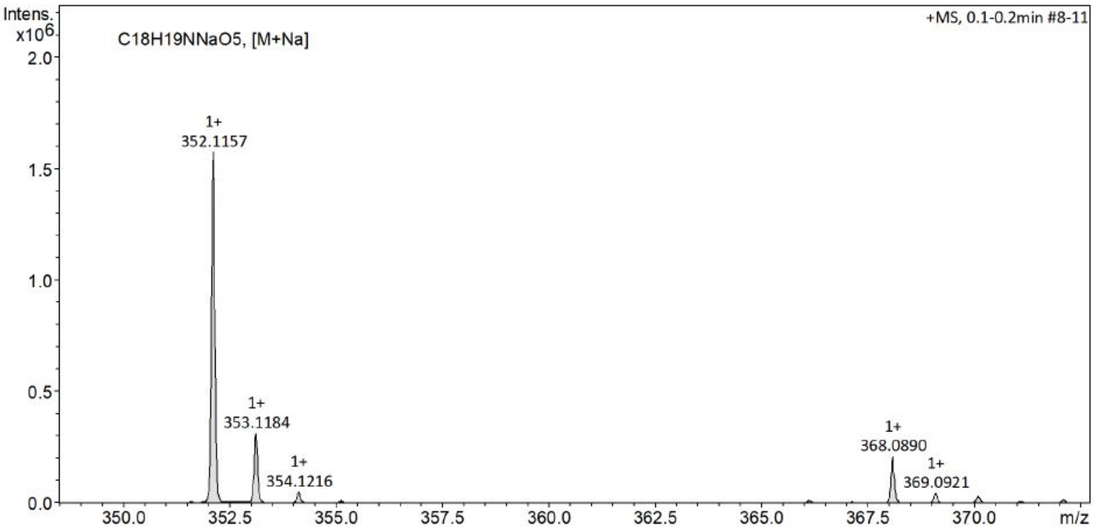

Figure S62. HRESIMS spectrum of compound **7** ($[M + Na]^+$, positive mode).

8 Spectroscopic data for compound 8

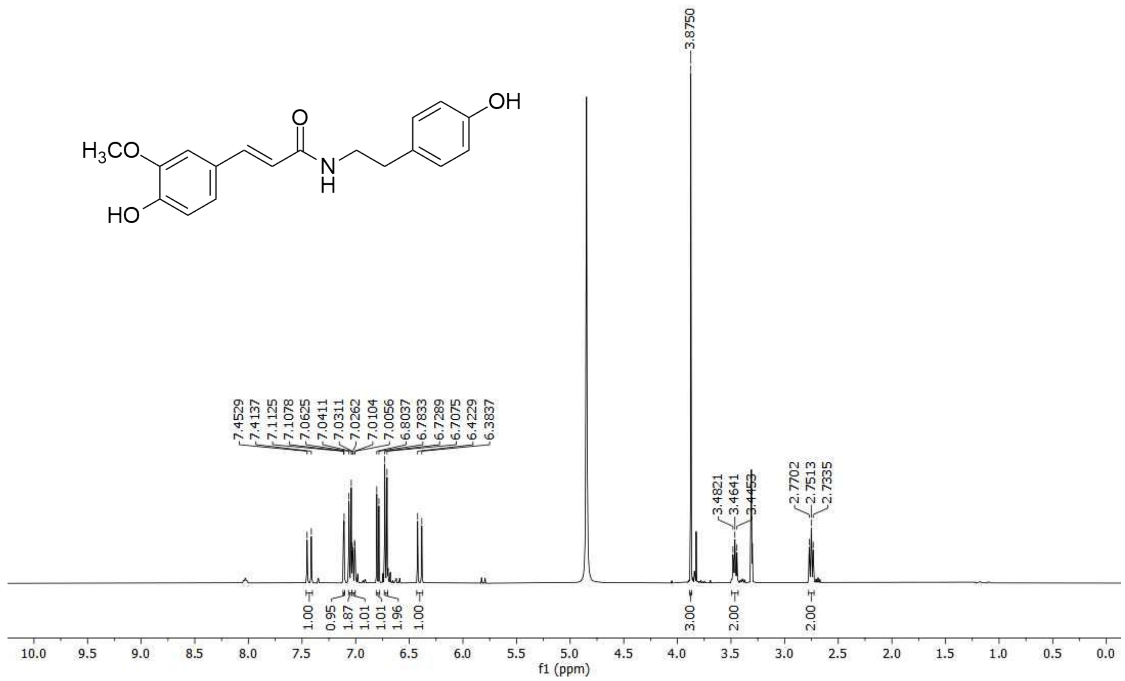

Figure S63. ^{1}H NMR spectrum of compound **8** (400 MHz, MeOD).

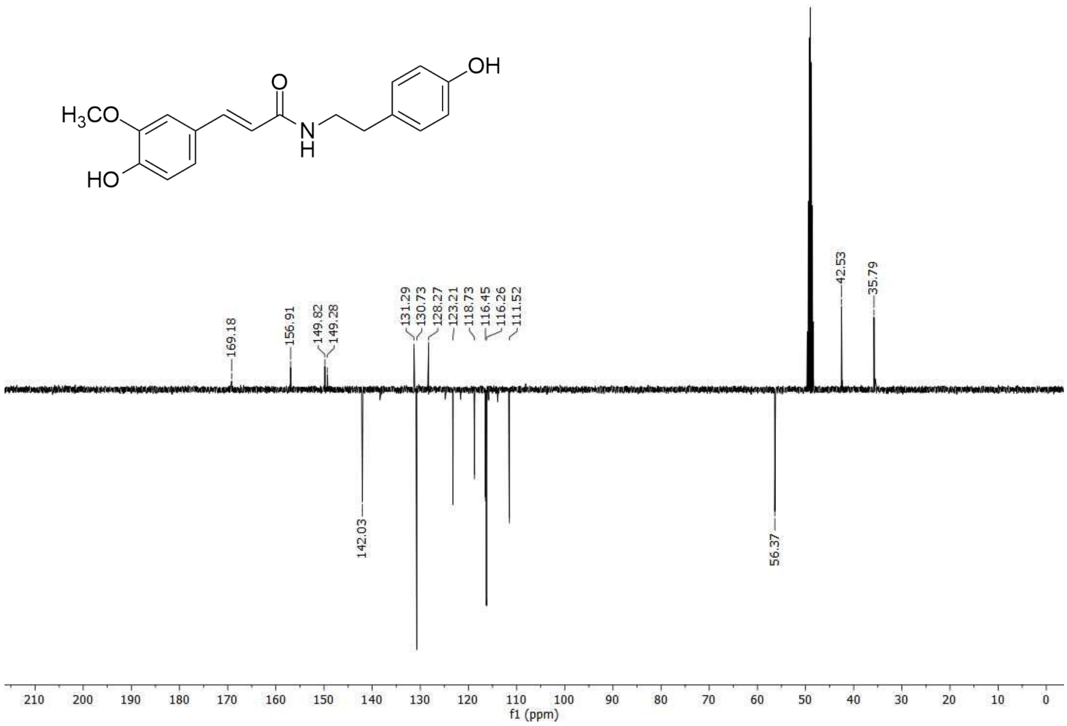

Figure S64. ^{13}C-APT NMR spectrum of compound **8** (100 MHz, MeOD).

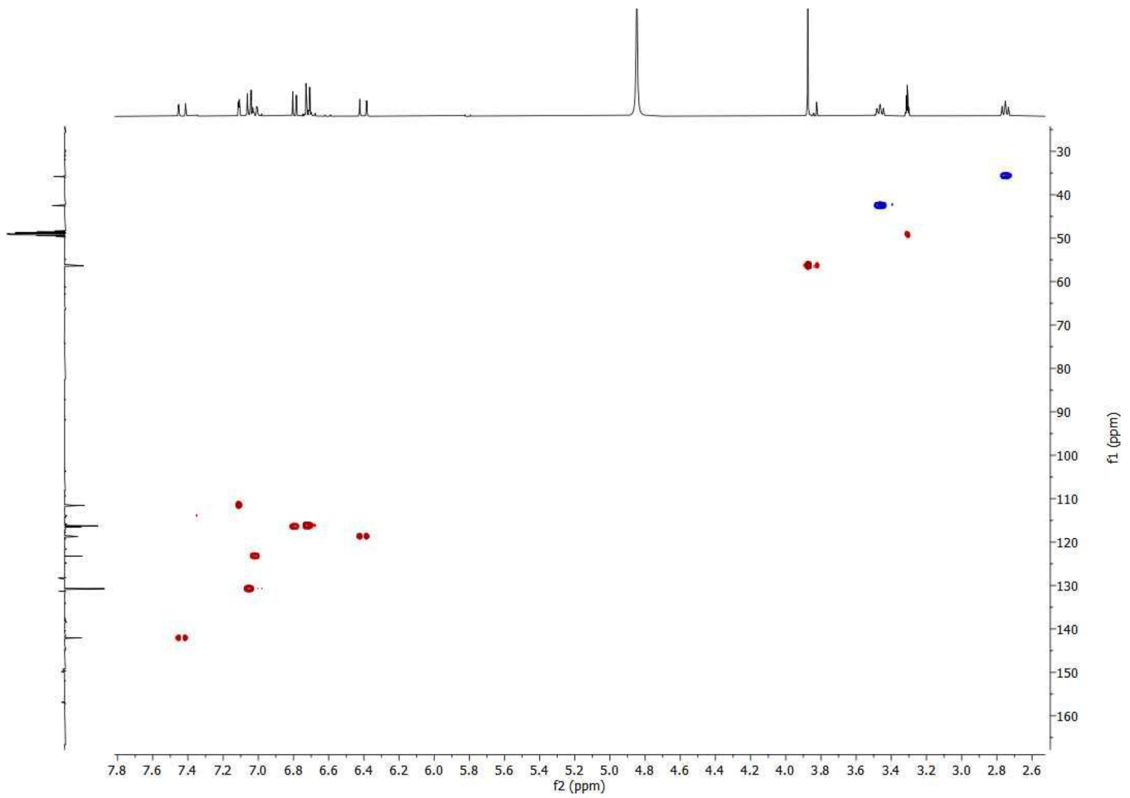

Figure S65. HSQC spectrum of compound **8 (**400/100 MHz, MeOD).

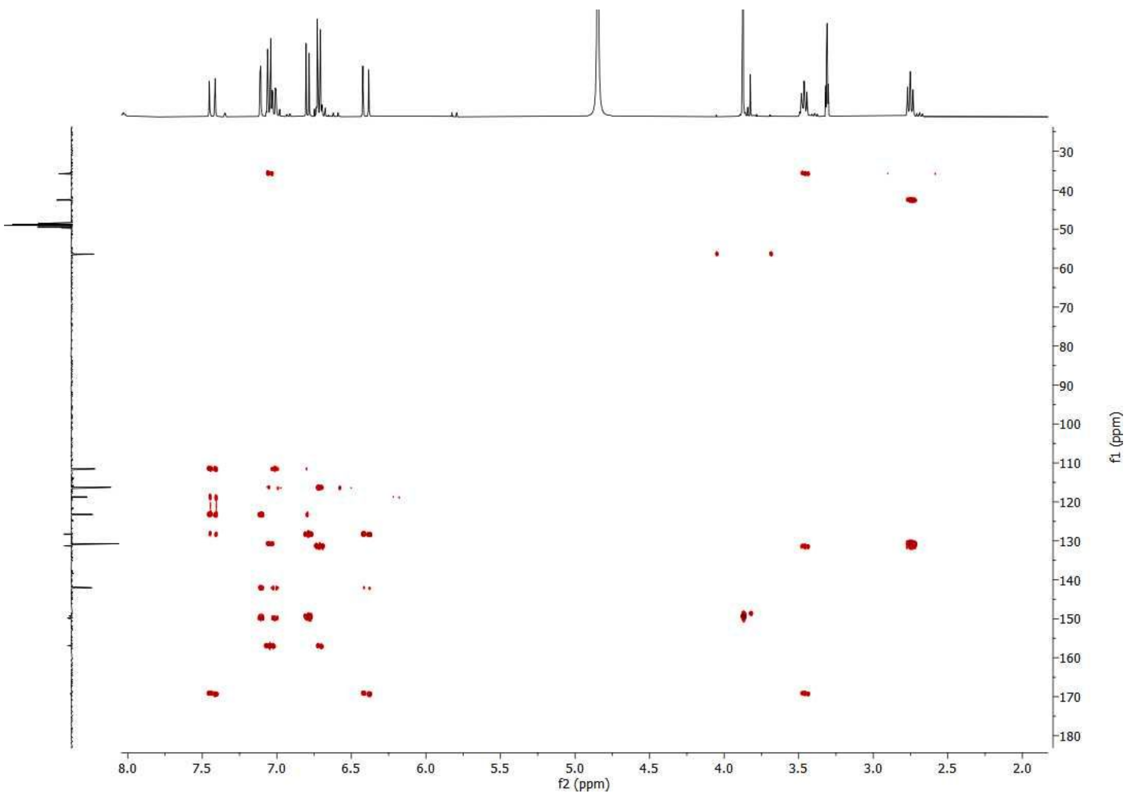

Figure S66. HMBC spectrum of compound **8 (**400/100 MHz, MeOD).

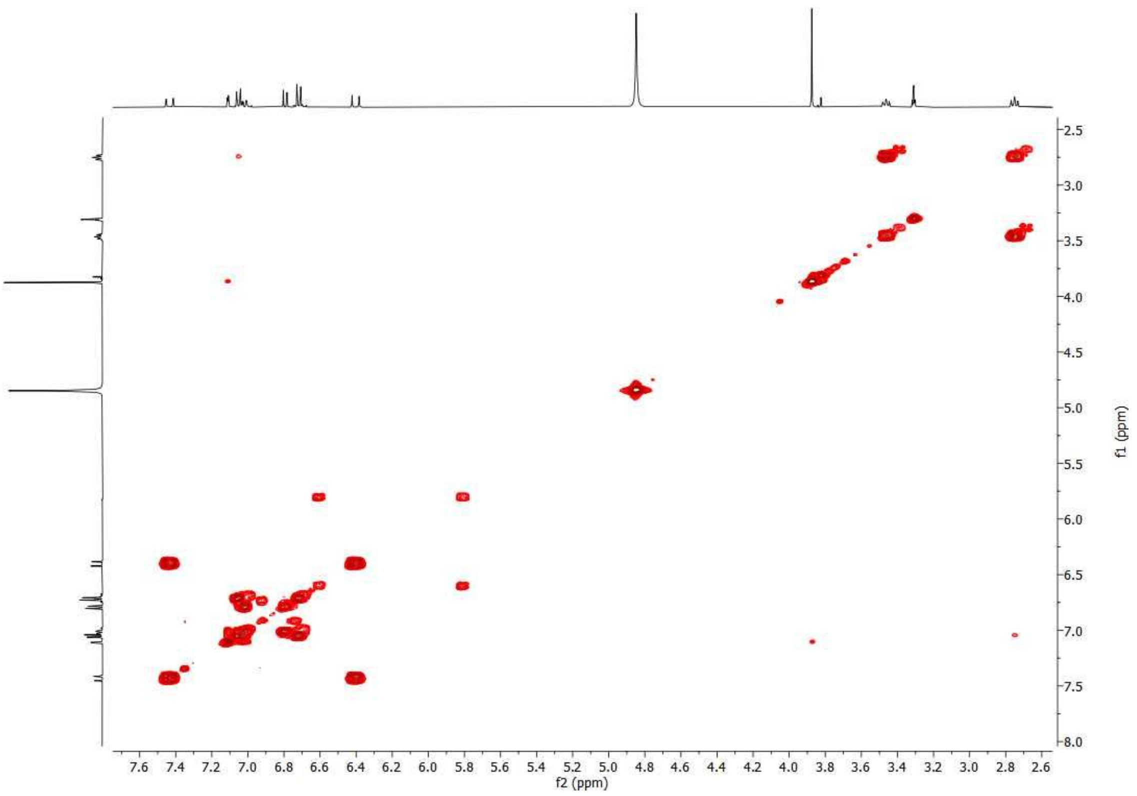

Figure S67. COSY spectrum of compound **8 (**400 MHz, MeOD).

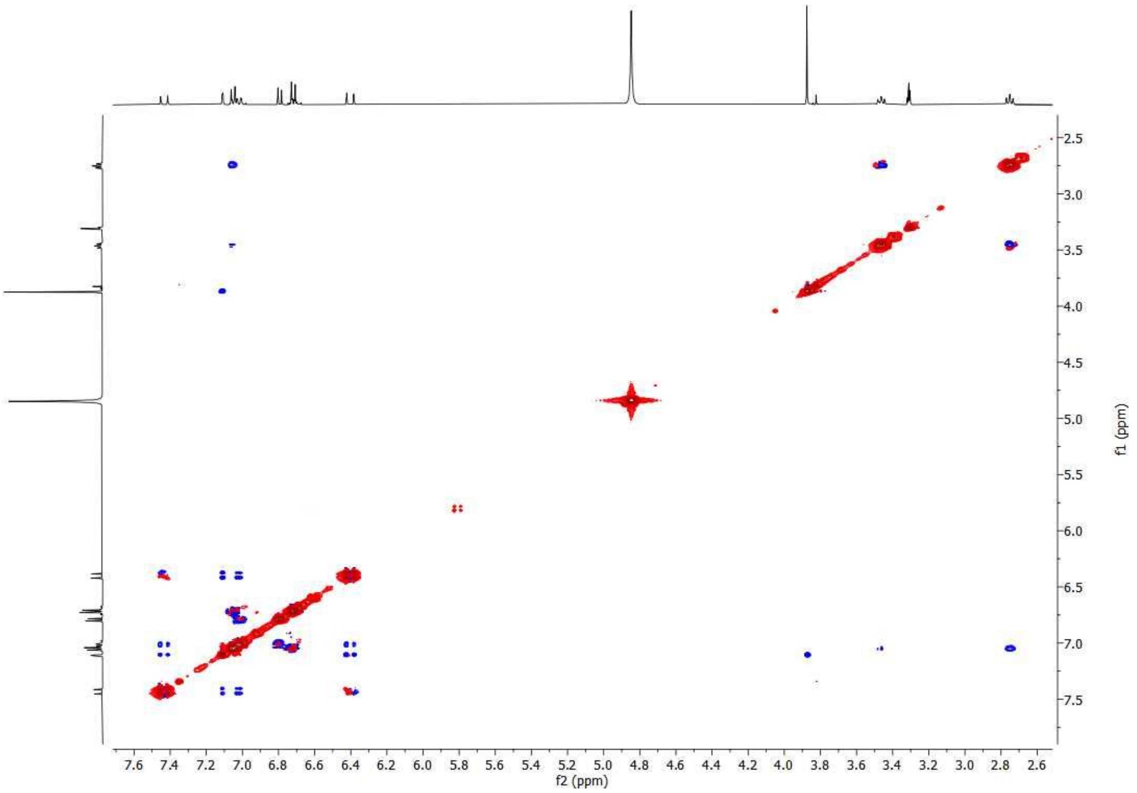

Figure S68. NOESY spectrum of compound **8 (**400 MHz, MeOD).

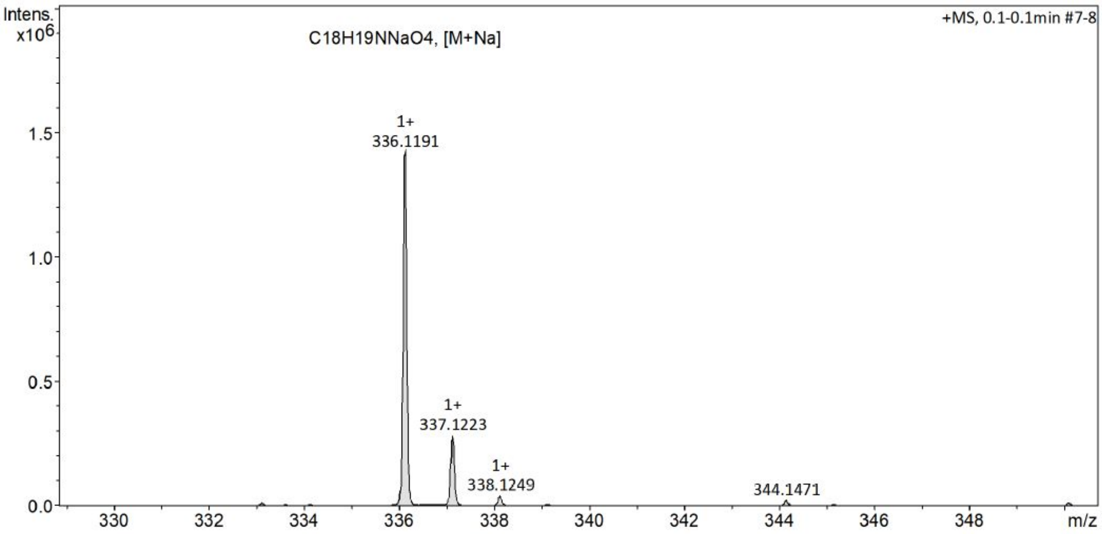

Figure S69. HRESIMS spectrum of compound **8** ([M + Na]$^{+}$, positive mode).

9 Spectroscopic data for compound 9

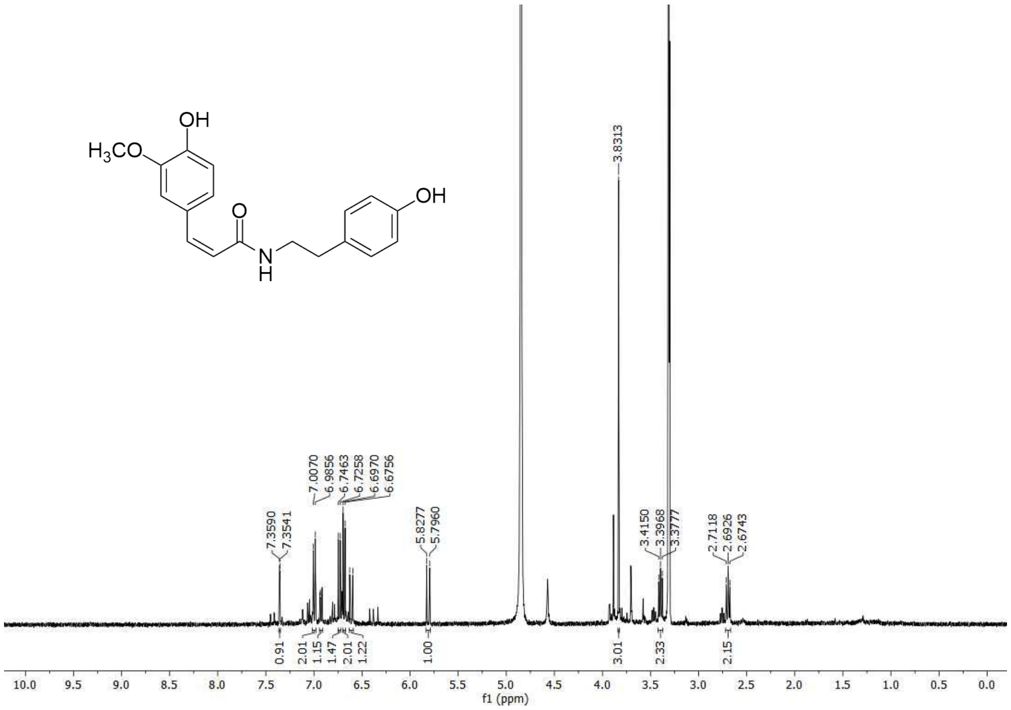

Figure S70. ^{1}H NMR spectrum of compound **9** (400 MHz, MeOD).

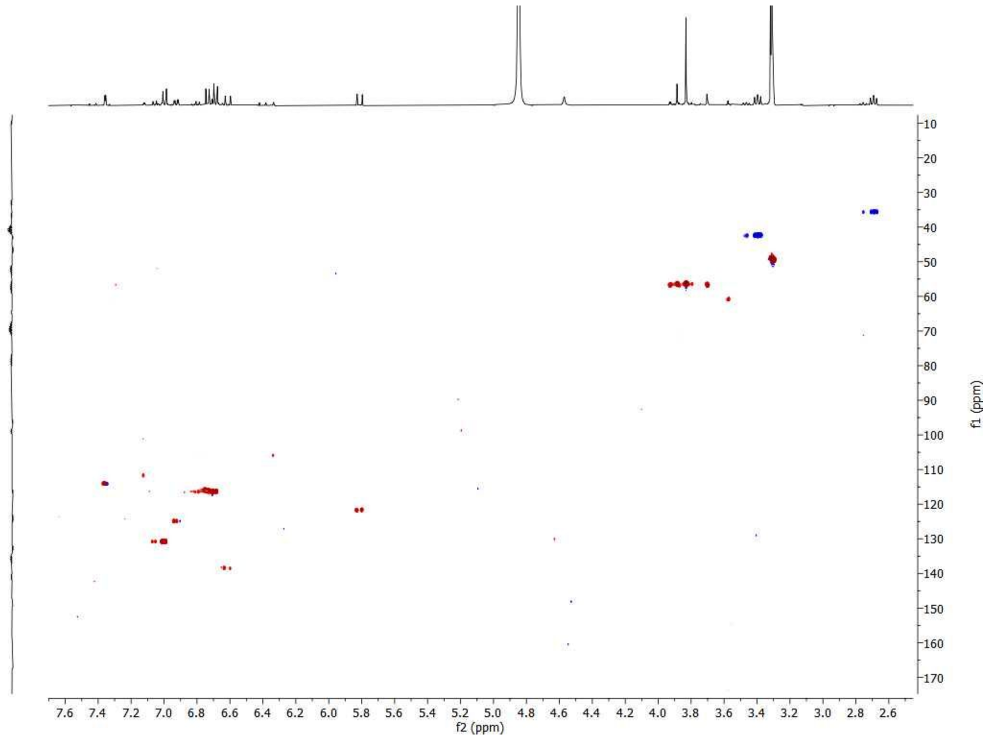

Figure S71. HSQC spectrum of compound **9** (400/100 MHz, MeOD).

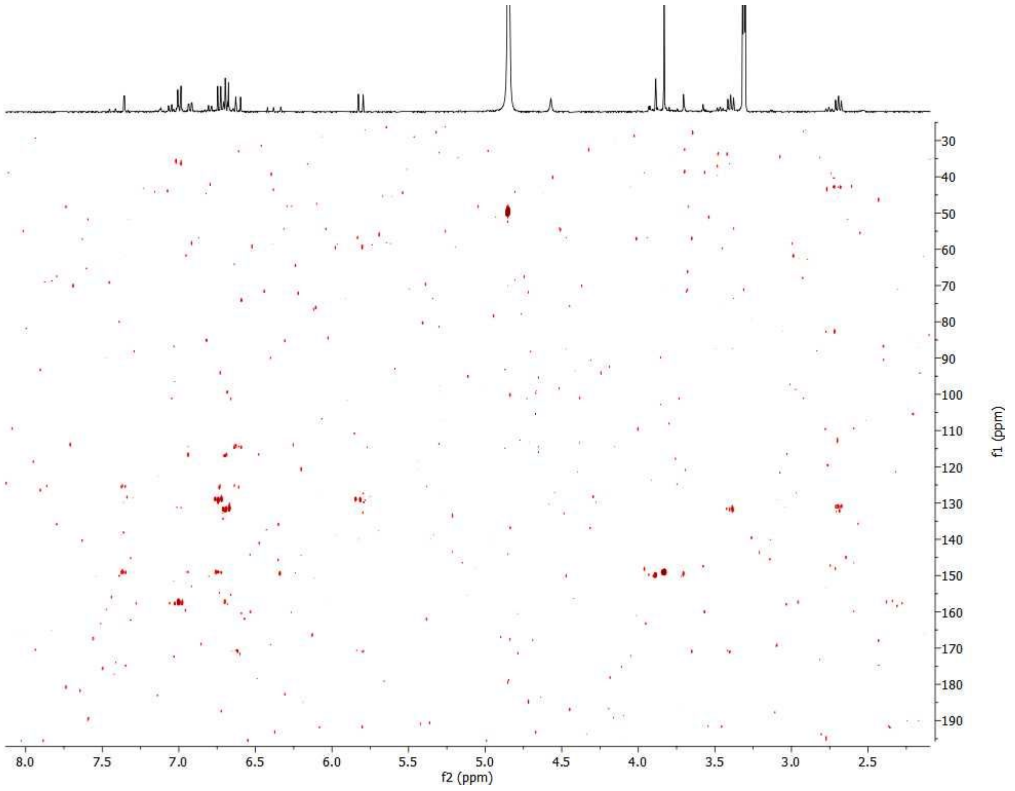

Figure S72. HMBC spectrum of compound **9** (400/100 MHz, MeOD).

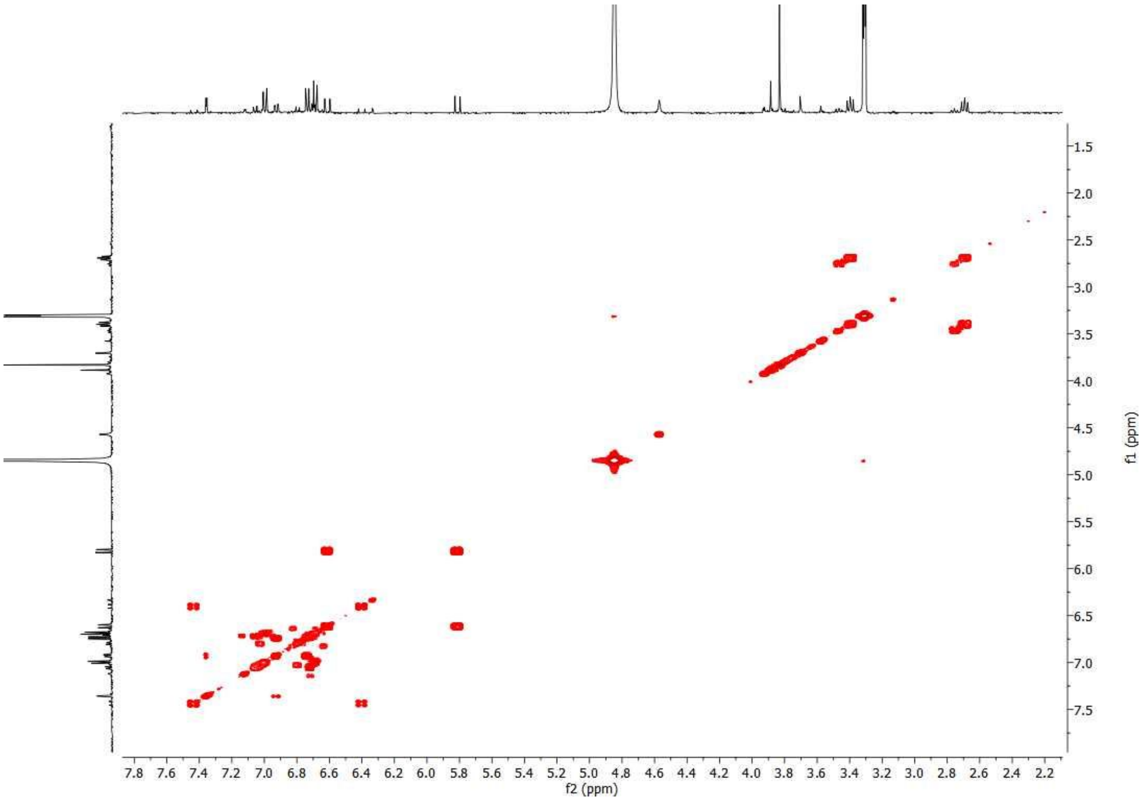

Figure S73. COSY spectrum of compound **9** (400 MHz, MeOD).

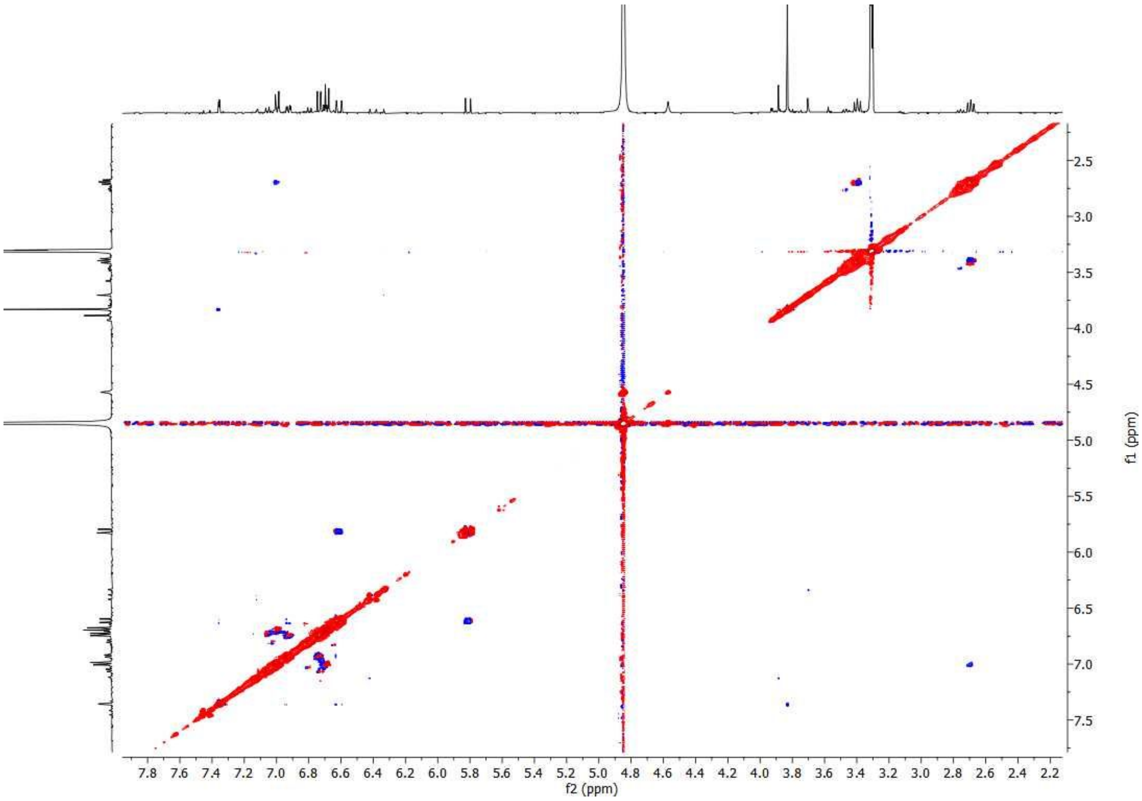

Figure S74. NOESY spectrum of compound **9** (400 MHz, MeOD).

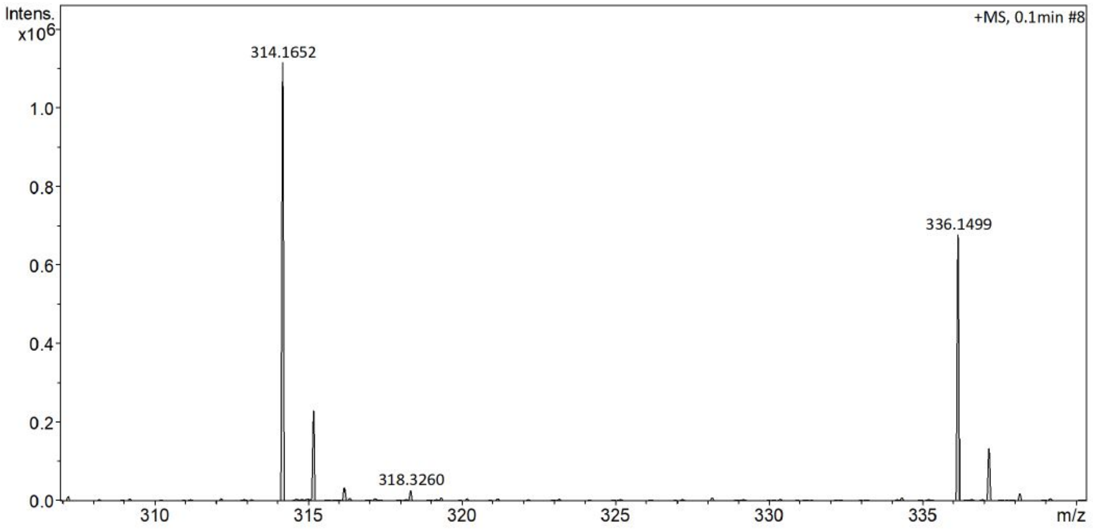

Figure S75. HRESIMS spectrum of compound **9** ([M + H]$^+$, positive mode).

10 Spectroscopic data for compound 10

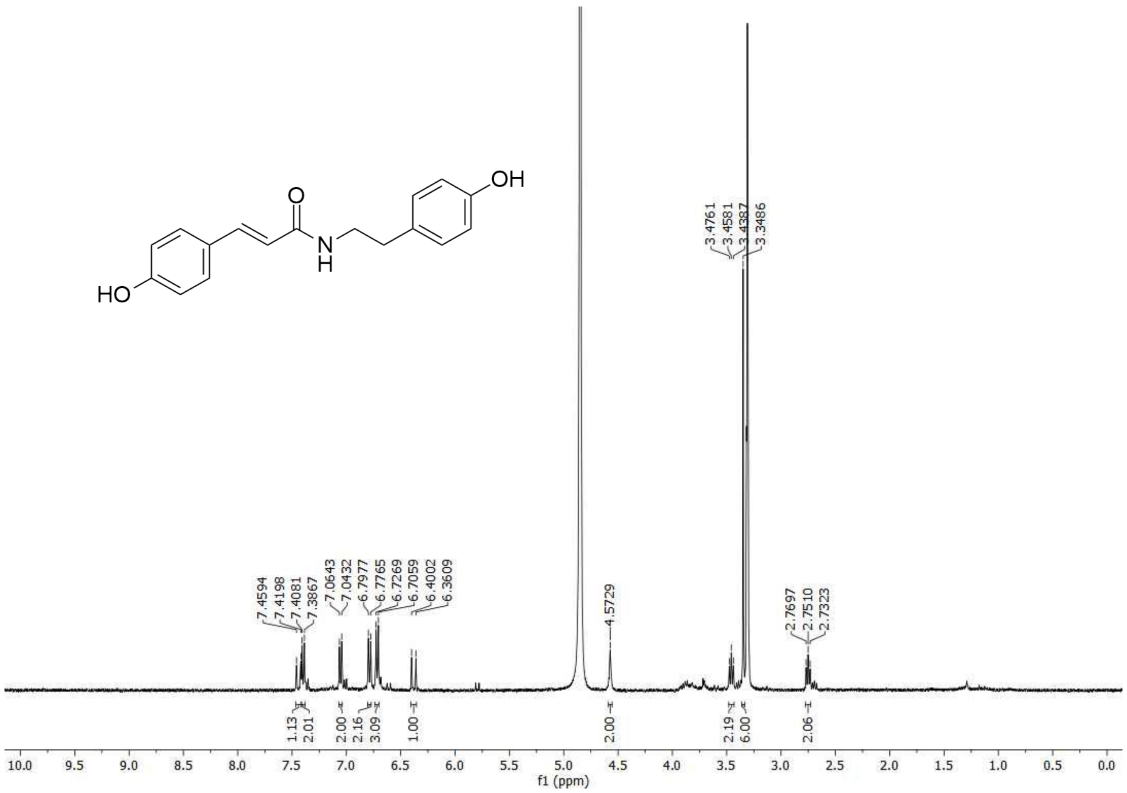

Figure S78. ^{1}H NMR spectrum of compound **10** (400 MHz, MeOD).

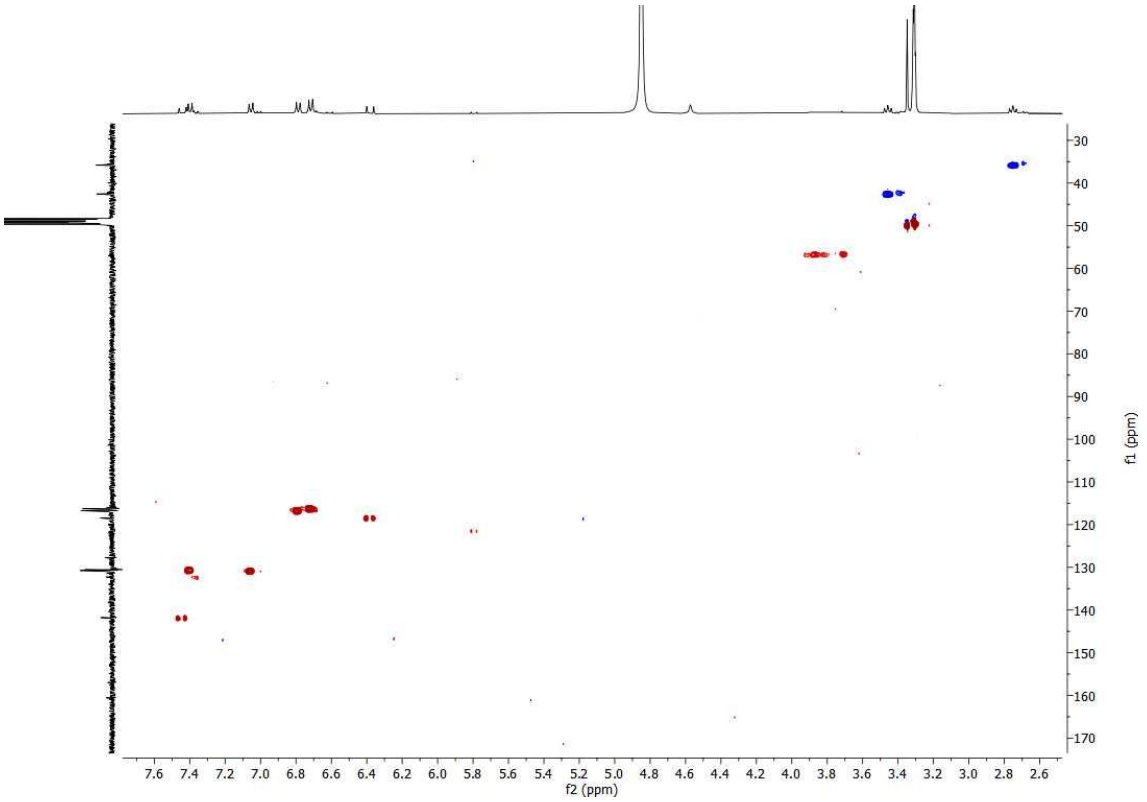

Figure S76. HSQC spectrum of compound **10 (**400/100 MHz, MeOD).

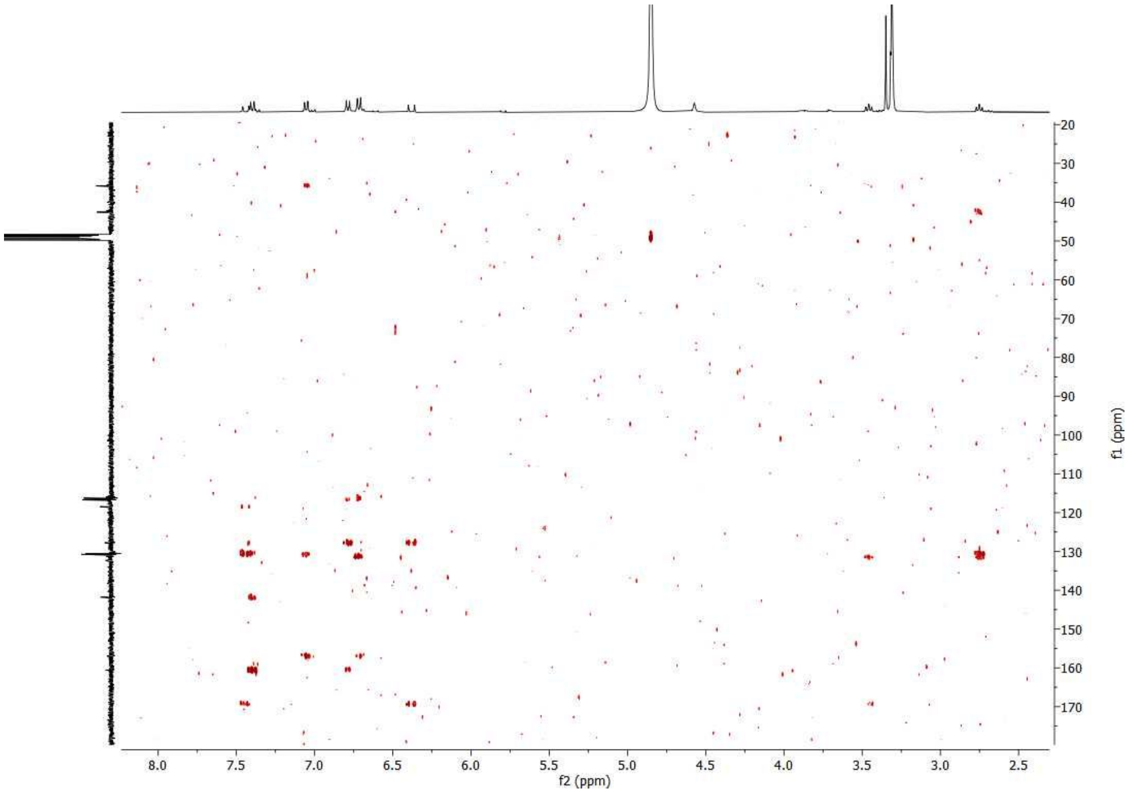

Figure S77. HMBC spectrum of compound **10 (**400/100 MHz, MeOD).

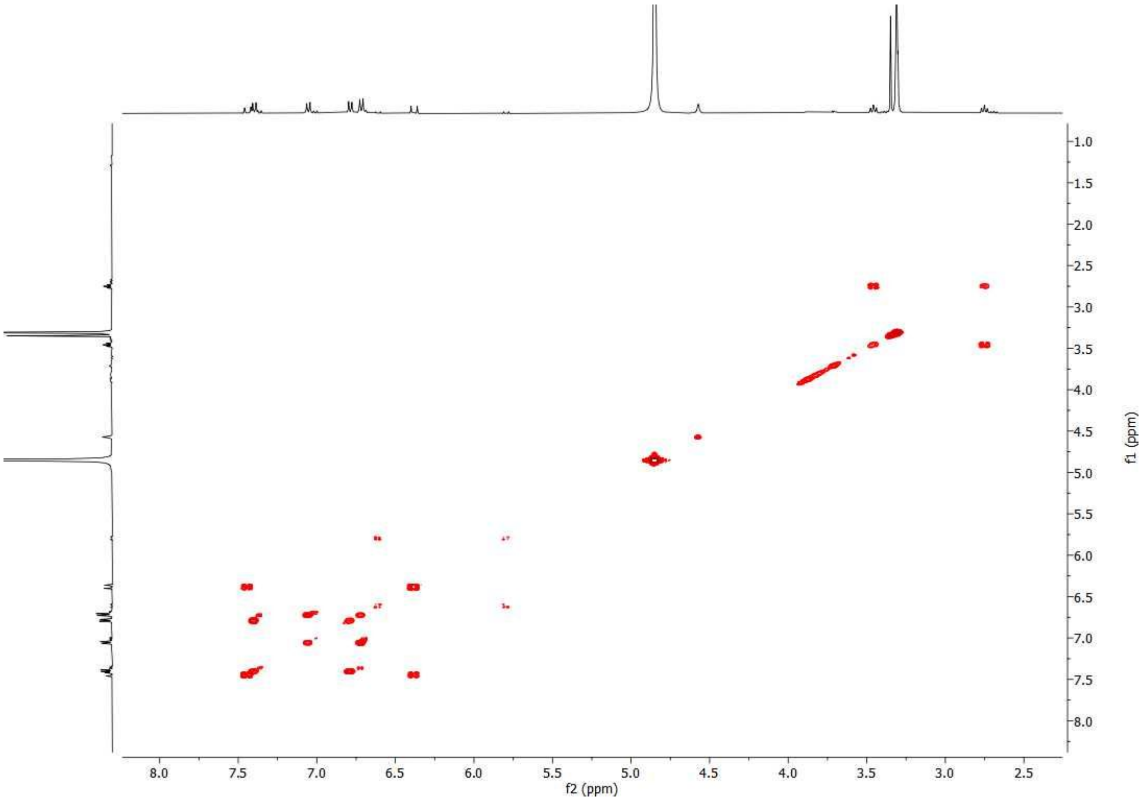

Figure S78. COSY spectrum of compound **10 (**400 MHz, MeOD).

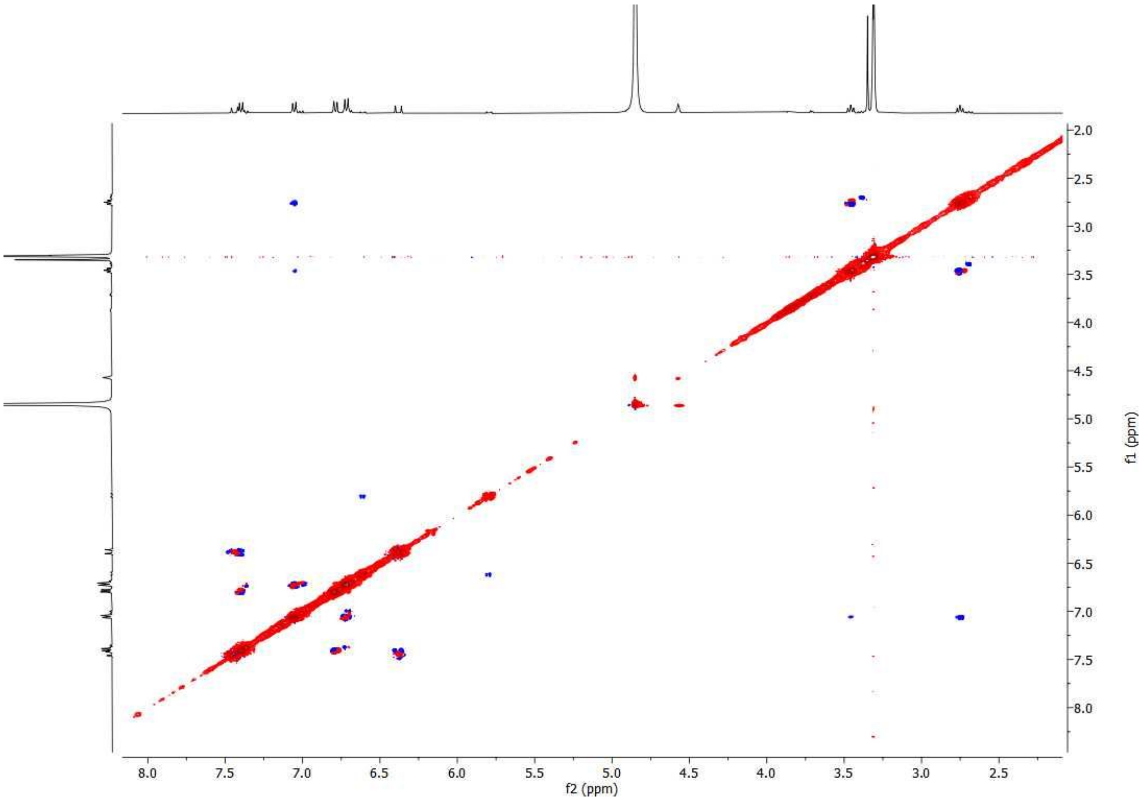

Figure S79. NOESY spectrum of compound **10 (**400 MHz, MeOD).

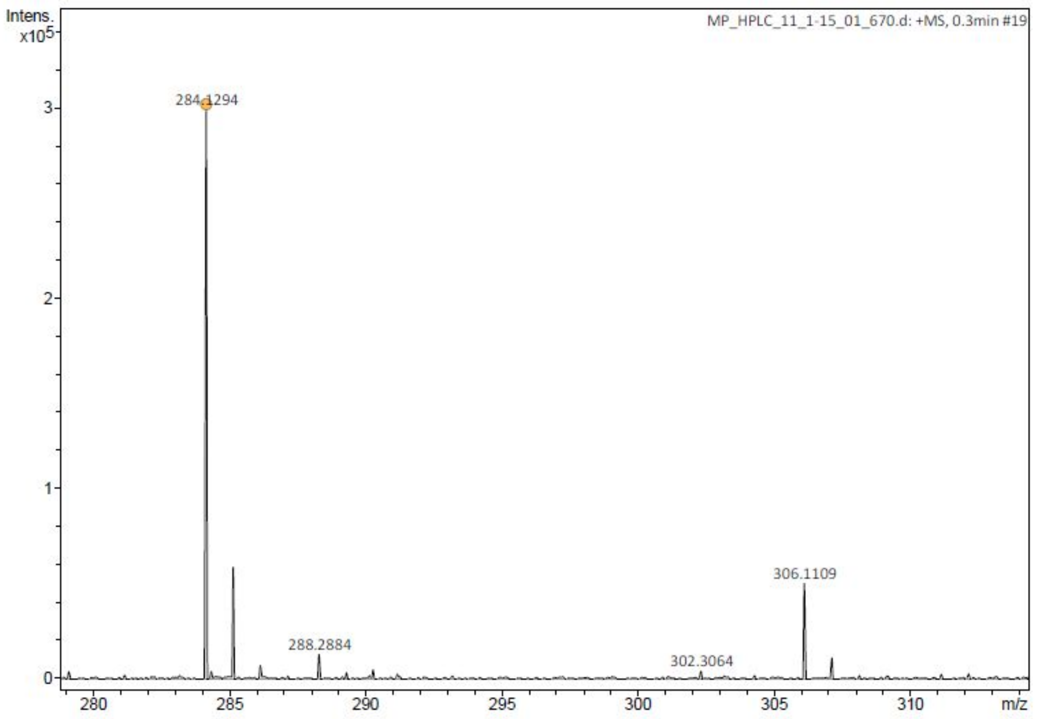

Figure S80. HRESIMS spectrum of compound **10** ([M + H]$^+$, positive mode).

11 Spectroscopic data for compounds 11,12

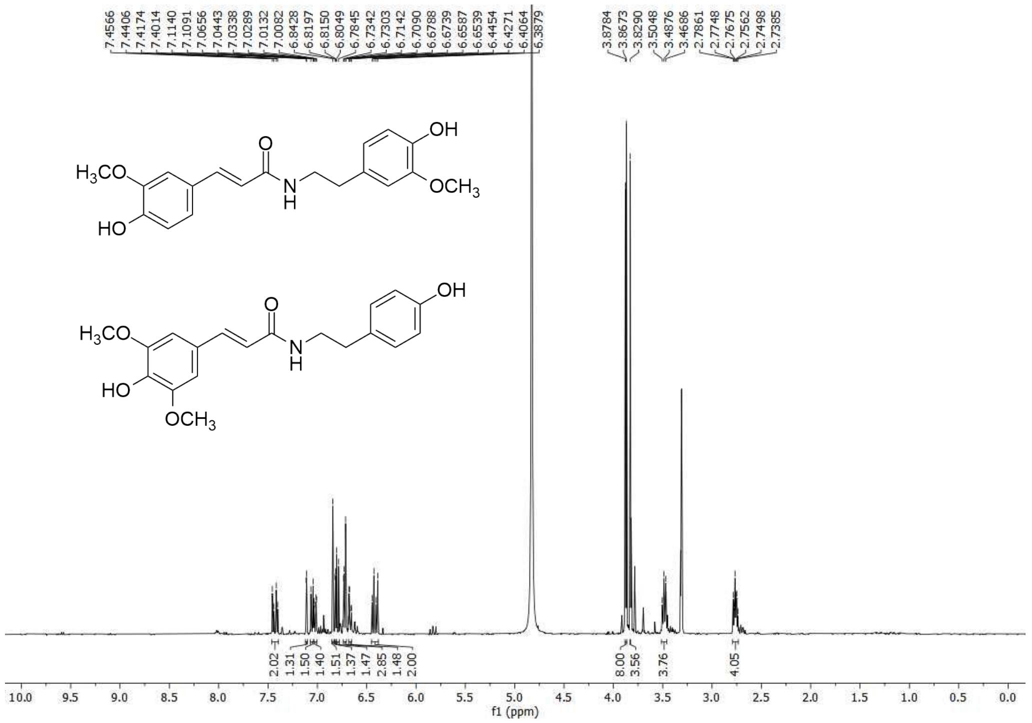

Figure S81. ^{1}H NMR spectrum of compound **11**, **12** (400 MHz, MeOD).

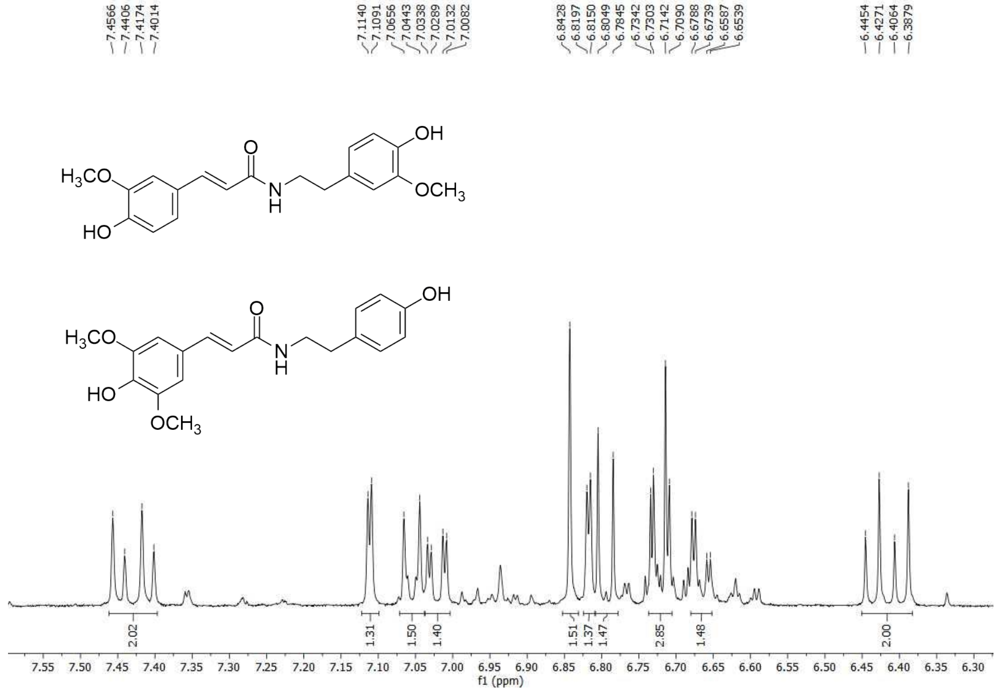

Figure S82. Amplification of the ^{1}H NMR spectrum of compound **11**, **12 (**400 MHz, MeOD).

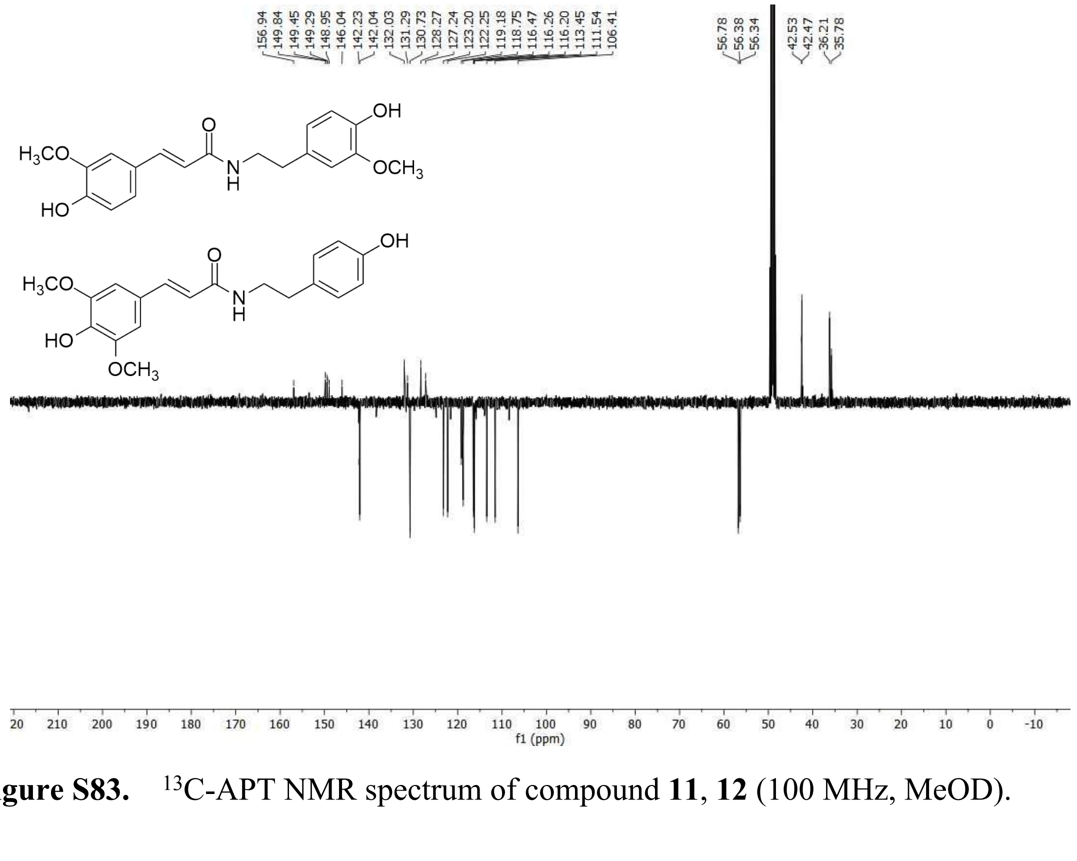

Figure S83. ^{13}C-APT NMR spectrum of compound **11**, **12** (100 MHz, MeOD).

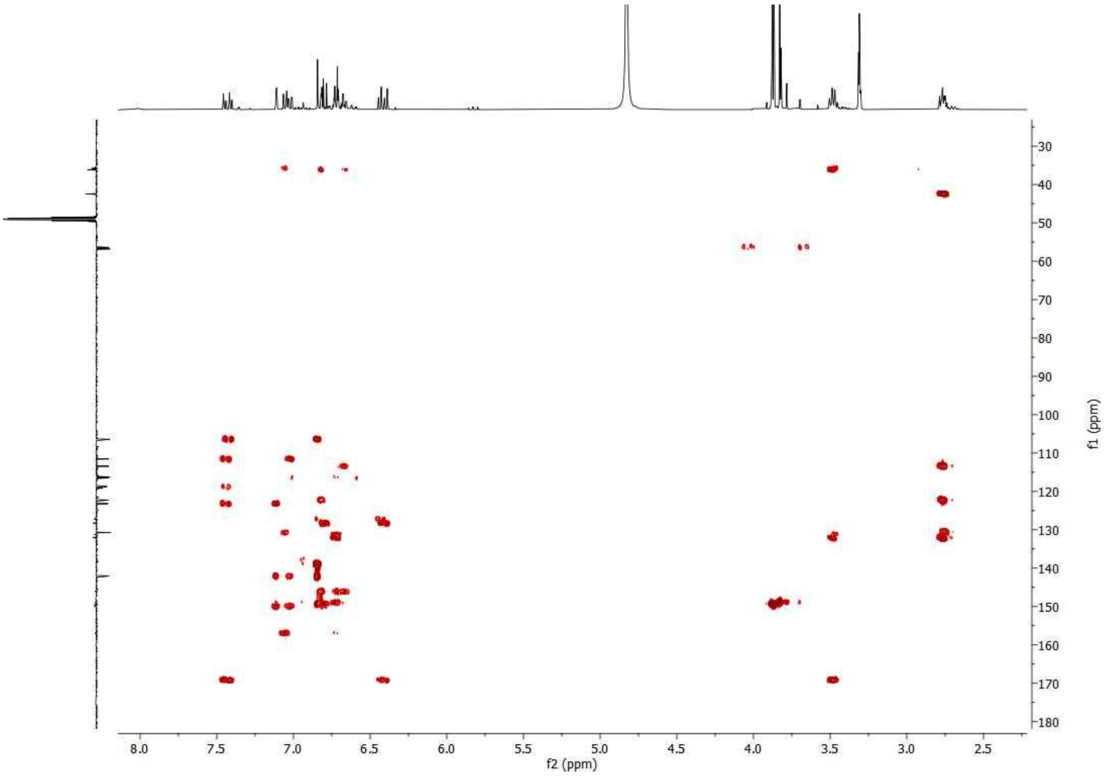

Figure S84. HMBC spectrum of compound **11**, **12** (400/100 MHz, MeOD).

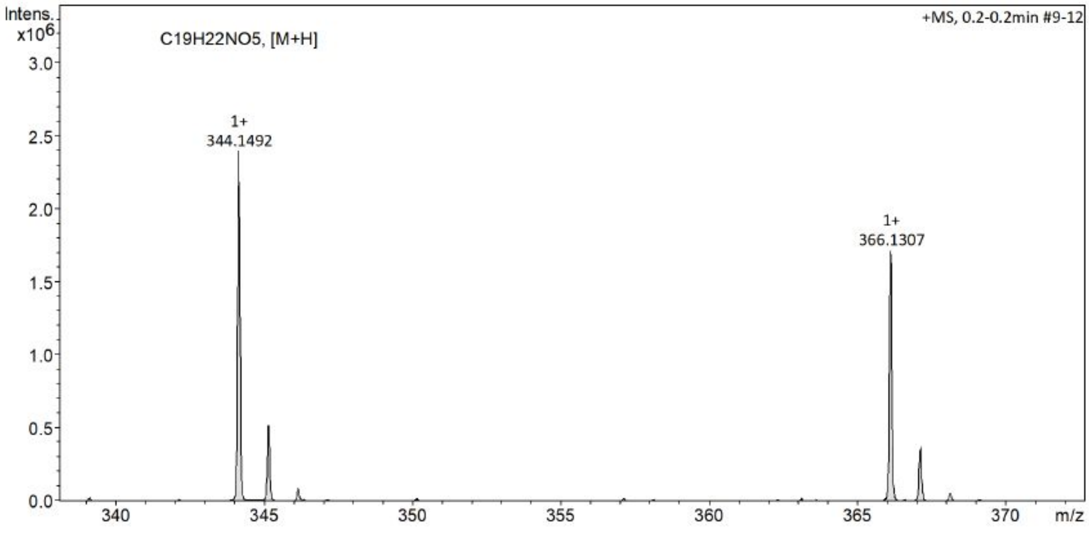

Figure S85. HRESIMS spectrum of compounds **11**, **12** ($[M + H]^+$, positive mode).

12 Spectroscopic data for compounds 13,14

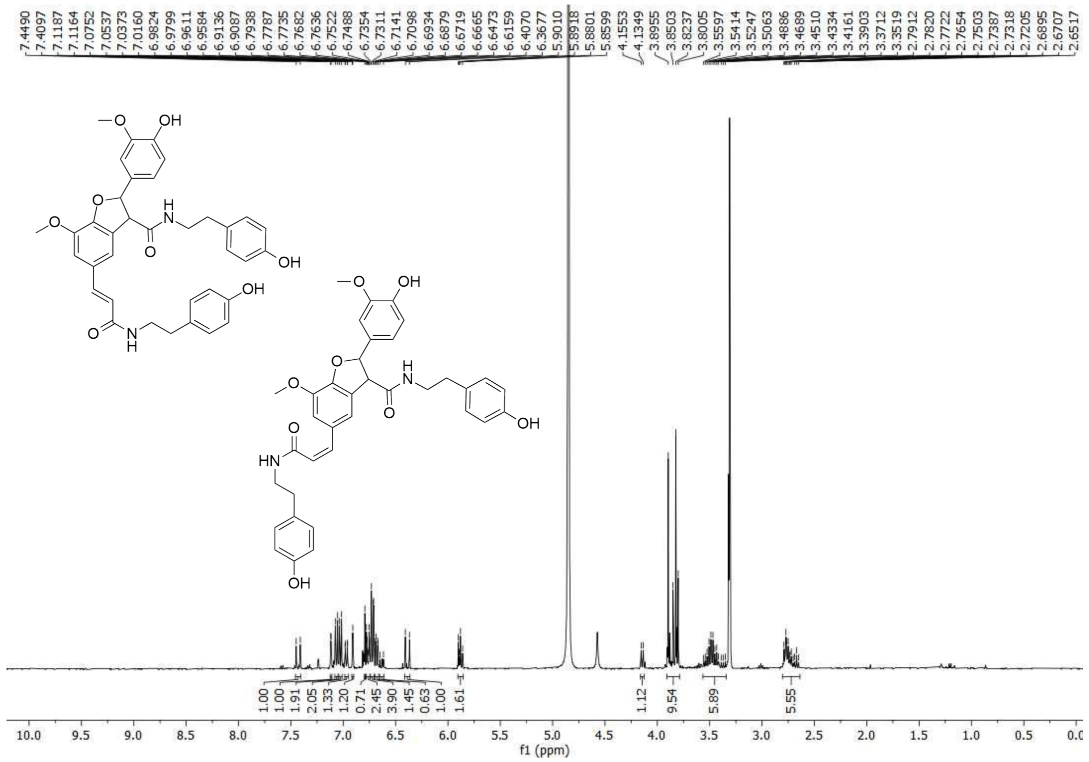

Figure S86. ^{1}H NMR spectrum of compounds **13**, **14 (**400 MHz, MeOD).

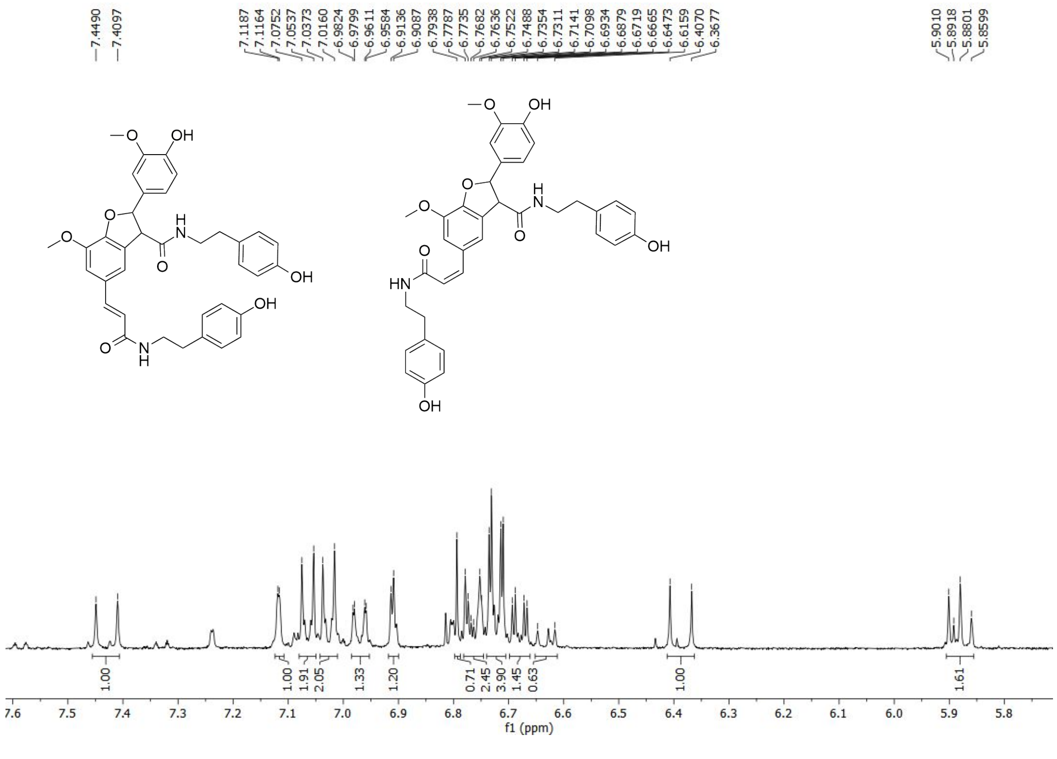

Figure S87. Amplification of the ^{1}H NMR spectrum of compounds **13**, **14 (**400 MHz, MeOD).

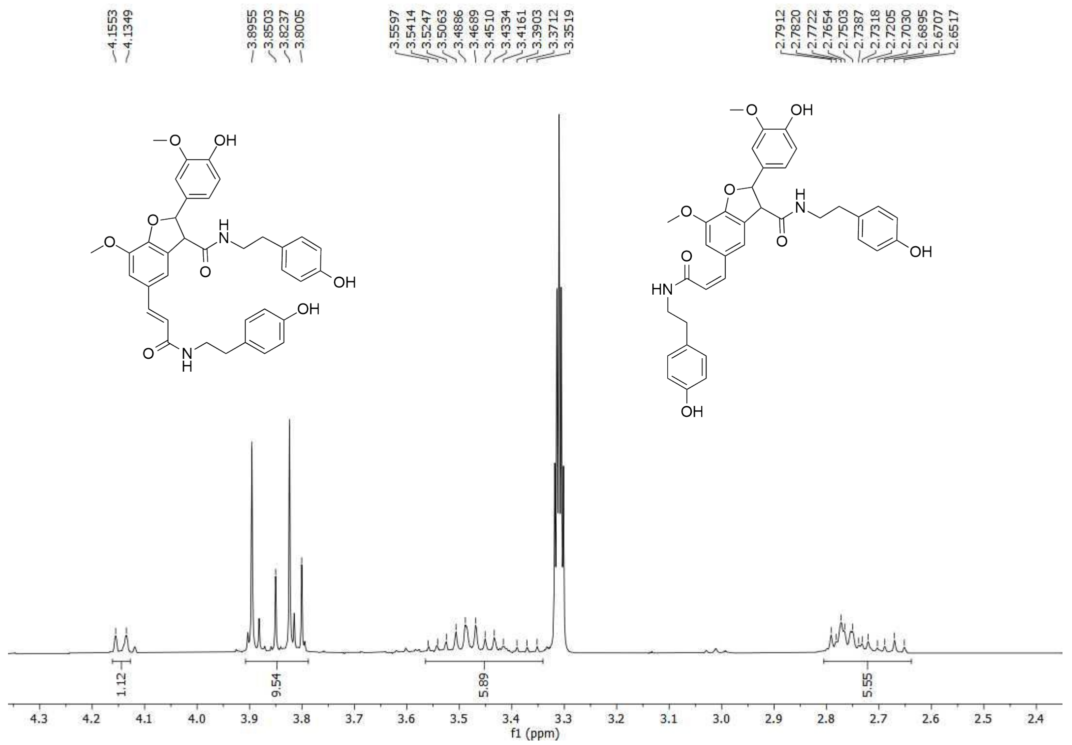

Figure S88. Amplification of the ^{1}H NMR spectrum of compounds **13**, **14** (400 MHz, MeOD).

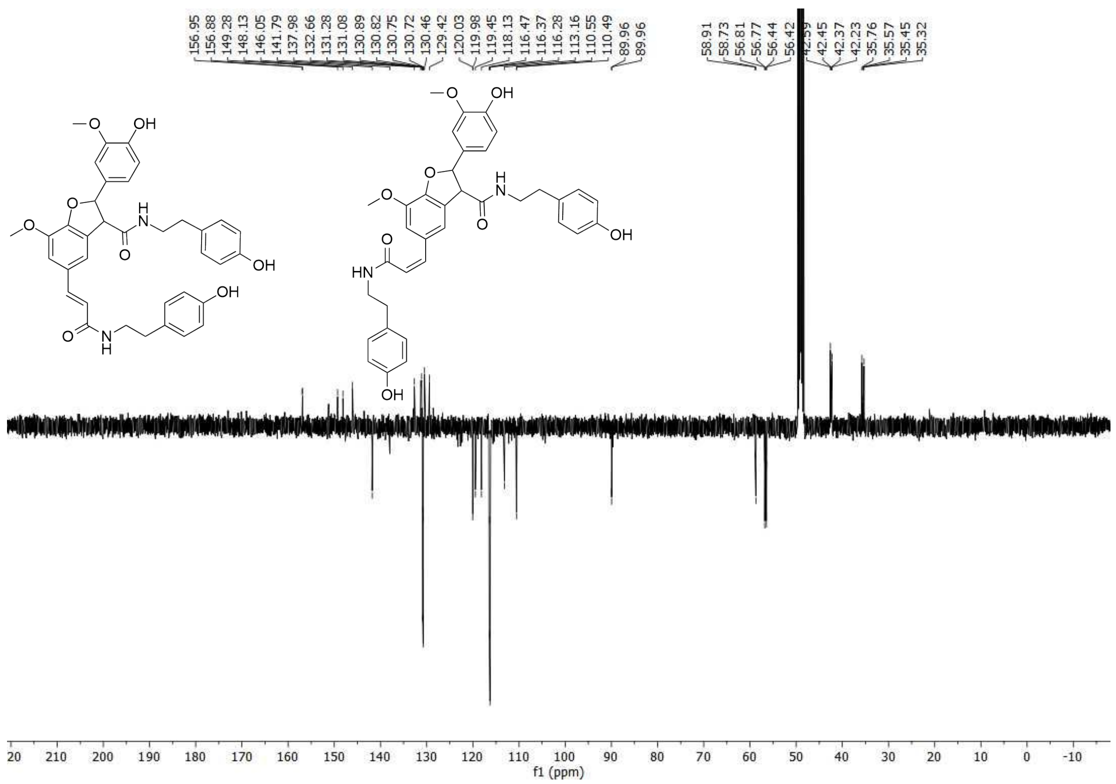

Figure S89. ^{13}C-APT NMR spectrum of compounds **13**, **14** (100 MHz, MeOD).

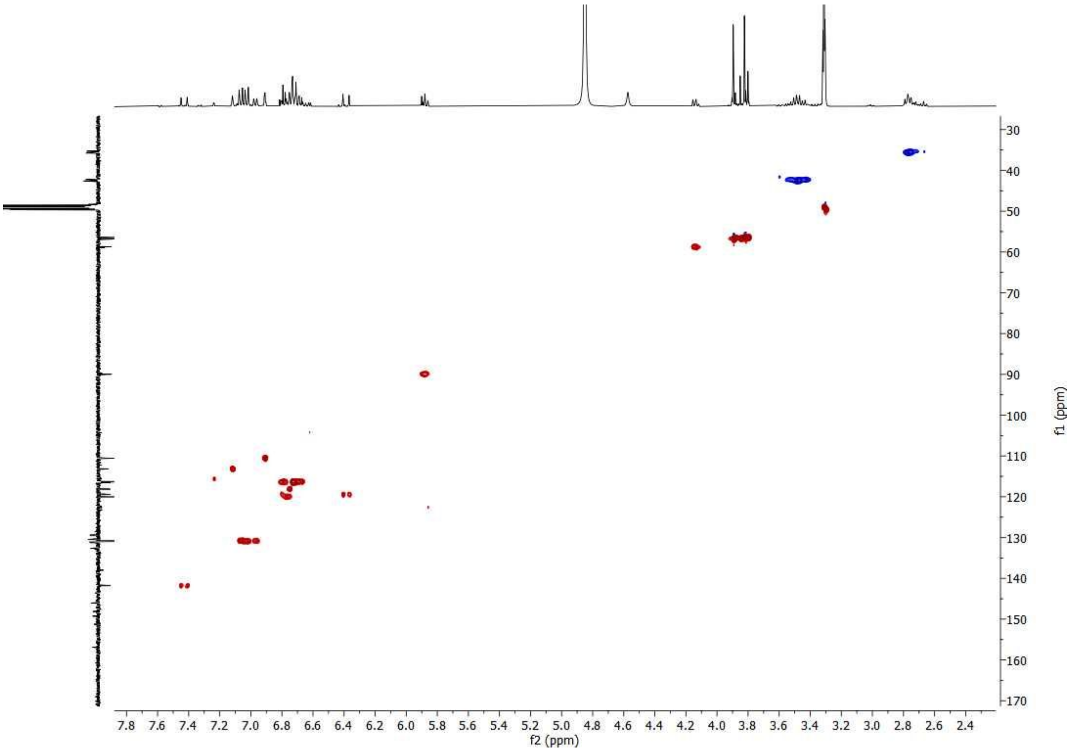

Figure S90. HSQC spectrum of compounds **13**, **14 (**400/100 MHz, MeOD).

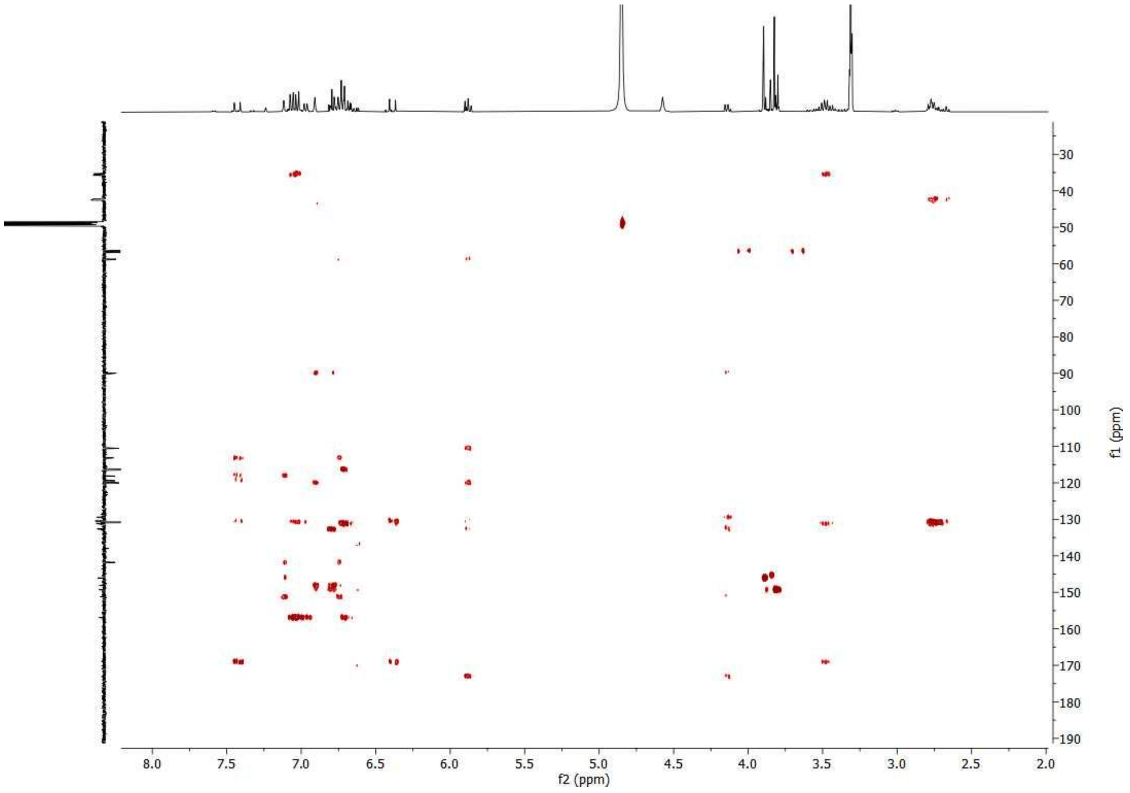

Figure S91. HMBC spectrum of compounds **13**, **14 (**400/100 MHz, MeOD).

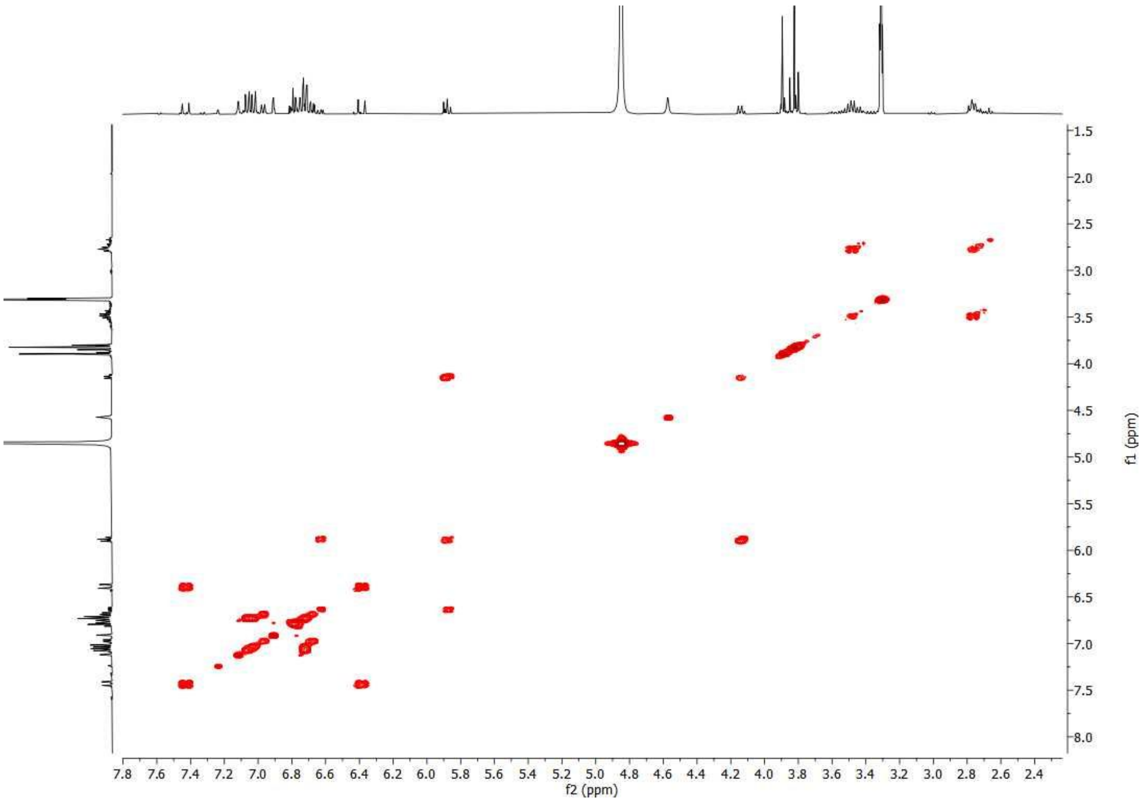

Figure S92. COSY spectrum of compounds **13**, **14 (**400 MHz, MeOD).

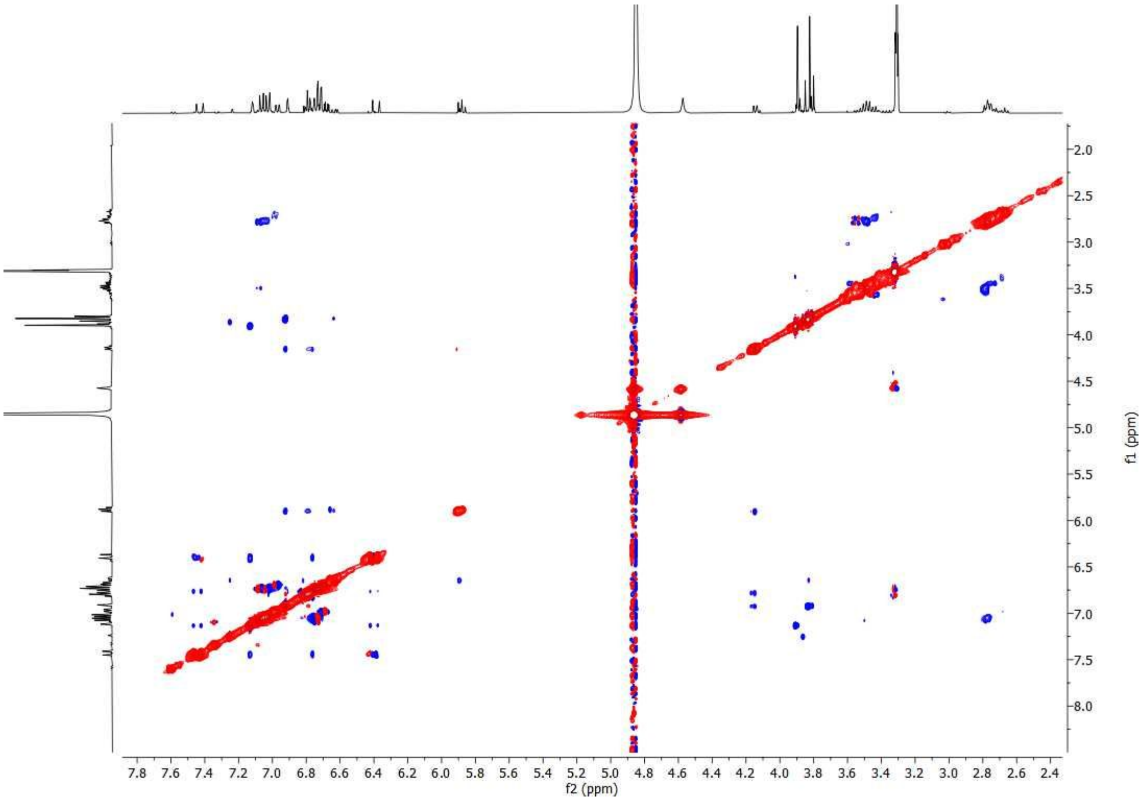

Figure S93. COSY spectrum of compounds **13**, **14 (**400 MHz, MeOD).

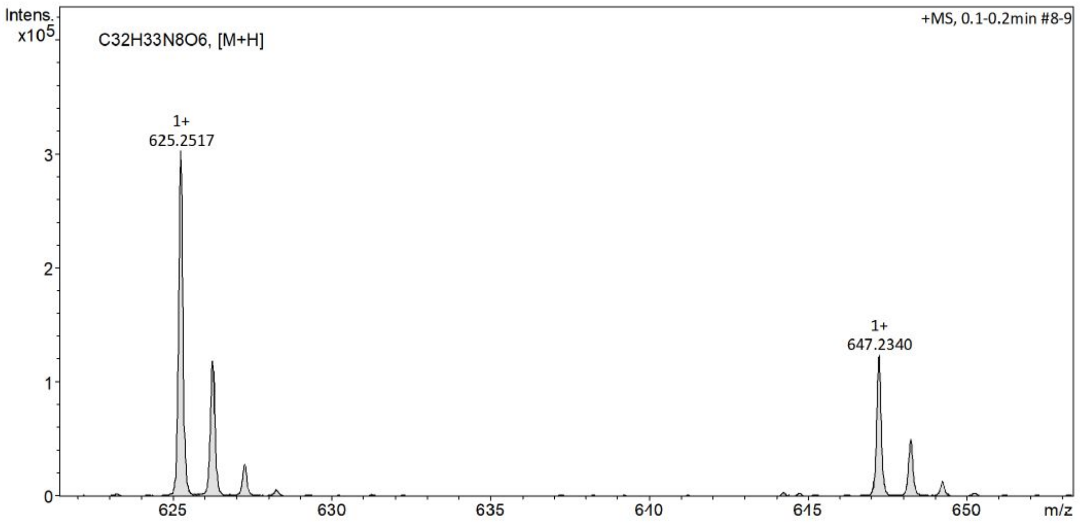

Figure S94. HRESIMS spectrum of compounds **13**, **14** ($[M + H]^+$, positive mode).

13 Spectroscopic data for compound 15

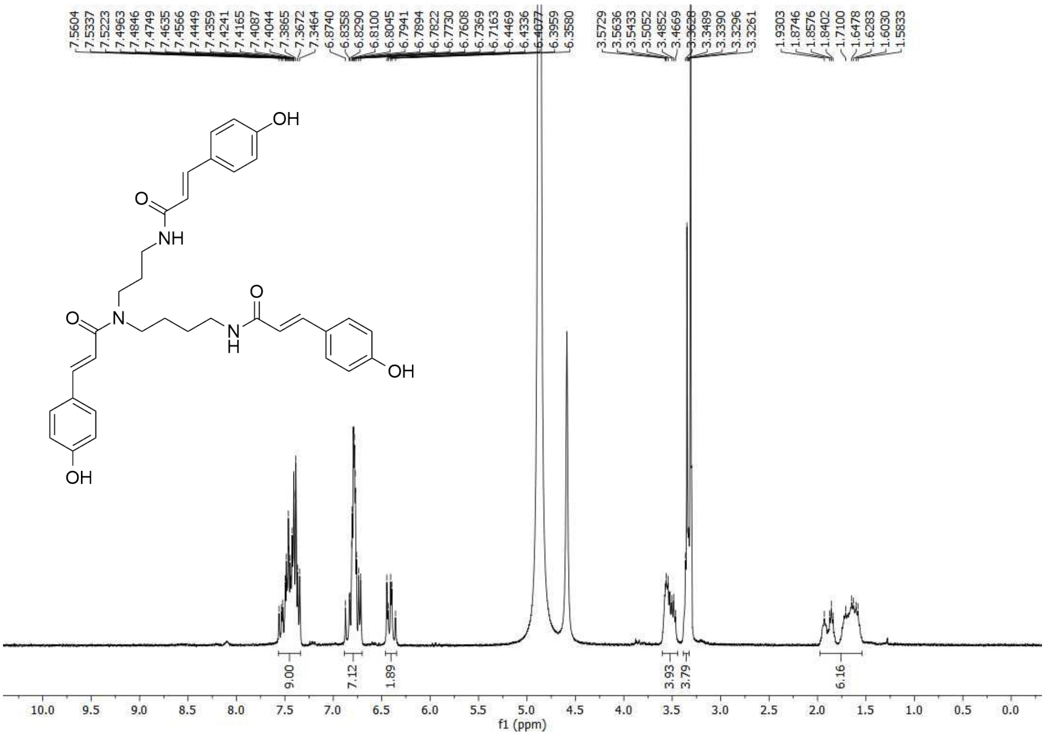

Figure S95. Amplification of the ^{1}H NMR spectrum of compound **15** (400 MHz, MeOD).

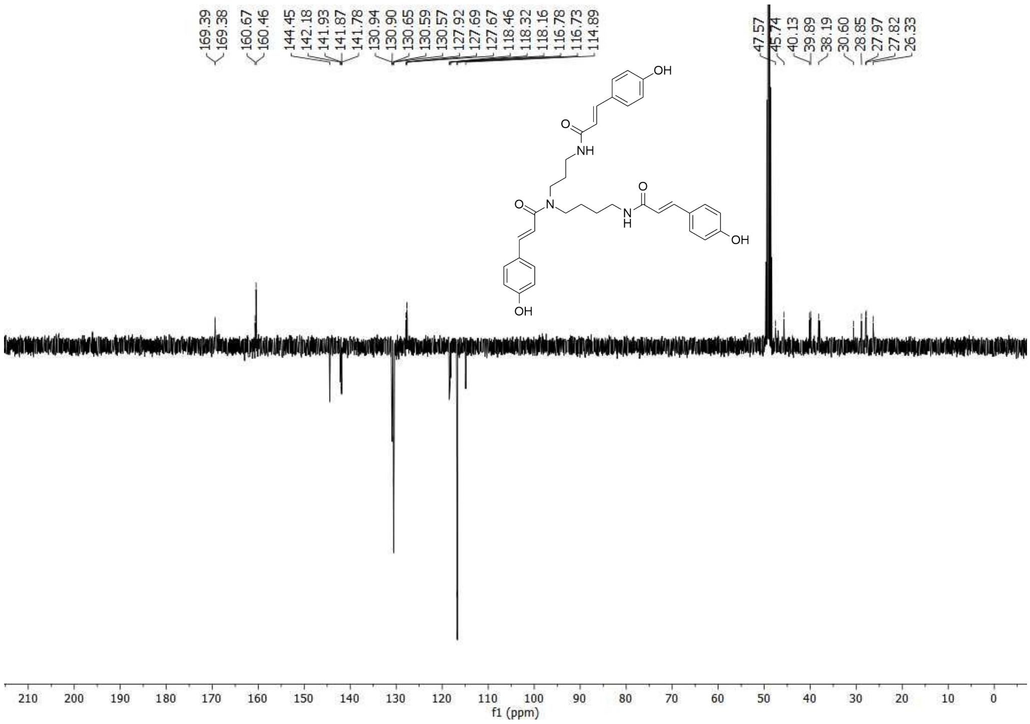

Figure S96. ^{13}C-APT NMR spectrum of compound **15** (100 MHz, MeOD).

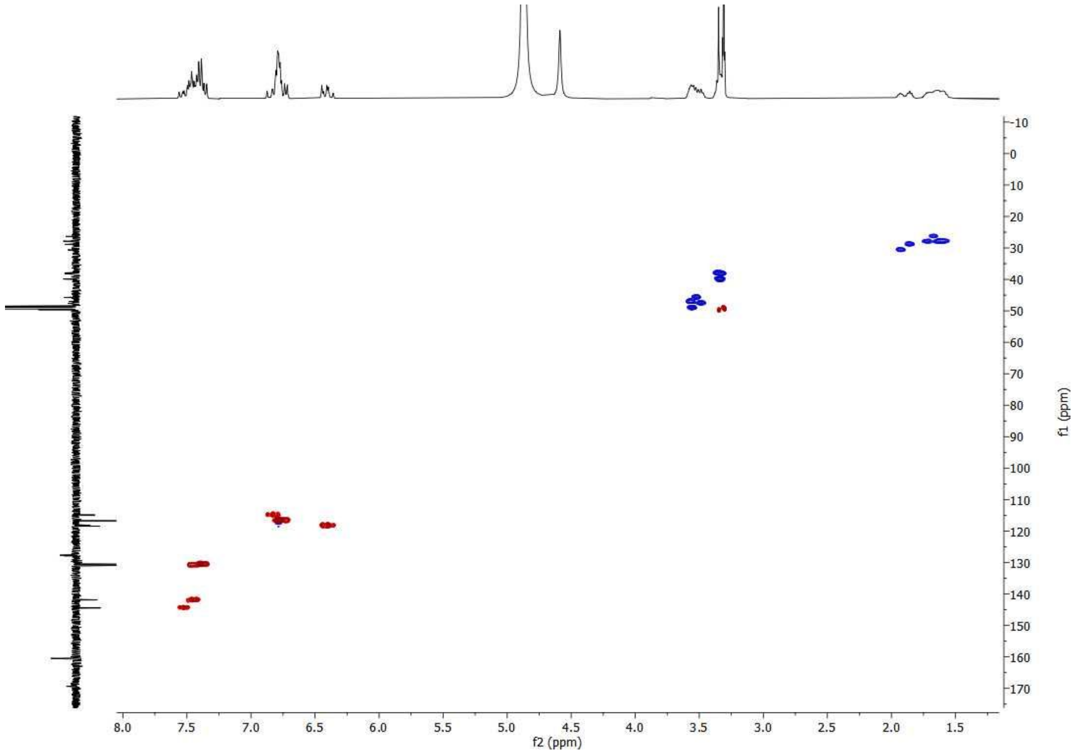

Figure S97. HSQC spectrum of compound **15 (**400/100 MHz, MeOD).

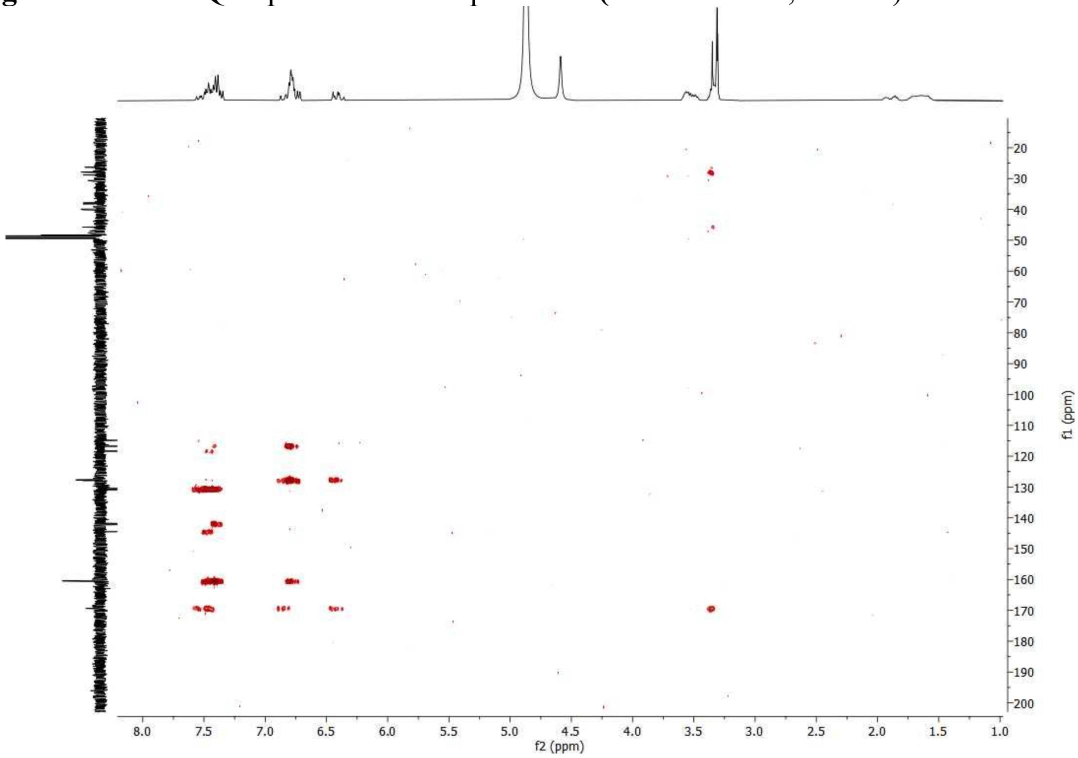

Figure S98. HMBC spectrum of compound **15 (**400/100 MHz, MeOD).

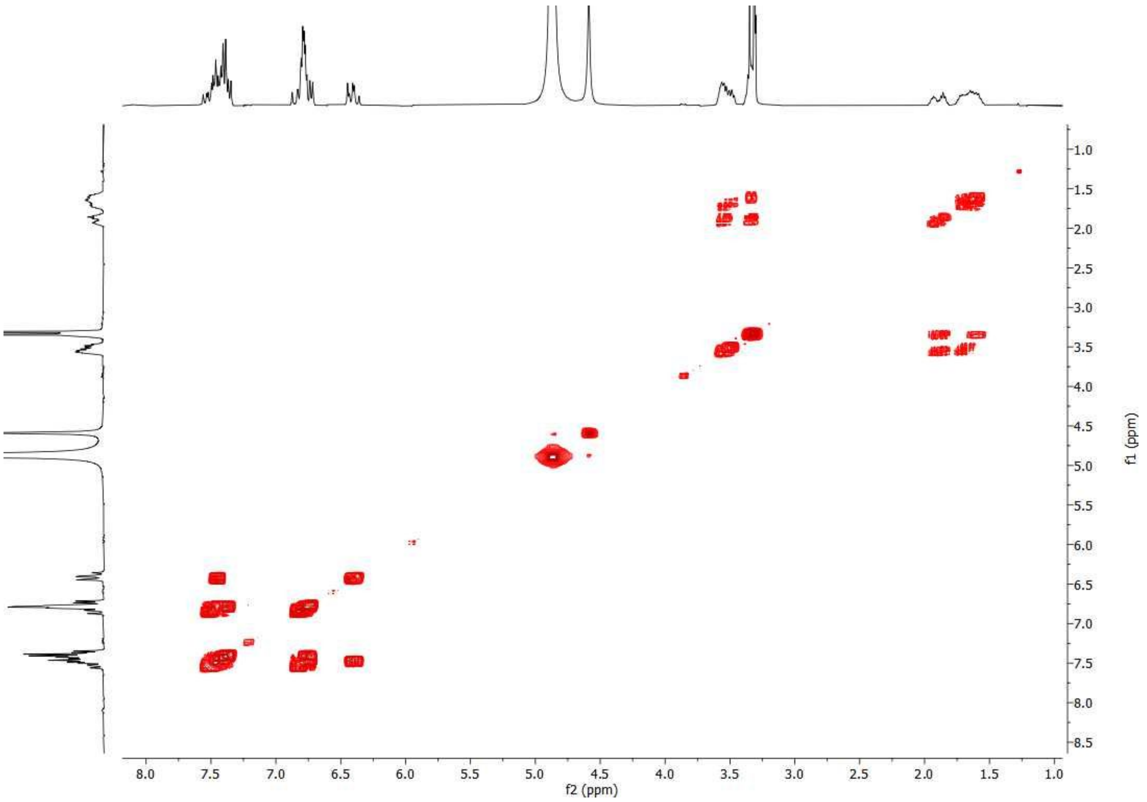

Figure S99. COSY spectrum of compound **15 (**400 MHz, MeOD).

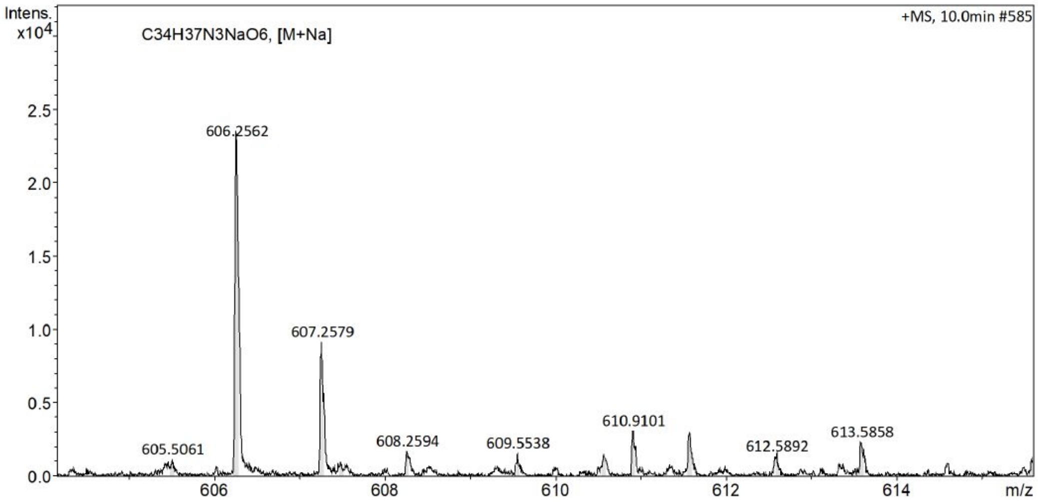

Figure S100. HRESIMS spectrum of compound **15** ($[M + Na]^+$, positive mode).

14 Quantum mechanical calculations for compound 1.

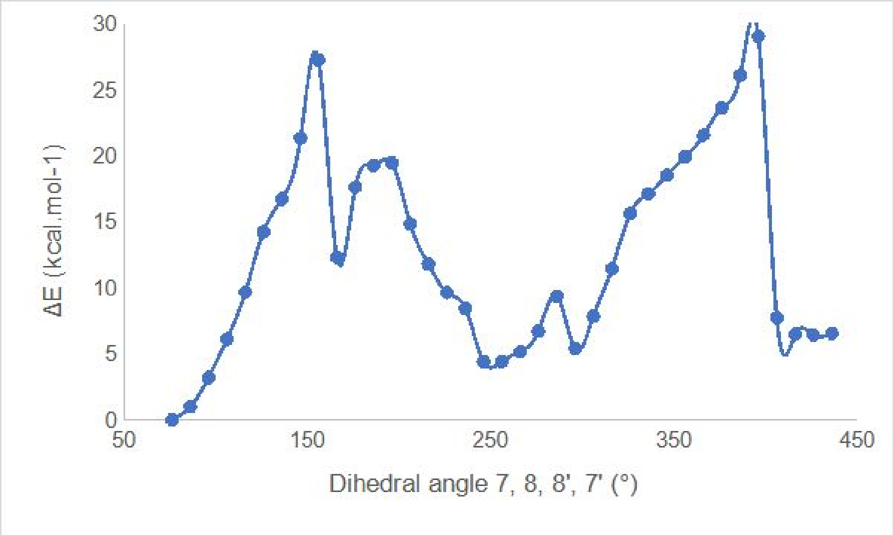

Figure S101. Calculated energy levels (in $kcal.mol^{-1}$) for the conformers of compound **1** as a function of rotation around the C7-C8-C8′-C7′ dihedral angle.

15 NMR calculation data for compound 1.

Table S1. DP4+ probabilities obtained by correlating the calculated ^{13}C and ^{1}H NMR chemical shifts of the 4 possible diastereoisomers of (a*S*)-compound **1** with the experimental NMR data of the isolated natural product.

	7″R*,7‴R*,8aS	***7″S*,7‴S*,8aS***	***7″R*,7‴S*,8aS***	***7″S*,7‴R*,8aS***
**DP4+ Probabilities**	0.00%	100.00%	0.00%	0.00%

Table S2. Calculated [GIAO-mPW1PW91/6-31G(d)-PCM//B3LYP/6-31G(d)] ^{1}H NMR chemical shifts (in ppm), obtained for 4 diastereoisomers of compound **1**, and experimental data of the isolated natural product.

**Nuclei**	δ_{calc} **[7″*R**,7‴*R**, 8a*S*]**	δ_{calc} **[7″*S**,7‴*S**, 8a*S*]**	δ_{calc} **[7″*R**,7‴*S**, 8a*S*]**	δ_{calc} **[7″*S**,7‴*R**, 8a*S*]**	δ_{exp}
**2**	8.45	6.13	6.70	6.65	6.87
**6**	7.62	6.99	6.37	6.66	6.87
**7**	8.06	6.53	7.71	6.44	7.82
**2′**	7.57	6.30	6.50	6.87	6.87
**6′**	7.41	6.65	7.17	8.06	6.87
**7′**	7.85	7.54	6.81	7.26	7.86
**2″**	8.23	7.84	7.21	7.52	6.94
**3″**	7.83	7.29	6.80	6.73	6.63
**5″**	7.39	6.59	6.30	6.93	6.63
**6″**	8.31	7.58	7.44	7.49	6.94
**7″**	6.03	4.98	5.08	5.29	4.54
**8a″**	4.35	4.05	2.98	3.95	3.40
**8b″**	5.58	3.35	4.42	2.68	3.40
**2‴**	8.50	7.22	7.36	7.59	7.03
**3‴**	7.86	6.95	6.88	6.82	6.69
**5‴**	7.67	6.67	6.75	6.81	6.69
**6‴**	8.49	7.29	7.85	7.24	7.03
**7‴**	6.13	4.85	5.08	4.87	4.38
**8a‴**	3.76	4.00	4.00	2.93	3.37
**8b‴**	4.91	3.02	2.70	4.36	3.37
**3′,5′-OCH$_3$**	4.92	3.84	3.99	3.94	3.75
**3-OCH$_3$**	4.87	3.78	3.86	4.03	3.75
**5-OCH$_3$**	4.86	3.94	3.79	3.65	3.75

Table S3. Calculated [GIAO-mPW1PW91/6-31G(d)-PCM//B3LYP/6-31G(d)] ^{13}C NMR chemical shifts (in ppm), obtained for 4 diastereoisomers of compound **1**, and experimental data of the isolated natural product.

Nuclei	δ_{calc} [7″*R**,7‴*R**, 8a*S*]	δ_{calc} [7″*S**,7‴*S**, 8a*S*]	δ_{calc} [7″*R**,7‴*S**, 8a*S*]	δ_{calc} [7″*S**,7‴*R**, 8a*S*]	δ_{exp}
**1**	119.5	115.6	118.3	116.3	126.7
**2**	104.1	103.5	109.6	114.1	108.8
**3**	139.1	136.1	134.1	134.3	139.0
**4**	134.3	128.5	131.5	132.5	149.2
**5**	137.9	136.7	137.9	137.7	139.0
**6**	111.1	98.7	101.2	97.5	108.8
**7**	136.6	130.5	134.2	123.1	142.3
**8**	125.4	122.1	126.0	125.9	127.4
**9**	166.2	160.9	163.3	160.1	168.2
**1′**	120.2	117.3	116.3	116.9	126.7
**2′**	104.0	99.6	113.7	107.5	108.9
**3′**	138.7	137.8	135.7	136.1	139.1
**4′**	132.5	130.1	132.9	131.8	149.2
**5′**	138.2	135.5	138.1	137.5	139.1
**6′**	107.6	108.6	100.5	100.4	108.9
**7′**	131.7	136.1	130.8	135.9	142.5
**8′**	130.5	124.1	123.1	123.3	127.6
**9′**	164.1	162.0	161.6	161.5	168.2
**1″**	125.5	126.6	124.9	123.0	134.2
**2″**	121.7	120.9	119.2	118.4	128.4
**3″**	109.5	107.6	105.5	105.5	116.0
**4″**	146.8	144.2	144.7	144.9	158.0
**5″**	108.9	104.7	104.9	105.9	116.0
**6″**	121.9	122.5	120.2	118.7	128.4
**7″**	75.9	70.4	71.7	68.8	73.0
**8″**	49.2	43.8	45.7	45.1	48.5
**1‴**	128.0	125.1	124.7	121.8	134.3
**2‴**	121.5	118.3	119.0	117.7	128.3
**3‴**	108.6	106.5	105.8	107.6	116.1
**4‴**	146.5	145.1	145.0	145.5	158.0
**5‴**	108.9	105.7	105.6	108.0	116.1
**6‴**	121.7	119.3	118.8	118.7	128.3
**7‴**	72.1	70.0	67.6	67.1	73.5
**8‴**	51.3	47.0	43.6	44.7	48.5

**3′,5′-OCH_3**	56.0	50.4	52.8	52.0	56.7
**3-OCH_3**	56.3	49.7	53.9	53.6	56.7
**5-OCH_3**	57.3	51.7	50.4	50.5	56.7

16 NMR calculation data for compound 2.

Table S4. DP4+ probabilities obtained by correlating the calculated ^{13}C and ^{1}H NMR chemical shifts of the 4 possible diastereoisomers of compound **2** with the experimental NMR data of the isolated natural product.

	7S,7′S*,7″S*, 7‴R*,8S*,8′S**	*7S*,7′S*,7″R*, 7‴S*,8S*,8′S**	*7S*,7′S*,7″R*, 7‴R*,8S*,8′S**	*7S*,7′S*,7″S*, 7‴S*,8S*,8′S**
**DP4+ Probabilities**	0.00%	0.00%	100.00%	0.00%

Table S5. Calculated [GIAO-mPW1PW91/6-31G(d)-PCM//B3LYP/6-31G(d)] ^{1}H NMR chemical shifts (in ppm), obtained for 4 diastereoisomers of compound **2**, and experimental data of the isolated natural product.

Nuclei	δ_{calc} [7*S**,7′*S**,7″*S**, 7‴*R**,8*S**,8′*S**]	δ_{calc} [7*S**,7′*S**,7″*R**, 7‴*S**,8*S**,8′*S**]	δ_{calc} [7*S**,7′*S**,7″*R**, 7‴*R**,8*S**,8′*S**]	δ_{calc} [7*S**,7′*S**,7″*S**, 7‴*S**,8*S**,8′*S**]	δ_{exp}
**2**	6.00	5.88	6.39	6.71	6.73
**6**	6.98	7.00	7.08	6.96	6.73
**7**	5.46	5.52	5.20	6.18	5.27
**8**	1.62	1.20	2.81	3.16	3.38
**2′**	5.89	6.98	6.97	7.18	6.73
**6′**	6.98	6.00	6.84	6.82	6.73
**7′**	5.53	5.46	6.13	4.78	5.29
**8′**	1.17	1.63	3.55	2.23	3.38
**2″**	7.18	7.99	7.25	7.46	6.96
**3″**	6.18	7.04	7.02	6.96	6.66
**5″**	7.07	6.28	6.89	7.41	6.66
**6″**	7.52	7.20	7.79	7.91	6.96
**7″**	5.00	5.18	4.22	5.33	4.49
**8a″**	3.67	3.06	2.69	3.70	3.45
**8b″**	3.46	4.47	3.70	4.13	3.22
**2‴**	7.26	7.19	7.75	7.65	6.96
**3‴**	6.35	6.24	7.00	7.11	6.66
**5‴**	6.97	7.08	6.79	7.16	6.66
**6‴**	7.92	7.54	7.71	7.85	6.96
**7‴**	5.18	5.00	4.88	5.51	4.49
**8a‴**	4.47	3.67	4.54	3.24	3.45
**8b‴**	3.05	3.46	3.25	4.55	3.22
**3′,5′-OCH_3**	4.08	3.94	4.04	4.58	3.87
**3-OCH_3**	4.10	4.10	4.07	4.19	3.87
**5-OCH_3**	3.94	3.95	4.12	4.46	3.87

Table S6. Calculated [GIAO-mPW1PW91/6-31G(d)-PCM//B3LYP/6-31G(d)] ^{13}C NMR chemical shifts (in ppm), obtained for 4 diastereoisomers of compound **2**, and experimental data of the isolated natural product.

Nuclei	δ_{calc} [7*S**,7′*S**,7″*S**, 7‴*R**,8*S**,8′*S**]	δ_{calc} [7*S**,7′*S**,7″*R**, 7‴*S**,8*S**,8′*S**]	δ_{calc} [7*S**,7′*S**,7″*R**, 7‴*R**,8*S**,8′*S**]	δ_{calc} [7*S**,7′*S**,7″*S**, 7‴*S**,8*S**,8′*S**]	δ_{exp}
**1**	126.0	125.3	123.1	128.7	132.3
**2**	104.8	105.4	107.0	97.8	104.8
**3**	134.8	134.1	135.6	138.6	149.4
**4**	129.4	130.0	130.9	130.2	136.6
**5**	137.6	137.4	138.6	135.1	149.4
**6**	96.3	97.9	95.8	103.5	104.8
**7**	75.9	76.0	79.3	76.7	86.3
**8**	56.4	55.7	57.5	54.6	60.3
**9**	163.5	159.7	163.1	163.9	172.5
**1′**	125.4	126.0	124.7	122.7	132.3
**2′**	104.5	96.3	95.6	108.4	104.8
**3′**	134.3	137.6	138.1	136.6	149.4
**4′**	129.6	129.4	130.2	132.3	136.6
**5′**	137.4	134.8	136.3	138.4	149.4
**6′**	97.5	104.8	105.1	101.5	104.8
**7′**	76.0	75.9	77.9	80.2	86.3
**8′**	55.9	56.4	53.5	56.3	60.3
**9′**	159.7	163.5	158.6	162.5	172.5
**1″**	124.2	120.4	123.8	125.2	134.2
**2″**	118.9	119.1	119.6	119.3	128.5
**3″**	107.1	107.6	106.5	107.8	116.1
**4″**	145.8	146.1	145.4	145.8	158.0
**5″**	107.0	107.4	106.0	107.3	116.1
**6″**	118.7	119.3	119.5	119.5	128.5
**7″**	67.2	69.0	71.7	69.7	73.1
**8″**	46.1	40.5	47.5	48.1	48.1
**1‴**	120.4	124.1	124.7	121.1	134.2
**2‴**	119.3	118.9	121.0	119.6	128.5
**3‴**	107.4	107.3	106.7	107.5	116.1
**4‴**	146.1	145.9	145.2	146.6	158.0
**5‴**	107.6	107.0	106.2	108.5	116.1
**6‴**	119.1	118.8	120.6	120.7	128.5
**7‴**	69.0	67.2	68.1	69.6	73.1
**8‴**	40.4	46.1	39.8	44.7	48.1

**3′,5′-OCH$_3$**	52.3	52.3	52.1	54.1	56.8
**3-OCH$_3$**	54.2	54.2	53.4	52.1	56.8
**5-OCH$_3$**	50.4	50.6	51.1	55.6	56.8

17 NMR calculation data for compound 3.

Table S7. DP4+ probabilities obtained by correlating the calculated ^{13}C and ^{1}H NMR chemical shifts of both stereoisomers of compound **3** with the experimental NMR data of the isolated natural product.

	7S,7′S,7‴S,8S,8′S	*7R,7′R,7‴S,8R,8′R*
**DP4+ Probabilities**	100.00%	0.00%

Table S8. Calculated [GIAO-mPW1PW91/6-31G(d)-PCM//B3LYP/6-31G(d)] ^{1}H NMR chemical shifts (in ppm), obtained for both diastereoisomers of compound **3**, and experimental data of the isolated natural product.

Nuclei	δ_{calc} [7*S*,7′*S*,7‴*S*,8*S*,8′*S*]	δ_{calc} [7*R*,7′*R*,7‴*S*,8*R*,8′*R*]	δ_{exp}
**2**	6.49	6.25	6.71
**6**	7.13	6.89	6.71
**7**	5.55	5.34	5.26
**8**	2.22	0.87	3.35
**2′**	6.26	6.61	6.73
**6′**	7.09	6.58	6.73
**7′**	5.45	5.63	5.28
**8′**	1.76	1.38	3.40
**2″**	7.38	7.52	6.83
**3″**	6.53	6.92	6.63
**5″**	7.02	6.36	6.63
**6″**	7.24	6.99	6.83
**7a″**	2.87	2.64	2.59
**7b″**	2.40	3.02	2.53
**8a″**	2.80	3.77	3.37
**8b″**	4.28	3.00	3.18
**2‴**	7.60	7.12	6.95
**3‴**	7.22	6.26	6.66
**5‴**	6.72	6.82	6.66
**6‴**	7.49	7.81	6.95
**7‴**	5.15	5.13	4.46
**8a‴**	3.38	4.38	3.49
**8b‴**	3.89	3.05	3.18
**3,5-OCH3**	4.12	4.03	3.88
**3′,5′-OCH3**	4.07	4.00	3.86

Table S9. Calculated [GIAO-mPW1PW91/6-31G(d)-PCM//B3LYP/6-31G(d)] ^{13}C NMR chemical shifts (in ppm), obtained for both diastereoisomers of compound **3**, and experimental data of the isolated natural product.

Nuclei	δ_{calc} [7*S*,7′*S*,7‴*S*,8*S*,8′*S*]	δ_{calc} [7*R*,7′*R*,7‴*S*,8*R*,8′*R*]	δ_{exp}
**1**	123.2	126.3	131.0
**2**	103.7	98.7	104.7
**3**	136.0	135.7	149.5
**4**	129.2	127.7	136.7
**5**	138.2	136.8	149.5
**6**	95.2	97.9	104.7
**7**	78.3	79.1	86.4
**8**	58.4	58.4	60.6
**9**	159.1	160.1	172.1
**1′**	125.6	126.8	132.4
**2′**	105.3	101.1	104.9
**3′**	135.2	136.0	149.4
**4′**	129.8	129.3	136.7
**5′**	137.9	136.0	149.4
**6′**	96.8	100.6	104.9
**7′**	77.6	78.5	86.4
**8′**	56.2	56.6	60.3
**9′**	165.9	161.1	172.5
**1″**	120.3	121.0	130.8
**2″**	121.9	121.8	130.7
**3″**	108.2	106.7	116.2
**4″**	145.6	145.3	156.9
**5″**	106.9	107.3	116.2
**6″**	122.3	122.5	130.7
**7″**	32.5	31.1	35.7
**8″**	33.8	37.0	42.6
**1‴**	126.5	121.0	134.5
**2‴**	119.5	118.7	128.5
**3‴**	107.3	106.9	116.1
**4‴**	145.2	145.5	158.0
**5‴**	106.0	106.5	116.1
**6‴**	118.8	118.8	128.5
**7‴**	68.4	68.4	73.2
**8‴**	47.2	41.2	48.1
**3,5-OCH3**	51.2	70.9	56.8
**3′,5′-OCH3**	54.6	51.8	56.9

18 NMR calculation data for compound 4.

Table S10. DP4+ probabilities obtained by correlating the calculated ^{13}C and ^{1}H NMR chemical shifts of the 4 possible diastereoisomers of compound **4** with the experimental NMR data of the isolated natural product.

	7′S*,7″R*, 7‴R*,8′R*	***7′S*,7″S*, 7‴S*,8′R****	***7′S*,7″R*, 7‴S*,8′R****	***7′S*,7″S*, 7‴R*,8′R****
**DP4+ Probabilities**	0.00%	0.00%	100.00%	0.00%

Table S11. Calculated [GIAO-mPW1PW91/6-31G(d)-PCM//B3LYP/6-31G(d)] ^{1}H NMR chemical shifts (in ppm), obtained for 4 diastereoisomers of compound **4**, and experimental data of the isolated natural product.

Nuclei	δ_{calc} [7′*S**,7″*R**, 7‴*R**,8′*R**]	δ_{calc} [7′*S**,7″*S**, 7‴*S**,8′*R**]	δ_{calc} [7′*S**,7″*R**, 7‴*S**,8′*R**]	δ_{calc} [7′*S**,7″*S**, 7‴*R**,8′*R**]	δ_{exp}
**2**	6.59	6.53	6.84	6.57	6.77
**7**	7.02	6.76	7.47	7.37	7.33
**2′**	5.66	6.82	6.45	6.59	6.33
**6′**	6.49	7.07	6.22	5.95	6.33
**7′**	5.25	4.67	4.87	4.61	4.87
**8′**	3.60	3.87	3.37	2.59	3.72
**2″**	7.15	7.40	7.46	7.07	7.14
**3″**	6.80	6.91	6.66	6.25	6.72
**5″**	6.38	6.73	6.81	6.89	6.72
**6″**	7.13	7.62	7.39	7.24	7.14
**7″**	5.03	5.19	5.11	5.09	4.68
**8a″**	3.29	2.70	3.23	3.33	3.39
**8b″**	4.08	3.58	3.22	4.05	3.39
**2‴**	7.49	7.55	7.21	7.25	6.96
**3‴**	6.71	6.87	6.74	6.89	6.67
**5‴**	6.91	6.78	6.92	6.70	6.67
**6‴**	7.54	7.26	7.44	7.25	6.96
**7‴**	4.85	4.42	4.84	5.02	4.53
**8a‴**	3.51	3.66	3.38	4.06	3.34
**8b‴**	2.87	2.91	3.29	3.12	3.25
**3′,5′-OCH$_3$**	3.66	3.89	3.90	4.18	3.68
**3-OCH$_3$**	3.97	3.95	4.04	4.15	3.92
**5-OCH$_3$**	4.05	3.89	3.49	3.63	3.58

Table S12. Calculated [GIAO-mPW1PW91/6-31G(d)-PCM//B3LYP/6-31G(d)] ^{13}C NMR chemical shifts (in ppm), obtained for 4 diastereoisomers of compound **4**, and experimental data of the isolated natural product.

Nuclei	δ_{calc} [7''*R**,7'''*R**, 8a*S*]	δ_{calc} [7''*S**,7'''*S**, 8a*S*]	δ_{calc} [7''*R**,7'''*S**, 8a*S*]	δ_{calc} [7''*S**,7'''*R**, 8a*S*]	δ_{exp}
**1**	115.4	113.9	116.0	116.9	124.2
**2**	100.7	99.6	109.2	101.3	109.1
**3**	136.9	137.1	137.1	137.8	149.3
**4**	135.5	133.1	137.2	134.4	143.3
**5**	135.1	135.5	136.9	135.3	146.9
**6**	119.8	115.9	118.1	114.1	125.2
**7**	129.9	121.7	129.9	129.6	135.4
**8**	117.9	124.7	118.8	118.4	126.8
**9**	164.4	161.7	160.0	158.8	170.3
**1′**	126.9	129.0	126.9	125.2	135.3
**2′**	99.5	106.1	100.5	103.5	106.0
**3′**	136.5	135.7	136.6	136.3	149.0
**4′**	123.7	129.8	125.8	128.6	135.3
**5′**	137.6	137.7	137.0	137.3	149.0
**6′**	96.6	98.4	96.2	99.4	106.0
**7′**	36.4	36.0	38.0	37.7	41.4
**8′**	47.6	49.1	48.9	47.5	50.2
**9′**	163.6	165.2	162.3	166.7	174.3
**1″**	123.6	124.1	123.5	125.2	134.7
**2″**	117.5	119.3	119.6	118.2	128.4
**3″**	107.0	105.7	106.0	106.3	116.1
**4″**	145.5	144.9	145.5	145.1	158.0
**5″**	106.4	105.7	106.2	106.9	116.1
**6″**	118.3	118.4	119.6	117.8	128.4
**7″**	69.4	67.0	68.3	69.5	73.4
**8″**	47.2	47.1	48.1	46.7	48.4
**1‴**	125.7	124.2	123.1	125.1	134.3
**2‴**	119.2	118.9	119.0	118.9	128.4
**3‴**	105.4	106.6	106.9	106.5	116.1
**4‴**	144.9	145.5	145.8	144.4	157.9
**5‴**	105.8	105.7	107.4	105.7	116.1
**6‴**	119.4	120.8	119.5	118.7	128.4
**7‴**	71.7	70.6	69.1	69.3	72.8
**8‴**	49.7	43.6	44.1	46.4	48.0

**3′,5′-OCH$_3$**	50.6	51.3	50.2	51.1	56.7
**3-OCH$_3$**	50.5	50.2	52.1	51.1	56.8
**5-OCH$_3$**	53.6	53.0	54.9	53.4	60.8

19 NMR calculation data for compound 5.

Table S13. DP4+ probabilities obtained by correlating the calculated ^{13}C and ^{1}H NMR chemical shifts of both diastereoisomers of compound **5** with the experimental NMR data of the isolated natural product.

	7′S*,7‴R*,8′R*	***7′S*,7‴S*,8′R****
**DP4+ Probabilities**	0.00%	100.00%

Table S14. Calculated [GIAO-mPW1PW91/6-31G(d)-PCM//B3LYP/6-31G(d)] ^{1}H NMR chemical shifts (in ppm), obtained for both diastereoisomers of compound **5**, and experimental data of the isolated natural product.

**Nuclei**	δ_{calc} **[7′*S*,7‴*R*,8′*R*]**	δ_{calc} **[7′*S*,7‴*S*,8′*R*]**	δ_{exp}
**2**	6.41	6.62	6.76
**7**	6.20	6.88	7.27
**7′**	5.08	5.31	4.90
**8′**	3.28	3.44	3.72
**2′,6′**	6.04	6.24	6.33
**2″,6″**	7.06	7.17	6.94
**3″,5″**	6.65	6.67	6.66
**7″**	2.82	2.22	2.68
**8″**	3.46	3.70	3.38
**2‴,6‴**	7.39	7.52	6.98
**3‴,5‴**	6.53	6.85	6.67
**7‴**	4.79	3.12	4.52
**8a‴**	3.90	4.15	3.35
**8b‴**	3.22	3.35	3.26
**3′-OCH3**	4.06	4.05	3.70
**5′-OCH3**	3.39	3.87	3.70
**3-OCH3**	4.05	4.24	3.92
**5-OCH3**	4.05	4.11	3.53

Table S15. Calculated [GIAO-mPW1PW91/6-31G(d)-PCM//B3LYP/6-31G(d)] ^{13}C NMRchemical shifts (in ppm), obtained for both diastereoisomers of compound **5**, and experimental data of the isolated natural product.

Nuclei	δ_{calc} [7′*S*,7‴*R*,8′*R*]	δ_{calc} [7′*S*,7‴*S*,8′*R*]	δ_{exp}
**1**	116.1	116.3	124.2
**2**	110.1	102.2	109.1
**3**	135.5	136.7	149.3
**4**	137.0	135.3	143.2
**5**	136.8	135.3	147.0
**6**	119.4	119.9	125.3
**7**	124.9	127.5	135.0
**8**	121.5	121.5	127.1
**9**	163.4	162.9	170.0
**1′**	126.2	127.9	135.2
**2′,6′**	100.3	98.6	106.1
**3′,5′**	136.7	137.3	149.0
**4′**	126.0	124.5	135.3
**7′**	36.0	37.9	41.4
**8′**	47.7	49.0	50.1
**9′**	159.5	158.9	174.2
**1″**	117.8	120.1	131.4
**2″,6″**	121.9	122.3	130.8
**3″,5″**	107.4	107.7	116.2
**4″**	145.6	145.8	156.8
**7″**	28.7	31.8	35.6
**8″**	36.9	40.8	42.8
**1‴**	123.3	125.9	134.4
**2‴,6‴**	119.2	121.3	128.4
**3‴,5‴**	106.3	123.8	116.0
**4‴**	145.0	145.6	157.9
**7‴**	68.7	70.8	72.8
**8‴**	41.6	43.1	47.9
**3′-OCH$_3$**	52.4	51.5	56.7
**5′-OCH$_3$**	49.8	51.6	56.7
**3-OCH$_3$**	52.9	52.1	56.8
**5-OCH$_3$**	55.5	54.7	60.8

20 Monte Carlo/MMFF force field calculations and Boltzmann population.

Table S16. Relative energy values (in kcal.mol^{-1}) and Boltzmann population of the conformers obtained from the Monte Carlo/MMFF force field calculations for each stereoisomer considered for compound **1**.

Conformers	Relative energy values (kcal.mol^{-1})	Boltzmann population (%)
7''R*,7'''R*,8aS		
**1**	0.0000	8.5690
**2**	0.0209	8.2717
**3**	0.0209	8.2717
**4**	0.0209	8.2717
**5**	0.1650	6.4861
**6**	0.1650	6.4861
**7**	0.3273	4.9321
**8**	0.3273	4.9321
**9**	0.3459	4.7800
**10**	0.3459	4.7799
**11**	0.3615	4.6558
**12**	0.4595	3.9459
**13**	0.4945	3.7194
**14**	0.5574	3.3451
**15**	1.0433	1.4731
**16**	1.0433	1.4731
**17**	1.1197	1.2949
**18**	1.1429	1.2452
**19**	1.2035	1.1241
**20**	1.2035	1.1241
**21**	1.2553	1.0301
**22**	1.2553	1.0301
**23**	1.3343	0.9015
**24**	1.4165	0.7846
**25**	1.4165	0.7846
**26**	1.4191	0.7813
**27**	1.5008	0.6806
**28**	1.6659	0.5151
**29**	1.8394	0.3843
**30**	1.8853	0.3557
**31**	1.9914	0.2974
**32**	2.0386	0.2746
**33**	2.1857	0.2143
**34**	2.1909	0.2124
**35**	2.2144	0.2041
**36**	2.2621	0.1883
**37**	2.2718	0.1853
**38**	2.2718	0.1853
**39**	2.2820	0.1821
**40**	2.3211	0.1705
**41**	2.3881	0.1522
**42**	2.4206	0.1441
**43**	2.4947	0.1272
**44**	2.4947	0.1272
**45**	2.5237	0.1211
**46**	2.6646	0.0955

**47**	2.6761	0.0936
**48**	2.6783	0.0933
**49**	2.7046	0.0892
**50**	2.7251	0.0862
**51**	2.8813	0.0662
**52**	2.9062	0.0635
**53**	3.0604	0.0490
**54**	3.3676	0.0291
**55**	3.3801	0.0285
**56**	3.4850	0.0239
**57**	3.7031	0.0165
**58**	4.0012	0.0100
**59**	4.0098	0.0099
**60**	4.3094	0.0059
**61**	4.5103	0.0042
**62**	4.5104	0.0042
**63**	4.5996	0.0036
**64**	4.6958	0.0031
**65**	4.8275	0.0025
**66**	5.0226	0.0018
**67**	5.1306	0.0015
**68**	5.5265	0.0008
**69**	5.8014	0.0005
**70**	6.0228	0.0003
**71**	6.0292	0.0003
**72**	6.0784	0.0003
**73**	6.3552	0.0002
**74**	6.3930	0.0002
**75**	6.4049	0.0002
**76**	6.5492	0.0001
**77**	6.5593	0.0001
**78**	6.6731	0.0001
**79**	6.7380	0.0001
**80**	6.8455	0.0001
**81**	7.0074	0.0001
**82**	7.2899	0.0000
**83**	7.8838	0.0000
**84**	8.0769	0.0000
**85**	8.3550	0.0000
**86**	8.4226	0.0000
**87**	8.5591	0.0000
**88**	8.5966	0.0000
**89**	8.7835	0.0000
**90**	8.8769	0.0000
**91**	8.9235	0.0000
**92**	8.9418	0.0000
**93**	9.0179	0.0000
**94**	9.0651	0.0000
**95**	9.1651	0.0000
**96**	9.1950	0.0000
**97**	9.4030	0.0000
**98**	9.7442	0.0000
**99**	9.7557	0.0000
**100**	9.8814	0.0000
7′′S*,7′′′S*,8aS		
**1**	0.0000	0.5070
**2**	0.2357	0.3406
**3**	0.7660	0.1392

**4**	2.2771	0.0109
**5**	4.0911	0.0005
**6**	4.1949	0.0004
**7**	4.3348	0.0003
**8**	4.3611	0.0003
**9**	4.6933	0.0002
**10**	5.3188	0.0001
**11**	5.5829	0.0000
**12**	5.7793	0.0000
**13**	5.8108	0.0000
**14**	5.8557	0.0000
**15**	5.9016	0.0000
**16**	5.9063	0.0000
**17**	6.0269	0.0000
**18**	6.0399	0.0000
**19**	6.0619	0.0000
**20**	6.0686	0.0000
**21**	6.1820	0.0000
**22**	6.1903	0.0000
**23**	6.2597	0.0000
**24**	6.3062	0.0000
**25**	6.3076	0.0000
**26**	6.3247	0.0000
**27**	6.3474	0.0000
**28**	6.4593	0.0000
**29**	6.5036	0.0000
**30**	6.5567	0.0000
**31**	6.5767	0.0000
**32**	6.5838	0.0000
**33**	6.5841	0.0000
**34**	6.6344	0.0000
**35**	6.6880	0.0000
**36**	6.7029	0.0000
**37**	6.8126	0.0000
**38**	6.8721	0.0000
**39**	6.9561	0.0000
**40**	7.0296	0.0000
**41**	7.0479	0.0000
**42**	7.1392	0.0000
**43**	7.1733	0.0000
**44**	7.1765	0.0000
**45**	7.1773	0.0000
**46**	7.2686	0.0000
**47**	7.2988	0.0000
**48**	7.3906	0.0000
**49**	7.4024	0.0000
**50**	7.4029	0.0000
**51**	7.4817	0.0000
**52**	7.4869	0.0000
**53**	7.5580	0.0000
**54**	7.6024	0.0000
**55**	7.6347	0.0000
**56**	7.7017	0.0000
**57**	7.7797	0.0000
**58**	7.8838	0.0000
**59**	7.9804	0.0000
**60**	8.1489	0.0000
**61**	8.1489	0.0000

**62**	8.1589	0.0000
**63**	8.1803	0.0000
**64**	8.1906	0.0000
**65**	8.2355	0.0000
**66**	8.3627	0.0000
**67**	8.3654	0.0000
**68**	8.3905	0.0000
**69**	8.4071	0.0000
**70**	8.4459	0.0000
**71**	8.5094	0.0000
**72**	8.5094	0.0000
**73**	8.5302	0.0000
**74**	8.5506	0.0000
**75**	8.6679	0.0000
**76**	8.7056	0.0000
**77**	8.9559	0.0000
**78**	8.9661	0.0000
**79**	9.0165	0.0000
**80**	9.0853	0.0000
**81**	9.1243	0.0000
**82**	9.1441	0.0000
**83**	9.1766	0.0000
**84**	9.2273	0.0000
**85**	9.3385	0.0000
**86**	9.3644	0.0000
**87**	9.3947	0.0000
**88**	9.5655	0.0000
**89**	9.5848	0.0000
**90**	9.6017	0.0000
**91**	9.6368	0.0000
**92**	9.7162	0.0000
**93**	9.7164	0.0000
**94**	9.7294	0.0000
**95**	9.7823	0.0000
**96**	9.8809	0.0000
**97**	9.8945	0.0000
**98**	9.9546	0.0000
**99**	9.9939	0.0000
**100**	9.9939	0.0000
**7''*R***,7'''*S***,8a*S***		
**1**	0.0000	0.4785
**2**	0.1715	0.3583
**3**	1.6139	0.0314
**4**	1.7959	0.0231
**5**	1.8344	0.0216
**6**	1.8980	0.0194
**7**	1.9399	0.0181
**8**	1.9676	0.0173
**9**	2.1054	0.0137
**10**	3.0161	0.0029
**11**	3.0593	0.0027
**12**	3.1241	0.0025
**13**	3.2690	0.0019
**14**	3.4246	0.0015
**15**	3.4477	0.0014
**16**	3.4625	0.0014
**17**	3.5293	0.0012
**18**	3.8456	0.0007

**19**	4.3533	0.0003
**20**	4.4129	0.0003
**21**	4.4263	0.0003
**22**	4.7272	0.0002
**23**	4.9532	0.0001
**24**	4.9620	0.0001
**25**	4.9773	0.0001
**26**	5.2757	0.0001
**27**	5.2757	0.0001
**28**	5.4126	0.0001
**29**	5.4212	0.0001
**30**	5.4848	0.0000
**31**	5.4930	0.0000
**32**	5.4996	0.0000
**33**	5.6077	0.0000
**34**	5.6176	0.0000
**35**	5.6402	0.0000
**36**	5.6469	0.0000
**37**	5.7228	0.0000
**38**	5.7945	0.0000
**39**	5.8213	0.0000
**40**	5.9035	0.0000
**41**	5.9721	0.0000
**42**	6.0904	0.0000
**43**	6.2142	0.0000
**44**	6.2142	0.0000
**45**	6.2203	0.0000
**46**	6.3783	0.0000
**47**	6.3860	0.0000
**48**	6.4346	0.0000
**49**	6.5282	0.0000
**50**	6.5282	0.0000
**51**	6.5282	0.0000
**52**	6.5916	0.0000
**53**	6.6627	0.0000
**54**	6.9334	0.0000
**55**	6.9741	0.0000
**56**	6.9881	0.0000
**57**	7.2907	0.0000
**58**	7.4147	0.0000
**59**	7.4428	0.0000
**60**	7.4781	0.0000
**61**	7.5432	0.0000
**62**	7.5432	0.0000
**63**	7.6305	0.0000
**64**	7.7574	0.0000
**65**	7.7961	0.0000
**66**	7.8515	0.0000
**67**	7.8831	0.0000
**68**	7.9897	0.0000
**69**	8.0404	0.0000
**70**	8.1427	0.0000
**71**	8.2598	0.0000
**72**	8.3844	0.0000
**73**	8.4197	0.0000
**74**	8.5094	0.0000
**75**	8.5094	0.0000
**76**	8.5569	0.0000

**77**	8.5899	0.0000
**78**	8.6345	0.0000
**79**	8.6769	0.0000
**80**	8.6930	0.0000
**81**	8.7489	0.0000
**82**	8.7876	0.0000
**83**	8.9889	0.0000
**84**	9.0014	0.0000
**85**	9.0038	0.0000
**86**	9.1194	0.0000
**87**	9.1366	0.0000
**88**	9.1779	0.0000
**89**	9.1903	0.0000
**90**	9.2469	0.0000
**91**	9.2483	0.0000
**92**	9.2871	0.0000
**93**	9.2872	0.0000
**94**	9.3736	0.0000
**95**	9.4640	0.0000
**96**	9.4946	0.0000
**97**	9.4946	0.0000
**98**	9.5000	0.0000
**99**	9.6790	0.0000
**100**	9.9669	0.0000
7''S*,7'''R*,8aS		
**1**	0.0000	0.2449
**2**	0.1320	0.1960
**3**	0.6308	0.0845
**4**	0.6780	0.0780
**5**	1.1641	0.0343
**6**	1.1641	0.0343
**7**	1.2035	0.0321
**8**	1.2035	0.0321
**9**	1.2244	0.0310
**10**	1.2244	0.0310
**11**	1.2612	0.0292
**12**	1.2612	0.0292
**13**	1.4257	0.0221
**14**	1.5012	0.0194
**15**	1.5012	0.0194
**16**	1.9860	0.0086
**17**	1.9860	0.0086
**18**	2.0108	0.0082
**19**	2.0108	0.0082
**20**	2.0156	0.0082
**21**	2.0156	0.0082
**22**	2.0670	0.0075
**23**	2.0670	0.0075
**24**	2.2002	0.0060
**25**	2.2307	0.0057
**26**	2.7248	0.0025
**27**	3.3742	0.0008
**28**	3.4393	0.0007
**29**	3.4739	0.0007
**30**	3.9859	0.0003
**31**	4.5069	0.0001
**32**	4.6936	0.0001
**33**	4.9698	0.0001

**34**	4.9833	0.0001
**35**	4.9833	0.0001
**36**	5.0324	0.0001
**37**	5.0547	0.0000
**38**	5.3279	0.0000
**39**	5.4244	0.0000
**40**	5.4981	0.0000
**41**	5.4988	0.0000
**42**	5.6296	0.0000
**43**	5.6988	0.0000
**44**	5.9575	0.0000
**45**	5.9883	0.0000
**46**	6.2254	0.0000
**47**	6.3318	0.0000
**48**	6.3478	0.0000
**49**	6.3580	0.0000
**50**	6.4715	0.0000
**51**	6.4715	0.0000
**52**	6.5614	0.0000
**53**	6.7668	0.0000
**54**	6.9544	0.0000
**55**	7.0048	0.0000
**56**	7.1901	0.0000
**57**	7.3691	0.0000
**58**	7.7395	0.0000
**59**	7.8269	0.0000
**60**	7.8836	0.0000
**61**	7.9716	0.0000
**62**	8.0099	0.0000
**63**	8.1025	0.0000
**64**	8.2941	0.0000
**65**	8.3668	0.0000
**66**	8.3787	0.0000
**67**	8.3787	0.0000
**68**	8.4182	0.0000
**69**	8.4803	0.0000
**70**	8.5165	0.0000
**71**	8.5900	0.0000
**72**	8.6343	0.0000
**73**	8.7250	0.0000
**74**	8.7255	0.0000
**75**	8.7345	0.0000
**76**	8.7733	0.0000
**77**	8.8228	0.0000
**78**	8.8833	0.0000
**79**	8.9197	0.0000
**80**	8.9387	0.0000
**81**	8.9454	0.0000
**82**	8.9504	0.0000
**83**	8.9840	0.0000
**84**	9.0548	0.0000
**85**	9.1032	0.0000
**86**	9.1089	0.0000
**87**	9.1437	0.0000
**88**	9.1440	0.0000
**89**	9.1511	0.0000
**90**	9.2641	0.0000
**91**	9.3626	0.0000

**92**	9.5135	0.0000
**93**	9.5521	0.0000
**94**	9.5809	0.0000
**95**	9.5944	0.0000
**96**	9.6309	0.0000
**97**	9.8343	0.0000
**98**	9.8570	0.0000
**99**	9.9638	0.0000
**100**	9.9709	0.0000

Table S17. Relative energy values (in kcal.mol^{-1}) and Boltzmann population of the conformers obtained from the Monte Carlo/MMFF force field calculations for each stereoisomer considered for compound **2**.

Conformers	Relative energy values (kcal.mol^{-1})	Boltzmann population (%)
7S*,7′S*,7″S*,7‴R*,8S*,8′S*		
**1**	0.0000	33.0733
**2**	0.0000	33.0733
**3**	0.0000	33.0733
**4**	2.2981	0.6841
**5**	3.8945	0.0462
**6**	3.8945	0.0462
**7**	6.0466	0.0012
**8**	6.1484	0.0010
**9**	6.7018	0.0004
**10**	6.7018	0.0004
**11**	7.3384	0.0001
**12**	7.3420	0.0001
**13**	7.7160	0.0001
**14**	8.1906	0.0000
**15**	8.3709	0.0000
**16**	8.6442	0.0000
**17**	8.8499	0.0000
**18**	9.1383	0.0000
**19**	9.2595	0.0000
**20**	9.6297	0.0000
**21**	9.6824	0.0000
**22**	9.8625	0.0000
**23**	9.9175	0.0000
**24**	9.9527	0.0000
**25**	10.0108	0.0000
**26**	10.0780	0.0000
**27**	10.3156	0.0000
**28**	10.9182	0.0000
**29**	11.0984	0.0000
**30**	11.1585	0.0000
**31**	11.5216	0.0000
**32**	11.7046	0.0000
**33**	11.7425	0.0000
**34**	12.3711	0.0000
**35**	12.4805	0.0000
**36**	12.5573	0.0000
**37**	12.5825	0.0000
**38**	13.0508	0.0000
**39**	13.3534	0.0000
**40**	13.5627	0.0000
**41**	13.6613	0.0000
**42**	14.5160	0.0000
**43**	14.5310	0.0000
**44**	14.5759	0.0000
**45**	14.6425	0.0000
**46**	14.7525	0.0000
**47**	15.4807	0.0000
**48**	15.6262	0.0000
**49**	15.9843	0.0000

**50**	16.1476	0.0000
**51**	16.3640	0.0000
**52**	16.8362	0.0000
**53**	16.9262	0.0000
**54**	17.1464	0.0000
**55**	17.2401	0.0000
**56**	17.5103	0.0000
**57**	17.7214	0.0000
**58**	18.3201	0.0000
**59**	18.4538	0.0000
**60**	18.6685	0.0000
**61**	18.6686	0.0000
**62**	19.0129	0.0000
**63**	19.2383	0.0000
**64**	19.3026	0.0000
**65**	20.1032	0.0000
**66**	20.2629	0.0000
**67**	20.3339	0.0000
**68**	20.8890	0.0000
**69**	20.9855	0.0000
**70**	21.0685	0.0000
**71**	21.1572	0.0000
**72**	21.3028	0.0000
**73**	21.4586	0.0000
**74**	21.6990	0.0000
**75**	21.8724	0.0000
**76**	22.1640	0.0000
**77**	22.2880	0.0000
**78**	22.5256	0.0000
**79**	22.6523	0.0000
**80**	22.7300	0.0000
**81**	23.0933	0.0000
**82**	23.1548	0.0000
**83**	23.1925	0.0000
**84**	23.4465	0.0000
**85**	23.6105	0.0000
**86**	23.9486	0.0000
**87**	24.7109	0.0000
**88**	26.1529	0.0000
**89**	26.7070	0.0000
**90**	28.3617	0.0000
**91**	28.3682	0.0000
**92**	28.4097	0.0000
**93**	28.5713	0.0000
**94**	28.6873	0.0000
**95**	28.7357	0.0000
**96**	30.5853	0.0000
**97**	31.4692	0.0000
**98**	31.5103	0.0000
**99**	34.6216	0.0000
**100**	38.1970	0.0000
7S*,7′S*,7″R*,7‴S*,8S*,8′S*		
**1**	0.0000	99.2048
**2**	3.1036	0.5269
**3**	3.7332	0.1821
**4**	4.5128	0.0488
**5**	5.5730	0.0082
**6**	5.6671	0.0070

**7**	5.8661	0.0050
**8**	5.9651	0.0042
**9**	5.9878	0.0041
**10**	6.3251	0.0023
**11**	6.5329	0.0016
**12**	6.6221	0.0014
**13**	6.7507	0.0011
**14**	6.7719	0.0011
**15**	7.0083	0.0007
**16**	7.1128	0.0006
**17**	8.6949	0.0000
**18**	8.6986	0.0000
**19**	9.0675	0.0000
**20**	9.0748	0.0000
**21**	9.1269	0.0000
**22**	9.2703	0.0000
**23**	9.4095	0.0000
**24**	10.0989	0.0000
**25**	10.6310	0.0000
**26**	11.1979	0.0000
**27**	11.2705	0.0000
**28**	11.2706	0.0000
**29**	11.6894	0.0000
**30**	11.9173	0.0000
**31**	12.0665	0.0000
**32**	12.0665	0.0000
**33**	13.2429	0.0000
**34**	13.9489	0.0000
**35**	14.0532	0.0000
**36**	14.5127	0.0000
**37**	14.5500	0.0000
**38**	14.5500	0.0000
**39**	14.6209	0.0000
**40**	14.8192	0.0000
**41**	14.8228	0.0000
**42**	14.8479	0.0000
**43**	14.9307	0.0000
**44**	15.9085	0.0000
**45**	16.1337	0.0000
**46**	16.2588	0.0000
**47**	16.7345	0.0000
**48**	16.7938	0.0000
**49**	16.9281	0.0000
**50**	17.0139	0.0000
**51**	17.2849	0.0000
**52**	17.5994	0.0000
**53**	17.9474	0.0000
**54**	18.1482	0.0000
**55**	18.3550	0.0000
**56**	18.4008	0.0000
**57**	18.8640	0.0000
**58**	19.5154	0.0000
**59**	19.5587	0.0000
**60**	19.9580	0.0000
**61**	19.9585	0.0000
**62**	20.7516	0.0000
**63**	20.8207	0.0000
**64**	21.1796	0.0000

**65**	21.3844	0.0000
**66**	21.3973	0.0000
**67**	21.7080	0.0000
**68**	22.0108	0.0000
**69**	22.0898	0.0000
**70**	22.4763	0.0000
**71**	24.0812	0.0000
**72**	24.4194	0.0000
**73**	28.7728	0.0000
**74**	28.8256	0.0000
**75**	28.9203	0.0000
**76**	29.9774	0.0000
**77**	31.4242	0.0000
**78**	31.4361	0.0000
**79**	31.6444	0.0000
**80**	31.9760	0.0000
**81**	32.0791	0.0000
**82**	32.2635	0.0000
**83**	32.7862	0.0000
**84**	33.0537	0.0000
**85**	33.0681	0.0000
**86**	33.1837	0.0000
**87**	33.2172	0.0000
**88**	33.4049	0.0000
**89**	33.6821	0.0000
**90**	35.0170	0.0000
**91**	35.3334	0.0000
**92**	35.8367	0.0000
**93**	36.7811	0.0000
**94**	36.9757	0.0000
**95**	37.1403	0.0000
**96**	37.6950	0.0000
**97**	38.5137	0.0000
**98**	45.8392	0.0000
**99**	48.1850	0.0000
**100**	48.4624	0.0000
7S*,7′S*,7′′R*,7′′′R*,8S*,8′S*		
**1**	0.0000	17.2060
**2**	0.0000	17.2060
**3**	0.1854	12.5841
**4**	0.5260	7.0820
**5**	0.5260	7.0820
**6**	0.6418	5.8245
**7**	0.8343	4.2087
**8**	0.8343	4.2087
**9**	0.9364	3.5429
**10**	1.1342	2.5372
**11**	1.1342	2.5372
**12**	1.2482	2.0932
**13**	1.3044	1.9036
**14**	1.3122	1.8787
**15**	1.4268	1.5485
**16**	1.5093	1.3473
**17**	1.6752	1.0183
**18**	1.7109	0.9587
**19**	1.9086	0.6867
**20**	1.9582	0.6315
**21**	2.0098	0.5789

**22**	2.0208	0.5683
**23**	2.0896	0.5059
**24**	2.1861	0.4299
**25**	2.4009	0.2992
**26**	2.6647	0.1917
**27**	2.6791	0.1871
**28**	3.0750	0.0959
**29**	3.0913	0.0933
**30**	3.1356	0.0866
**31**	3.1735	0.0812
**32**	3.2582	0.0704
**33**	3.2582	0.0704
**34**	3.2862	0.0671
**35**	3.4086	0.0546
**36**	3.6292	0.0376
**37**	3.6591	0.0358
**38**	3.6726	0.0350
**39**	3.6772	0.0347
**40**	3.7223	0.0322
**41**	3.7263	0.0320
**42**	3.7506	0.0307
**43**	3.7851	0.0289
**44**	3.8065	0.0279
**45**	3.8228	0.0272
**46**	3.8469	0.0261
**47**	3.9025	0.0237
**48**	4.0025	0.0200
**49**	4.1504	0.0156
**50**	4.2431	0.0134
**51**	4.3138	0.0119
**52**	4.3801	0.0106
**53**	4.5164	0.0084
**54**	4.5222	0.0083
**55**	4.6074	0.0072
**56**	4.6558	0.0067
**57**	4.6720	0.0065
**58**	4.7008	0.0062
**59**	4.7136	0.0060
**60**	4.7777	0.0054
**61**	4.8986	0.0044
**62**	4.9426	0.0041
**63**	4.9434	0.0041
**64**	4.9434	0.0041
**65**	5.2115	0.0026
**66**	5.2377	0.0025
**67**	5.3140	0.0022
**68**	5.3351	0.0021
**69**	5.6559	0.0012
**70**	5.6842	0.0012
**71**	5.6842	0.0012
**72**	5.7859	0.0010
**73**	5.8367	0.0009
**74**	5.9189	0.0008
**75**	5.9307	0.0008
**76**	6.0950	0.0006
**77**	6.3718	0.0004
**78**	6.6375	0.0002
**79**	6.7636	0.0002

**80**	6.8120	0.0002
**81**	6.8535	0.0002
**82**	7.0124	0.0001
**83**	7.1298	0.0001
**84**	7.2250	0.0001
**85**	7.3355	0.0001
**86**	7.4099	0.0001
**87**	7.6127	0.0000
**88**	7.6418	0.0000
**89**	7.6704	0.0000
**90**	7.7269	0.0000
**91**	7.9015	0.0000
**92**	7.9193	0.0000
**93**	8.0118	0.0000
**94**	8.0827	0.0000
**95**	8.4418	0.0000
**96**	8.5330	0.0000
**97**	9.3188	0.0000
**98**	9.3838	0.0000
**99**	9.8595	0.0000
**100**	9.9848	0.0000
7S*,7′S*,7″S*,7‴S*,8S*,8′S*		
**1**	0.0000	34.0631
**2**	0.3462	18.9918
**3**	0.5899	12.5878
**4**	0.8730	7.8051
**5**	0.9178	7.2373
**6**	1.1296	5.0622
**7**	1.3614	3.4233
**8**	1.5178	2.6292
**9**	1.9374	1.2949
**10**	1.9535	1.2603
**11**	1.9535	1.2603
**12**	2.2358	0.7826
**13**	2.2796	0.7268
**14**	2.3001	0.7022
**15**	2.5982	0.4246
**16**	2.6188	0.4101
**17**	2.9096	0.2510
**18**	2.9248	0.2447
**19**	3.0450	0.1997
**20**	3.1567	0.1654
**21**	3.4223	0.1056
**22**	3.5775	0.0813
**23**	3.7401	0.0618
**24**	3.9252	0.0452
**25**	3.9507	0.0433
**26**	4.1727	0.0298
**27**	4.3700	0.0213
**28**	4.3701	0.0213
**29**	4.7690	0.0109
**30**	4.8707	0.0092
**31**	4.8707	0.0092
**32**	5.1156	0.0061
**33**	5.2038	0.0052
**34**	5.2759	0.0046
**35**	5.3476	0.0041
**36**	5.3476	0.0041

**37**	5.5131	0.0031
**38**	5.5853	0.0027
**39**	5.9041	0.0016
**40**	6.0998	0.0012
**41**	6.1286	0.0011
**42**	6.4258	0.0007
**43**	6.5823	0.0005
**44**	6.5901	0.0005
**45**	6.5968	0.0005
**46**	6.9547	0.0003
**47**	7.0770	0.0002
**48**	7.1739	0.0002
**49**	7.2017	0.0002
**50**	7.2479	0.0002
**51**	7.3289	0.0001
**52**	7.4301	0.0001
**53**	7.4332	0.0001
**54**	7.4417	0.0001
**55**	7.4699	0.0001
**56**	7.5034	0.0001
**57**	7.5038	0.0001
**58**	7.5473	0.0001
**59**	7.6497	0.0001
**60**	7.7486	0.0001
**61**	7.7859	0.0001
**62**	7.8215	0.0001
**63**	7.9647	0.0000
**64**	8.0631	0.0000
**65**	8.0674	0.0000
**66**	8.1469	0.0000
**67**	8.1719	0.0000
**68**	8.2715	0.0000
**69**	8.2827	0.0000
**70**	8.3292	0.0000
**71**	8.3300	0.0000
**72**	8.3441	0.0000
**73**	8.5853	0.0000
**74**	8.6069	0.0000
**75**	8.6689	0.0000
**76**	8.6708	0.0000
**77**	8.6882	0.0000
**78**	8.7274	0.0000
**79**	8.7383	0.0000
**80**	8.7771	0.0000
**81**	8.9583	0.0000
**82**	8.9877	0.0000
**83**	9.0802	0.0000
**84**	9.1610	0.0000
**85**	9.1669	0.0000
**86**	9.2127	0.0000
**87**	9.2951	0.0000
**88**	9.3543	0.0000
**89**	9.4714	0.0000
**90**	9.5032	0.0000
**91**	9.5395	0.0000
**92**	9.5966	0.0000
**93**	9.6446	0.0000
**94**	9.6781	0.0000

**95**	9.6820	0.0000
**96**	9.6861	0.0000
**97**	9.7356	0.0000
**98**	9.8756	0.0000
**99**	9.8835	0.0000
**100**	9.9630	0.0000

Table S18. Relative energy values (in kcal.mol^{-1}) and Boltzmann population of the conformers obtained from the Monte Carlo/MMFF force field calculations for each stereoisomer considered for compound **3**.

Conformers	Relative energy values (kcal.mol^{-1})	Boltzmann population (%)
	7S,7′S,7′′′S,8S,8′S	
**1**	0.0000	93.4641
**2**	2.2101	2.2427
**3**	2.4292	1.5493
**4**	2.8251	0.7942
**5**	3.1619	0.4499
**6**	3.4406	0.2811
**7**	3.6901	0.1845
**8**	3.7061	0.1796
**9**	3.7072	0.1792
**10**	3.9564	0.1177
**11**	3.9727	0.1145
**12**	4.5663	0.0420
**13**	4.5741	0.0415
**14**	4.6040	0.0395
**15**	4.6548	0.0362
**16**	4.8089	0.0279
**17**	4.8336	0.0268
**18**	4.9080	0.0236
**19**	4.9686	0.0213
**20**	4.9790	0.0210
**21**	5.0257	0.0194
**22**	5.2863	0.0125
**23**	5.2863	0.0125
**24**	5.2866	0.0125
**25**	5.3062	0.0121
**26**	5.4056	0.0102
**27**	5.4706	0.0091
**28**	5.6186	0.0071
**29**	5.6413	0.0069
**30**	5.8325	0.0050
**31**	5.8753	0.0046
**32**	6.0275	0.0036
**33**	6.0587	0.0034
**34**	6.0655	0.0033
**35**	6.0755	0.0033
**36**	6.1881	0.0027
**37**	6.1968	0.0027
**38**	6.1968	0.0027
**39**	6.2145	0.0026
**40**	6.2145	0.0026
**41**	6.3200	0.0022
**42**	6.3352	0.0021
**43**	6.3408	0.0021
**44**	6.3889	0.0019
**45**	6.4329	0.0018
**46**	6.4903	0.0016
**47**	6.5436	0.0015
**48**	6.7385	0.0011
**49**	6.8499	0.0009

**50**	6.8988	0.0008
**51**	6.9080	0.0008
**52**	7.0848	0.0006
**53**	7.0849	0.0006
**54**	7.1077	0.0006
**55**	7.1822	0.0005
**56**	7.2074	0.0005
**57**	7.2097	0.0005
**58**	7.2909	0.0004
**59**	7.2909	0.0004
**60**	7.2950	0.0004
**61**	7.3903	0.0004
**62**	7.5238	0.0003
**63**	7.5238	0.0003
**64**	7.5512	0.0003
**65**	7.6174	0.0002
**66**	7.6566	0.0002
**67**	7.7521	0.0002
**68**	7.8356	0.0002
**69**	7.8804	0.0002
**70**	7.9416	0.0001
**71**	8.0022	0.0001
**72**	8.1363	0.0001
**73**	8.2153	0.0001
**74**	8.2622	0.0001
**75**	8.3024	0.0001
**76**	8.3795	0.0001
**77**	8.4228	0.0001
**78**	8.4647	0.0001
**79**	8.5500	0.0001
**80**	8.6197	0.0000
**81**	8.6278	0.0000
**82**	8.8834	0.0000
**83**	8.9886	0.0000
**84**	9.1247	0.0000
**85**	9.2238	0.0000
**86**	9.2462	0.0000
**87**	9.3258	0.0000
**88**	9.3299	0.0000
**89**	9.4397	0.0000
**90**	9.5381	0.0000
**91**	9.5383	0.0000
**92**	9.6322	0.0000
**93**	9.6512	0.0000
**94**	9.6924	0.0000
**95**	9.7235	0.0000
**96**	9.7302	0.0000
**97**	9.8046	0.0000
**98**	9.8853	0.0000
**99**	9.9679	0.0000
**100**	9.9747	0.0000
7R,7′R,7′′′S,8R,8′R		
**1**	0.0000	45.6698
**2**	0.3853	23.8357
**3**	1.2617	5.4312
**4**	1.3594	4.6051
**5**	1.3594	4.6051
**6**	1.5808	3.1694

**7**	1.5808	3.1694
**8**	2.0553	1.4230
**9**	2.1040	1.3106
**10**	2.2164	1.0843
**11**	2.5184	0.6513
**12**	2.6302	0.5393
**13**	2.7112	0.4704
**14**	2.7350	0.4519
**15**	2.7398	0.4482
**16**	2.7922	0.4103
**17**	2.8275	0.3865
**18**	2.8275	0.3865
**19**	2.8427	0.3767
**20**	2.8427	0.3767
**21**	2.9988	0.2895
**22**	3.2842	0.1788
**23**	3.5732	0.1098
**24**	3.5812	0.1083
**25**	3.7437	0.0824
**26**	3.9931	0.0541
**27**	4.2081	0.0376
**28**	4.2501	0.0350
**29**	4.2562	0.0347
**30**	4.2562	0.0347
**31**	4.2719	0.0338
**32**	4.4534	0.0249
**33**	4.4673	0.0243
**34**	4.5087	0.0226
**35**	4.6836	0.0169
**36**	4.7168	0.0159
**37**	4.7275	0.0157
**38**	5.0125	0.0097
**39**	5.0192	0.0096
**40**	5.1081	0.0082
**41**	5.1387	0.0078
**42**	5.3062	0.0059
**43**	5.4626	0.0045
**44**	5.5313	0.0040
**45**	5.5313	0.0040
**46**	5.5471	0.0039
**47**	5.5975	0.0036
**48**	5.7320	0.0029
**49**	5.9323	0.0020
**50**	6.0895	0.0016
**51**	6.0929	0.0016
**52**	6.1755	0.0014
**53**	6.1854	0.0013
**54**	6.3700	0.0010
**55**	6.3862	0.0010
**56**	6.5408	0.0007
**57**	6.6489	0.0006
**58**	6.9507	0.0004
**59**	6.9740	0.0004
**60**	6.9951	0.0003
**61**	7.0454	0.0003
**62**	7.1378	0.0003
**63**	7.1663	0.0003
**64**	7.2179	0.0002

**65**	7.2517	0.0002
**66**	7.4042	0.0002
**67**	7.4079	0.0002
**68**	7.4919	0.0001
**69**	7.5502	0.0001
**70**	7.5502	0.0001
**71**	7.5675	0.0001
**72**	7.5675	0.0001
**73**	7.5675	0.0001
**74**	7.6641	0.0001
**75**	7.6828	0.0001
**76**	7.7473	0.0001
**77**	7.7473	0.0001
**78**	7.9727	0.0001
**79**	8.0519	0.0001
**80**	8.1326	0.0000
**81**	8.1663	0.0000
**82**	8.1826	0.0000
**83**	8.2414	0.0000
**84**	8.4790	0.0000
**85**	8.7227	0.0000
**86**	8.7690	0.0000
**87**	8.8022	0.0000
**88**	8.8107	0.0000
**89**	8.8768	0.0000
**90**	9.0088	0.0000
**91**	9.1232	0.0000
**92**	9.1565	0.0000
**93**	9.3744	0.0000
**94**	9.3814	0.0000
**95**	9.4500	0.0000
**96**	9.5003	0.0000
**97**	9.5575	0.0000
**98**	9.6430	0.0000
**99**	9.8197	0.0000
**100**	9.8594	0.0000

Table S19. Relative energy values (in kcal.mol^{-1}) and Boltzmann population of the conformers obtained from the Monte Carlo/MMFF force field calculations for each stereoisomer considered for compound **4**.

Conformers	Relative energy values (kcal.mol^{-1})	Boltzmann population (%)
7′S,7″R*,7‴R*,8′R**		
**1**	0.0000	24.8622
**2**	0.0109	24.4095
**3**	0.3115	14.6969
**4**	0.4198	12.2420
**5**	0.9989	4.6069
**6**	1.0647	4.1227
**7**	1.1904	3.3344
**8**	1.3652	2.4825
**9**	1.4071	2.3130
**10**	1.4150	2.2825
**11**	1.8529	1.0901
**12**	1.8868	1.0294
**13**	2.0824	0.7400
**14**	2.2984	0.5139
**15**	2.8728	0.1949
**16**	3.0202	0.1520
**17**	3.0584	0.1425
**18**	3.1862	0.1149
**19**	3.2599	0.1014
**20**	3.3441	0.0880
**21**	3.3920	0.0812
**22**	3.5290	0.0644
**23**	3.6000	0.0571
**24**	3.7820	0.0420
**25**	3.8571	0.0370
**26**	3.9192	0.0333
**27**	4.3471	0.0162
**28**	4.3752	0.0154
**29**	4.4399	0.0138
**30**	4.4818	0.0129
**31**	4.5525	0.0114
**32**	4.5763	0.0110
**33**	4.5884	0.0108
**34**	4.5971	0.0106
**35**	4.6440	0.0098
**36**	4.6731	0.0093
**37**	4.8513	0.0069
**38**	4.9302	0.0061
**39**	4.9598	0.0058
**40**	4.9921	0.0055
**41**	5.3387	0.0030
**42**	5.5421	0.0022
**43**	5.5995	0.0020
**44**	5.7484	0.0015
**45**	5.8858	0.0012
**46**	5.9637	0.0011
**47**	6.0079	0.0010
**48**	6.0634	0.0009
**49**	6.1413	0.0008

**50**	6.2065	0.0007
**51**	6.3531	0.0005
**52**	6.4410	0.0005
**53**	6.4437	0.0005
**54**	6.5028	0.0004
**55**	6.5073	0.0004
**56**	6.5279	0.0004
**57**	6.6079	0.0004
**58**	6.6727	0.0003
**59**	6.7552	0.0003
**60**	6.7994	0.0003
**61**	7.0076	0.0002
**62**	7.1417	0.0001
**63**	7.2206	0.0001
**64**	7.2346	0.0001
**65**	7.3881	0.0001
**66**	7.4542	0.0001
**67**	7.5734	0.0001
**68**	7.7071	0.0001
**69**	7.7086	0.0001
**70**	7.8340	0.0000
**71**	7.9632	0.0000
**72**	7.9636	0.0000
**73**	8.0311	0.0000
**74**	8.3795	0.0000
**75**	8.5033	0.0000
**76**	8.5120	0.0000
**77**	8.5628	0.0000
**78**	8.6727	0.0000
**79**	8.7387	0.0000
**80**	8.7426	0.0000
**81**	8.7742	0.0000
**82**	8.9077	0.0000
**83**	8.9327	0.0000
**84**	8.9377	0.0000
**85**	9.1200	0.0000
**86**	9.1363	0.0000
**87**	9.1550	0.0000
**88**	9.2015	0.0000
**89**	9.2037	0.0000
**90**	9.2052	0.0000
**91**	9.2936	0.0000
**92**	9.3931	0.0000
**93**	9.4454	0.0000
**94**	9.5483	0.0000
**95**	9.6348	0.0000
**96**	9.6994	0.0000
**97**	9.7597	0.0000
**98**	9.8769	0.0000
**99**	9.8842	0.0000
**100**	9.9142	0.0000
7′S*,7′′S*,7′′′S*,8′R*		
**1**	0.0000	56.0088
**2**	0.2614	36.0319
**3**	1.7922	2.7207
**4**	2.1548	1.4753
**5**	2.4012	0.9733
**6**	2.4365	0.9171

**7**	2.5959	0.7007
**8**	3.0530	0.3240
**9**	3.2102	0.2485
**10**	3.5725	0.1348
**11**	3.7663	0.0972
**12**	3.9754	0.0683
**13**	4.0267	0.0626
**14**	4.0858	0.0567
**15**	4.3970	0.0335
**16**	4.5998	0.0238
**17**	4.7650	0.0180
**18**	4.9199	0.0139
**19**	4.9510	0.0132
**20**	5.1189	0.0099
**21**	5.1955	0.0087
**22**	5.2143	0.0084
**23**	5.4465	0.0057
**24**	5.5200	0.0050
**25**	5.5806	0.0045
**26**	5.6504	0.0040
**27**	5.6768	0.0039
**28**	5.7312	0.0035
**29**	5.7637	0.0033
**30**	5.7747	0.0033
**31**	5.7949	0.0032
**32**	5.9811	0.0023
**33**	6.1064	0.0019
**34**	6.4036	0.0011
**35**	6.4637	0.0010
**36**	6.5122	0.0009
**37**	6.7455	0.0006
**38**	6.8541	0.0005
**39**	6.9215	0.0005
**40**	6.9450	0.0005
**41**	6.9644	0.0004
**42**	6.9940	0.0004
**43**	7.0324	0.0004
**44**	7.0576	0.0004
**45**	7.0947	0.0004
**46**	7.1803	0.0003
**47**	7.1985	0.0003
**48**	7.2113	0.0003
**49**	7.2321	0.0003
**50**	7.2550	0.0003
**51**	7.3784	0.0002
**52**	7.5479	0.0002
**53**	7.6693	0.0001
**54**	8.1248	0.0001
**55**	8.1672	0.0001
**56**	8.2146	0.0001
**57**	8.2449	0.0001
**58**	8.3623	0.0000
**59**	8.3803	0.0000
**60**	8.5087	0.0000
**61**	8.5188	0.0000
**62**	8.5240	0.0000
**63**	8.6031	0.0000
**64**	8.6288	0.0000

**65**	8.7202	0.0000
**66**	8.9103	0.0000
**67**	9.0109	0.0000
**68**	9.0827	0.0000
**69**	9.1258	0.0000
**70**	9.1506	0.0000
**71**	9.2411	0.0000
**72**	9.2795	0.0000
**73**	9.4952	0.0000
**74**	9.5413	0.0000
**75**	9.7095	0.0000
**76**	9.7196	0.0000
**77**	9.7741	0.0000
**78**	9.7983	0.0000
**79**	9.8566	0.0000
**80**	9.8613	0.0000
7'S*,7''R*,7'''S*,8'R*		
**1**	0.0000	64.3308
**2**	0.9094	13.8639
**3**	0.9644	12.6355
**4**	1.7393	3.4164
**5**	2.4308	1.0635
**6**	2.5702	0.8406
**7**	2.7034	0.6714
**8**	2.9460	0.4458
**9**	2.9685	0.4292
**10**	3.1754	0.3027
**11**	3.2117	0.2847
**12**	3.2629	0.2611
**13**	3.2630	0.2611
**14**	3.3421	0.2285
**15**	3.3566	0.2230
**16**	3.6683	0.1317
**17**	3.7594	0.1130
**18**	3.8860	0.0912
**19**	4.0840	0.0653
**20**	4.2149	0.0524
**21**	4.2903	0.0461
**22**	4.3299	0.0431
**23**	4.3570	0.0412
**24**	4.4171	0.0372
**25**	4.4822	0.0334
**26**	5.1807	0.0103
**27**	5.1991	0.0099
**28**	5.2811	0.0087
**29**	5.4376	0.0067
**30**	5.5235	0.0058
**31**	5.5872	0.0052
**32**	5.6240	0.0049
**33**	5.7164	0.0042
**34**	5.7211	0.0041
**35**	5.7646	0.0038
**36**	5.7964	0.0036
**37**	5.8313	0.0034
**38**	5.9685	0.0027
**39**	6.3097	0.0015
**40**	6.3959	0.0013
**41**	6.4290	0.0012

**42**	6.5043	0.0011
**43**	6.6171	0.0009
**44**	6.6307	0.0009
**45**	6.6495	0.0009
**46**	6.8270	0.0006
**47**	6.8736	0.0006
**48**	7.0394	0.0004
**49**	7.1326	0.0004
**50**	7.1758	0.0004
**51**	7.1788	0.0004
**52**	7.4115	0.0002
**53**	7.4502	0.0002
**54**	7.4569	0.0002
**55**	7.4781	0.0002
**56**	7.5029	0.0002
**57**	7.5170	0.0002
**58**	7.5430	0.0002
**59**	7.6001	0.0002
**60**	7.6111	0.0002
**61**	7.6305	0.0002
**62**	7.6903	0.0001
**63**	7.7841	0.0001
**64**	7.8527	0.0001
**65**	7.9054	0.0001
**66**	7.9282	0.0001
**67**	8.0711	0.0001
**68**	8.1066	0.0001
**69**	8.1156	0.0001
**70**	8.1639	0.0001
**71**	8.2136	0.0001
**72**	8.2169	0.0001
**73**	8.3008	0.0001
**74**	8.4011	0.0000
**75**	8.4253	0.0000
**76**	8.5681	0.0000
**77**	8.5939	0.0000
**78**	8.6055	0.0000
**79**	8.6981	0.0000
**80**	8.7472	0.0000
**81**	8.7772	0.0000
**82**	8.7919	0.0000
**83**	8.9574	0.0000
**84**	8.9646	0.0000
**85**	9.0368	0.0000
**86**	9.1555	0.0000
**87**	9.1973	0.0000
**88**	9.2466	0.0000
**89**	9.3435	0.0000
**90**	9.4750	0.0000
**91**	9.6051	0.0000
**92**	9.6169	0.0000
**93**	9.6331	0.0000
**94**	9.6886	0.0000
**95**	9.7468	0.0000
**96**	9.7896	0.0000
**97**	9.8717	0.0000
**98**	9.8973	0.0000
**99**	9.9292	0.0000

**100**	9.9508	0.0000
7′S*,7″S*,7‴R*,8′R*		
**1**	0.0000	57.8901
**2**	0.9767	11.1352
**3**	1.0667	9.5671
**4**	1.1819	7.8763
**5**	1.2315	7.2443
**6**	1.9992	1.9829
**7**	2.3408	1.1141
**8**	2.5656	0.7624
**9**	2.7902	0.5218
**10**	2.9697	0.3854
**11**	3.0053	0.3629
**12**	3.4159	0.1815
**13**	3.4842	0.1617
**14**	3.5163	0.1532
**15**	3.5769	0.1383
**16**	3.6983	0.1127
**17**	3.7353	0.1059
**18**	3.8656	0.0850
**19**	4.2523	0.0442
**20**	4.4831	0.0300
**21**	4.5659	0.0261
**22**	4.6265	0.0235
**23**	4.7267	0.0199
**24**	4.8916	0.0150
**25**	5.2289	0.0085
**26**	5.5190	0.0052
**27**	5.5634	0.0048
**28**	5.6094	0.0045
**29**	5.6509	0.0042
**30**	5.7216	0.0037
**31**	5.7950	0.0033
**32**	5.8390	0.0030
**33**	5.8407	0.0030
**34**	5.9290	0.0026
**35**	5.9401	0.0026
**36**	5.9962	0.0023
**37**	6.0051	0.0023
**38**	6.4700	0.0010
**39**	6.4908	0.0010
**40**	6.5136	0.0010
**41**	6.5886	0.0009
**42**	6.6839	0.0007
**43**	6.8322	0.0006
**44**	6.8809	0.0005
**45**	6.8996	0.0005
**46**	6.9425	0.0005
**47**	7.0989	0.0004
**48**	7.1627	0.0003
**49**	7.1987	0.0003
**50**	7.2185	0.0003
**51**	7.2973	0.0003
**52**	7.3556	0.0002
**53**	7.4198	0.0002
**54**	7.4930	0.0002
**55**	7.4950	0.0002
**56**	7.6026	0.0002

**57**	7.6716	0.0001
**58**	7.7197	0.0001
**59**	7.8214	0.0001
**60**	8.0874	0.0001
**61**	8.0932	0.0001
**62**	8.1500	0.0001
**63**	8.3858	0.0000
**64**	8.7093	0.0000
**65**	8.7144	0.0000
**66**	8.8055	0.0000
**67**	8.8058	0.0000
**68**	8.8721	0.0000
**69**	8.9009	0.0000
**70**	8.9061	0.0000
**71**	8.9288	0.0000
**72**	8.9609	0.0000
**73**	9.0619	0.0000
**74**	9.0775	0.0000
**75**	9.0848	0.0000
**76**	9.1418	0.0000
**77**	9.3026	0.0000
**78**	9.3843	0.0000
**79**	9.5775	0.0000
**80**	9.5917	0.0000
**81**	9.8066	0.0000
**82**	9.8473	0.0000
**83**	9.8604	0.0000
**84**	9.8795	0.0000
**85**	9.9233	0.0000
**86**	9.9261	0.0000
**87**	9.9705	0.0000
**88**	9.9786	0.0000
**89**	9.9795	0.0000
**90**	9.9829	0.0000

Table S20. Relative energy values (in kcal.mol^{-1}) and Boltzmann population of the conformers obtained from the Monte Carlo/MMFF force field calculations for each stereoisomer considered for compound **5**.

Conformers	Relative energy values (kcal.mol^{-1})	Boltzmann population (%)
7'S*,7'''R*,8'R*		
**1**	0.0000	51.7000
**2**	0.4400	24.5000
**3**	1.0100	9.4000
**4**	1.4441	4.5177
**5**	1.7956	2.4962
**6**	1.8952	2.1097
**7**	2.2652	1.1300
**8**	2.5023	0.7573
**9**	2.5161	0.7399
**10**	2.6006	0.6415
**11**	2.6657	0.5748
**12**	3.0149	0.3189
**13**	3.1620	0.2487
**14**	3.2030	0.2321
**15**	3.3872	0.1701
**16**	3.7637	0.0901
**17**	3.8550	0.0772
**18**	3.8865	0.0732
**19**	4.2460	0.0399
**20**	4.2599	0.0390
**21**	4.2858	0.0373
**22**	4.3234	0.0350
**23**	4.5841	0.0226
**24**	4.8372	0.0147
**25**	4.9108	0.0130
**26**	4.9193	0.0128
**27**	4.9221	0.0128
**28**	4.9812	0.0115
**29**	5.0384	0.0105
**30**	5.0845	0.0097
**31**	5.1771	0.0083
**32**	5.1847	0.0082
**33**	5.3240	0.0065
**34**	5.3296	0.0064
**35**	5.4538	0.0052
**36**	5.5003	0.0048
**37**	5.8767	0.0025
**38**	5.9147	0.0024
**39**	6.0295	0.0020
**40**	6.0525	0.0019
**41**	6.1628	0.0016
**42**	6.2281	0.0014
**43**	6.3183	0.0012
**44**	6.4923	0.0009
**45**	6.5525	0.0008
**46**	6.6361	0.0007
**47**	6.6393	0.0007
**48**	6.6808	0.0007
**49**	6.7461	0.0006

**50**	6.7819	0.0006
**51**	6.7989	0.0005
**52**	6.8353	0.0005
**53**	6.8577	0.0005
**54**	6.9101	0.0004
**55**	6.9671	0.0004
**56**	7.0158	0.0004
**57**	7.1100	0.0003
**58**	7.2216	0.0003
**59**	7.3088	0.0002
**60**	7.3698	0.0002
**61**	7.5946	0.0001
**62**	7.6327	0.0001
**63**	7.6717	0.0001
**64**	7.7084	0.0001
**65**	7.7729	0.0001
**66**	8.0619	0.0001
**67**	8.0874	0.0001
**68**	8.1578	0.0001
**69**	8.2122	0.0000
**70**	8.2508	0.0000
**71**	8.2653	0.0000
**72**	8.3414	0.0000
**73**	8.3846	0.0000
**74**	8.3972	0.0000
**75**	8.4325	0.0000
**76**	8.4503	0.0000
**77**	8.4770	0.0000
**78**	8.6443	0.0000
**79**	8.6901	0.0000
**80**	8.6980	0.0000
**81**	8.6992	0.0000
**82**	8.7203	0.0000
**83**	8.8273	0.0000
**84**	8.9041	0.0000
**85**	9.0070	0.0000
**86**	9.0342	0.0000
**87**	9.1554	0.0000
**88**	9.1793	0.0000
**89**	9.2959	0.0000
**90**	9.3506	0.0000
**91**	9.3724	0.0000
**92**	9.4083	0.0000
**93**	9.4176	0.0000
**94**	9.4873	0.0000
**95**	9.5743	0.0000
**96**	9.6294	0.0000
**97**	9.6934	0.0000
**98**	9.8254	0.0000
**99**	9.8608	0.0000
**100**	9.9165	0.0000
7'S*,7'''S*,8'R*		
**1**	0.0000	25.3914
**2**	0.1490	19.7452
**3**	0.3329	14.4765
**4**	0.6771	8.0985
**5**	0.9235	5.3436
**6**	0.9785	4.8697

**7**	1.2449	3.1064
**8**	1.3005	2.8280
**9**	1.4830	2.0785
**10**	1.6045	1.6929
**11**	1.6115	1.6730
**12**	1.6601	1.5414
**13**	1.7253	1.3808
**14**	1.8126	1.1915
**15**	1.8733	1.0757
**16**	1.9674	0.9176
**17**	2.0426	0.8083
**18**	2.1373	0.6889
**19**	2.2582	0.5617
**20**	2.4768	0.3885
**21**	2.4811	0.3856
**22**	2.5751	0.3291
**23**	2.6042	0.3133
**24**	2.7118	0.2612
**25**	3.2046	0.1137
**26**	3.3556	0.0881
**27**	3.4066	0.0809
**28**	3.5774	0.0606
**29**	3.6280	0.0557
**30**	3.7681	0.0439
**31**	3.8684	0.0371
**32**	3.9034	0.0350
**33**	3.9081	0.0347
**34**	3.9391	0.0329
**35**	4.0815	0.0259
**36**	4.0818	0.0259
**37**	4.1263	0.0240
**38**	4.2032	0.0211
**39**	4.2056	0.0210
**40**	4.2793	0.0185
**41**	4.2871	0.0183
**42**	4.3805	0.0156
**43**	4.4862	0.0131
**44**	4.5280	0.0122
**45**	4.5424	0.0119
**46**	4.5538	0.0117
**47**	4.6212	0.0104
**48**	4.9883	0.0056
**49**	5.1727	0.0041
**50**	5.3095	0.0033
**51**	5.3838	0.0029
**52**	5.3993	0.0028
**53**	5.4604	0.0025
**54**	5.4629	0.0025
**55**	5.4718	0.0025
**56**	5.5877	0.0020
**57**	5.6744	0.0018
**58**	5.6938	0.0017
**59**	5.7442	0.0016
**60**	5.7977	0.0014
**61**	5.8084	0.0014
**62**	6.0730	0.0009
**63**	6.2302	0.0007
**64**	6.3006	0.0006

**65**	6.3565	0.0006
**66**	6.6612	0.0003
**67**	6.9510	0.0002
**68**	6.9699	0.0002
**69**	7.2123	0.0001
**70**	7.2404	0.0001
**71**	7.2980	0.0001
**72**	7.3075	0.0001
**73**	7.6532	0.0001
**74**	7.7004	0.0001
**75**	7.7029	0.0001
**76**	7.7462	0.0001
**77**	7.7614	0.0001
**78**	7.7825	0.0001
**79**	7.9328	0.0000
**80**	7.9440	0.0000
**81**	8.0832	0.0000
**82**	8.1518	0.0000
**83**	8.4283	0.0000
**84**	8.4395	0.0000
**85**	8.4572	0.0000
**86**	8.4659	0.0000
**87**	8.5106	0.0000
**88**	8.6083	0.0000
**89**	8.7127	0.0000
**90**	9.1970	0.0000
**91**	9.3264	0.0000
**92**	9.3273	0.0000
**93**	9.3463	0.0000
**94**	9.3766	0.0000
**95**	9.5612	0.0000
**96**	9.5912	0.0000
**97**	9.6797	0.0000
**98**	9.8304	0.0000
**99**	9.8584	0.0000
**100**	9.9324	0.0000

Table S21. Relative energy values (in kcal.mol^{-1}) and Boltzmann population of the conformers obtained from the Monte Carlo/MMFF force field calculations for each stereoisomer considered for compound **6**.

Conformers	Relative energy values (kcal.mol^{-1})	Boltzmann population (%)
	7′R	
**1**	0.0000	0.2690
**2**	0.0538	0.2456
**3**	0.4760	0.1205
**4**	0.6309	0.0927
**5**	0.8314	0.0661
**6**	1.0000	0.0497
**7**	1.5445	0.0198
**8**	1.6442	0.0168
**9**	1.6716	0.0160
**10**	1.6992	0.0153
**11**	1.7092	0.0150
**12**	1.8463	0.0119
**13**	1.8593	0.0117
**14**	2.1331	0.0073
**15**	2.1724	0.0069
**16**	2.1846	0.0067
**17**	2.3074	0.0055
**18**	2.5704	0.0035
**19**	2.5909	0.0034
**20**	2.6865	0.0029
**21**	3.0285	0.0016
**22**	3.0893	0.0015
**23**	3.2275	0.0012
**24**	3.2496	0.0011
**25**	3.2673	0.0011
**26**	3.3606	0.0009
**27**	3.4511	0.0008
**28**	3.4982	0.0007
**29**	3.5160	0.0007
**30**	3.5471	0.0007
**31**	3.6767	0.0005
**32**	3.7859	0.0005
**33**	4.0558	0.0003
**34**	4.0778	0.0003
**35**	4.3343	0.0002
**36**	4.3717	0.0002
**37**	4.4474	0.0001
**38**	4.4744	0.0001
**39**	4.5128	0.0001
**40**	4.5472	0.0001
**41**	4.5742	0.0001
**42**	4.6828	0.0001
**43**	4.7409	0.0001
**44**	5.0291	0.0001
**45**	5.0291	0.0001
**46**	5.0293	0.0001
**47**	5.0971	0.0000
**48**	5.1490	0.0000
**49**	5.1538	0.0000

**50**	5.4576	0.0000
**51**	5.5053	0.0000
**52**	5.5960	0.0000
**53**	5.7592	0.0000
**54**	5.7837	0.0000
**55**	5.8344	0.0000
**56**	6.0552	0.0000
**57**	6.1319	0.0000
**58**	6.1492	0.0000
**59**	6.3266	0.0000
**60**	6.4406	0.0000
**61**	6.4466	0.0000
**62**	6.4894	0.0000
**63**	6.5707	0.0000
**64**	6.7058	0.0000
**65**	6.8110	0.0000
**66**	6.9090	0.0000
**67**	7.1094	0.0000
**68**	7.2290	0.0000
**69**	7.3039	0.0000
**70**	7.3229	0.0000
**71**	7.3554	0.0000
**72**	7.4127	0.0000
**73**	7.4590	0.0000
**74**	7.5309	0.0000
**75**	7.6418	0.0000
**76**	7.7912	0.0000
**77**	7.8582	0.0000
**78**	7.9293	0.0000
**79**	7.9517	0.0000
**80**	8.0039	0.0000
**81**	8.0918	0.0000
**82**	8.1581	0.0000
**83**	8.1661	0.0000
**84**	8.2054	0.0000
**85**	8.2466	0.0000
**86**	8.2866	0.0000
**87**	8.3273	0.0000
**88**	8.3559	0.0000
**89**	8.5322	0.0000
**90**	8.6223	0.0000
**91**	8.7758	0.0000
**92**	8.8972	0.0000
**93**	9.2438	0.0000
**94**	9.4383	0.0000
**95**	9.6274	0.0000
**96**	9.6981	0.0000
**97**	9.7441	0.0000
**98**	9.7493	0.0000
**99**	9.8048	0.0000
**100**	9.9038	0.0000

Table S22. Boltzmann population of the conformers considered in the final simulated ECD spectra of compounds **1-6**.

Conformers	Boltzmann population (%)	Total population (%)
**Compound 1**		
1	36.60	100.00
2	34.83	
3	27.80	
4	0.77	
**Compound 2**		
1	52.35	99.47
2	28.93	
3	8.28	
4	7.22	
5	2.69	
**Compound 3**		
1	50.15	99.55
2	28.46	
3	14.26	
4	4.90	
5	1.78	
**Compound 4**		
1	29.29	99.91
2	19.25	
3	11.05	
4	10.68	
5	10.32	
6	9.17	
7	4.92	
8	3.38	
9	1.85	
**Compound 5**		
1	51.42	99.10
2	16.91	
3	8.66	
4	4.47	
5	4.33	
6	1.70	
7	1.56	
8	1.54	
9	1.44	
10	1.41	
11	1.29	
12	1.28	
13	1.02	
14	0.78	
15	0.66	
16	0.34	
17	0.29	
**Compound 6**		
1	9.21	99.97
2	7.38	
3	7.37	
4	7.36	
5	5.45	
6	5.27	

7	5.26	
8	4.78	
9	4.58	
10	4.56	
11	3.79	
12	3.73	
13	3.35	
14	3.33	
15	3.05	
16	2.72	
17	2.45	
18	2.43	
19	2.40	
20	2.24	
21	2.17	
22	1.96	
23	1.81	
24	1.37	
25	1.34	
26	0.16	
27	0.15	
28	0.11	
29	0.10	
30	0.09	
